# Supplementary material for: Mandibular form and function is more disparate in amniotes than in non-amniote tetrapods from the late Palaeozoic
Source: PeerJ. 2025 Nov 26;13:e20243. doi: 10.7717/peerj.20243 (PMC12664332; doi:10.7717/peerj.20243)
Supplement: Supplemental Information 2 [file peerj-13-20243-s002.docx]

SUPPLEMENTARY FILE 2 Description of measurements

**Anterior, posterior and opening mechanical advantage (characters 1-3).**

Three continuous characters are related to mechanical advantage, a commonly used measurement in biomechanical studies across gnathostome jaws (e.g., fishes: Westneat 2003; Kammerer et al. 2006; Anderson 2009; Anderson et al. 2011; early tetrapods: Anderson et al. 2013; lissamphibians: Emerson 1985; squamates: Stayton 2006; turtles: Ponstein et al. 2024; archosaurs: Rayfield et al. 2007; Sakamoto 2010; Stubbs et al. 2013; Mallon and Anderson 2015; Nabavizadeh 2016; MacLaren et al. 2017; Navalón et al. 2018; Ma et al. 2020, 2022; Singh et al. 2021, 2024; Meade and Ma, 2022; Johnson et al. 2022; synapsids: Maynard Smith and Savage 1959; Benevento et al. 2019; Singh et al. 2021, 2024; Morales-García et al. 2021). Mechanical advantage measurements are derived from the interpretation of the mandible as a lever, in which the mandible rotates around a fulcrum (i.e., the jaw joint) and has an input moment arm that characterizes the muscle insertion site with which movement is achieved, and an output moment arm on which the movement is exerted (Westneat 1994, 2003). The ratio of input and output moment arms (i.e., in-lever divided by out-lever) generally determines the efficiency by which an input (=muscle) force is converted into an output force (=movement). Lower values of mechanical advantage signify a faster but less powerful action than higher values (Westneat 1994, 2003). The relative position of the fulcrum to the input and output moment arms and the relative lengths of the latter determine the type of lever. In a third-order lever, which is commonly applicable to gnathostome jaws (Stayton 2006; Anderson 2009; Anderson et al. 2011, 2013; Benevento et al. 2019; Singh et al. 2021), the fulcrum is posterior to the moment arms and the input moment arm is shorter than the output moment arm. In a second-order lever, the relative position of the fulcrum remains the same, but the output moment arm is shorter than the input moment arm resulting in a mechanical advantage greater than 1, so that bite force exceeds muscle force. This has been measured in the posterior bite of herbivorous hadrosaurid and ceratopsid dinosaurs (MacLaren et al. 2017) and various ecologically diverse extant turtles (Ponstein et al. 2024).


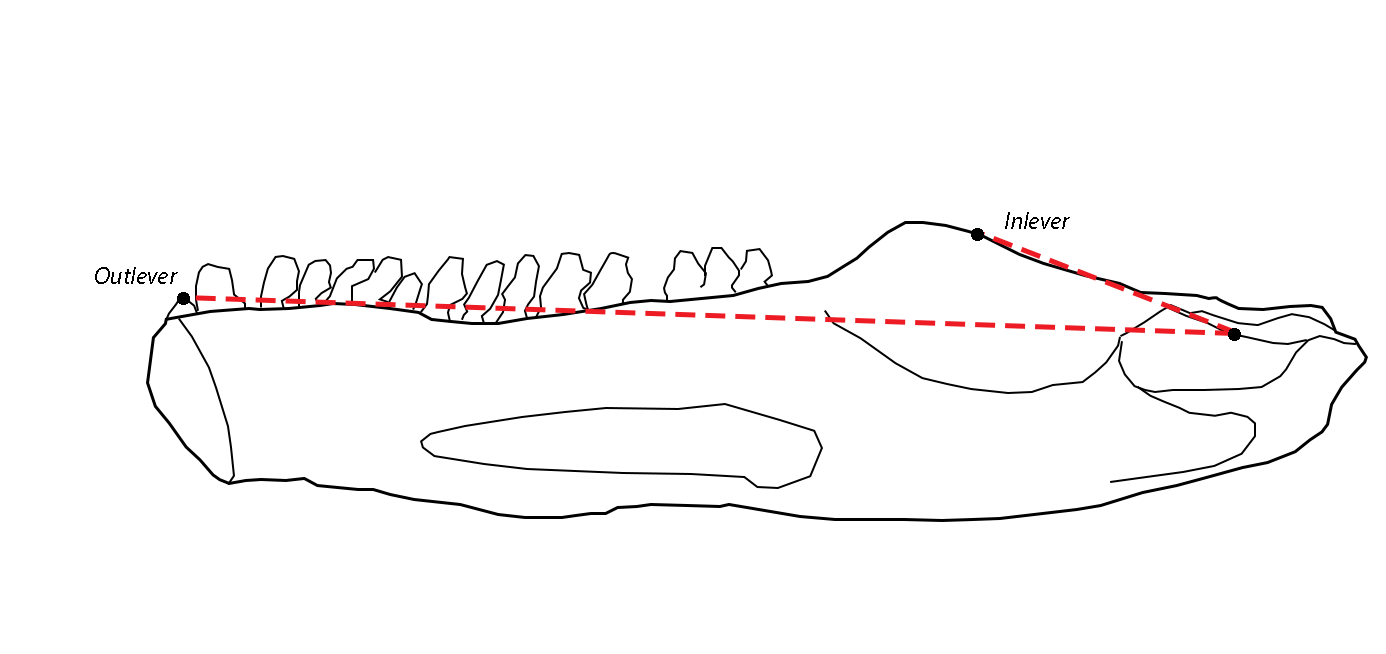


**Measurements required for Anterior Mechanical Advantage**. Mandible drawing from *Diadectes absitus* MNG 14473

We use anterior and posterior mechanical advantage as measurements that characterize the force transmission of the jaw adductor musculature to anterior and posterior bites, respectively. In both, the in-lever is defined as the distance between the fulcrum (i.e., jaw joint) and the point of muscle action. We use the centre of the articular surface of the jaw joint as the starting point for all measurements that involve this structure. The point of muscle action is measured at the midpoint of the adductor fossa, as this is an easily identifiable and homologous structure among most tetrapod jaws (e.g., Romer 1956). Consequently, we quantify specifically the force transmission of the adductor fossa musculature. Various neomorphic adductor muscles insert laterally on the dentary in derived therapsid clades, in addition to the adductor fossa musculature (e.g., Crompton and Hotton 1967; King 1981; Cox 1998; Kemp 2009; Lautenschlager et al. 2017). These neomorphic muscles are not homologous with the jaw adductor musculature in other tetrapods. Since our study primarily focuses on the comparison between amniotes and non-amniote tetrapods, we do not consider these neomorphics. The out-lever in anterior mechanical advantage is the distance between the jaw joint and the anteriormost tooth. This includes procumbent teeth that extend anteriorly to the anteriormost point of the jaw, as in *Orobates pabsti*. The out-lever in posterior mechanical advantage is measured as the distance between the jaw joint and the posteriormost tooth. We do not consider fields of minute denticles, as these are not used in mastication. Some taxa bear large fangs on the coronoid or extensive dental fields on the prearticular dental fields, such as in *Anthracosaurus russelli* (Panchen 1981) and *Edaphosaurus boanerges* (Modesto 1995), respectively. We do consider these teeth in determining the most posterior tooth, as these structures are clearly used for food processing. In some specimens not all teeth are preserved or figured, which may underestimate posterior mechanical advantage in these specimens. Summarizing, anterior mechanical advantage specifies an ‘anterior bite’ (resulting in a minimum possible value for mechanical advantage), whereas the posterior mechanical advantage specifies a ‘posterior bite’ (resulting in a maximum possible value for mechanical advantage). All distances are measured as straight lines along the sagittal plane. In modern clades, herbivores generally display a higher mechanical advantage than non-herbivorous relatives (e.g., Maynard-Smith and Savage 1959; Stayton 2006; Navalón et al. 2018; Grossnickle et al. 2020; Ponstein et al. 2024). Jaw-closing speed is less relevant to herbivores and a stronger bite is helpful in cutting through tougher vegetation.


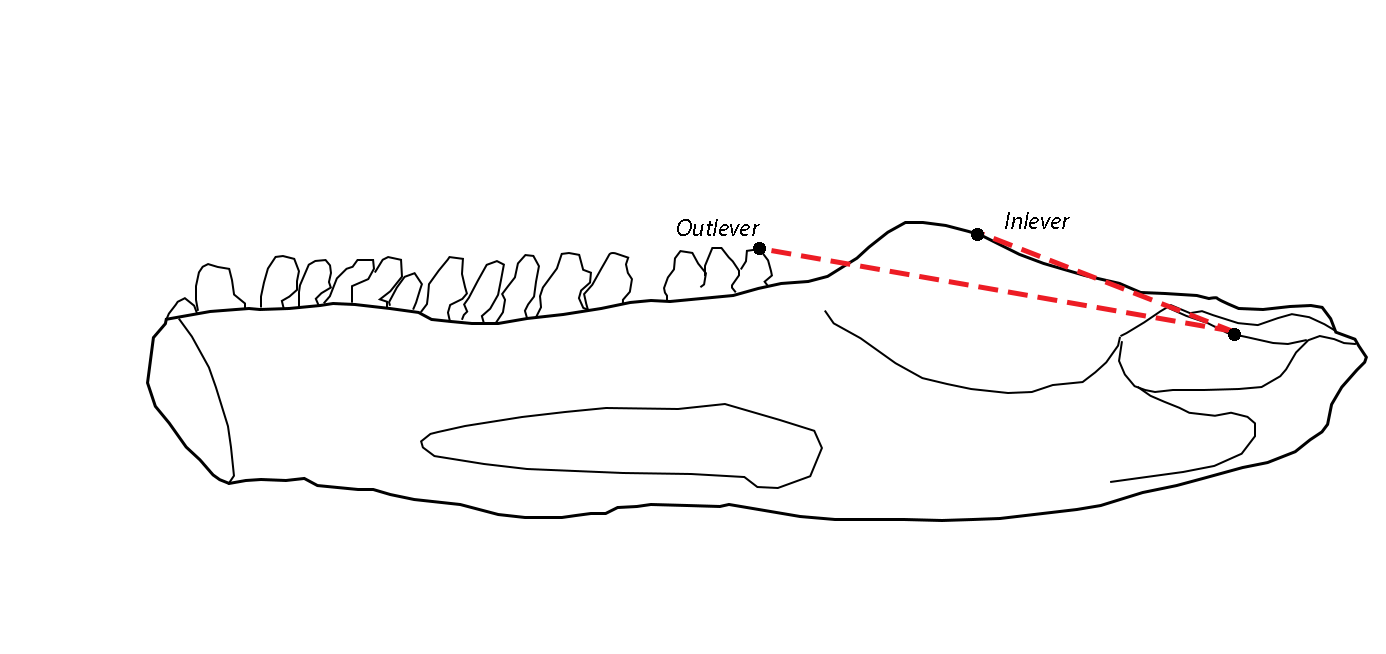


**Measurements required for Posterior Mechanical Advantage**. Mandible drawing from *Diadectes absitus* MNG 14473

We use jaw opening mechanical advantage as a proxy for velocity of jaw opening (Westneat 1994). Jaw opening mechanics in gnathostomes can be modelled as a first-order lever, in which the fulcrum (i.e., jaw joint) is positioned between the input and output moment arms (Westneat 1994). The input moment arm is the muscle insertion site for the jaw depressor muscles, which attach to the posteroventral end of the mandible, at the retroarticular process, but posterior to the position of the jaw joint. Thus, we represented the depressor musculature attachment site by the distance between the jaw joint and the posteriormost point of the jaw. The output moment arm is the part of the mandible that is rotated around the fulcrum and can thus be measured as the distance between the jaw joint and the anteriormost point of the mandible (or anteriormost tooth position as a functional equivalent). Jaw opening mechanical advantage is then again calculated by dividing the input moment arm by the output moment arm, and low values correspond to fast jaw opening movement and vice versa (MacLaren et al. 2017; Ma et al. 2020). As a result, herbivores are more likely to have higher jaw opening mechanical advantage values than carnivorous relatives (Ma et al. 2020).


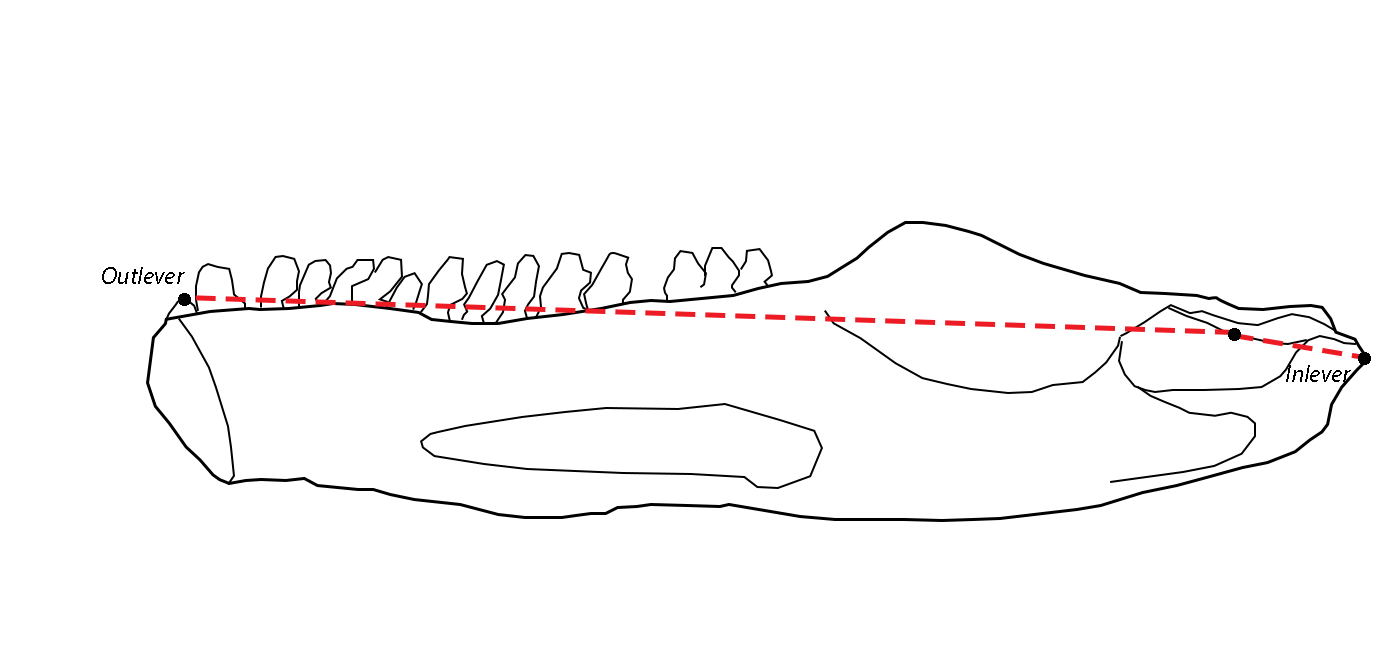


**Measurements required for Opening Mechanical Advantage**. Mandible drawing from *Diadectes absitus* MNG 14473

**Maximum and average aspect ratio (characters 4-5).**

Flexural stiffness of the mandible is an important mechanical property that describes the resistance of an object to deformation caused by bending (Wainwright et al. 1976). Stiffness quantifies how much force is required to deform an object by a unit distance, but this cannot be measured directly from fossils. Stiffness is approximated by measuring the cross-sectional area of a beam (or jaw). Since the majority of jaws measured in this study are figured in mediolateral view rather than anteroposterior view, aspect ratio is chosen as a proxy for flexural stiffness (Anderson et al. 2011, 2013; Stubbs and Benton 2016; MacLaren et al. 2017; Benevento et al. 2019; Singh et al. 2021, 2024). Maximum aspect ratio is measured as the deepest cross-section of the jaw divided by jaw length. Some taxa boast extremely tall coronoid processes like *Belebey vegrandis* (Reisz et al. 2007) or deep ventral angular bosses like derived pareiasaurs (e.g., Tsuji 2013; Van den Brandt et al. 2021), resulting in a maximum depth that is much greater than the rest of the jaw. Therefore, this metric may overestimate stiffness in these groups. We additionally measure the average aspect ratio, measured as the surface area of the jaw in medial view divided by length twice. Gnathostomes that exhibit high bite force or orally process tougher food items are expected to have mandibles with a higher stiffness (i.e., higher resistance to dorsoventral bending). The combined use of maximum and average aspect ratio is still useful in comparing non-amniote tetrapod jaw depth to amniote jaw depth. Non-amniote tetrapods are likely constrained to a relatively shallow jaw depth, resulting in lower maximum and average aspect ratios overall.


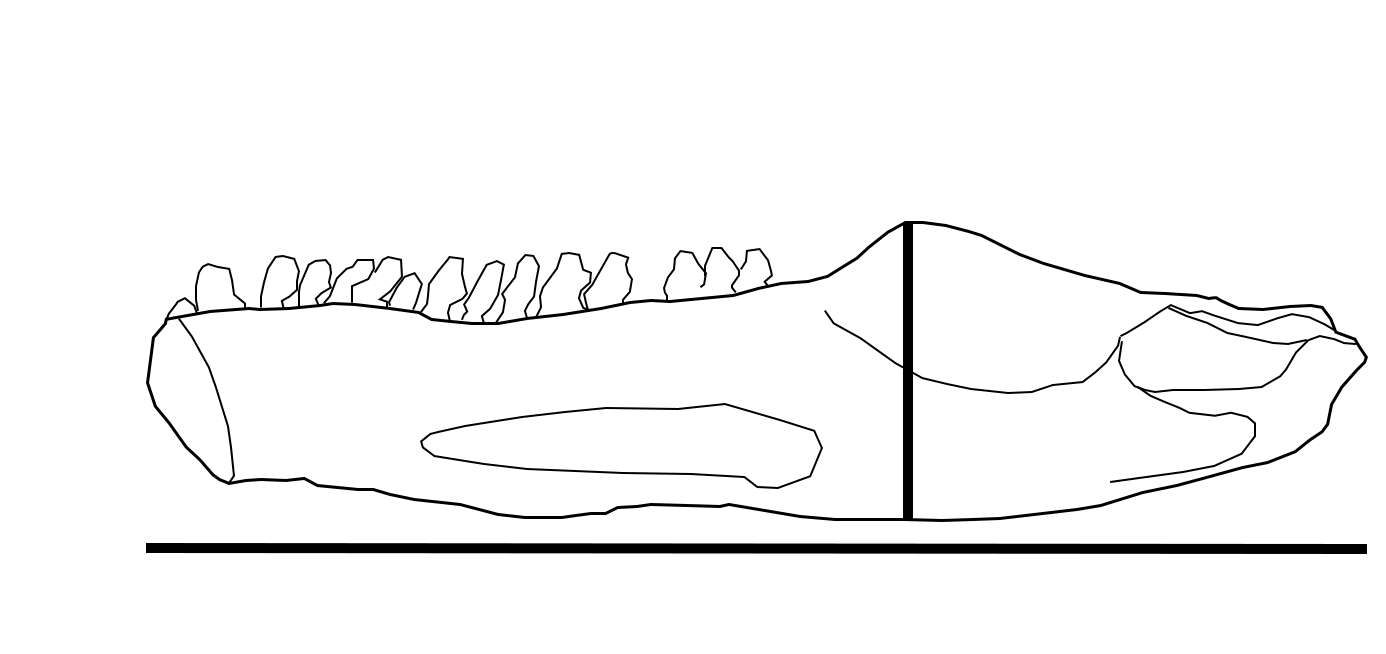


**Measurements required for Maximum Aspect Ratio**. Mandible drawing from *Diadectes absitus* MNG 14473


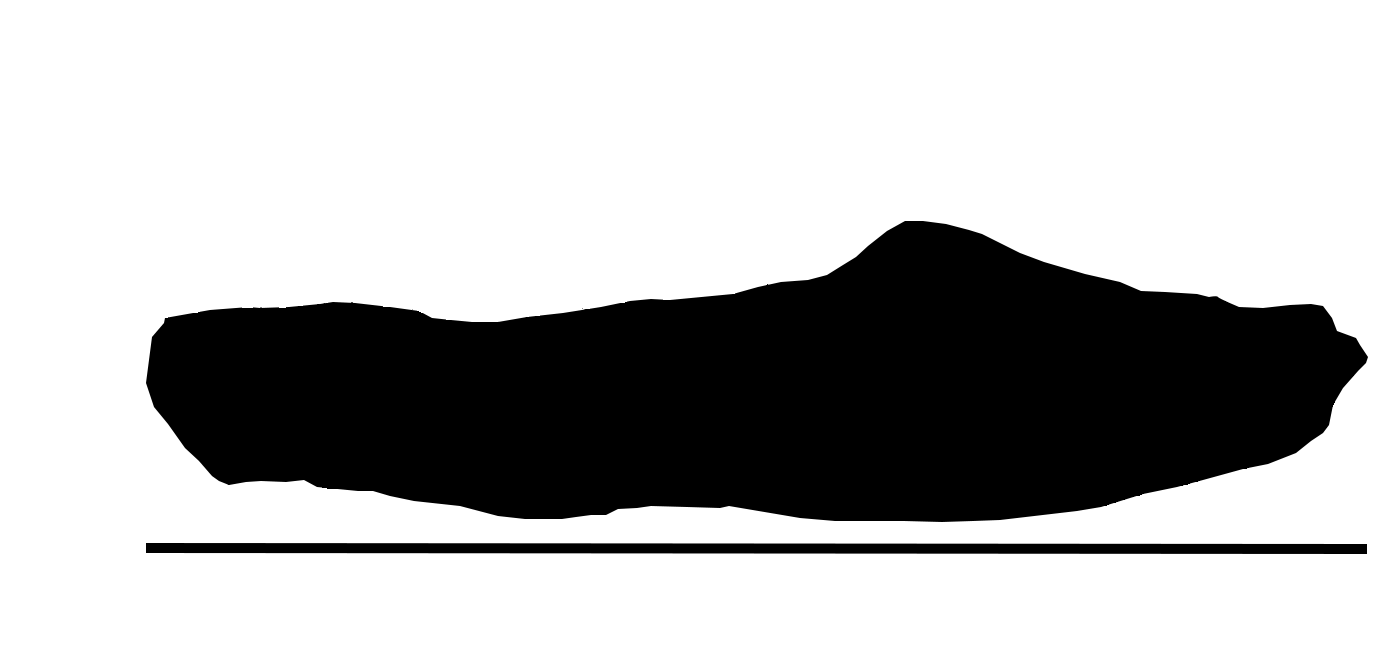


**Measurements required for Average Aspect Ratio**. Mandible drawing from *Diadectes absitus* MNG 14473

**Relative articular offset (character 6).**

The offset between the jaw joint and functional tooth row determines the dental occlusion pattern (Anderson 2009; Anderson et al. 2013; Stubbs et al. 2013; Button et al. 2014; Benevento et al. 2019; Singh et al. 2021, 2024). Articular offset is measured by drawing a line through the functional marginal tooth row or equivalent. Again, fields of minute denticles are excluded in this regard. Then a line is constructed from the articular that intersects the functional tooth row line at a right angle. We treat this distance as the absolute value, i.e. do not discriminate between jaw joints ventral to the functional tooth row line, as *Diadectes lentus* (Welles 1941), or jaw joints dorsal to this line, as in *Archeria crassidisca* (Holmes 1989). This distance is divided by jaw length to obtain the relative articular offset. Taxa whereby the jaw joint is situated on the same line as the functional tooth row, i.e. with an articular offset of 0, occlude the teeth one-by-one, as a pair of scissors. This is more applicable to a carnivorous diet. On the other hand, taxa that process plants or other tough food occlude all their teeth at once, like a vice. This translates to taxa with a high articular offset.


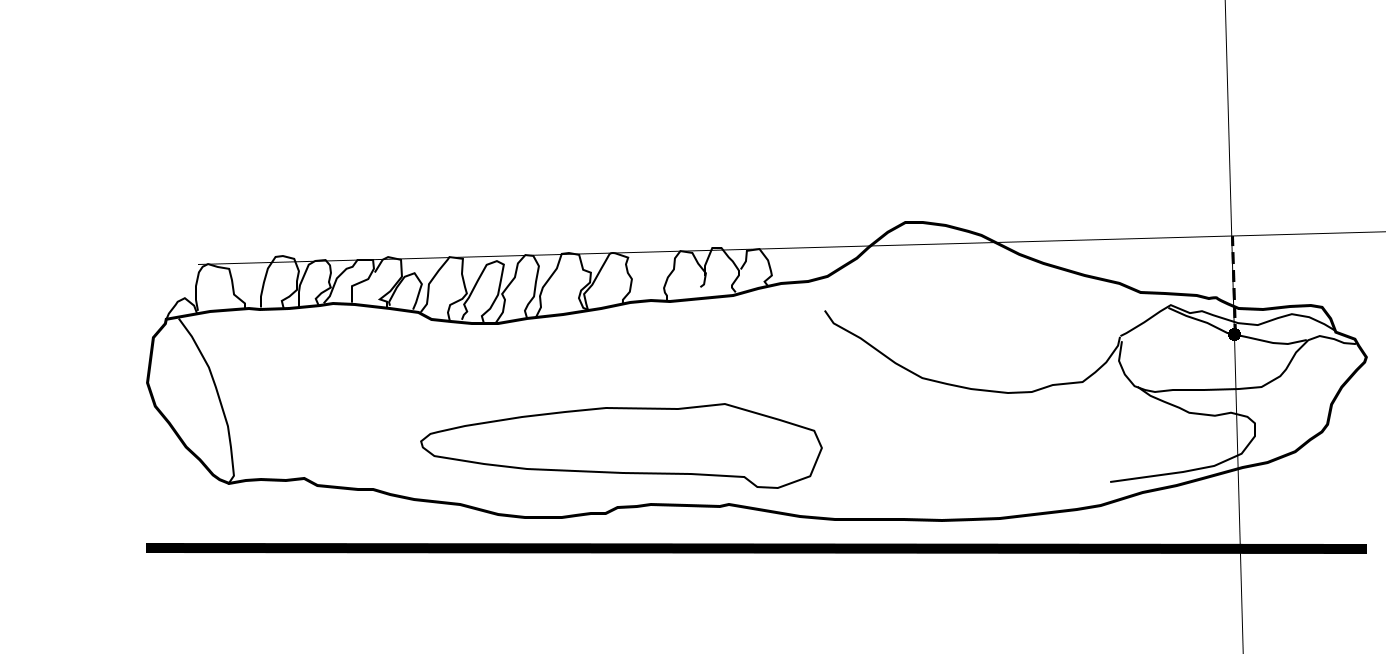


**Measurements required for Articular Offset**. Mandible drawing from *Diadectes absitus* MNG 14473

**Relative ‘dental row’ length (character 7).**

The greater the section of the jaw that is covered by teeth or equivalent, the more the animal can vary in bite force and speed (Anderson et al. 2013; Stubbs et al. 2013; Button et al. 2014; Stubbs and Benton 2016; MacLaren et al. 2017; Benevento et al. 2019; Singh et al. 2021, 2024). For this character, the total length of the functional marginal tooth row or equivalent is measured and divided by jaw length. Edentulous taxa like derived anomodonts bear a cornified sheath – the rhamphotheca (Benoit et al. 2018). In these taxa, the length of the shearing portion of the dentary is measured instead (Singh et al. 2021).


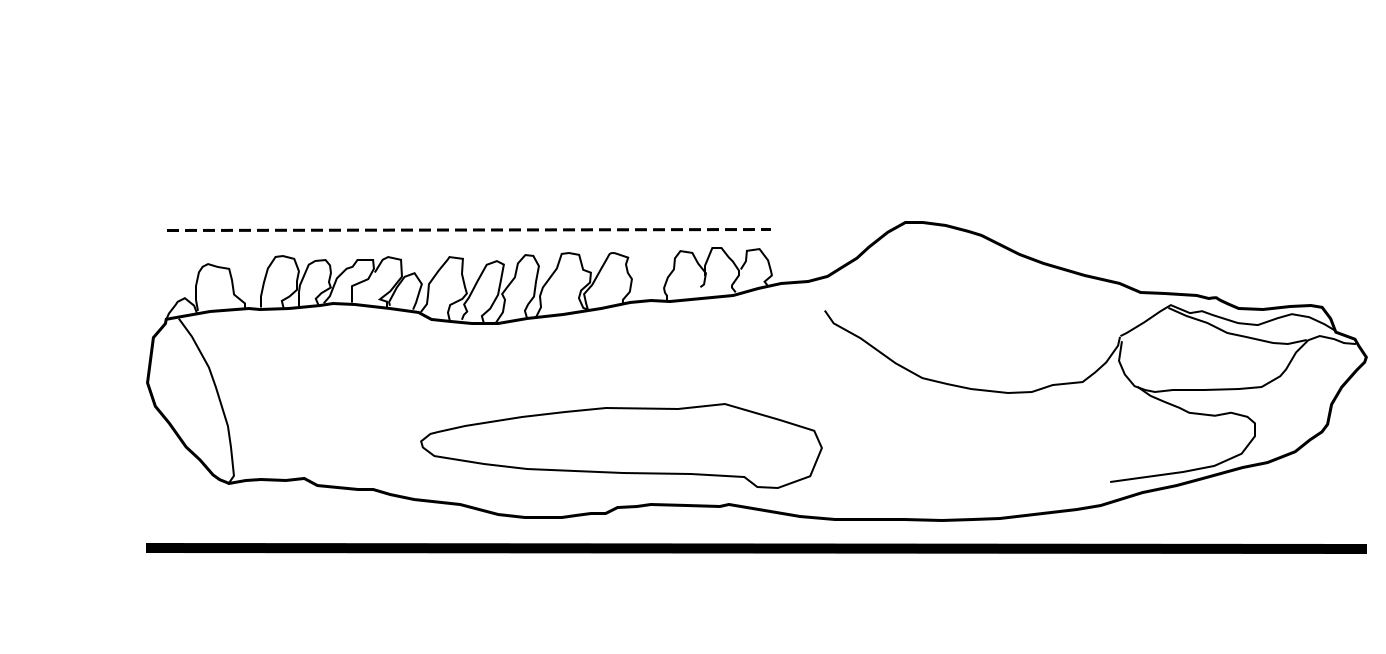


**Measurements required for Dental Row Length**. Mandible drawing from *Diadectes absitus* MNG 14473

**Relative adductor fossa length (character 8).**

Muscle force is proportional to the cross-sectional area of the muscle (Anderson et al. 2011, 2013). The strength of the adductor musculature, then, can be approximated by measuring the length of the adductor fossa (Anderson et al. 2011, 2013; Button et al. 2014; MacLaren et al. 2017). In most tetrapods, the adductor fossa delimits the insertion site of jaw adductor musculature and acts as a recognizable and homologous landmark (e.g., Romer 1956; Barghusen 1973). However, in derived therapsids such as dicynodonts, gorgonopsians, therocephalians and cynodonts, there is no clearly delined adductor fossa (e.g., Barghusen 1968). In dicynodont anomodonts, the medial adductor musculature inserts on the posteriormost section that is enclosed by the surangular and prearticular (King et al. 1989). In gorgonopsians and therocephalians, the insertion site is a small, posterior, dorsal groove on the prearticular (e.g., Kemp 1969; Van den Heever 1987). These regions are measured instead. The length of the adductor fossa or equivalent is then divided by jaw length.


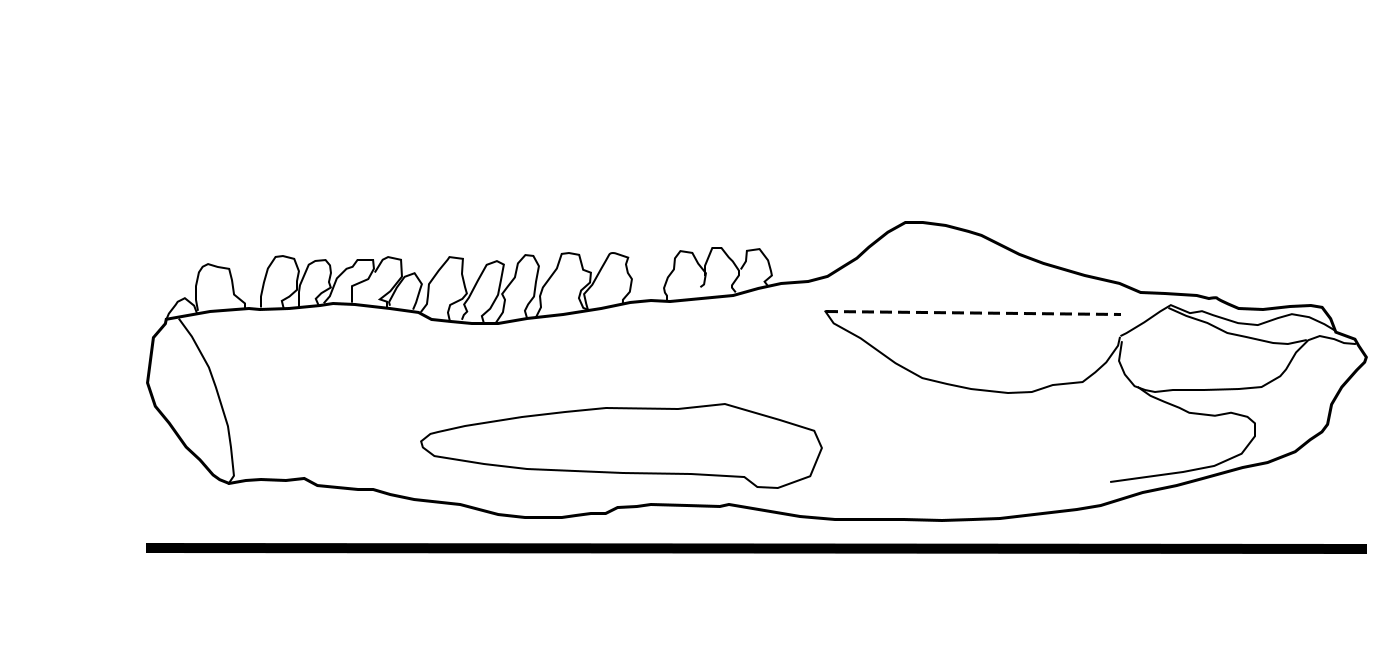


**Measurements required for Adductor Fossa Length**. Mandible drawing from *Diadectes absitus* MNG 14473

**Relative symphysial length (character 9).**

The symphysis is an important biomechanical structure in withstanding shear, bending and torsional stresses (Porro et al. 2011; Walmsley et al. 2013) and is thus related to the robustness of the mandible (Foffa et al. 2024; Singh et al. 2024). Symphysial length is measured as the maximum anteroposterior length along the symphysis (Stubbs et al. 2013; MacLaren et al. 2017) in medial view. This value is then divided by jaw length.


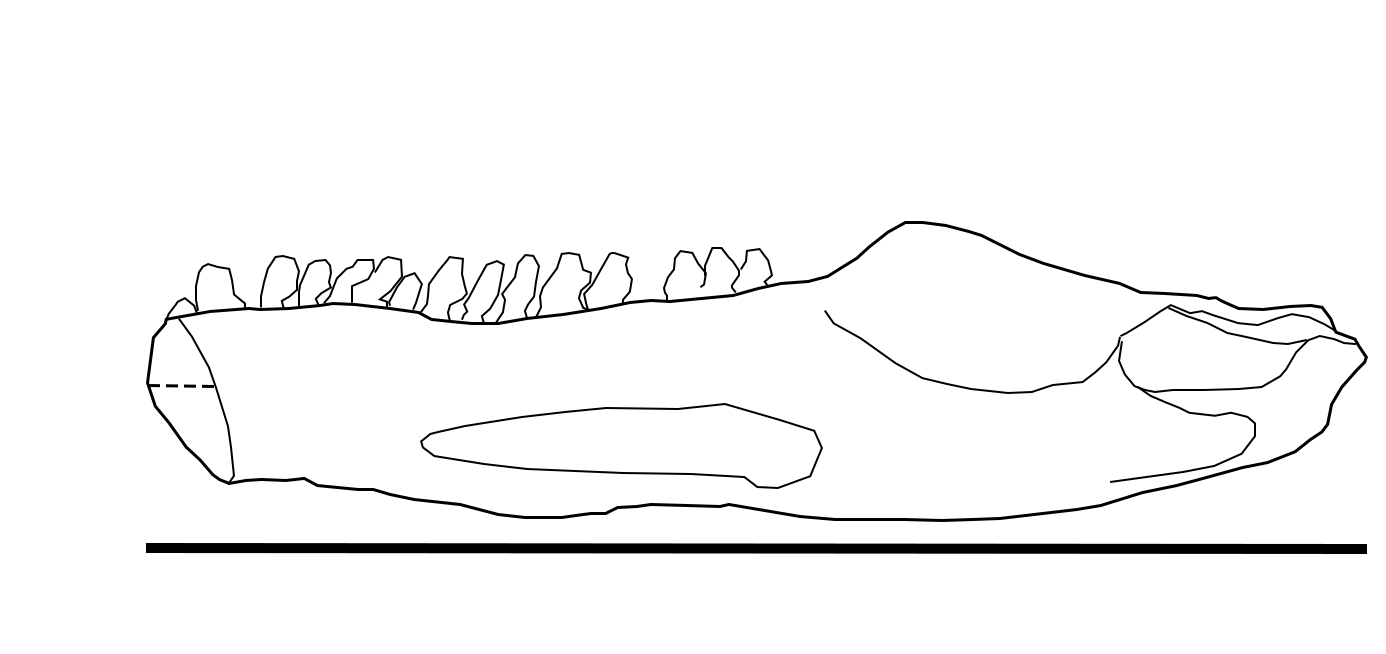


**Measurements required for Symphysial Length**. Mandible drawing from *Diadectes absitus* MNG 14473

Remarks: Measurements 1,2,3,6,7,8 and 9 are usually performed in lateral view, as jaws are most frequently figured in that view. However, some of the required functional landmarks are obscured in lateral view, such as the posterior extent of the tooth row, the adductor fossa and maximum extent of the symphysis. For example, the measurements of Anderson et al. (2013) could not take into account the dental field in *Edaphosaurus boanerges* extending beyond the coronoid process (figured in Modesto 1995), thus underappreciating posterior mechanical advantage in this taxon. MacLaren et al. (2017) also noted this, as the various ornithischian dinosaurs in their dataset wield dental batteries extending medially beyond the coronoid process. The authors argued to take the maximal extent of the tooth row in lateral view for consistency, but ultimately this decision underestimates mechanical advantage and thus functional disparity. Another issue pertains the adductor fossa. In tetrapodomorphs and early tetrapods the adductor fossa faces dorsally, so that Anderson et al. (2011, 2013) could reliably measure its extent in lateral view. In more derived tetrapods, however, the surangular crest is raised dorsally and the adductor fossa faces medially (e.g., Ahlberg and Clack 1998), obscuring its extent from lateral view. Both the relative length of the adductor fossa and the midpoint of this fossa are relevant functional landmarks. Lastly, the precise section of the mandible that sutures with its counterpart at the symphysis is only shown in medial view. These issues are remedied by performing all biomechanical measurements on specimens in medial view.

References

Ahlberg PE and Clack JA. 1998. Lower jaws, lower tetrapods-a review based on the Devonian genus *Acanthostega*. *Transactions of the Royal Society of Edinburgh: Earth Sciences* 89: 11 – 46.

Anderson PSL. 2009. Biomechanics, functional patterns, and disparity in Late Devonian arthrodires. *Paleobiology* 35(3): 321 – 342.

Anderson PSL, Friedman M, Brazeau M and Rayfield EJ. 2011. Initial radiation of jaws demonstrated stability despite faunal and environmental change. *Nature* 476: 206 – 209.

Anderson PSL, Friedman M and Ruta M. 2013. Late to the Table: Diversification of Tetrapod Mandibular Biomechanics Lagged Behind the Evolution of Terrestriality. *Integrative and Comparative Biology* 53(2): 197 – 208.

Barghusen HR. 1968. The lower jaw of cyndonts (Reptilia, Therapsida) and the evolutionary origin of mammal- like adductor jaw musculature. *Postilla* 116: 1 – 49.

Benevento GL, Benson RBJ and Friedman M. 2019. Patterns of mammalian jaw ecomorphological disparity during the Mesozoic/Cenozoic transition. *Proceedings of the Royal Society B* 286: 20190347.

Benoit J, Angielczyk KD, Miyamae JA, Manger P, Fernandez V and Rubidge R. 2018. Evolution of facial innervation in anomodont therapsids (Synapsida): Insights from X-ray computerized microtomography. *Journal of Morphology* 279(5): 673 – 701.

Button DJ, Rayfield EJ and Barrett PM. 2014. Cranial biomechanics underpins high sauropod diversity in resource-poor environments. *Proceedings of the Royal Society B* 281: 20142114.

Cox CB. 1998. The jaw function and adaptive radiation of the dicynodont mammal-like reptiles of the Karoo basin of South Africa. *Zoological Journal of the Linnean Society* 122: 349 – 384.

Crompton AW and Hotton N. 1967. Functional morphology of the masticatory apparatus of two dicynodonts (Reptilia, Therapsida). *Postilla* 109.

Emerson SB. 1985. Skull shape in frogs: correlations with diet. *Herpetologica* 41(2): 177 – 188.

Foffa D, Young MT and Brusatte SL. 2024. Comparative functional morphology indicates niche partitioning among sympatric marine reptiles. *Royal Society Open Science* 11: 231951.

Grossnickle DM. 2020. Feeding ecology has a stronger evolutionary influence on functional morphology than on body mass in mammals. *Evolution* 74(3): 610 – 628.

Holmes R. 1989. The skull and axial skeleton of the lower Permian anthracosauroid amphibian *Archeria crassidisca* Cope. *Palaeontographica Abteilung A*: 161 – 206.

Kammerer CF, Grande L and Westneat MW. 2006. Comparative and Developmental Functional Morphology of the Jaws of Living and Fossil Gars (Actinopterygii: Lepisosteidae). *Journal of Morphology* 267: 1017 – 1031.

Kemp TL. 1969. On the functional morphology of the gorgonopsid skull. *Philosophical Transactions of the Royal Society B* 256(801).

Kemp TL. 2009. Phylogenetic interrelationships and pattern of evolution of the therapsids: testing for polytomy. *Palaeontologia Africana* 44: 1 – 12.

King GM. 1981. The functional anatomy of a Permian dicynodont. *Philosophical Transactions of The Royal Society of London B. Biological Sciences* 291(1050): 243 – 322.

King GM, Oelofsen BW and Rubidge BS. 1989. The evolution of the dicynodont feeding system. *Zoological Journal of the Linnean Society* 96: 185 – 211.

Lautenschlager S, Gill P, Luo Z-X, Fagan MJ and Rayfield EJ. 2017. Morphological evolution of the mammalian jaw adductor complex. *Biological Reviews* 92: 1910 – 1940.

Ma W, Pittman M, Butler RJ and Lautenschlager S. 2022. Macroevolutionary trends in theropod dinosaur feeding mechanics. *Current Biology* 32(3): 677 – 686.

Ma W, Pittman M, Lautenschlager S, Meade LE and Xu X. 2020. Functional Morphology of the Oviraptorosaurian and Scansoriopterygid Skull. *Bulletin of the American Museum of Natural History* 440: 229 – 249.

MacLaren JA, Anderson PSL, Barrett PM and Rayfield EJ. 2017. Herbivorous dinosaur jaw disparity and its relationship to extrinsic evolutionary drivers. *Paleobiology* 43: 15 – 33.

Mallon JC and Anderson JS. 2015. Jaw mechanics and evolutionary paleoecology of the megaherbivorous dinosaurs from the Dinosaur Park Formation (upper Campanian) of Alberta, Canada. *Journal of Vertebrate Paleontology* 35(2): e904323.

Maynard Smith J and Savage RJG. 1959. The mechanics of mammalian jaws. *School Science Review* 40(4): 289 – 301.

Meade LE and Ma W. 2022. Cranial muscle reconstructions quantify adaptation for high bite forces in Oviraptorosauria. *Scientific Reports* 12:3010.

Modesto SP. 1995. The skull of the herbivorous synapsid *Edaphosaurus boanerges* from the lower Permian of Texas. *Palaeontology* 38: 213 – 239.

Morales-García NM, Gill PG, Janis CM and Rayfield EJ. 2021. Jaw shape and mechanical advantage are indicative of diet in Mesozoic mammals. *Nature Communications Biology* 4: 242.

Nabavizadeh A. 2016. Evolutionary Trends in the Jaw Adductor Mechanics of Ornithischian Dinosaurs. *The Anatomical Record* 299: 271 – 294.

Navalón G, Bright JA, Marugán-Lobón J and Rayfield EJ. 2018. The evolutionary relationship among beak shape, mechanical advantage, and feeding ecology in modern birds. *Evolution* 73(3): 422 – 435.

Panchen AL. 1981. A jaw ramus of the Coal Measure amphibian *Anthracosaurus* from Northumberland. *Palaeontology* 24: 85 – 92.

Ponstein J, Hermanson G, Jansen MW, Renaudie J, Fröbisch J and Evers SW. 2024. Functional and character disparity are decoupled in turtle mandibles. *Ecology and Evolution* 14(11): e70557.

Porro LB, Holliday CM, Anapol F, Ontiveros LC, Ontiveros LT and Ross CF. 2011. Free Body Analysis, Beam Mechanics, and Finite Element Modeling of the Mandible of *Alligator mississippiensis*. *Journal of Morphology* 272(8): 910 – 937.

Rayfield EJ, Milner AC, Bui Xuan V and Young PG. 2007. Functional morphology of spinosaur ‘crocodile-mimic’ dinosaurs. *Journal of Vertebrate Paleontology* 27(4): 892 – 901.

Romer AS. 1956. Osteology of the Reptiles. The University of Chicago Press. Chicago & London. 800 pp.

Sakamoto M. 2010. Jaw biomechanics and the evolution of biting performance in theropod dinosaurs. *Proceedings of the Royal Society B* 277: 3327 – 3333.

Singh SA, Elsler A, Stubbs TL, Bond R, Rayfield EJ and Benton MJ. 2021. Niche partitioning shaped herbivore macroevolution through the early Mesozoic. *Nature Communications Biology* 12:2796.

Singh SA, Elsler A, Stubbs TL, Rayfield EJ and Benton MJ. 2024. Predatory synapsid ecomorphology signals growing dynamism of late Palaeozoic terrestrial ecosystems. *Nature Communications* *Biology* 7: 201.

Stayton CT. 2006. Testing hypotheses of convergence with multivariate data: morphological and functional convergence among herbivorous lizards. *Evolution* 60(4): 824 – 841.

Stubbs TL and Benton MJ. 2016. Ecomorphological diversifications of Mesozoic marine reptiles: the roles of ecological opportunity and extinction. *Paleobiology* 42(4): 547 – 573.

Stubbs TL, Pierce SE, Rayfield EJ and Anderson PSL. 2013. Morphological and biomechanical disparity of crocodile-line archosaurs following the end-Triassic extinction. *Proceedings of the Royal Society B*: 280: 20131940.

Tsuji LA. 2013. Anatomy, cranial ontogeny and phylogenetic relationships of the pareiasaur *Deltavjatia rossicus* from the Late Permian of central Russia. *Earth and Environmental Science Transactions of the Royal Society of Edinburgh* 104: 1 – 42.

Van den Brandt MJ, Rubidge BS, Benoit J and Abdala F. 2021. Cranial morphology of the middle Permian pareiasaur *Nochelesaurus alexanderi* from the Karoo Basin of South Africa. *Earth and Environmental Science Transactions of the Royal Society of Edinburgh* 112: 29 – 49.

Van den Heever JA. 1987. The comparative and functional cranial morphology of the early Therocephalia (Amniota: Therapsida). PhD dissertation, University of Stellenbosch, Stellenbosch, South Africa.

Wainwright SA, Biggs WD, Currey JD and Gosline JM. 1976. Mechanical design in organisms. Princeton, NJ: Princeton University Press.

Walmsley CW, Smits PD, Quayle MR, McCurry MR, Richards HS, Oldfield CC, Wroe S, Clausen PD and McHenry CR. 2013. Why the Long Face? The Mechanics of Mandibular Symphysis Proportions in Crocodiles. *PLoS ONE* 8(1): e53873

Welles SP. 1941. The mandible of a diadectid cotylosaur. *University of California Publications in Geological Sciences*: 423 – 431.

Westneat MW. 1994. Transmission of force and velocity in the feeding mechanism of labrid fishes (Teleostei: Perciformes). *Zoomorphology* 114: 103 – 118.

Westneat MW. 2003. A biomechanical model for analysis of muscle force, power output and lower jaw motion in fishes. *Journal of Theoretical Biology* 223: 269 – 281.
