# Supplementary material for: Mandibular form and function is more disparate in amniotes than in non-amniote tetrapods from the late Palaeozoic
Source: PeerJ. 2025 Nov 26;13:e20243. doi: 10.7717/peerj.20243 (PMC12664332; doi:10.7717/peerj.20243)
Supplement: Supplemental Information 5 [file peerj-13-20243-s005.docx]

SUPPLEMENTARY FILE 6 Journal references to specimens

Adams GR, Mann A and Maddin HC. 2019. New embolomerous tetrapod material and a faunal overview of the Mississippian-aged Point Edward locality, Nova Scotia, Canada. *Canadian Journal of Earth Sciences* 57(3): 407 – 417.

Albright GM, Sumida SS and Jung JP. 2021. A new genus of Captorhinid reptile (Amniota: Eureptilia) from the lower Permian Hennesey Formation of central Oklahoma, and a consideration of homoplasy in the family Captorhinidae. *Annals of Carnegie Museum* 87(2): 89 – 116.

Anderson JS. 2003. Cranial anatomy of *Coloraderpeton brilli*, postcranial anatomy of *Oestocephalus amphiuminus*, and reconsideration of Ophiderpetontidae (Tetrapoda: Lepospondyli: Aistopoda). *Journal of Vertebrate Paleontology* 23(3): 532 – 543.

Andrews SM and Carroll RL. 1991. The Order Adelospondyli: Carboniferous lepospondyl amphibians. *Transactions of the Royal Society of Edinburgh: Earth Sciences* 82: 239 – 275.

Angielczyk KD, Benoit J and Rubidge BS. 2019. A new tusked cistecephalid dicynodont (Therapsida, Anomodontia) from the upper Permian upper Madumabisa Mudstone Formation, Luangwa Basin, Zambia. *Papers in Palaeontology* 7: 405 – 446.

Angielczyk KD, Rubidge BS, Day MO and Lin F. 2016. A reevaluation of *Brachyprosopus broomi* and *Chelydontops altidentalis*, dicynodonts (Therapsida, Anomodontia) from the middle Permian Tapinocephalus Assemblage Zone of the Karoo Basin, South Africa. *Journal of Vertebrate Paleontology* 36(2): e1078342.

Arbez T, Atkins JB and Maddin HC. 2022. Cranial anatomy and systematics of *Dendrerpeton* cf. *helogenes* (Tetrapoda, Temnospondyli) from the Pennsylvanian of Joggins, revisited through micro-CT scanning. *Papers in Paleontology*: e1421.

Azevedo KL, Vega CS and Soaras BS. 2017. A new specimen of *Australerpeton cosgriffi* Barbarena, 1998 (Stereospondyli: Rhinesuchidae) from the middle/ upper Permian Rio do Rasto Formation, Paraná Basin, Brazil. *Revista Brasileira de Paleontologia* 20(3): 333 – 344.

Barták P and Ivanov M. 2022. The exceptionally well-preserved *Sauropleura scalaris* (Nectridea: Urocordylidae) from the late Carboniferous of the Czech Republic: new information on ontogeny, lateral line and tail. *Zoological Journal of the Linnean Society* 199: 392 – 416.

Beaumont EH. 1977. Cranial morphology of the Loxommatidae (Amphibia: Labyrinthodontia). *Philosophical Transactions of The Royal Society of London B. Biological Sciences* 280(971): 29 – 101.

Beerbower JR. 1963. Morphology, paleoecology, and phylogeny of the Permo-Pennysylvanian amphibian *Diploceraspis*. *Bulletin of the Museum of Comparative Zoology at Harvard College* 130(2): 31 – 108.

Bendel E-M, Kammerer CF, Kardjilov N, Fernandez V and Fröbisch J. 2018. Cranial anatomy of the gorgonopsian *Cynariops robustus* based on CT-reconstruction. *PLoS ONE* 13(11): e0207367.

Benton MJ. 2016. The Chinese pareiasaurs. *Zoological Journal of the Linnean Society* 177: 813 – 853.

Berman DS, Reisz RR, Bolt JR and Scott D. 1995. The cranial anatomy and relationships of the synapsid *Varanosaurus* (Eupelycosauria: Ophiacodontidae) from the early Permian of Texas and Oklahoma. *Annals of Carnegie Museum* 64(2): 99 – 133.

Berman DS, Reisz RR and Scott D. 2010. Redescription of the skull of *Limnoscelis paludis* Williston (Diadectomorpha: Limnoscelidae) from the Upper Pennsylvanian of El Cobre Canyon, northern New Mexico. *Bulletin of New Mexico Museum of Natural History and Science* 49: 185 – 210.

Bolt JR and Lombard RE. 2001. The mandible of the primitive tetrapod *Greererpeton*, and the early evolution of the tetrapod lower jaw. *Journal of Paleontology* 75(5): 1016 – 1042.

Bolt JR and Lombard RE. 2006. *Sigournea multidentata*, a new stem tetrapod from the Upper Mississippian of Iowa, USA. *Journal of Paleontology* 80(4): 717 – 725.

Bolt JR and Wassersug RJ. 1975. Functional morphology of the skull in *Lysorophus*: a snake-like Paleozoic amphibian (Lepospondyli). *Paleobiology* 1: 320 – 332.

Boy JA. 1990. On some representatives of the Eryopoidea (Amphibia: Temnospondyli) from the European Rotliegend (? uppermost Carboniferous - Permian) 3. *Onchiodon*. *Paläontologisches Zeitschrift* 64(3/4): 287 – 312.

Boy JA. 1995. On the Micromelerpetontidae (Amphibia: Temnospondyli). 1. Morphology and palaeoecology of *Micromelerpeton credneri* (Lower Permian; southwestern Germany). *Paläontologisches Zeitschrift* 69(3/4): 429 – 547.

Broom R. 1914. A further comparison of the South African dinocephalians with the American pelycosaurs. *Bulletin of the American Museum of Natural History* 33(9): 135 – 141.

Bulanov VV and Sennikov AG. 2015. Substantiation of Validity of the Late Permian Genus *Weigeltisaurus* Kuhn, 1939 (Reptilia, Weigeltisauridae). *Paleontological Journal* 49(10): 1101 – 1111.

Castanhinha R, Araújo R, Júnior LC, Angielczyk KD, Martins GG, Martins RMS, Chaouiya C, Beckmann F and Wilde F. 2014. [Bringing Dicynodonts Back to Life: Paleobiology and Anatomy of a New Emydopoid Genus from the Upper Permian of Mozambique.](https://www.ncbi.nlm.nih.gov/pmc/articles/PMC3852158) PLOS ONE 8 (12): e80974.

Carroll RL. 1981. Plesiosaur ancestors from the upper Permian of Madagascar. *Philosophical Transactions of the Royal Society of London B* 293(1066): 315 – 383.

Carroll RL. 1998. Cranial anatomy of ophiderpetontid aistopods: Palaeozoic limbless amphibians. *Zoological Journal of the Linnean Society* 122: 143 – 166.

Carroll RL, Kuhn O and Tatarinov LP. 1972. Encyclopdia of Paleoherpetology. Part 5 Batrachosauria (Anthracosauria) B: Gephyrostegida-Chroniosuchida. Gustav Fischer Verlag Stuttgart, Portland USA.

Chase JN. 1965. *Neldasaurus wrightae*, a new rhachitomous labyrinthodont from the Texas Lower Permian. *Bulletin of the Museum of Comparative Zoology* 133(3): 153 – 225.

Cisneros JC, Marsicano C, Angielczyk KD, Smith RHM, Richter M, Fröbisch J, Kammerer CF and Sadleir RW. 2015. New Permian fauna from tropical Gondwana. *Nature Communications* 6: 8676.

Clack JA. 1987. *Pholiderpeton scutigerum* Huxley, an amphibian from the Yorkshire Coal Measures. *Philosophical Transactions of The Royal Society of London B. Biological Sciences* 318: 1 – 107.

Clack JA and Milner AR. 2009. Morphology and systematics of the Pennsylvanian amphibian *Platyrhinops lyelli* (Amphibia: Temnospondyli). *Earth and Environmental Science Transactions of the Royal Society of Edinburgh* 100: 275 – 295.

Clack JA, Ruta M, Milner AR, Marshall JEA, Smithson TR and Smithson KZ. 2019. *Acherontiscus caledoniae*: the earliest heterodont and durophagous tetrapod. *Royal Society Open Science* 6: 182087.

Clark J and Carroll RL. 1973. Romeriid reptiles from the lower Permian. *Bulletin of the Museum of Comparative Zoology at Harvard College* 144: 353 – 407.

Cox CB and Angielczyk KD. 2015. A new endothiodont dicynodont (Therapsida, Anomodontia) from the Permian Ruhuhu Formation (Songea Group) of Tanzania and its feeding system. *Journal of Vertebrate Paleontology* 35(4): e935388.

Day MO, Smith RMH, Benoit J, Fernandez V and Rubidge BS. 2018. A new species burnetiid (Therapsida, Burnetiamorpha) from the early Wuchiapingian of South Africa and implications for the evolutionary ecology of the family Burnetiidae. *Papers in Paleontology* 4(3): 453 – 475.

De Órfão Correia JI. 2019. On Captorhinids: Analysis of morphological characters with particular attention to skull sculpturing. Master thesis. Universidade de Aveiro, Brazil. 220 pp.

Dias EV, Dias-Da Silva S and Schultz CL. 2020. A new short-snouted rhinesuchid from the Permian of southern Brazil. *Revista Brasileira de Paleontologia* 23(2): 98 – 122.

Dilkes DW. 1990. A new trematopsid amphibian (Temnospondyli: Dissorophoidea) from the Lower Permian of Texas. *Journal of Vertebrate Paleontology* 10(2): 222 – 243.

Dodick JT and Modesto SP. 1995. The cranial anatomy of the captorhinid reptile *Labidosaurikos meachami* from the lower Permian of Oklahoma. *Palaeontology* 38(3): 687 – 711.

Douthitt H. 1917. The structure and relationships of *Diplocaulus*. PhD thesis, University of Chicago, 41 pp.

Englehorn J, Small BJ and Huttenlocker A. 2008. A Redescription Of *Acroplous vorax* (Temnospondyli: Dvinosauria) Based On New Specimens From the Early Permian Of Nebraska and Kansas, U.S.A. *Journal of Vertebrate Paleontology* 28(2): 291 – 305.

Ewer RF. 1961. The anatomy of the anomodont *Daptocephalus leoniceps* (Owen). *Proceedings of the Zoological Society of London* 136(3): 375 – 402.

Gebauer EVI. 2012. Re-assessment of the Taxonomic Position of the Specimen GPIT/RE/7113 (*Sauroctonus parringtoni* comb. nov., Gorgonopsia). *In*: CF Kammerer, KD Angielczyk, J Fröbisch (eds.). Early Evolutionary History of the Synapsida. Springer Dordrecht. pp 185 – 209.

Gee BM, Bevitt JJ and Reisz RR. 2019b. A Juvenile Specimen of the Trematopid *Acheloma* From Richards Spur, Oklahoma and Challenges of Trematopid Ontogeny. *Frontiers in Earth Science* 7:38.

Gee BM, Bevitt JJ, Garbe U and Reisz RR. 2019a. New material of the ‘microsaur’ *Llistrofus* from the cave deposits of Richards Spur, Oklahoma and the paleoecology of the Hapsidopareiidae. *PeerJ* 7:e6327.

Gee BM, Bevitt JJ and Reisz RR. 2021. Computed tomographic analysis of the cranium of the early Permian recumbirostran ‘microsaur’ *Euryodus dalyae* reveals new details of the braincase and mandible. *Papers in Palaeontology* 7(2): 721 – 749.

Glienke S. 2015. Two new species of the genus *Batropetes* (Tetrapoda, Lepospondyli) from the Central European Rotliegend (basal Permian) in Germany. *Journal of Vertebrate Paleontology* e918041.

Godfrey SJ and Holmes RB. 1989. A tetrapod lower jaw from the Pennsylvanian (Westphalian A) of Nova Scotia. *Canadian Journal of Earth Sciences* 26: 1036 – 1040.

Heaton MJ. 1979. Cranial anatomy of primitive captorhinid reptiles from the late Pennsylvanian and early Permian Oklahoma and Texas. *Bulletin of the University of Oklahoma* 127: 1 – 84.

Holmes R. 1984. The Carboniferous amphibian *Proterogyrinus scheelei* Romer, and the early evolution of tetrapods. *Philosophical Transactions of The Royal Society of London B. Biological Sciences* 306: 431 – 524.

Holmes R. 1989. The skull and axial skeleton of the lower Permian anthracosauroid amphibian *Archeria crassidisca* Cope. *Palaeontographica Abteilung A*: 161 – 206.

Holmes R, Berman DS and Anderson JS. 2013. A new dissorophid (Temnospondyli, Dissorophoidea) from the Early Permian of New Mexico (United States). *Comptes Rendus Palevol* 12: 419 – 435.

Hook RW. 1983. *Colosteus scutellatus* (Newberry), a Primitive Temnospondyl Amphibian from the Middle Pennsylvanian of Linton, Ohio. *American Museum Novitates* 2770: 1 – 41.

Hunt AK, Ford DP, Fernandez V, Choiniere JN and Benson RBJ. 2023. A description of the palate and mandible of *Youngina capensis* (Sauropsida, Diapsida) based on synchrotron tomography, and the phylogenetic implications. *Papers in Palaeontology* e1521.

Huttenlocker AK, Pardo JD, Small BJ and Anderson JS. 2013. Cranial morphology of recumbirostrans (Lepospondyli) from the Permian of Kansas and Nebraska, and early morphological evolution inferred by micro-computed tomography. *Journal of Vertebrate Paleontology* 33(3): 540 – 552.

Ivakhnenko MF. 2008. Cranial Morphology and Evolution of Permian Dinomorpha (Eotherapsida) of Eastern Europe. *Paleontological Journal* 42(9): 859 – 995.

Ivakhnenko MF. 2011. Permian and Triassic Therocephals (Eutherapsida) of Eastern Europe. *Paleontological Journal* 45(9): 981 – 1144.

Ivakhnenko MF. 2013. Cranial Morphology of *Dvinia prima* Amalitzky (Cynodontia, Theromorpha). *Paleontological Journal* 47(2): 210 – 222.

Jenkins KM, Foster W, Napoli JG, Meyer DL, Bever GS and Bhullar B-AS. 2024. Cranial anatomy and phylogenetic affinities of *Bolosaurus major*, with new information on the unique bolosaurid feeding apparatus and evolution of the impedance-matching ear. *The Anatomical Record*.

Kemp TS. 1969. On the functional morphology of the gorgonopsid skull. *Philosophical Transactions of the Royal Society of London B* 256(801): 1 – 83.

Kemp TS. 1979. The Primitive Cynodont *Procynosuchus*: Functional Anatomy of the Skull and Relationships. *Philosophical Transactions of the Royal Society of London* *B* 285(1005): 73 – 122.

Kemp TS. 2005. The origin and evolution of mammals. Oxford University Press. 325 pp.

King GM. 1981. The functional anatomy of a Permian dicynodont. *Proceedings and philosophical Transactions of the Royal Society* 291: 243 – 322.

King GM. 1988. Anomodontia. *Handbuch der Paläoherpetologie*, *Teil 17C*: 1 – 174.

King GM. 1994. The early anomodont *Venjukovia* and the evolution of the anomodont skull. *Journal of Zoology* 232(4): 651 – 673.

Kissel RA. 2010. Morphology, Phylogeny and Evolution of Diadectidae (Cotylosauria: Diadectomorpha). PhD thesis, University of Toronto, 185 pp.

Klembara J. 1997. The cranial anatomy of *Discosauriscus* Kuhn, a seymouriamorph tetrapod from the Lower Permian of the Boskovice Furrow (Czech Republic). *Philosophical Transactions of the Royal Society of London B* 352: 257 – 302.

Klembara J. 2005. A new discosauriscid seymouriamorph tetrapod from the Lower Permian of Moravia, Czech Republic. *Acta Palaeontologica Polonica* 50: 25 – 48.

Klembara J. 2009. The skeletal anatomy and relationships of a new discosauriscid seymouriamorph from the lower Permian of Moravia (Czech Republic). *Annals of Carnegie Museum* 77(4): 451 – 483.

Klembara J. 2011. The cranial anatomy, ontogeny, and relationships of *Karpinskiosaurus secundus* (Amalitzky) (Seymouriamorpha, Karpinskiosauridae) from the Upper Permian of European Russia. *Zoological Journal of the Linnean Society* 161: 184 – 212.

Klembara J, Clack JA, Milner AR and Ruta M. 2014. Cranial anatomy, ontogeny, and relationships of the Late Carboniferous tetrapod *Gephyrostegus bohemicus* Jaekel, 1902. *Journal of Vertebrate Paleontology* 34(4): 774 – 792.

Klembara J and Ruta M. 2003. The seymouriamorph tetrapod *Utegenia shpinari* from the ?Upper Carboniferous–Lower Permian of Kazakhstan. Part I: Cranial anatomy and ontogeny. *Transactions of the Royal Society of Edinburgh: Earth Sciences* 94: 45 – 74.

Klembara J and Ruta M. 2005. The seymouriamorph tetrapod *Ariekanerpeton sigalovi* from the Lower Permian of Tadzhikistan. Part I: Cranial anatomy and ontogeny. *Transactions of the Royal Society of Edinburgh: Earth Sciences* 96: 43 – 70.

Klembara J, Ruta M, Anderson J, Mayer T, Hain M and Valaška D. 2024. A redescription of *Brouffia orientalis* Carroll & Baird, 1972 from the Upper Carboniferous of the Czech Republic and the status and affinities of protorothyridid amniotes. *Swiss Journal of Palaeontology* 143: 33.

Kuhn O. 1969. Encyclopedia of Palaeoherpetology. Part 6: Cotylosauria. Gustav Fischer Verlag, Stuttgart, Portland-USA. 98 pp.

Langston W and Reisz RR. 1981. *Aerosaurus wellesi*, new species, a varanopseid mammal-like reptile (Synapsida: Pelycosauria) from the lower Permian of New Mexico. *Journal of Vertebrate Palaeontology* 1: 73 – 96.

Laurin M. 1993. Anatomy and relationships of *Haptodus garnettensis*, a Pennsylvanian synapsid from Kansas. *Journal of Vertebrate Paleontology* 13(2): 200 – 229.

Laurin M. 1996. A redescription of the cranial anatomy of *Seymouria baylorensis*, the best known eymouriamorph (Vertebrata: Seymouriamorpha). *PaleoBios* 17: 1 – 16.

Laurin M and Soler-Gijón R. 2006. The oldest known Stegocephalian (Sarcopterygii: Temnospondyli) from Spain. *Journal of Vertebrate Paleontology* 26(2): 284 – 299.

Lee MSY. 1997. Pareiasaur phylogeny and the origin of turtles. *Zoological Journal of the Linnean Society* 120: 197 – 280.

Levy F. 2023. A Description of the Cranial Anatomy of a Specimen of the Gorgonopsian *Lycaenops* through CT Reconstruction. BSc thesis. Wesleyan University. 100 pp.

Liu J. 2023. The tetrapod fauna of the upper Permian Naobaogou Formation of China: 9. A new species of *Gansurhinus* (Reptilia: Captorhinidae) and a revision of Chinese captorhinids. *Journal of Vertebrate Paleontology* e2203200.

Liu J and Bever GS. 2015. The last diadectomorph sheds light on Late Palaeozoic tetrapod biogeography. *Biology Letters* 11: 20150100.

Liu J and Chen J. 2020. The tetrapod fauna of the upper Permian Naobaogou Formation of China: 7. *Laosuchus hun* sp. nov. (Chroniosuchia) and interrelationships of chroniosuchians. *Journal of Systematic Palaeontology* 18(24): 2043 – 2058.

Liu J, Rubidge B and Li J. 2010. A new specimen of *Biseridens qilianicus* indicates its phylogenetic position as the most basal anomodont. *Proceedings of the Royal Society B* 277: 285 – 292.

Lombard RE and Bolt JR. 2006. The mandible of *Whatcheeria deltae*, an early tetrapod from the Late Mississipian of Iowa. In: Carrano MT et al. (eds.). Amniote Paleobiology: Perspectives on the Evolution of Mammals, Birds and Reptiles. University of Chicago Press, pp. 21 – 52.

Lucas SG, Rinehart LF and Celeskey MD. 2018. The oldest specialized tetrapod herbivore: A new eupelycosaur from the Permian of New Mexico, USA. *Palaeontologia Electronica* 21.3.39A: 1 – 42.

MacDougall MJ, Scott D, Modesto SP, Williams SA and Reisz RR. 2017. New material of the reptile *Colobomycter pholeter* (Parareptilia: Lanthanosuchoidea) and the diversity of reptiles during the Early Permian (Cisuralian). *Zoological Journal of the Linnean Society* 180: 661 – 671.

MacDougall MJ, Winge A, Ponstein J, Jansen MA, Reisz RR and Fröbisch J. 2019. New information on the early Permian lanthanosuchoid *Feeserpeton oklahomensis* based on computed tomography. *PeerJ* 7: e7753.

Maddin HC, Olori JC and Anderson JS. 2011. A Redescription of *Carrolla craddocki* (Lepospondyli: Brachystelechidae) Based on High-Resolution CT, and the Impacts of Miniaturization and Fossoriality on Morphology. *Journal of Morphology* 272: 722 – 742.

Maddin HC, Sidor CA and Reisz RR. 2008. Cranial anatomy of *Ennatosaurus tecton* (Synapsida: Caseidae) from the Middle Permian of Russia and the evolutionary relationships of Caseidae. *Journal of Vertebrate Paleontology* 28: 160 – 180.

Maisch MW. 2003. Lower jaw morphology and jaw adductor musculature of the giant Permian dicynodont *Rhachiocephalus* Seeley 1898 (Therapsida) from the late Permian of Tanzania. *Geologica et Paleontologica* 37: 89 – 106.

Mann A, MacDaniel EJ, McColville ER and Maddin HC. 2019. *Carbonodraco lundi* gen et sp. nov., the oldest parareptile, from Linton, Ohio, and new insights into the early radiation of reptiles. *Royal Society Open Science* 6: 191191.

Mann A, Pardo JD and Maddin HC. 2022. Snake-like limb loss in a Carboniferous amniote. *Nature Ecology & Evolution* 6(5): 1 – 8.

Marsicano CA, Latimer E, Rubidge B and Smith RHM. 2017. The Rhinesuchidae and early history of the Stereospondyli (Amphibia: Temnospondyli) at the end of the Palaeozoic. *Zoological Journal of the Linnean Society* 181: 357 – 384.

Matamales-Andreu R, Mujal E, Galobart A and Fortuny J. 2023. A new medium-sized moradisaurine captorhinid eureptile from the Permian of Mallorca (Balearic Islands, western Mediterranean) and correlation with the co-occurring ichnogenus *Hyloidichnus*. *Papers in Palaeontology* e1498.

Milner AR and Schoch RR. 2013. *Trimerorhachis* (Amphibia: Temnospondyli) from the Lower Permian of Texas and New Mexico: cranial osteology, taxonomy and biostratigraphy. *Neues Jahrbuch für Geologie und Paläontologie – Abhandlungen* 270: 91 – 128.

Milner AR and Sequeria SEK. 1994. The temnospondyl amphibians from the Visean of East Kirkton, West Lothian, Scotland. *Transactions of the Royal Society of Edinburgh: Earth Sciences* 84: 331 – 361.

Milner AR and Sequeira SEK. 1998. A cochleosaurid temnospondyl amphibian from the Middle Pennsylvanian of Linton, Ohio, U.S.A. *Zoological Journal of the Linnean Society* 122: 261 – 290.

Modesto SP. 1995. The skull of the herbivorous synapsid *Edaphosaurus boanerges* from the Lower Permian of Texas. *Palaeontology* 38: 213 – 239.

Modesto SP, Richards CD, Ide O and Sidor CA. 2019. The vertebrate fauna of the upper Permian of Niger – X. The mandible of the captorhinid reptile *Moradisaurus grandis*. *Journal of Vertebrate Paleontology*: e1531877.

Modesto SP, Rubidge B and Welmann J. 1999. The most basal anomodont therapsid and the primacy of Gondwana in the evolution of the anomodonts. *Proceedings of the Royal Society of London B* 266: 331 – 337.

Modesto SP and Rybczynski N. 2001. The amniote faunas of the Russian Permian: implications for Late Permian terrestrial vertebrate biogeography. *In*: MJ Benton, MA Shishkin, DM Unwin and EM Kurochkin (eds.). The age of dinosaurs in Russia and Mongolia. Cambridge University Press, Cambridge: pp 13 – 34.

Modesto SP, Scott DM, Berman DS, Müller J and Reisz RR. 2007. The skull and the palaeoecological significance of *Labidosaurus hamatus*, a captorhinid reptile from the Lower Permian of Texas. *Zoological Journal of the Linnean Society* 149: 237 – 262.

Moss JM. 1972. The morphology and phylogenetic relationships of the Lower Permian tetrapod *Tseajaia campi* Vaughn (Amphibia: Seymouriamorpha). *University of California Publications in Geological Sciences* 98: 1 – 59.

Novikov IV, Shishkin MA and Golubev K. 2001. Permian and Triassic anthracosaurs from Eastern Europe. *In*: MJ Benton, MA Shishkin, DM Unwin and EM Kurochkin (eds.). The age of dinosaurs in Russia and Mongolia. Cambridge University Press, Cambridge. pp 60 – 70.

Olroyd SL and Sidor CA. 2022. Nomenclature, comparative anatomy, and evolution of the reflected lamina of the angular in non-mammalian synapsids. *Journal of Vertebrate Paleontology* e2101923.

Olson EC. 1984. The Taxonomic Status and Morphology of *Pleuristion brachycoelus* Case; Referred to *Protocaptorhinus pricei* Clark and Carroll (Reptilia: Captorhinomorpha). *Journal of Paleontology* 58(5): 1282 – 1295.

Olson EC and Barghusen H. 1962. Permian vertebrates from Oklahoma and Texas. Vol 2. University of Oklahoma. 68 pp.

Panchen AL. 1972. The skull and skeleton of *Eogyrinus attheyi* Watson (Amphibia: Labyrinthodontia). *Philosophical Transactions of the Royal Society of London B. Biological Sciences* 263: 279 – 326.

Panchen AL. 1977. On *Anthracosaurus russelli* Huxley (Amphibia: Labyrinthodontia) and the family Anthracosauridae. *Philosophical Transactions of The Royal Society of London B. Biological Sciences* 279: 447 – 512.

Panchen AL. 1981. A jaw ramus of the Coal Measure amphibian *Anthracosaurus* from Northumberland. *Palaeontology* 24: 85 – 92.

Pardo JD, Szostakiwskyj M, Ahlberg PE and Anderson JS. 2017. Hidden morphological diversity among early tetrapods. *Nature* 546(7660): 642 – 645.

Piñeiro G, Ferigolo J, Ramos A and Laurin M. 2012. Cranial morphology of the Early Permian mesosaurid *Mesosaurus tenuidens* and the evolution of the lower temporal fenestration reassessed. *Comptes Rendus Palevol* 11: 379 – 391.

Ponstein J, MacDougall MJ and Fröbisch J. 2024. A comprehensive phylogeny and revised taxonomy of Diadectomorpha with a discussion on the origin of tetrapod herbivory. *Royal Society Open Science* 11: 231566.

Porro LB, Martin-Silverstone E and Rayfield EJ. 2024. Descriptive anatomy and three-dimensional reconstruction of the skull of the tetrapod *Eoherpeton watsoni* Panchen, 1975 from the Carboniferous of Scotland. *Earth and Environmental Science Transactions of the Royal Society of Edinburgh* 1 – 21.

Porro LB, Rayfield EJ and Clack JA. 2023. Computed tomography and three-dimensional reconstruction of the skull of the stem tetrapod *Crassigyrinus scoticus*. *Journal of Vertebrate Paleontology*: e2183134.

Pusch LC, Ponstein J, Kammerer CF and Fröbisch J. 2020. Novel endocranial data on the early therocephalian *Lycosuchus vanderrieti* underpin high character variability in early theriodont evolution. *Frontiers in Ecology and Evolution* 7:464.

Rawson JRG, Porro LB, Martin-Silverstone E and Rayfield EJ. 2021. Osteology and digital reconstruction of the skull of the early tetrapod *Whatcheeria deltae*. *Journal of Vertebrate Paleontology* e1927749.

Ray S. 2000. Endothiodont dicynodonts from the late Permian Kundaram Formation, India. *Palaeontology* 43(2): 375 – 404.

Reisz RR. 1981. A diapsid reptile from the Pennsylvanian of Kansas. *Special Publication of the Museum of Natural History, University of Kansas* 7: 1 – 77.

Reisz RR. 1986. Encyclopedia of Palaeoherpetology. Part 17: Pelycosauria. Gustav Fischer Verlag, Stuttgart, New York.

Reisz RR, Berman DS and Scott D. 1992. The cranial anatomy and relationships of *Secodontosaurus*, an unusual mammal-like reptile (Synapsida: Sphenacodontidae) from the early Permian of Texas. *Zoological Journal of the Linnean Society* 104: 127 – 184.

Reisz RR, MacDougall MJ, LeBlanc ARH, Scott D and Nagesan RS. 2020. Lateralized Feeding Behavior in a Paleozoic Reptile. *Current Biology* 30(12): 2374 – 2378.

Reisz RR, Modesto SP and Scott DM. 2011. A new Early Permian reptile and its significance in early diapsid evolution. *Proceedings of the Royal Society B* 278: 3731 – 3737.

Reisz RR, Müller J, Tsuji L and Scott D. 2007. The cranial osteology of *Belebey vegrandis* (Parareptilia: Bolosauridae), from the Middle Permian of Russia, and its bearing on reptilian evolution. *Zoological Journal of the Linnean Society* 151: 191 – 214.

Reisz RR and Scott D. 2002. *Owenetta kitchingorum*, sp. nov., a small parareptile (Procolophonia: Owenettidae) from the Lower Triassic of South Africa. *Journal of Vertebrate Paleontology* 22(2): 244 – 256.

Reisz RR, Scott D and Modesto SP. 2022. Cranial Anatomy of the Caseid Synapsid *Cotylorhynchus romeri*, a Large Terrestrial Herbivore From the Lower Permian of Oklahoma, U.S.A. *Frontiers in Earth Science* 10:847560.

Romer AS. 1963. The larger embolomerous amphibians of the American Carboniferous. *Bulletin of the Museum of Comparative Zoology at Harvard College* 128(9): 415 – 454.

Romer AS and Price LW. 1940. Review of the Pelycosauria. *Geological Society of America* 28.

Rowe DCT, Bevitt JJ and Reisz RR. 2023. Skeletal anatomy of the early Permian parareptile *Delorhynchus* with new information provided by neutron tomography. *PeerJ* 11: e15935.

Ruta M and Clack JA. 2006. A review of *Silvanerpeton miripedes*, a stem amniote from the Lower Carboniferous of East Kirkton, West Lothian, Scotland. *Transactions of the Royal Society of Edinburgh: Earth Sciences* 97: 31 – 63.

Ruta M, Clack JA and Smithson TR. 2020. A review of the stem amniote *Eldeceeon rolfei* from the Viséan of East Kirkton, Scotland. *Earth and Environmental Science Transactions of the Royal Society of Edinburgh* 111(3): 173 – 192.

Ruta M, Milner AR and Coates MI. 2002. The tetrapod *Caerorhachis bairdi* Holmes and Carroll from the Lower Carboniferous of Scotland*. Transactions of the Royal Society of Edinburgh: Earth Sciences* 92: 229 – 261.

Rybczynski N. 2000. Cranial anatomy and phylogenetic position of *Suminia getmanovi*, a basal anomodont (Amniota: Therapsida) from the Late Permian of Eastern Europe. *Zoological Journal of the Linnean Society* 130: 329 – 373.

Sawin HJ. 1941. The cranial anatomy of *Eryops megacephalus*. *Bulletin of the Museum of Comparative Zoology at Harvard College* 88(5): 407 – 463.

Schoch RR and Witzmann F. 2009. Osteology and relationships of the temnospondyl genus *Sclerocephalus*. *Zoological Journal of the Linnean Society* 157: 135 – 168.

Sequeira SEK. 2003. The skull of Cochleosaurus bohemicus Frič, a temnospondyl from the Czech Republic (Upper Carboniferous) and cochleosaurid interrelationships. *Transactions of the Royal Society of Edinburgh: Earth Sciences* 94: 21 – 43.

Shi Y-T and Liu J. 2023. The tetrapod fauna of the upper Permian Naobaogou Formation of China: 10. *Jimusaria monanensis* sp. nov. (Dicynodontia) shows a unique epipterygoid. *PeerJ* 11:e15783.

Sidor CA. 2003. Evolutionary trends and the origin of the mammalian lower jaw. *Paleobiology* 29(4): 605 – 640.

Smithson TR. 1980. A new labyrinthodont amphibian from the Carboniferous Scotland. *Palaeontology* 23(4): 915 – 923.

Spindler F. 2015. The basal Sphenacodontia - systematic revision and evolutionary implications. PhD thesis. Freiberg, 385 pp.

Spindler F. 2016. Morphological description and taxonomic status of *Palaeohatteria* and *Pantelosaurus* (Synapsida: Sphenacodontia). *Freiberger Forschungshefte* C 550: 1 – 57.

Spindler F. 2020. The skull of *Tetraceratops insignis* (Synapsida, Sphenacodontia). *Palaeo Vertebrata* 43: e1.

Spindler F, Scott D and Reisz RR. 2015. New information on the cranial and postcranial anatomy of the early synapsid *Ianthodon schultzei* (Sphenacomorpha: Sphenacodontia), and its evolutionary significance. *Fossil Record* 18: 17 – 30.

Spindler F, Werneburg R, Schneider JW, Luthardt L, Annacker V and Rößler R. 2018. First arboreal ’pelycosaurs’ (Synapsida: Varanopidae) from the early Permian Chemnitz Fossil Lagerstätte, SE Germany, with a review of varanopid phylogeny. *Paläontologisches Zeitschrift* 92: 315 – 364.

Sullivan C and Reisz RR. 2005. Cranial anatomy and taxonomy of the late Permian dicynodont *Diictodon*. *Annals of Carnegie Museum* 74: 45 – 75.

Tsuji LA. 2013. Anatomy, cranial ontogeny and phylogenetic relationships of the pareiasaur *Deltavjatia rossicus* from the Late Permian of central Russia. *Earth and Environmental Science Transactions of the Royal Society of Edinburgh* 104: 1 – 42.

Tverdohklebov VP, Tverdohklebova GI, Minihk AV, Surkov MV and Benton MJ. 2005. Upper Permian vertebrates and their sedimentological context in the South Urals, Russia. *Earth-Science Reviews* 69: 27 – 77.

Vallin G and Laurin M. 2004. Cranial morphology and affinities of *Microbrachis*, and a reappraisal of the phylogeny and lifestyle of the first amphibians. *Journal of Vertebrate Paleontology* 24: 56 – 72.

Van den Brandt MJ, Rubidge BS, Benoit J and Abdala F. 2021. Cranial morphology of the middle Permian pareiasaur *Nochelesaurus alexanderi* from the Karoo Basin of South Africa. *Earth and Environmental Science Transactions of the Royal Society of Edinburgh* 112: 29 – 49.

Van den Heever JA. 1994. The cranial anatomy of the early Therocephalia (Amniota: Therapsida). *Annales University of Stellenbosch* 1: 1 – 59.

Vaughn PP. 1955. The Permian reptile *Araeoscelis* restudied. *Bulletin of the Museum of Comparative Zoology at Harvard College* 113(5): 305 – 469.

Watson DMS. 1954. On *Bolosaurus* and the origin and classification of reptiles. *Bulletin of the Museum of Comparative Zoology at Harvard College* 9: 299 – 450.

Watson DMS. 1957. On *Millerosaurus* and the Early History of the Sauropsid Reptiles. *Philosophical Transactions of the Royal Society of London B* 240(673): 325 – 400.

Welles SP. 1941. The mandible of a diadectid cotylosaur. *University of California Publications in Geological Sciences*: 423 – 431.

Wellstead CF. 1991. Taxonomic revision of the Lysorophia, Permo-Carboniferous lepospondyl amphibians. *Bulletin of the American Museum of Natural History* 209: 1 – 90.

Werneburg R, Štamberg S and Steyer J-S. 2020. A new stereospondylomorph, *Korkonterpeton kalnense* gen. et sp. nov., from lower Permian of the Czech Krkonoše Piedmont Basin and a redescription of *Intasuchus silvicola* from the lower Permian of Russia (Temnospondyli, Amphibia). *Fossil Imprint* 76(2): 217 – 242.

Werneburg R and Steyer J-S. 1999. Redescription of the holotype of *Actinodon frossardi* (Amphibia, Temnospondyli) from the Lower Permian of the Autun basin (France). *Geobios* 32(4): 599 – 607.

Werneburg R, Witzmann F, Rinehart L, Fischer J and Voigt S. 2024. A new eryopid temnospondyl from the Carboniferous–Permian boundary of Germany. *Journal of Paleontology* 97(6): 1251 – 1281.

Williston SW. 1913. The skulls of *Araeoscelis* and *Casea*, Permian reptiles. *The Journal of Geology* 21(8): 743 – 747.

Williston SW. 1925. Osteology of the Reptiles. Cambridge Harvard University Press. 320 pp.

Witzmann F. 2005. Cranial ontogeny of *Archegosaurus decheni*. *Transactions of the Royal Society of Edinburgh: Earth Sciences* 96(2): 131 – 162.

Witzmann F. 2013. The stratigraphically oldest eryopoid temnospondyl from the Permo-Carboniferous Saar- Nahe Basin, Germany. *Paläontologisches Zeitschrift* 87: 259 – 267.

Xiong Z. 2023. A new species of pantylid ‘microsaur’ from the Carboniferous of Nova Scotia and implications for its ecology. MSc thesis. Carleton University, Ottawa, Ontario, Canada. 148 pp.
