# Supplementary figures and images for: Mandibular form and function is more disparate in amniotes than in non-amniote tetrapods from the late Palaeozoic (part 2 of 2)
Source: PeerJ. 2025 Nov 26;13:e20243. doi: 10.7717/peerj.20243 (PMC12664332; doi:10.7717/peerj.20243)

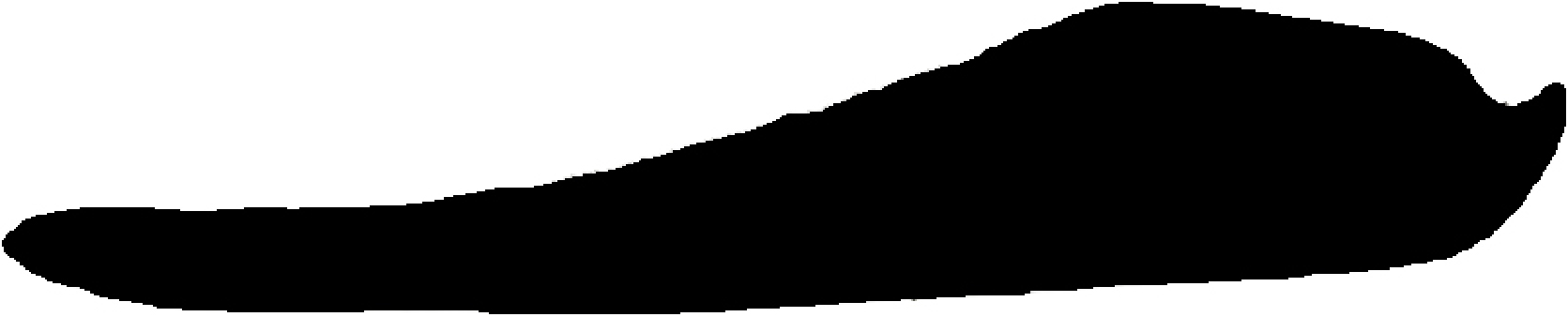

Supplement: Supplemental Information 6 [file peerj-13-20243-s006.zip › SUPPLEMENTARY FILE 7 Code_R2/Code shape lateral/Silhouette_lateral/Protorothyris_archeri.jpg]

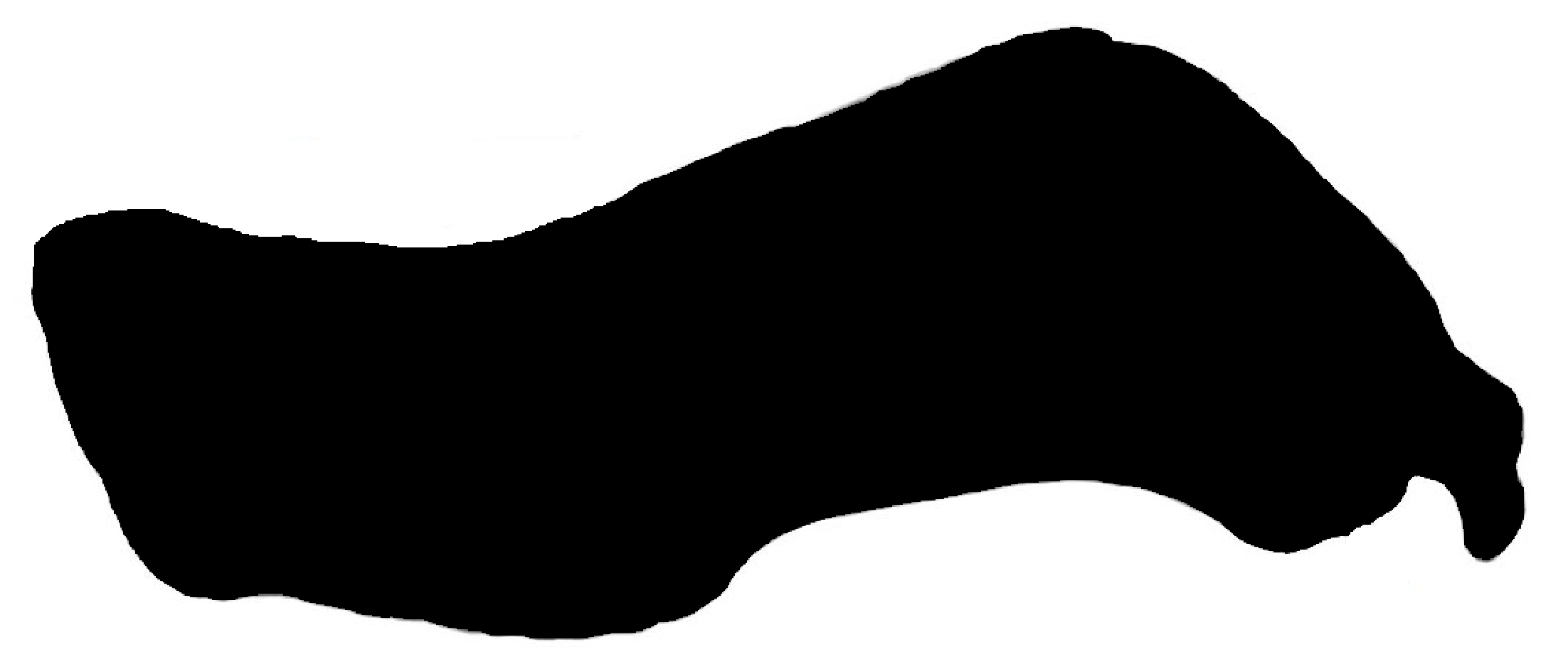

Supplement: Supplemental Information 6 [file peerj-13-20243-s006.zip › SUPPLEMENTARY FILE 7 Code_R2/Code shape lateral/Silhouette_lateral/Ulemica_invisa.jpg]

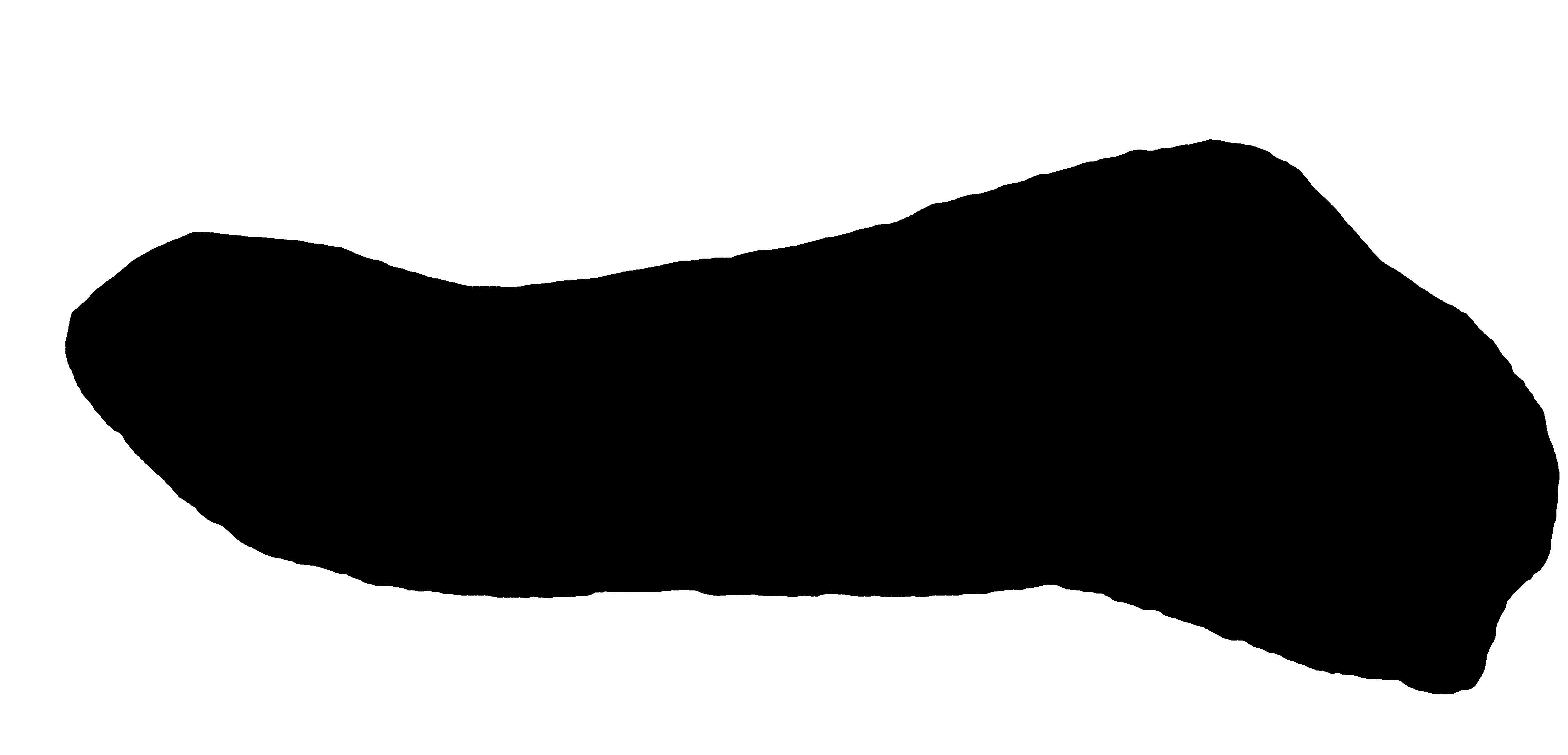

Supplement: Supplemental Information 6 [file peerj-13-20243-s006.zip › SUPPLEMENTARY FILE 7 Code_R2/Code shape lateral/Silhouette_lateral/Jonkeria_truculenta.jpg]

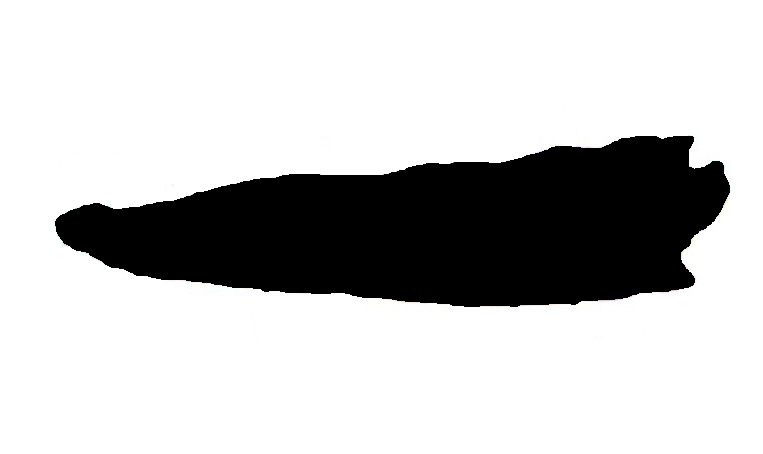

Supplement: Supplemental Information 6 [file peerj-13-20243-s006.zip › SUPPLEMENTARY FILE 7 Code_R2/Code shape lateral/Silhouette_lateral/Pantylus_cordatus.jpg]

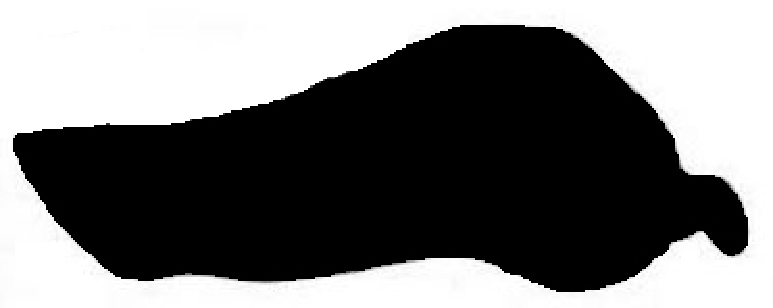

Supplement: Supplemental Information 6 [file peerj-13-20243-s006.zip › SUPPLEMENTARY FILE 7 Code_R2/Code shape lateral/Silhouette_lateral/Ulemosaurus_svigagensis.jpg]

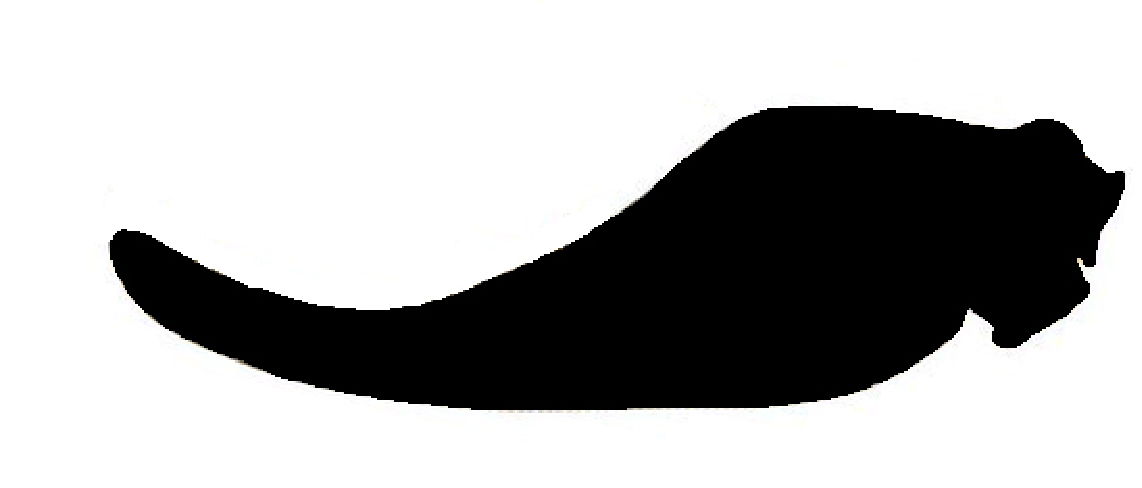

Supplement: Supplemental Information 6 [file peerj-13-20243-s006.zip › SUPPLEMENTARY FILE 7 Code_R2/Code shape lateral/Silhouette_lateral/Tetraceratops_insignis.jpg]

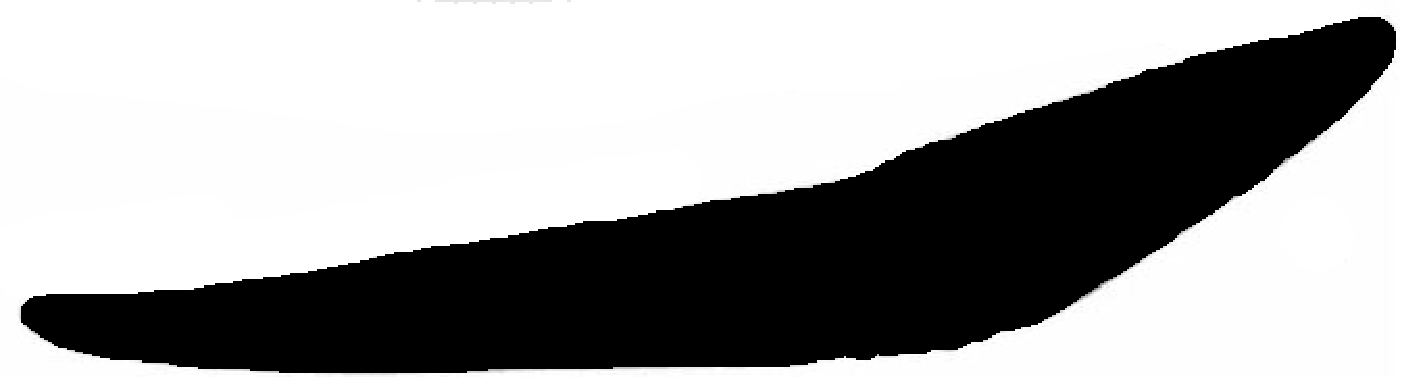

Supplement: Supplemental Information 6 [file peerj-13-20243-s006.zip › SUPPLEMENTARY FILE 7 Code_R2/Code shape lateral/Silhouette_lateral/Varanops_brevirostris.jpg]

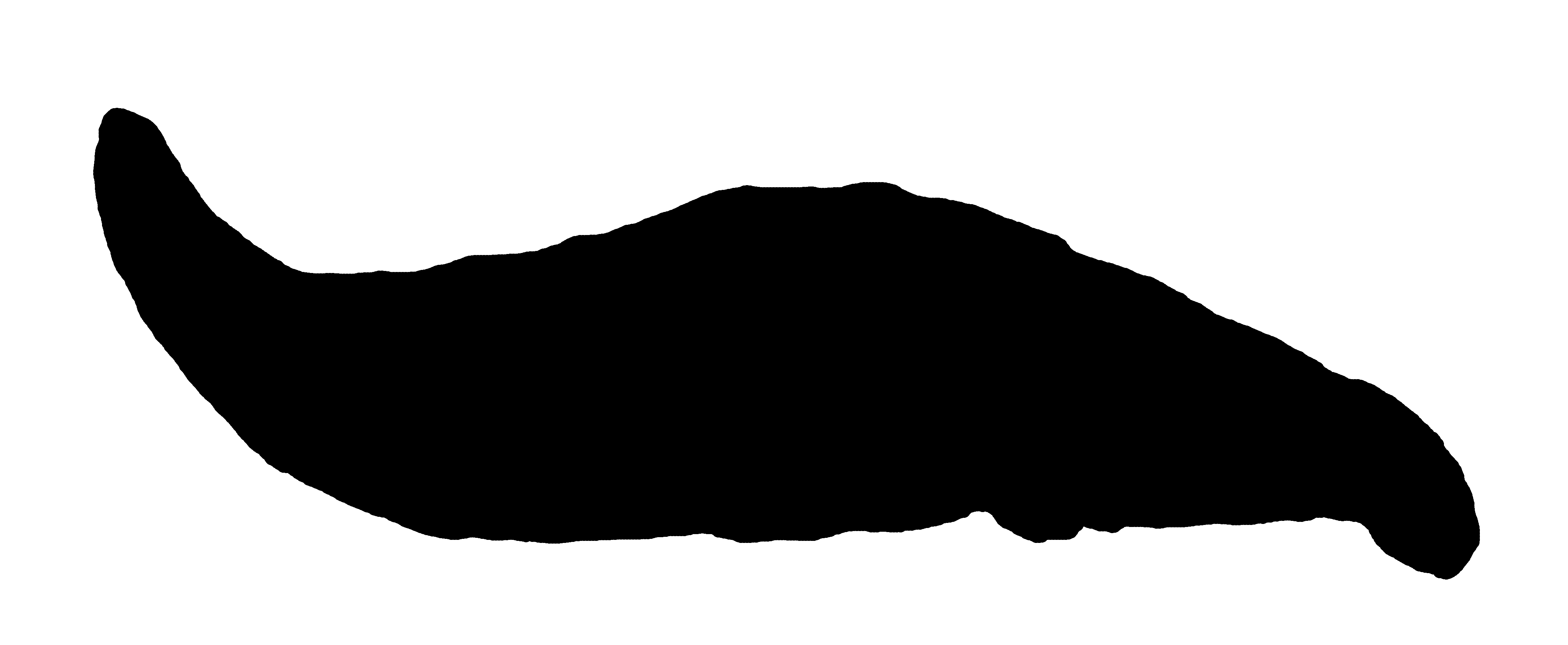

Supplement: Supplemental Information 6 [file peerj-13-20243-s006.zip › SUPPLEMENTARY FILE 7 Code_R2/Code shape lateral/Silhouette_lateral/Dicynodontoides_sp.jpg]

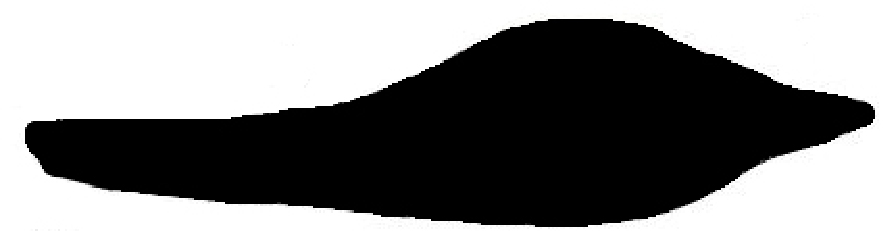

Supplement: Supplemental Information 6 [file peerj-13-20243-s006.zip › SUPPLEMENTARY FILE 7 Code_R2/Code shape lateral/Silhouette_lateral/Rhodotheratus_parvus.jpg]

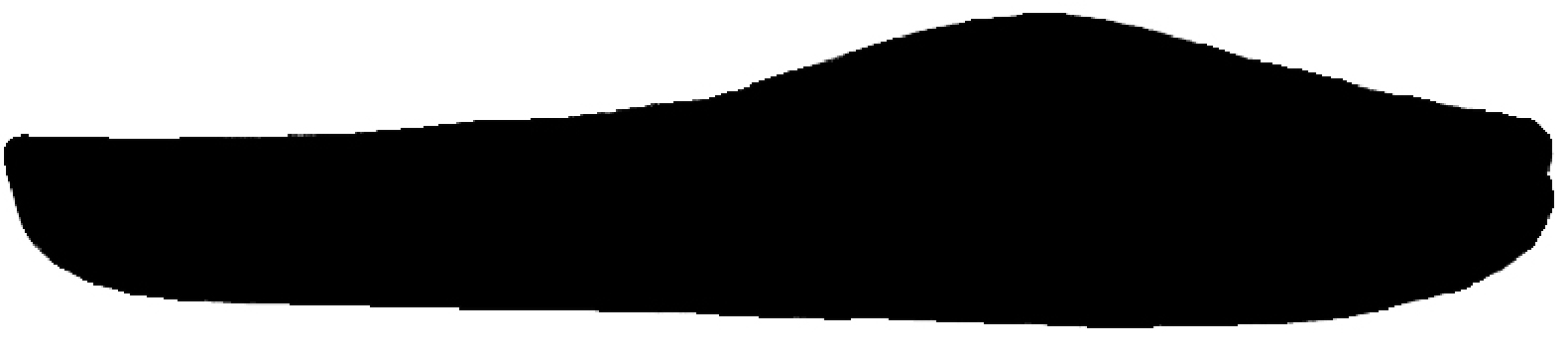

Supplement: Supplemental Information 6 [file peerj-13-20243-s006.zip › SUPPLEMENTARY FILE 7 Code_R2/Code shape lateral/Silhouette_lateral/Utegenia_shpinari.jpg]

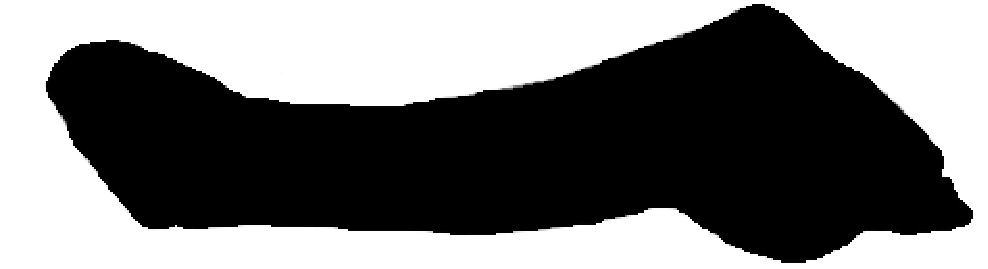

Supplement: Supplemental Information 6 [file peerj-13-20243-s006.zip › SUPPLEMENTARY FILE 7 Code_R2/Code shape lateral/Silhouette_lateral/Titanophoneus_potens.jpg]

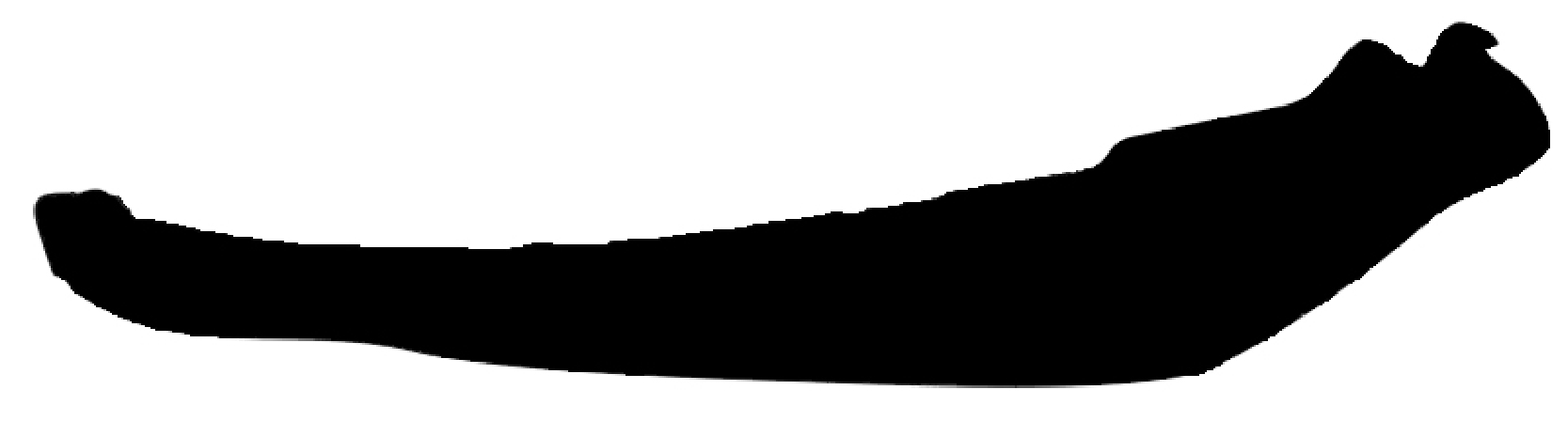

Supplement: Supplemental Information 6 [file peerj-13-20243-s006.zip › SUPPLEMENTARY FILE 7 Code_R2/Code shape lateral/Silhouette_lateral/Trimerorhachis_insignis.jpg]

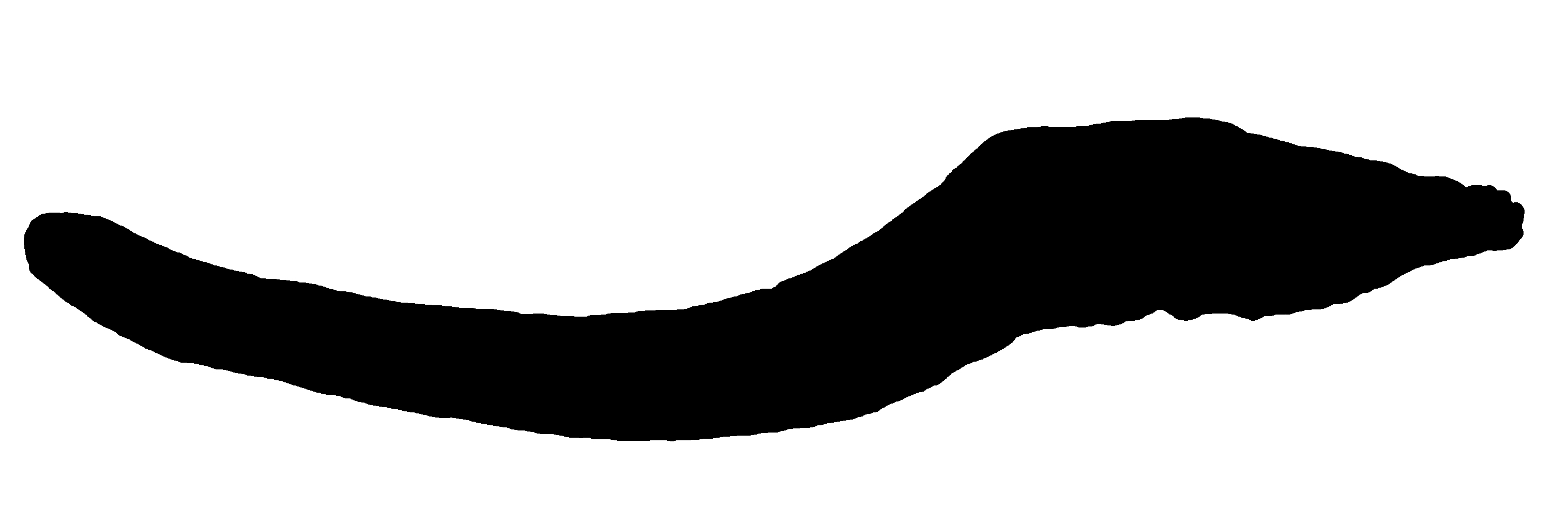

Supplement: Supplemental Information 6 [file peerj-13-20243-s006.zip › SUPPLEMENTARY FILE 7 Code_R2/Code shape lateral/Silhouette_lateral/Lycideops_longiceps.jpg]

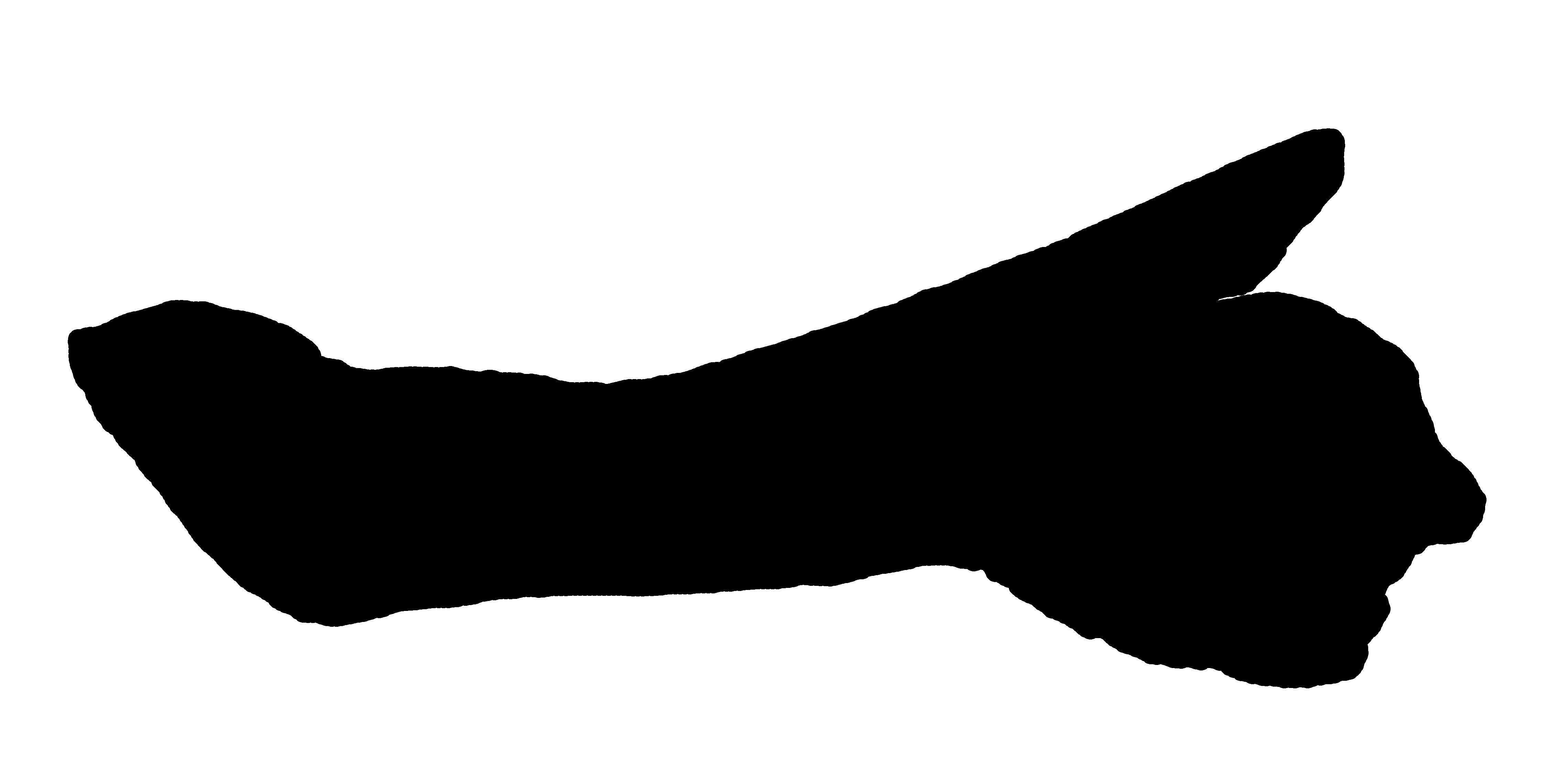

Supplement: Supplemental Information 6 [file peerj-13-20243-s006.zip › SUPPLEMENTARY FILE 7 Code_R2/Code shape lateral/Silhouette_lateral/Suchogorgon_golubevi.jpg]

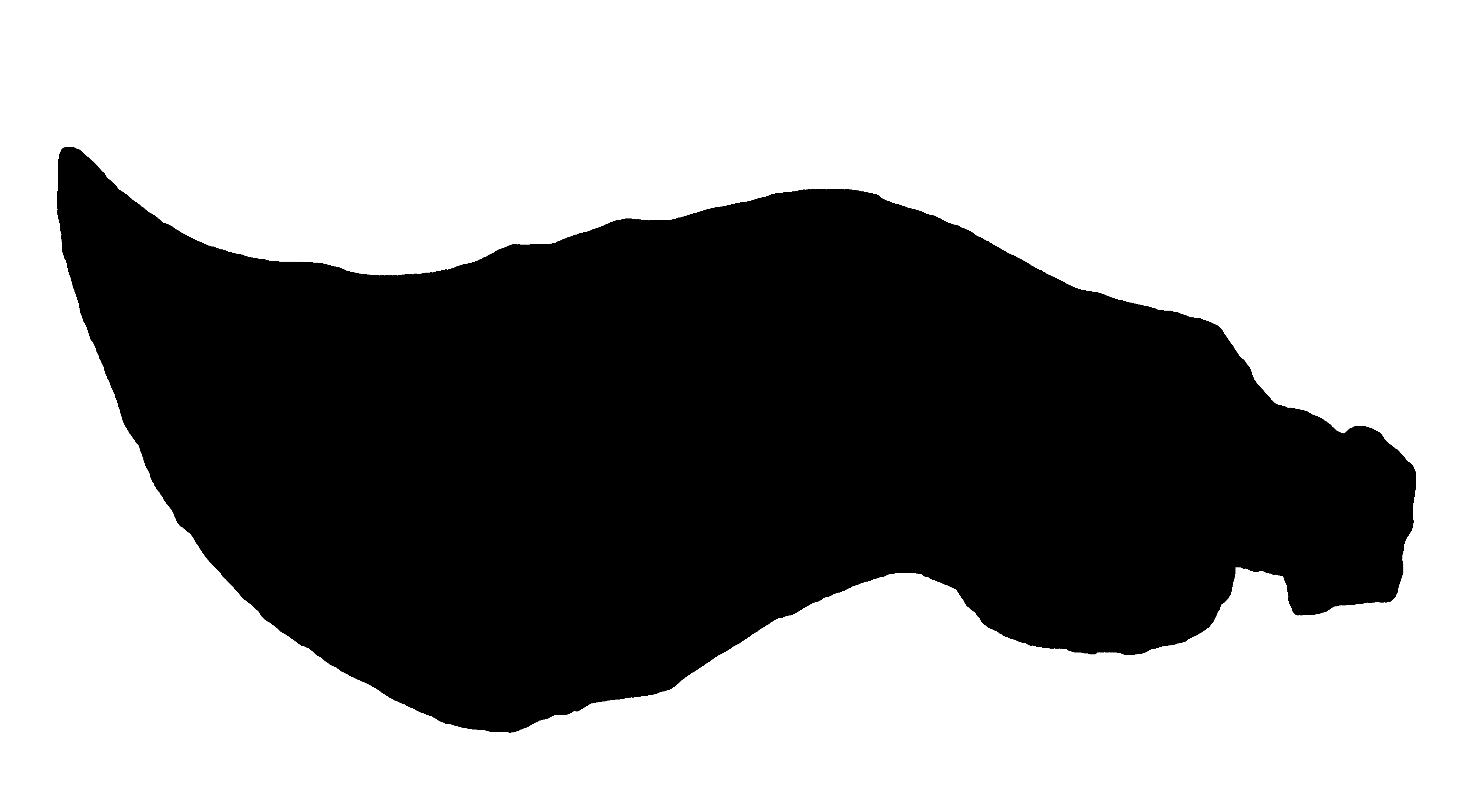

Supplement: Supplemental Information 6 [file peerj-13-20243-s006.zip › SUPPLEMENTARY FILE 7 Code_R2/Code shape lateral/Silhouette_lateral/Aulacephalodon_kapoliwacela.jpg]

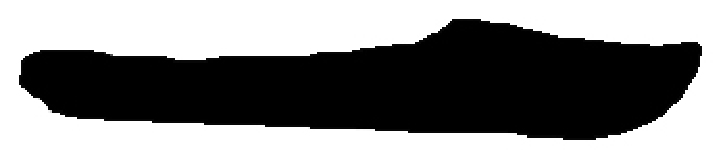

Supplement: Supplemental Information 6 [file peerj-13-20243-s006.zip › SUPPLEMENTARY FILE 7 Code_R2/Code shape lateral/Silhouette_lateral/Solenodonsaurus_janenschi.jpg]

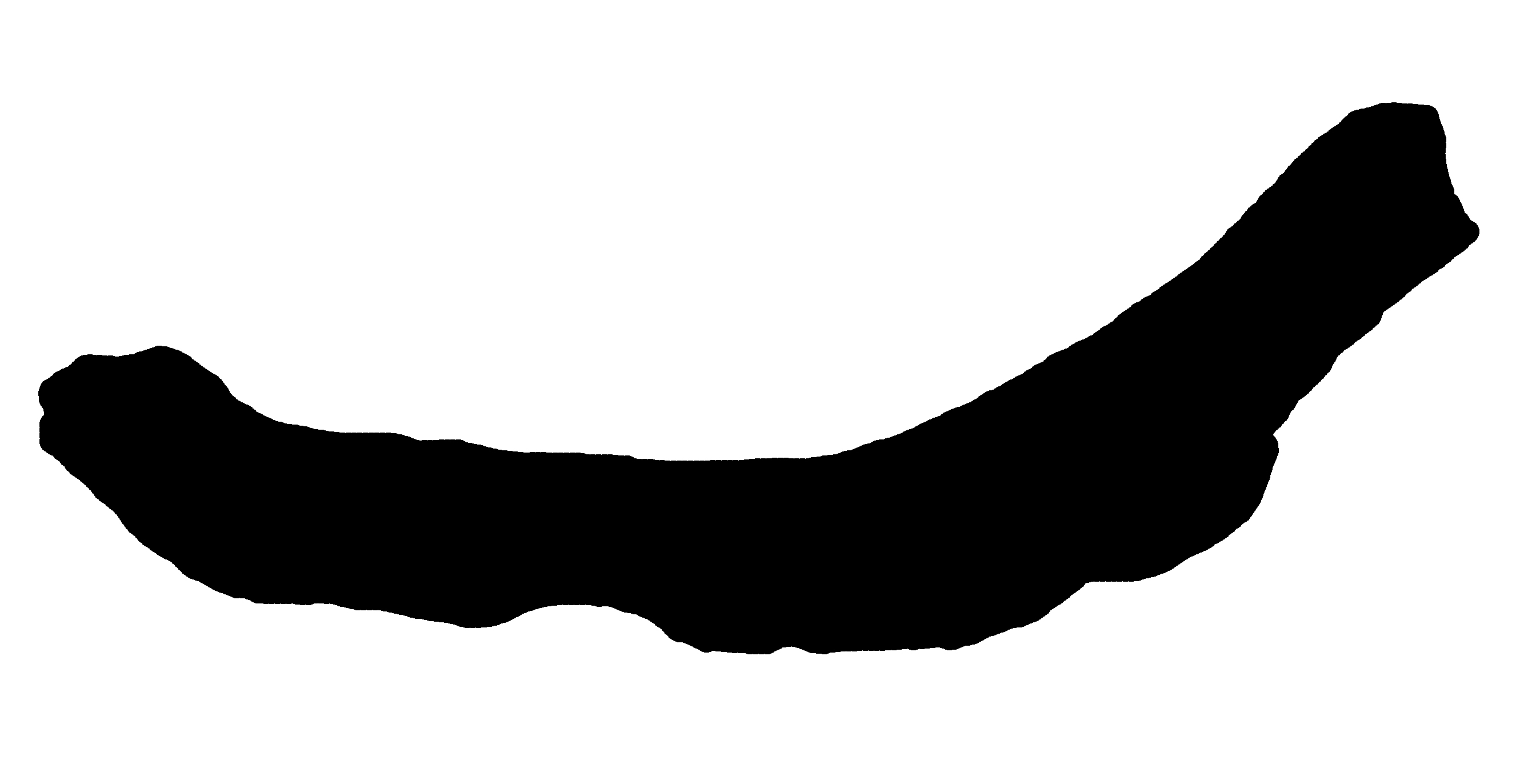

Supplement: Supplemental Information 6 [file peerj-13-20243-s006.zip › SUPPLEMENTARY FILE 7 Code_R2/Code shape lateral/Silhouette_lateral/Perplexisaurus_foveatus.jpg]

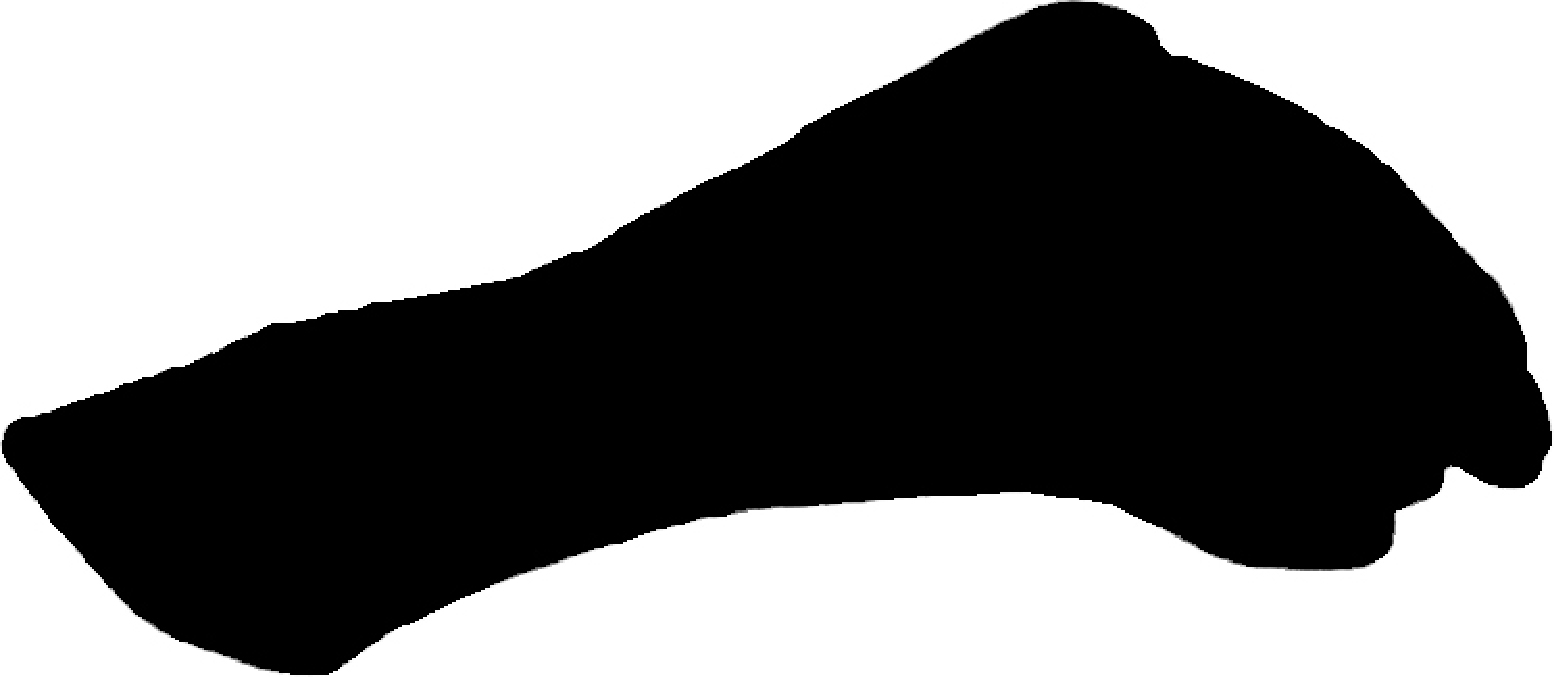

Supplement: Supplemental Information 6 [file peerj-13-20243-s006.zip › SUPPLEMENTARY FILE 7 Code_R2/Code shape lateral/Silhouette_lateral/Moschognathus_whaitsi.jpg]

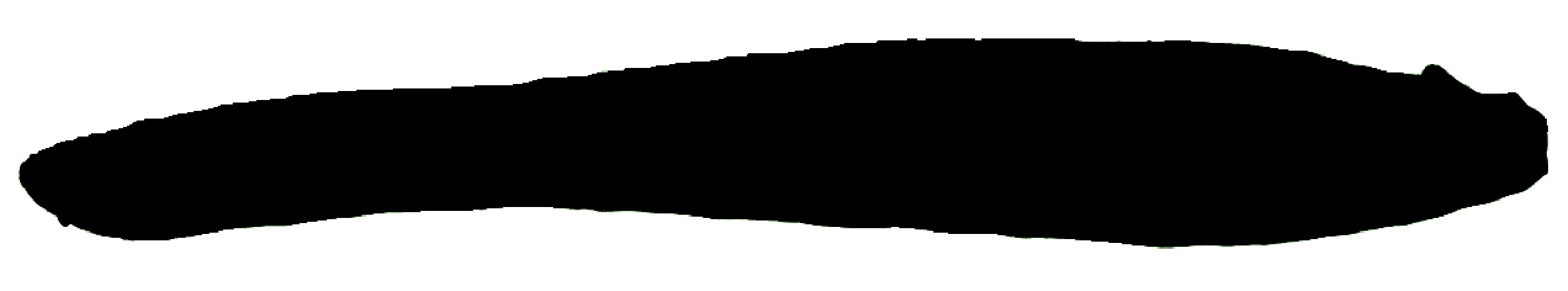

Supplement: Supplemental Information 6 [file peerj-13-20243-s006.zip › SUPPLEMENTARY FILE 7 Code_R2/Code shape lateral/Silhouette_lateral/Sigournea_multidentata.jpg]

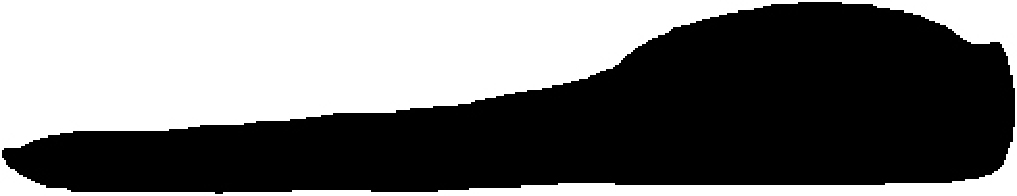

Supplement: Supplemental Information 6 [file peerj-13-20243-s006.zip › SUPPLEMENTARY FILE 7 Code_R2/Code shape lateral/Silhouette_lateral/Petrolacosaurus_kansensis.jpg]

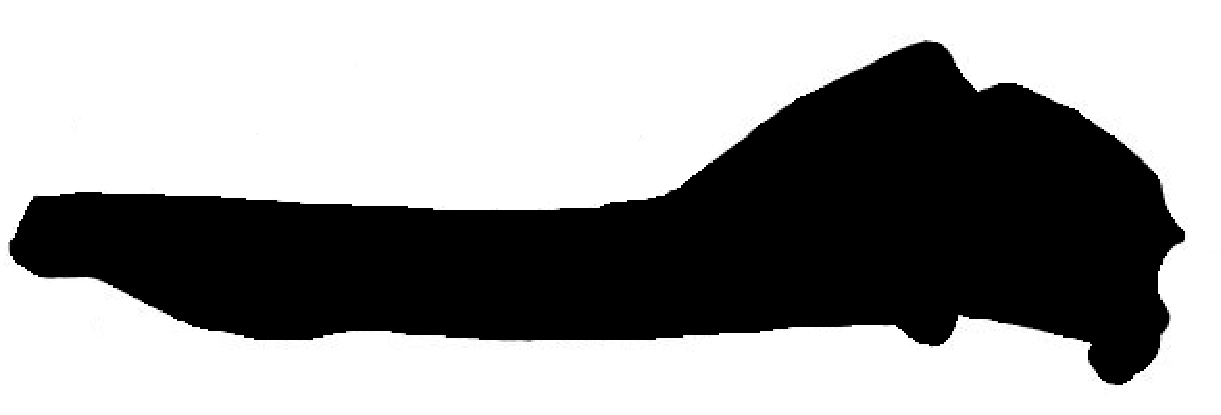

Supplement: Supplemental Information 6 [file peerj-13-20243-s006.zip › SUPPLEMENTARY FILE 7 Code_R2/Code shape lateral/Silhouette_lateral/Procynosuchus_delaharpeae.jpg]

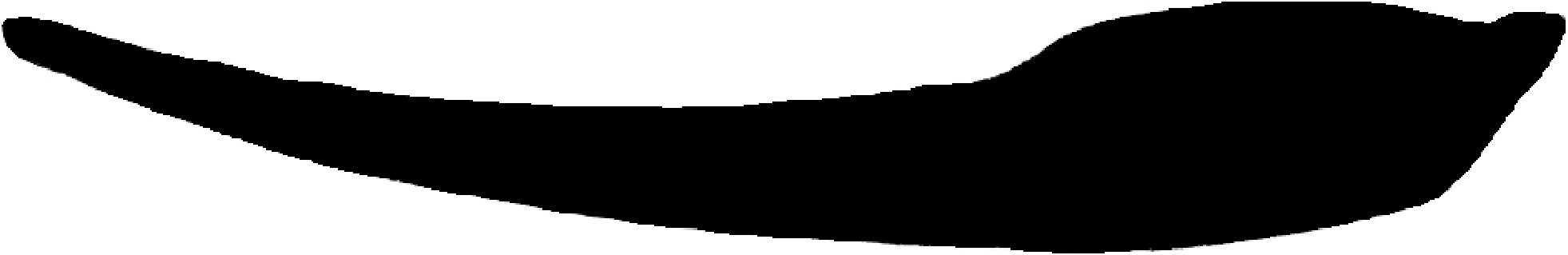

Supplement: Supplemental Information 6 [file peerj-13-20243-s006.zip › SUPPLEMENTARY FILE 7 Code_R2/Code shape lateral/Silhouette_lateral/Varanosaurus_acutirostris.jpg]

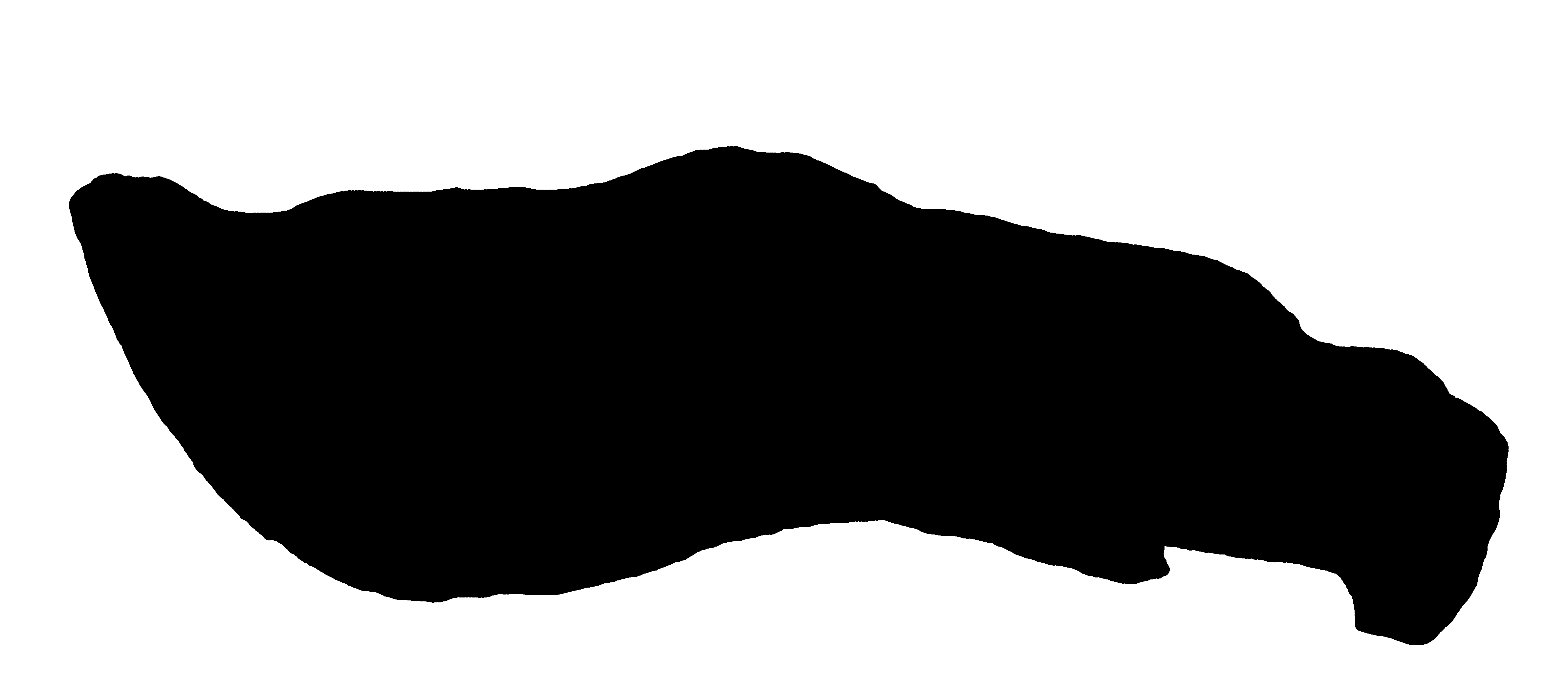

Supplement: Supplemental Information 6 [file peerj-13-20243-s006.zip › SUPPLEMENTARY FILE 7 Code_R2/Code shape lateral/Silhouette_lateral/Oudenodon_bainii.jpg]

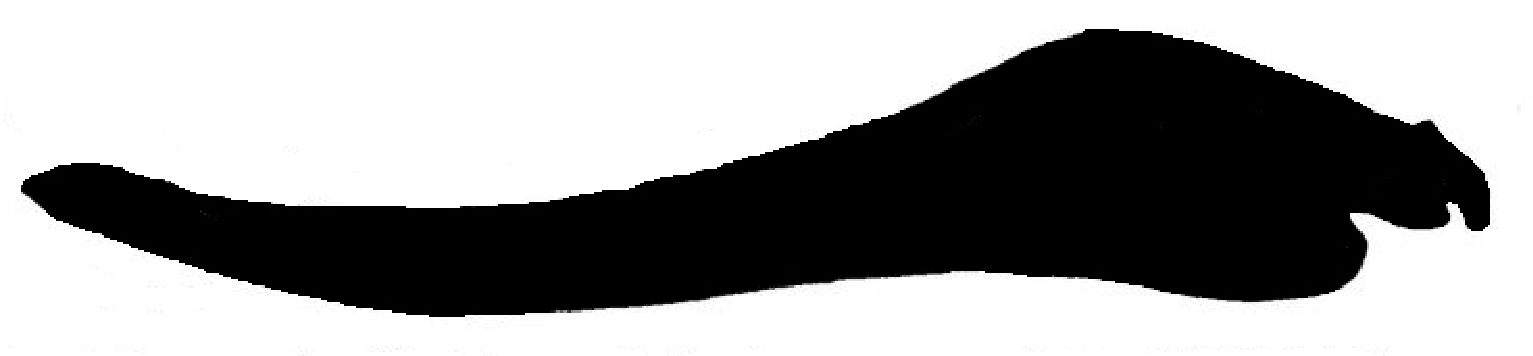

Supplement: Supplemental Information 6 [file peerj-13-20243-s006.zip › SUPPLEMENTARY FILE 7 Code_R2/Code shape lateral/Silhouette_lateral/Secodontosaurus_obtusidens.jpg]

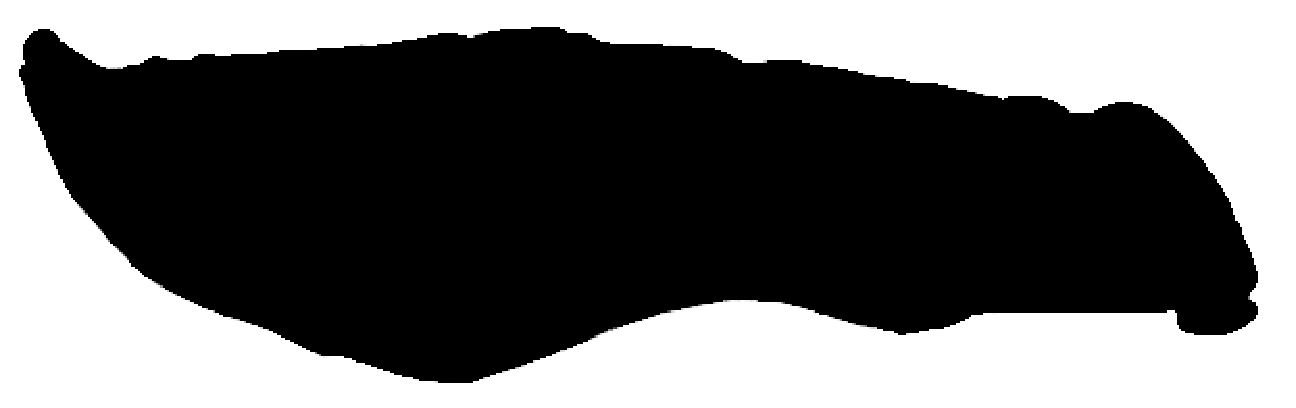

Supplement: Supplemental Information 6 [file peerj-13-20243-s006.zip › SUPPLEMENTARY FILE 7 Code_R2/Code shape lateral/Silhouette_lateral/Rhachiocephalus_magnus.jpg]

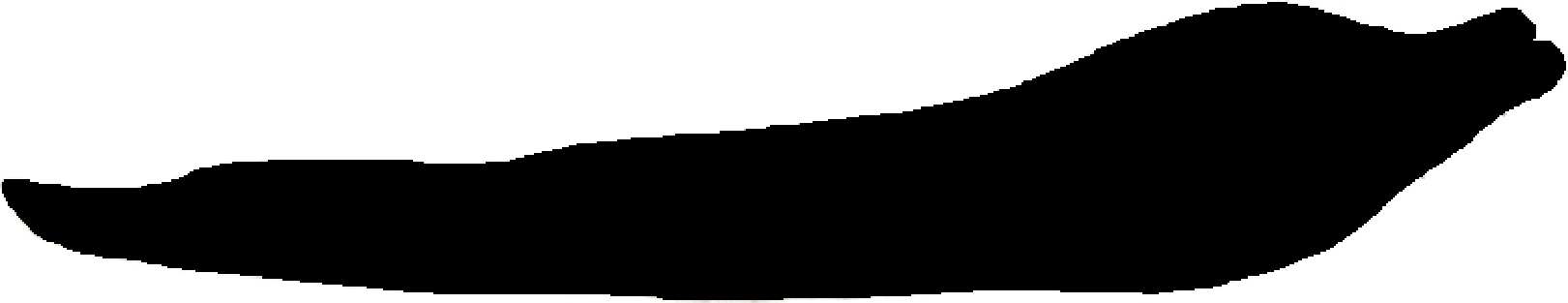

Supplement: Supplemental Information 6 [file peerj-13-20243-s006.zip › SUPPLEMENTARY FILE 7 Code_R2/Code shape lateral/Silhouette_lateral/Neldasaurus_wrightae.jpg]

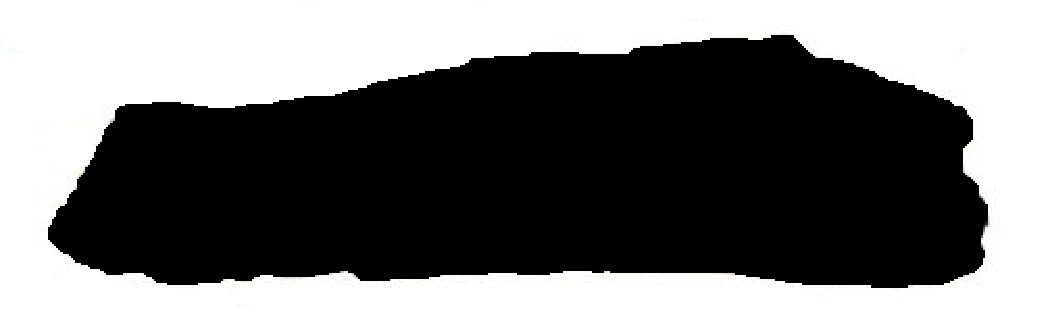

Supplement: Supplemental Information 6 [file peerj-13-20243-s006.zip › SUPPLEMENTARY FILE 7 Code_R2/Code shape lateral/Silhouette_lateral/Shihtienfenia_permica.jpg]

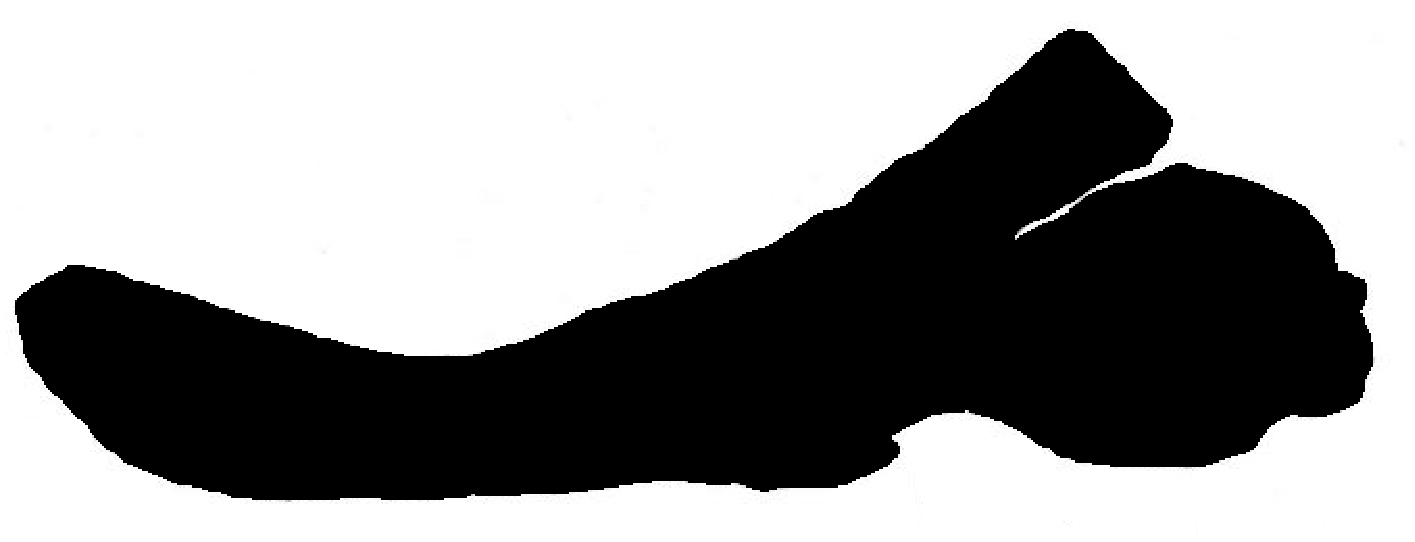

Supplement: Supplemental Information 6 [file peerj-13-20243-s006.zip › SUPPLEMENTARY FILE 7 Code_R2/Code shape lateral/Silhouette_lateral/Glanosuchus_macrops.jpg]

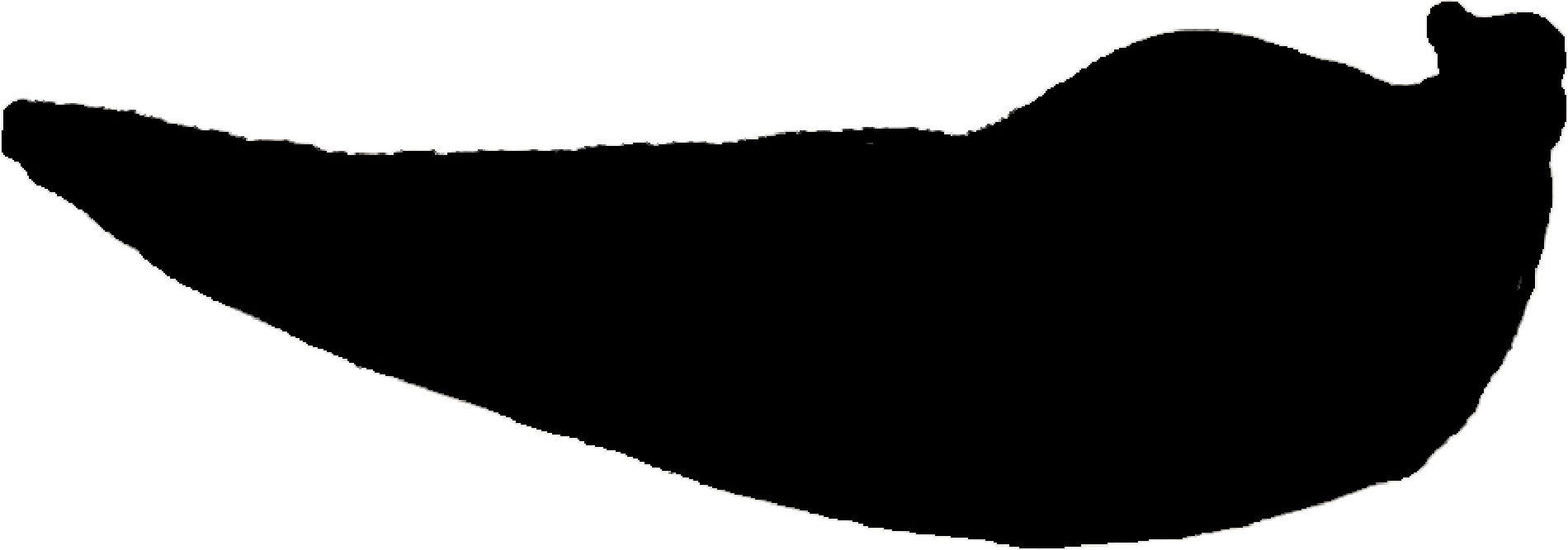

Supplement: Supplemental Information 6 [file peerj-13-20243-s006.zip › SUPPLEMENTARY FILE 7 Code_R2/Code shape lateral/Silhouette_lateral/Pholiderpeton_attheyi.jpg]

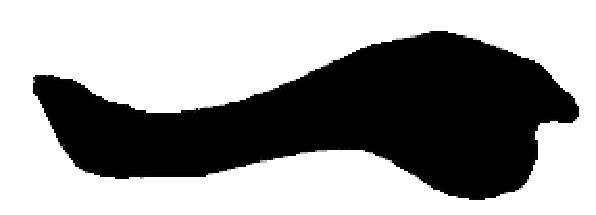

Supplement: Supplemental Information 6 [file peerj-13-20243-s006.zip › SUPPLEMENTARY FILE 7 Code_R2/Code shape lateral/Silhouette_lateral/Proburnetia_viatkensis.jpg]

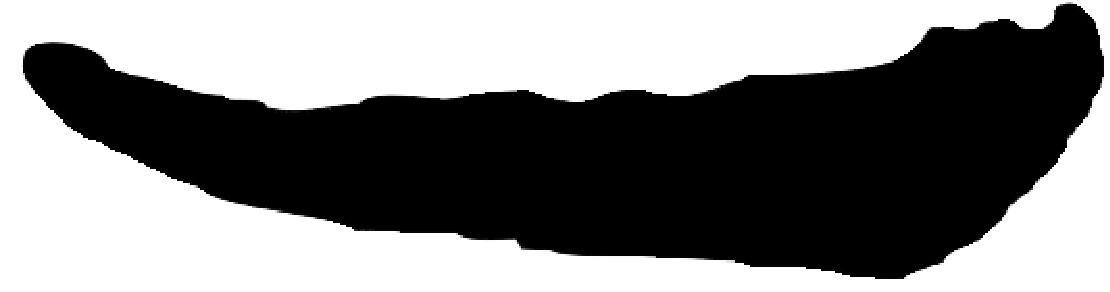

Supplement: Supplemental Information 6 [file peerj-13-20243-s006.zip › SUPPLEMENTARY FILE 7 Code_R2/Code shape lateral/Silhouette_lateral/Rhinesuchus_whaitsi.jpg]

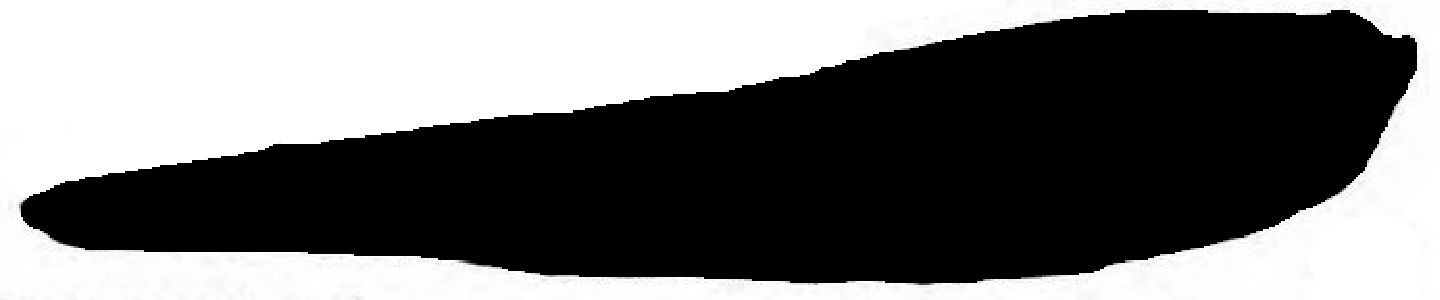

Supplement: Supplemental Information 6 [file peerj-13-20243-s006.zip › SUPPLEMENTARY FILE 7 Code_R2/Code shape lateral/Silhouette_lateral/Silvanerpeton_miripedes.jpg]

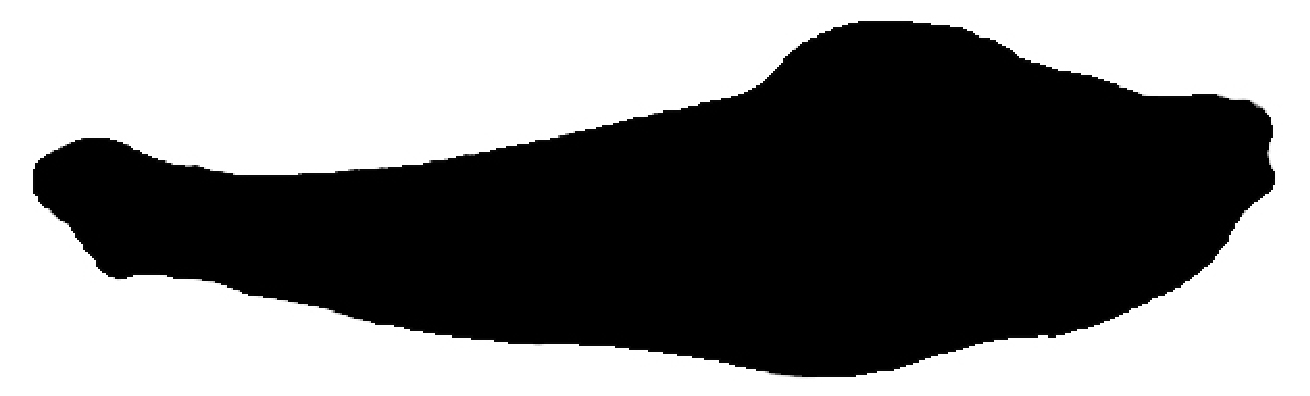

Supplement: Supplemental Information 6 [file peerj-13-20243-s006.zip › SUPPLEMENTARY FILE 7 Code_R2/Code shape lateral/Silhouette_lateral/Seymouria_baylorensis.jpg]

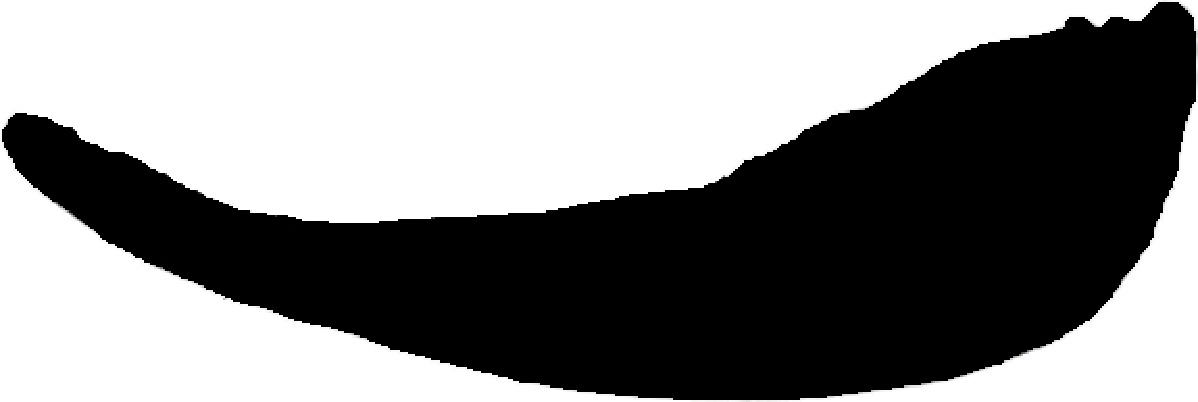

Supplement: Supplemental Information 6 [file peerj-13-20243-s006.zip › SUPPLEMENTARY FILE 7 Code_R2/Code shape lateral/Silhouette_lateral/Neopteroplax_conemaughensis.jpg]

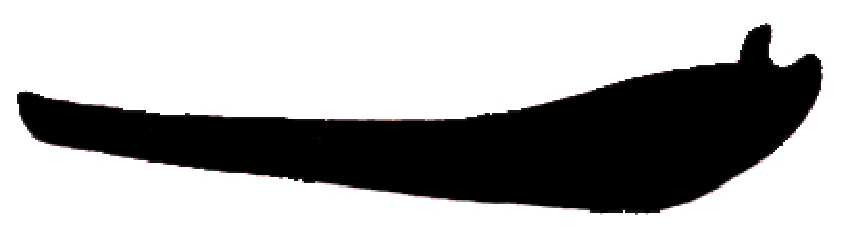

Supplement: Supplemental Information 6 [file peerj-13-20243-s006.zip › SUPPLEMENTARY FILE 7 Code_R2/Code shape lateral/Silhouette_lateral/Oestocephalus_amphiuminus.jpg]

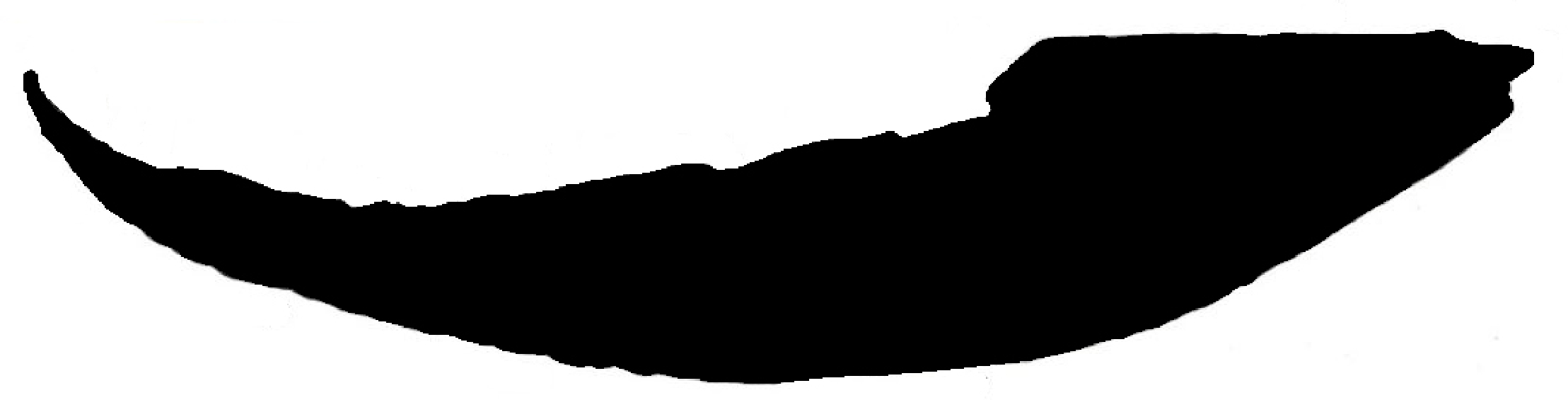

Supplement: Supplemental Information 6 [file peerj-13-20243-s006.zip › SUPPLEMENTARY FILE 7 Code_R2/Code shape lateral/Silhouette_lateral/Stenokranio_boldi.jpg]

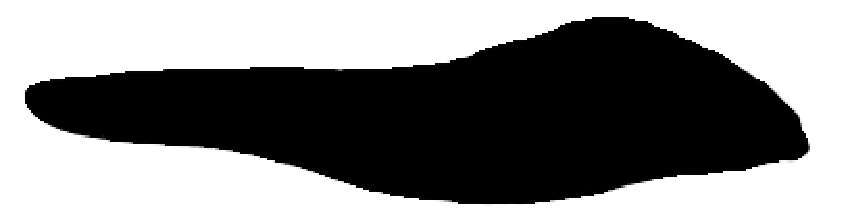

Supplement: Supplemental Information 6 [file peerj-13-20243-s006.zip › SUPPLEMENTARY FILE 7 Code_R2/Code shape lateral/Silhouette_lateral/Protocaptorhinus_pricei.jpg]

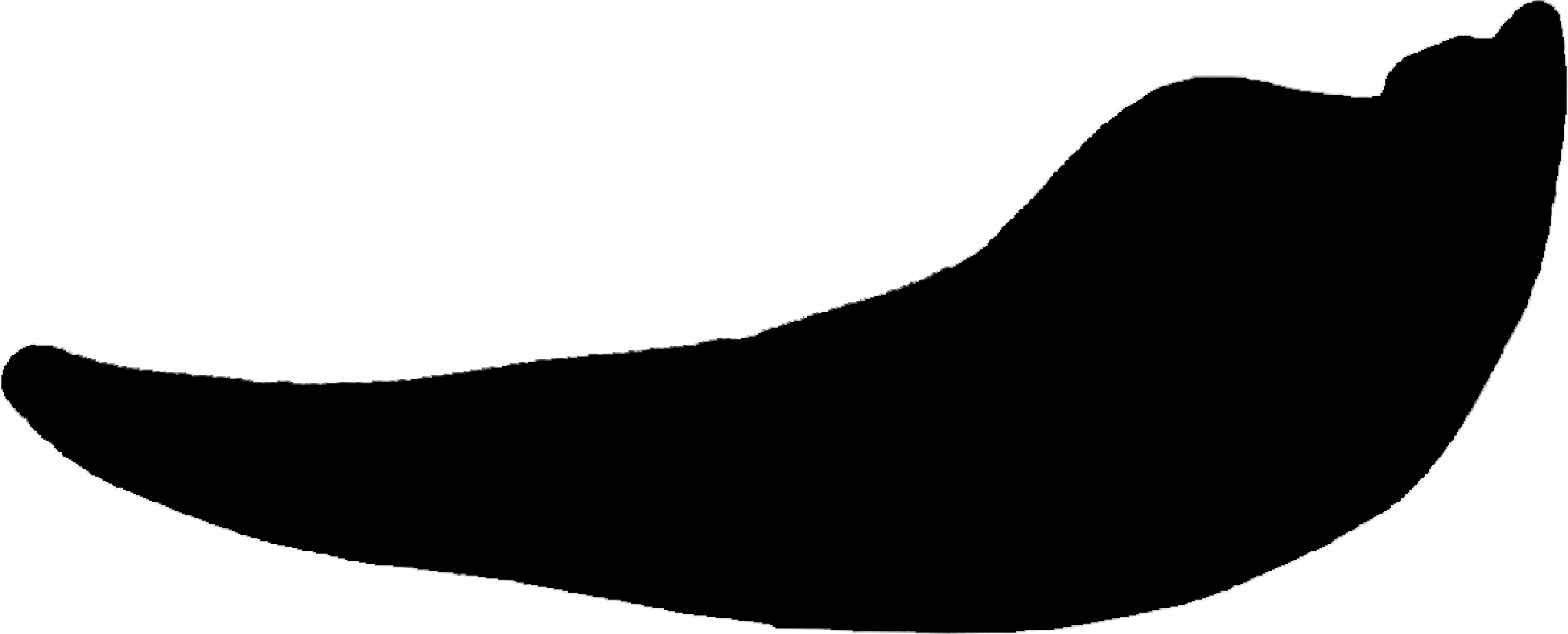

Supplement: Supplemental Information 6 [file peerj-13-20243-s006.zip › SUPPLEMENTARY FILE 7 Code_R2/Code shape lateral/Silhouette_lateral/Pholiderpeton_scutigerum.jpg]

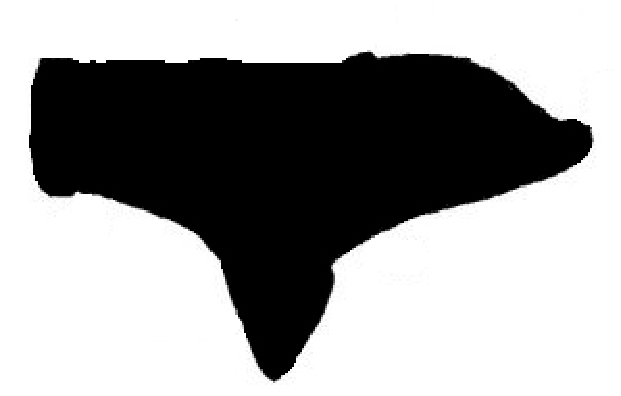

Supplement: Supplemental Information 6 [file peerj-13-20243-s006.zip › SUPPLEMENTARY FILE 7 Code_R2/Code shape lateral/Silhouette_lateral/Scutosaurus_sp.jpg]

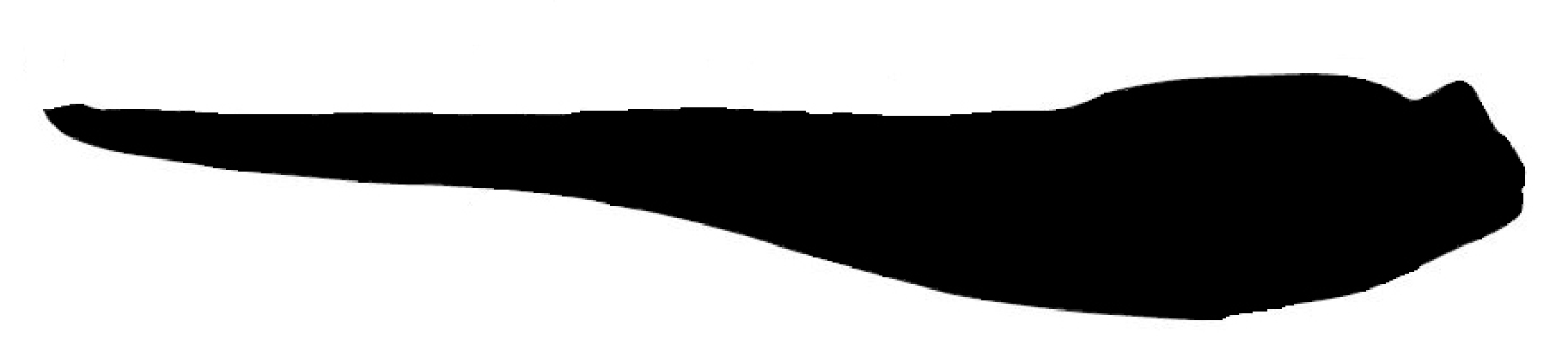

Supplement: Supplemental Information 6 [file peerj-13-20243-s006.zip › SUPPLEMENTARY FILE 7 Code_R2/Code shape lateral/Silhouette_lateral/Sauropleura_scalaris.jpg]

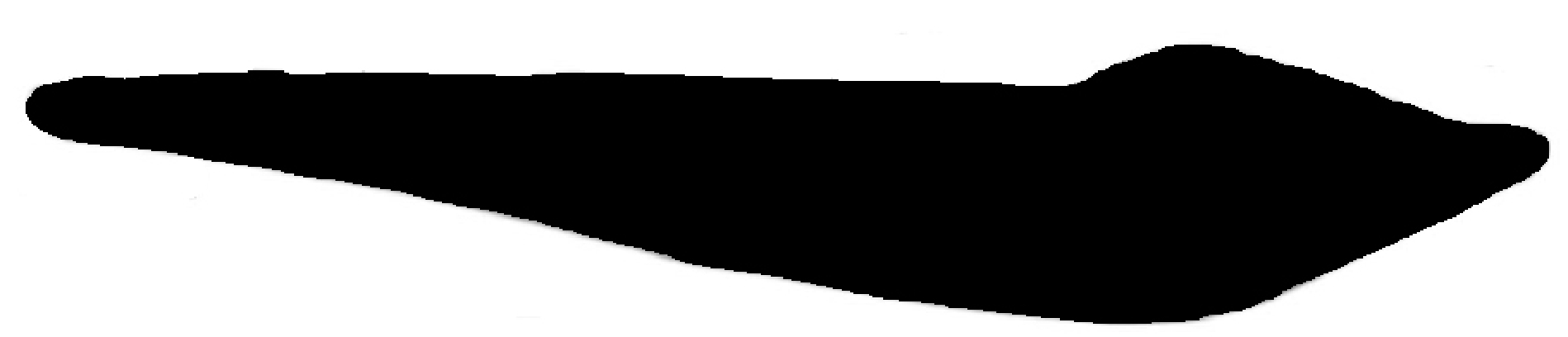

Supplement: Supplemental Information 6 [file peerj-13-20243-s006.zip › SUPPLEMENTARY FILE 7 Code_R2/Code shape lateral/Silhouette_lateral/Onchiodon_labyrinthicus.jpg]

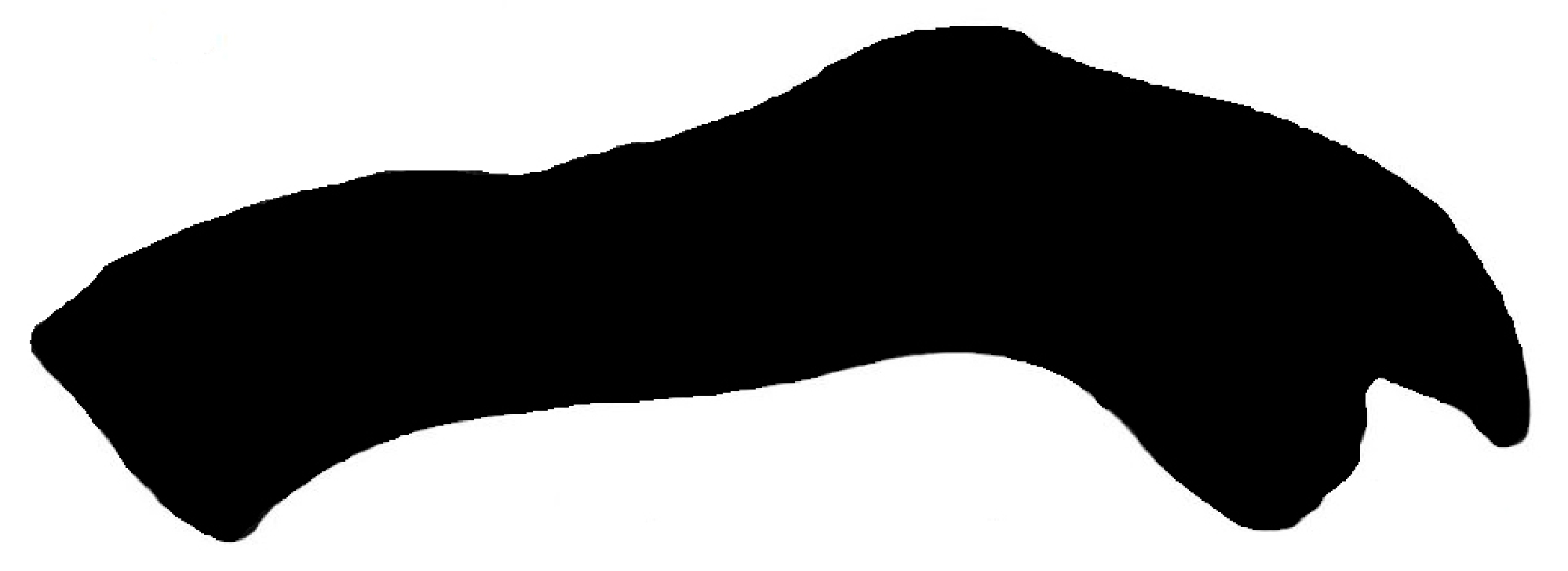

Supplement: Supplemental Information 6 [file peerj-13-20243-s006.zip › SUPPLEMENTARY FILE 7 Code_R2/Code shape lateral/Silhouette_lateral/Suminia_getmanovi.jpg]

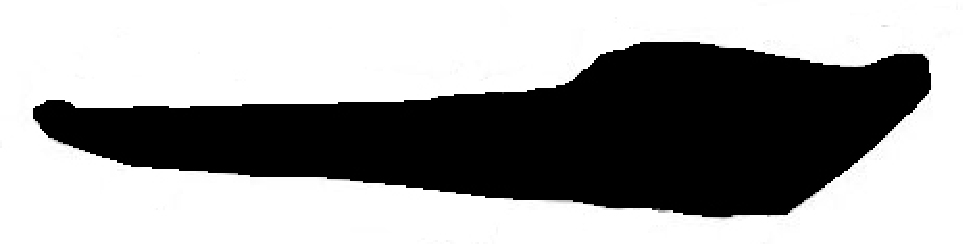

Supplement: Supplemental Information 6 [file peerj-13-20243-s006.zip › SUPPLEMENTARY FILE 7 Code_R2/Code shape lateral/Silhouette_lateral/Sclerocephalus_haeuseri.jpg]

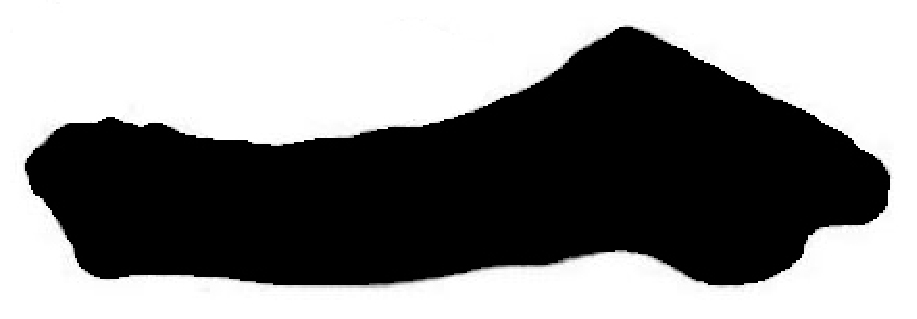

Supplement: Supplemental Information 6 [file peerj-13-20243-s006.zip › SUPPLEMENTARY FILE 7 Code_R2/Code shape lateral/Silhouette_lateral/Phthinosaurus_borissiaki.jpg]

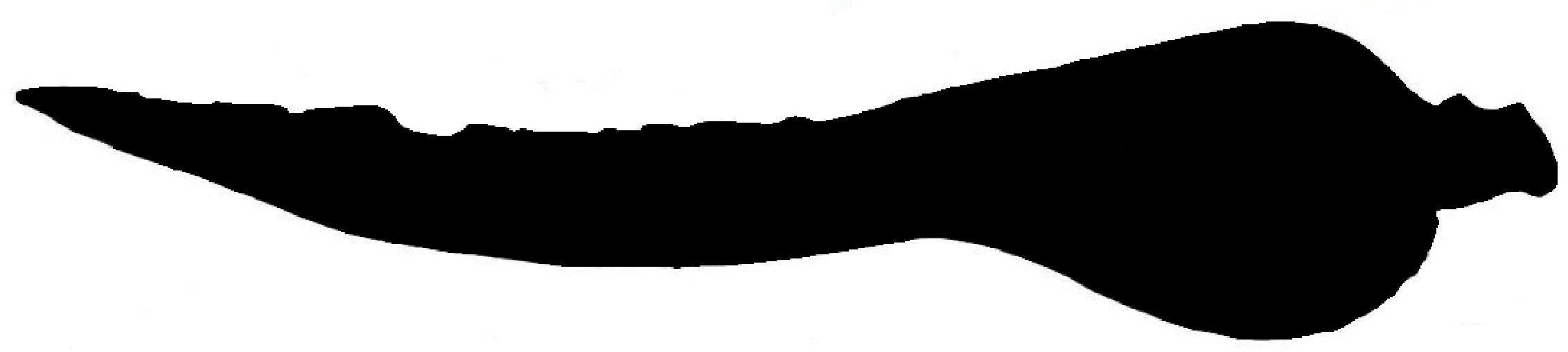

Supplement: Supplemental Information 6 [file peerj-13-20243-s006.zip › SUPPLEMENTARY FILE 7 Code_R2/Code shape lateral/Silhouette_lateral/Reiszia_gubini.jpg]

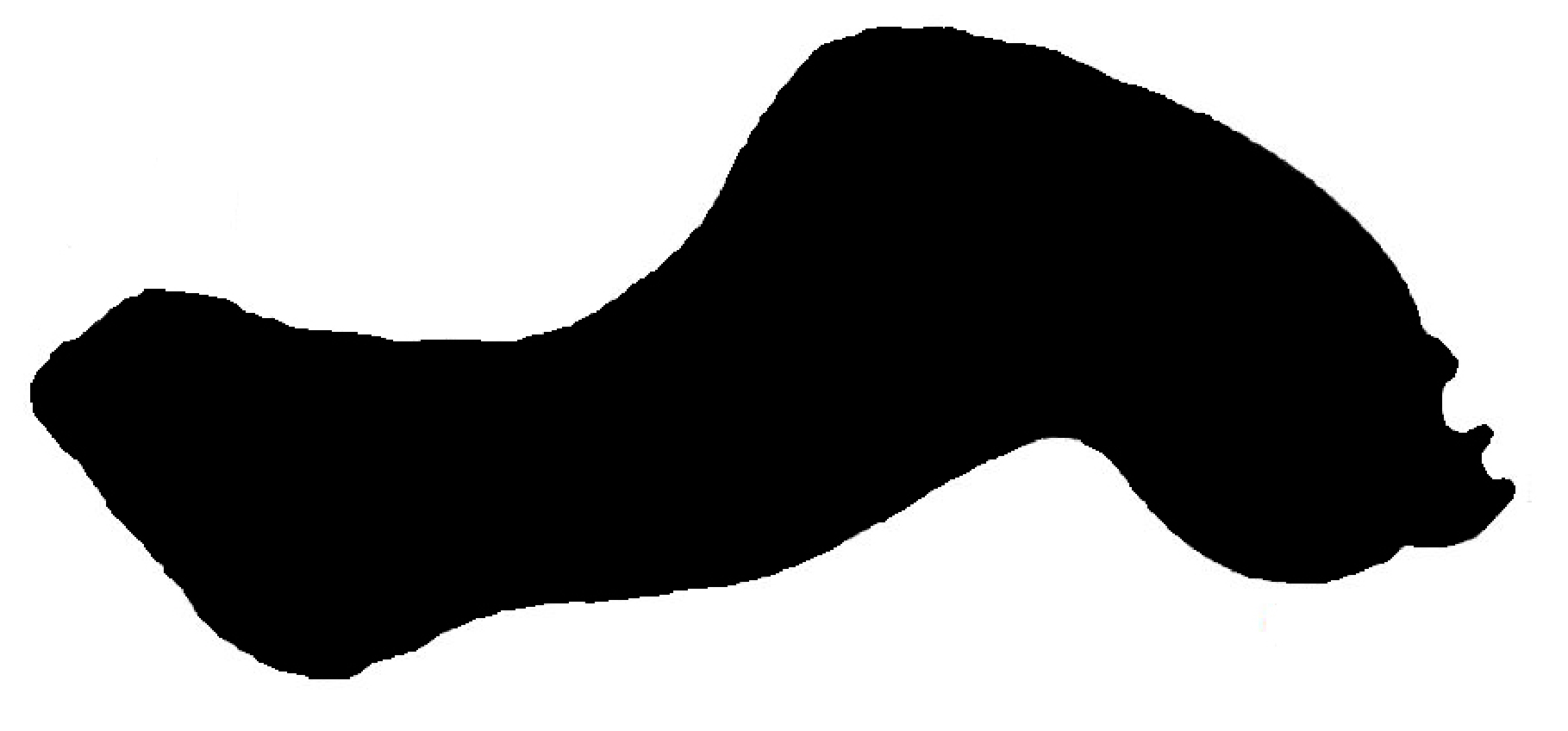

Supplement: Supplemental Information 6 [file peerj-13-20243-s006.zip › SUPPLEMENTARY FILE 7 Code_R2/Code shape lateral/Silhouette_lateral/Purlovia_maxima.jpg]

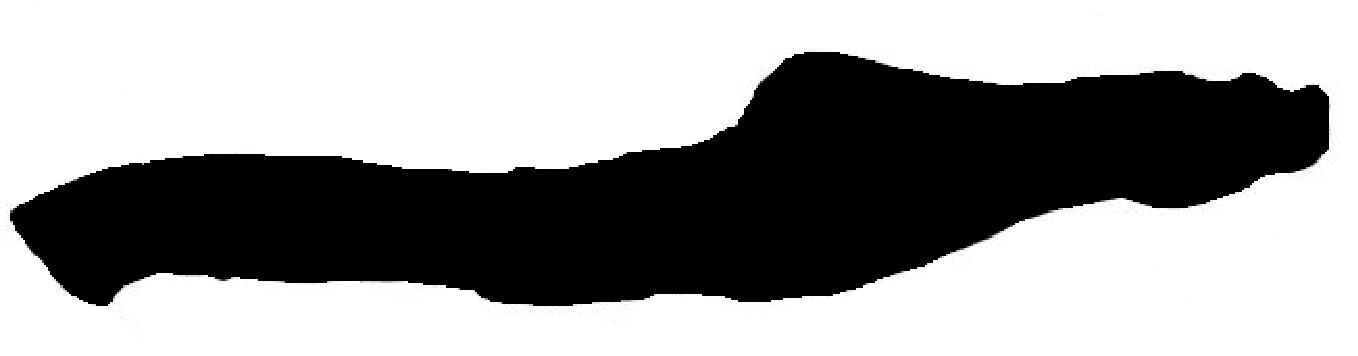

Supplement: Supplemental Information 6 [file peerj-13-20243-s006.zip › SUPPLEMENTARY FILE 7 Code_R2/Code shape lateral/Silhouette_lateral/Moradisaurus_grandis.jpg]

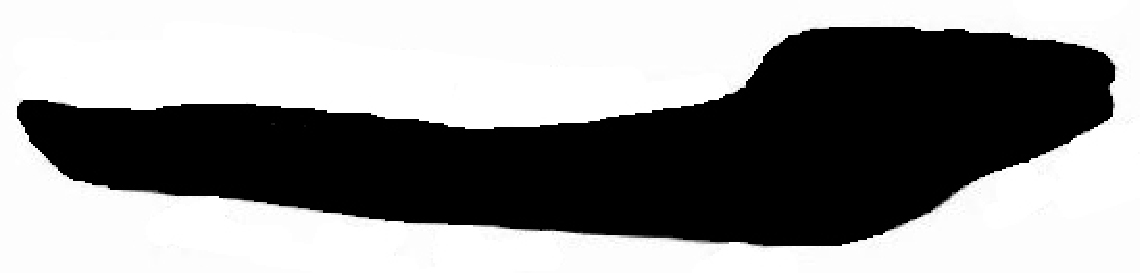

Supplement: Supplemental Information 6 [file peerj-13-20243-s006.zip › SUPPLEMENTARY FILE 7 Code_R2/Code shape lateral/Silhouette_lateral/Phonerpeton_pricei.jpg]

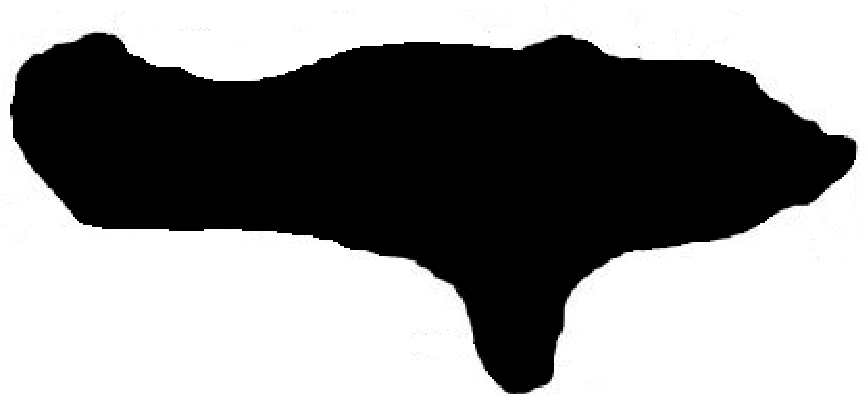

Supplement: Supplemental Information 6 [file peerj-13-20243-s006.zip › SUPPLEMENTARY FILE 7 Code_R2/Code shape lateral/Silhouette_lateral/Nochelesaurus_alexanderi.jpg]

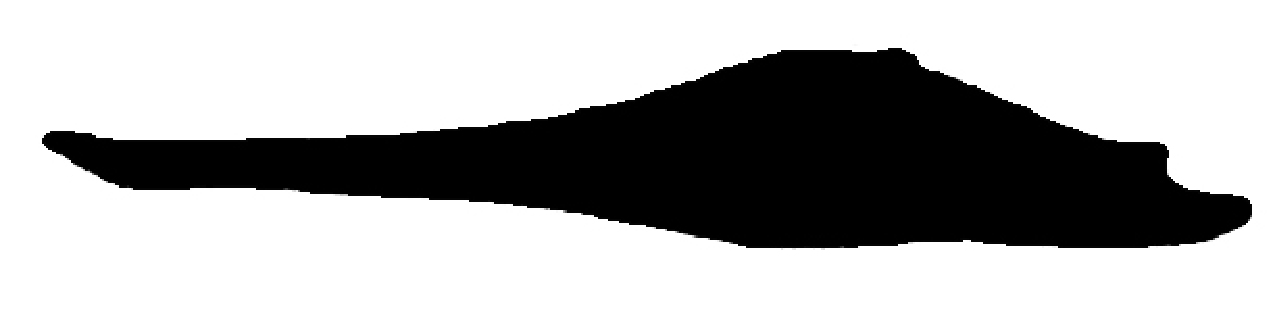

Supplement: Supplemental Information 6 [file peerj-13-20243-s006.zip › SUPPLEMENTARY FILE 7 Code_R2/Code shape lateral/Silhouette_lateral/Milleropsis_pricei.jpg]

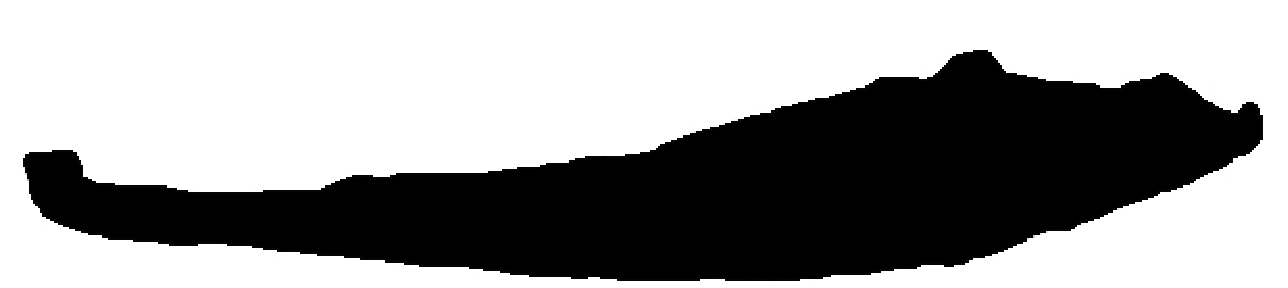

Supplement: Supplemental Information 6 [file peerj-13-20243-s006.zip › SUPPLEMENTARY FILE 7 Code_R2/Code shape lateral/Silhouette_lateral/Laosuchus_hun.jpg]

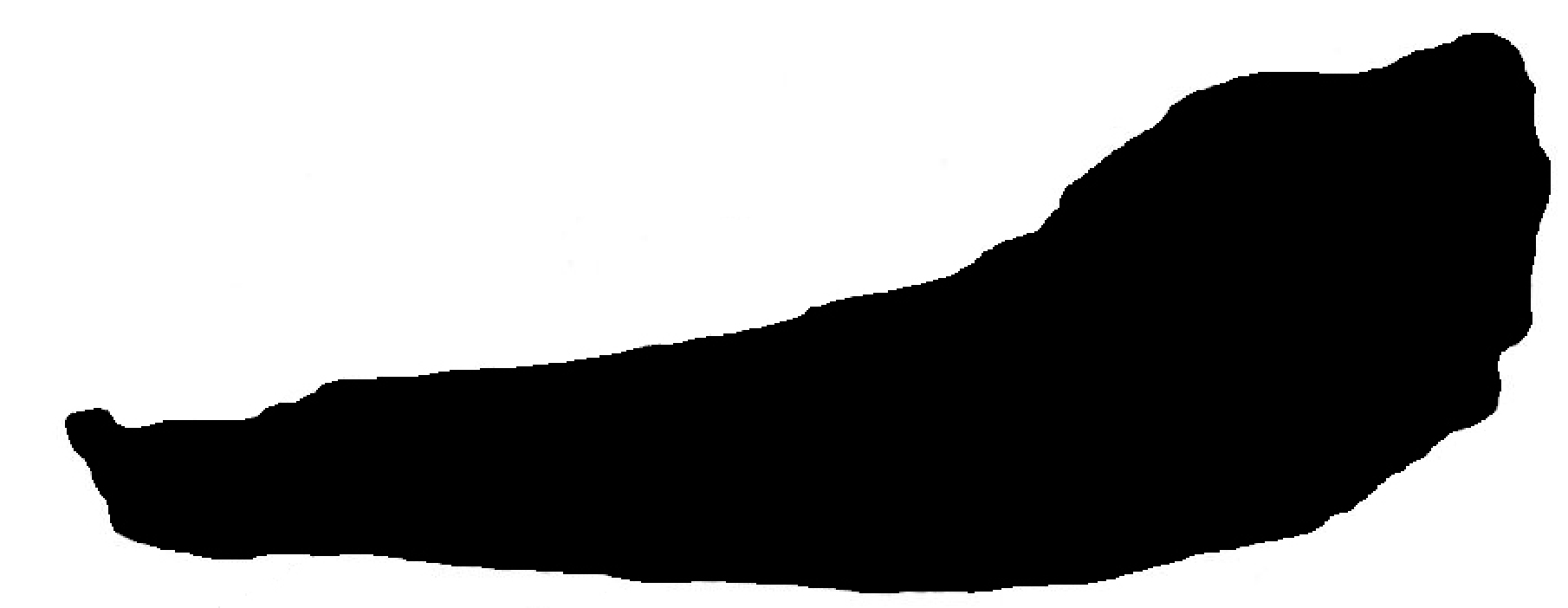

Supplement: Supplemental Information 6 [file peerj-13-20243-s006.zip › SUPPLEMENTARY FILE 7 Code_R2/Code shape lateral/Silhouette_lateral/Pointedward_jaw.jpg]

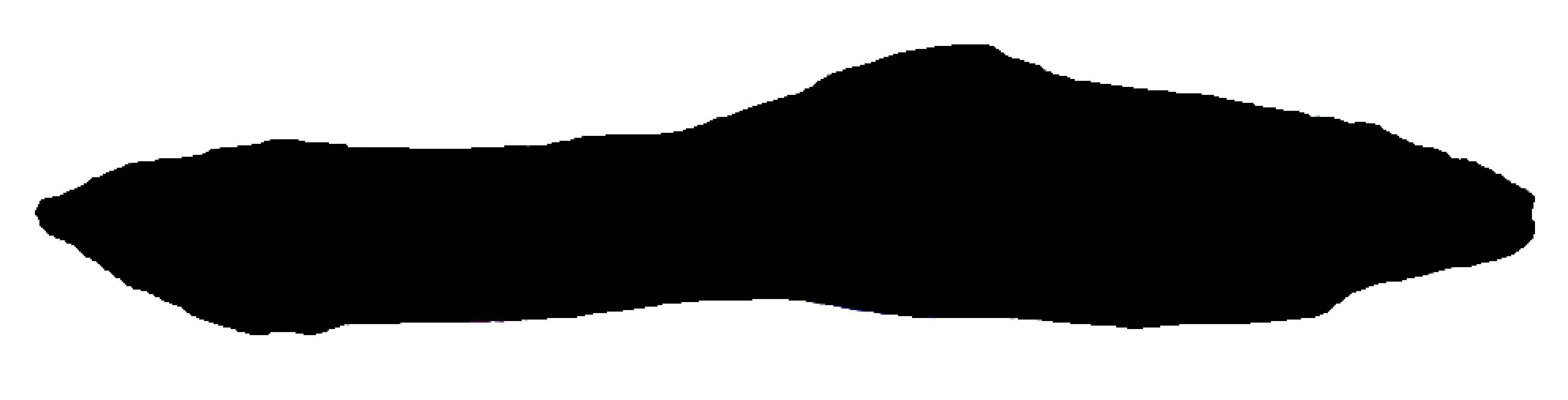

Supplement: Supplemental Information 6 [file peerj-13-20243-s006.zip › SUPPLEMENTARY FILE 7 Code_R2/Code shape lateral/Silhouette_lateral/Orobates_pabsti.jpg]

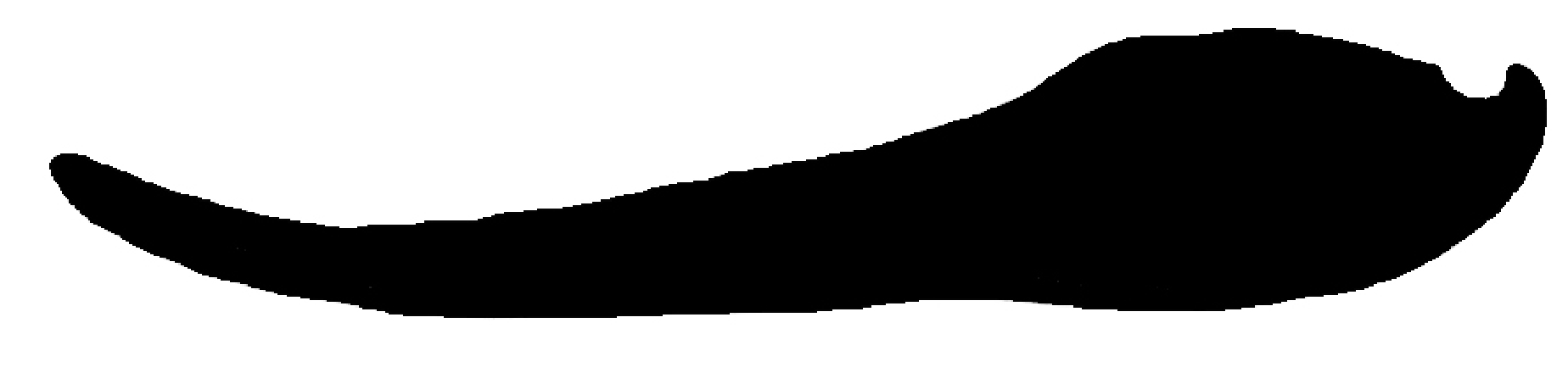

Supplement: Supplemental Information 6 [file peerj-13-20243-s006.zip › SUPPLEMENTARY FILE 7 Code_R2/Code shape lateral/Silhouette_lateral/Ophiacodon_uniformis.jpg]

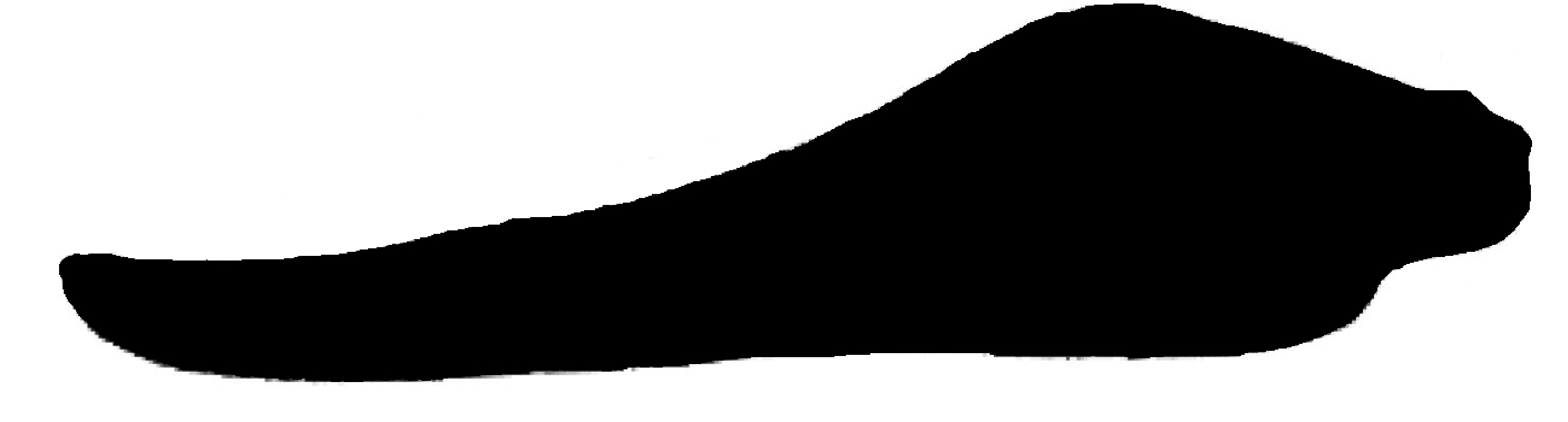

Supplement: Supplemental Information 6 [file peerj-13-20243-s006.zip › SUPPLEMENTARY FILE 7 Code_R2/Code shape lateral/Silhouette_lateral/Palaeohatteria_longicaudata.jpg]

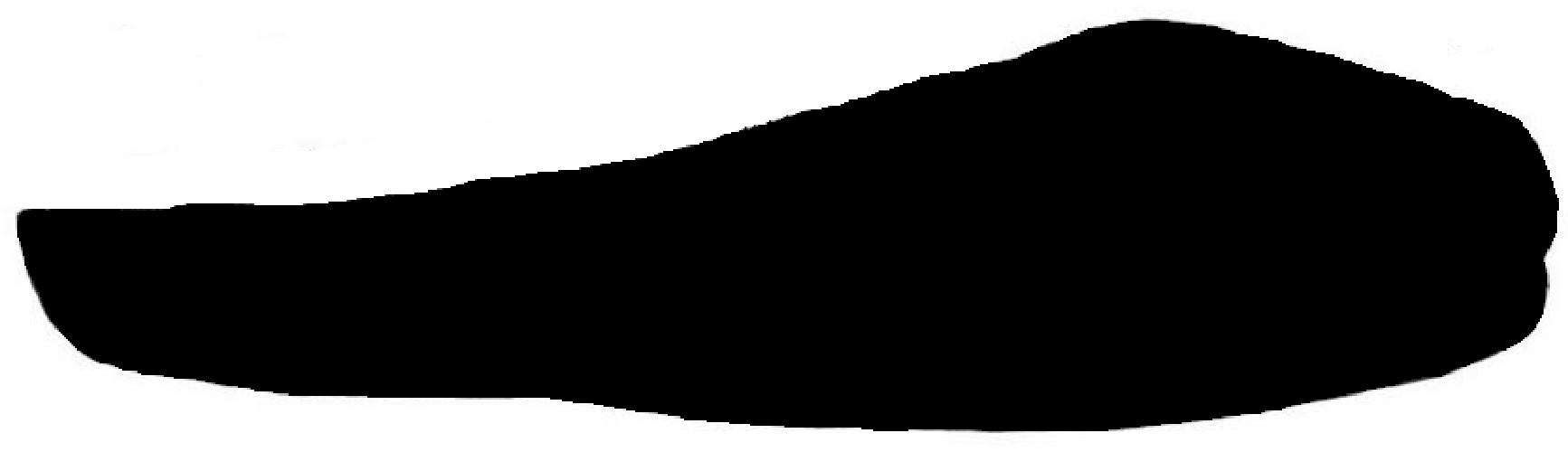

Supplement: Supplemental Information 6 [file peerj-13-20243-s006.zip › SUPPLEMENTARY FILE 7 Code_R2/Code shape lateral/Silhouette_lateral/Makowskia_laticephala.jpg]

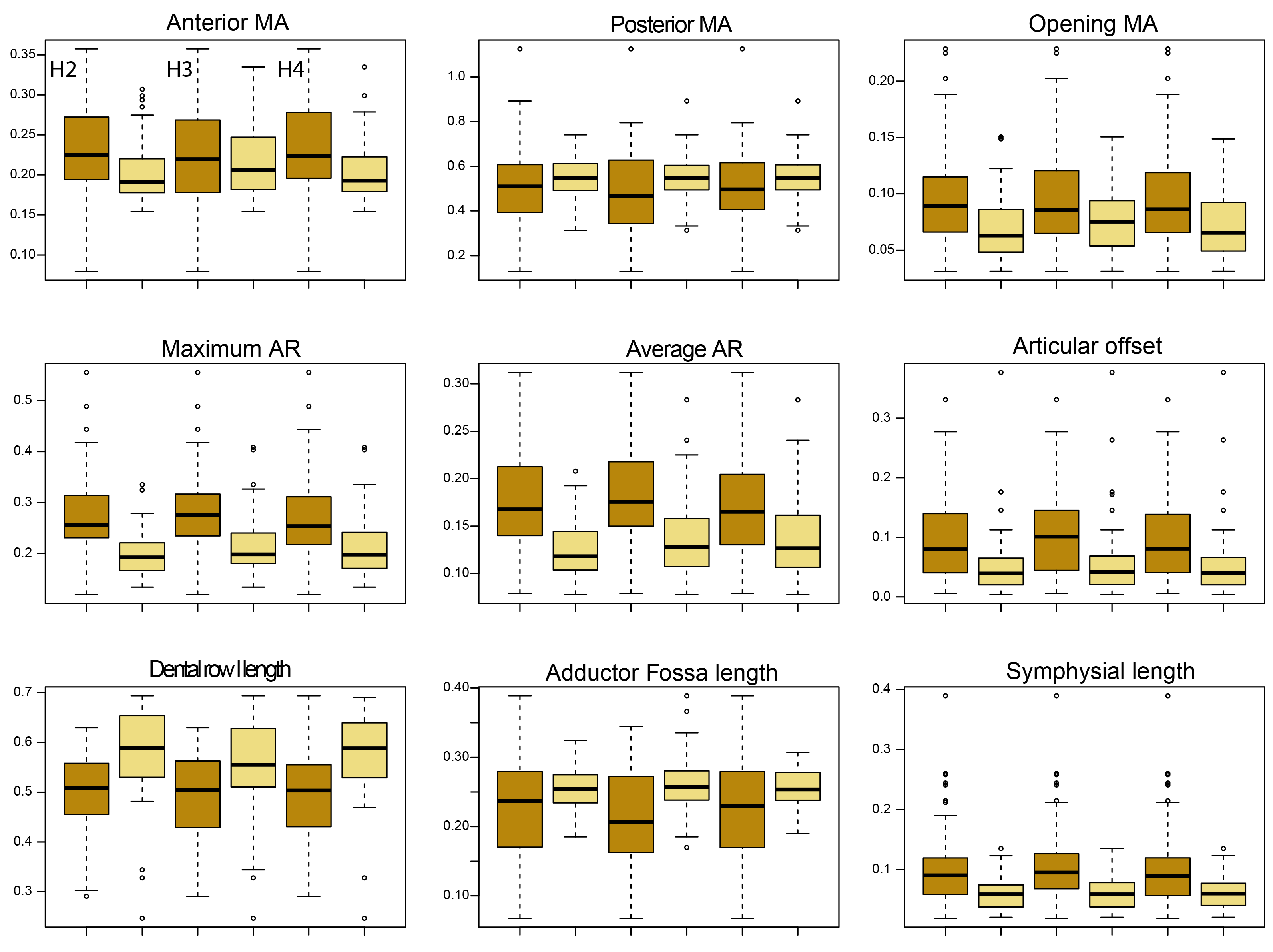

Supplement: Supplemental Information 7 — Comparing biomechanical traits between amniotes (darkgoldenrod) and non-amniote (lightgoldenrod) tetrapods under phylogenetic hypothesis 2 (H2; Diadectomorpha are synapsid amniotes), hypothesis 3 (H3; Captorhinidae and Araeoscelidia are non-amniote tetrapods) and hypothesis 4 (H4; Recumbirostra are sauropsid amniotes). [file peerj-13-20243-s007.png]

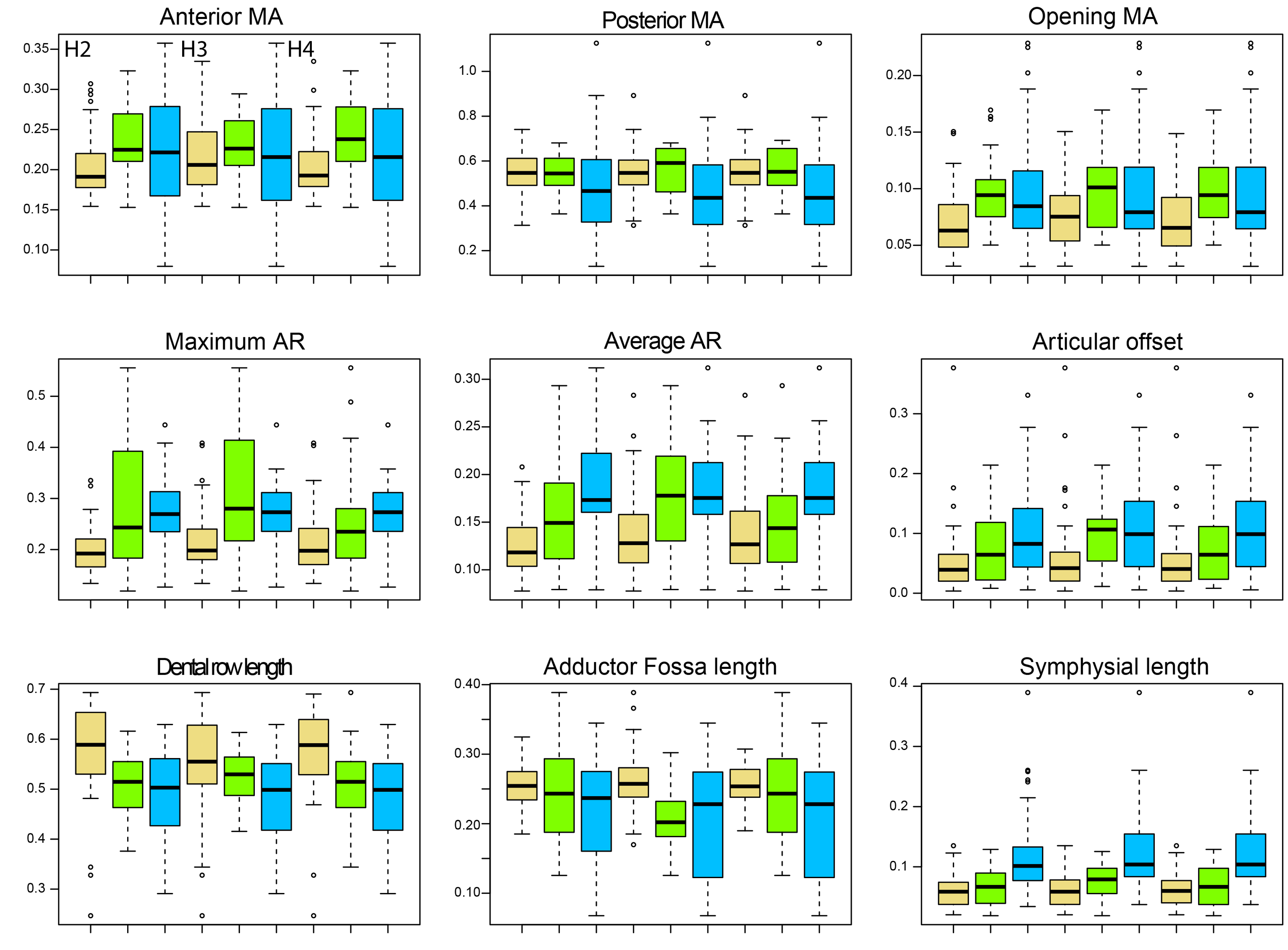

Supplement: Supplemental Information 8 — Comparing biomechanical traits between non-amniote tetrapods (lightgoldenrod), sauropsids (green) and synapsids (blue) under phylogenetic hypothesis 2 (H2; Diadectomorpha are synapsid amniotes), hypothesis 3 (H3; Captorhinidae and Araeoscelidia are non-amniote tetrapods) and hypothesis 4 (H4; Recumbirostra are sauropsid amniotes). [file peerj-13-20243-s008.png]

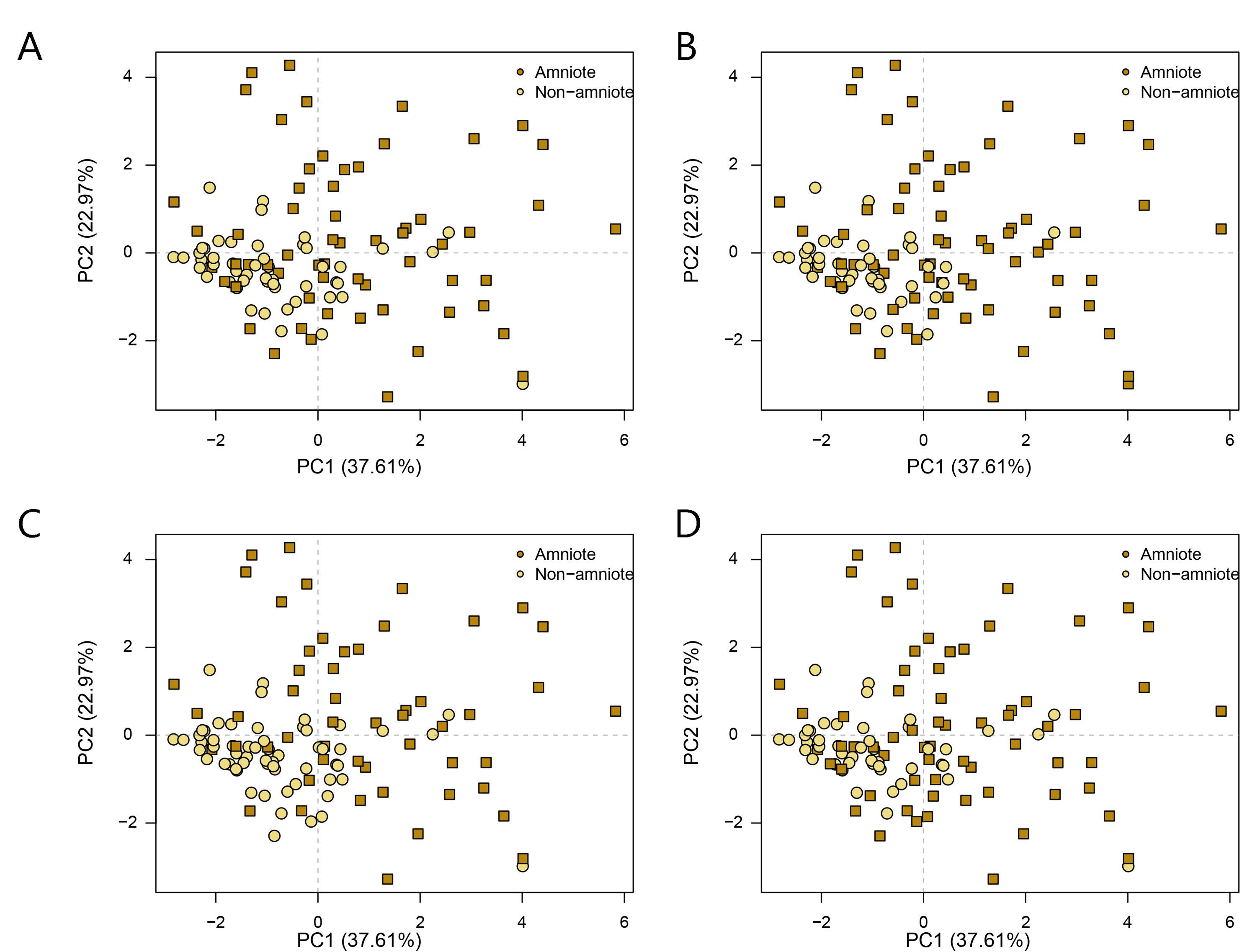

Supplement: Supplemental Information 9 — A = baseline hypothesis; B = hypothesis 2 (Diadectomorpha are synapsid amniotes); C = hypothesis 3 (Captorhinidae and Araeoscelidia are non-amniote tetrapods); D = hypothesis 4 (Recumbirostra are sauropsid amniotes). [file peerj-13-20243-s009.png]

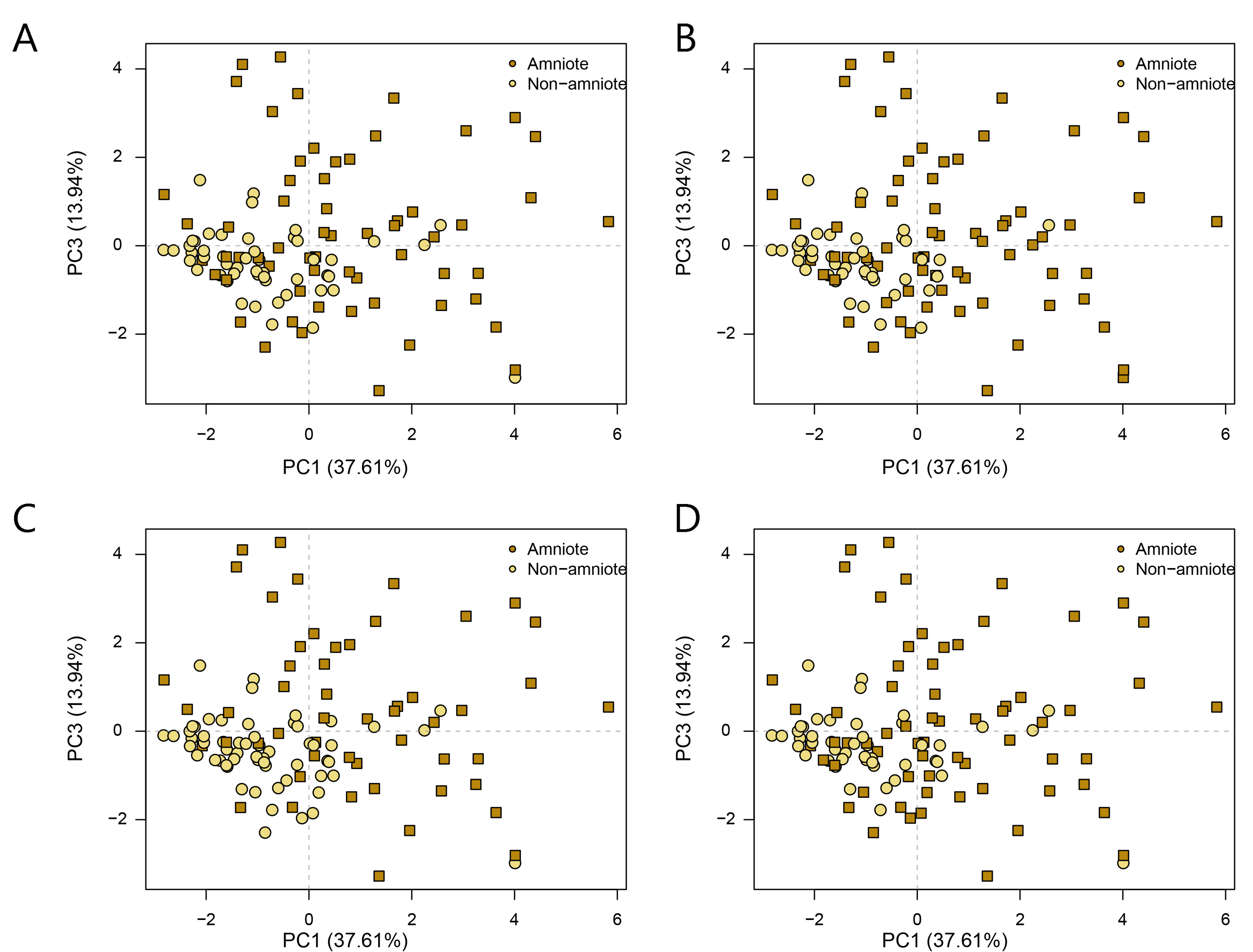

Supplement: Supplemental Information 10 — A = baseline hypothesis; B = hypothesis 2 (Diadectomorpha are synapsid amniotes); C = hypothesis 3 (Captorhinidae and Araeoscelidia are non-amniote tetrapods); D = hypothesis 4 (Recumbirostra are sauropsid amniotes). [file peerj-13-20243-s010.png]

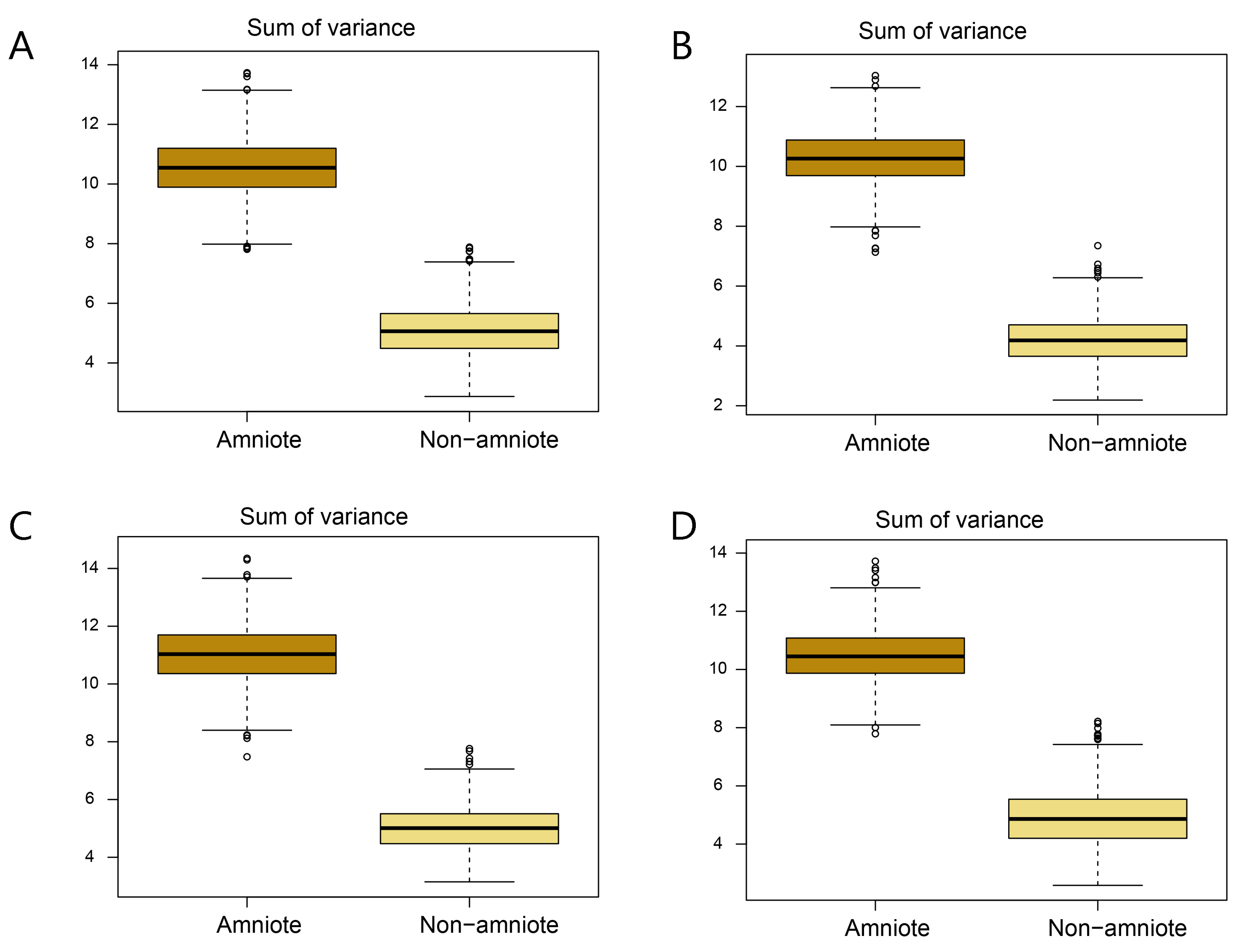

Supplement: Supplemental Information 11 — A = baseline hypothesis; B = hypothesis 2 (Diadectomorpha are synapsid amniotes); C = hypothesis 3 (Captorhinidae and Araeoscelidia are non-amniote tetrapods); D = hypothesis 4 (Recumbirostra are sauropsid amniotes). [file peerj-13-20243-s011.png]

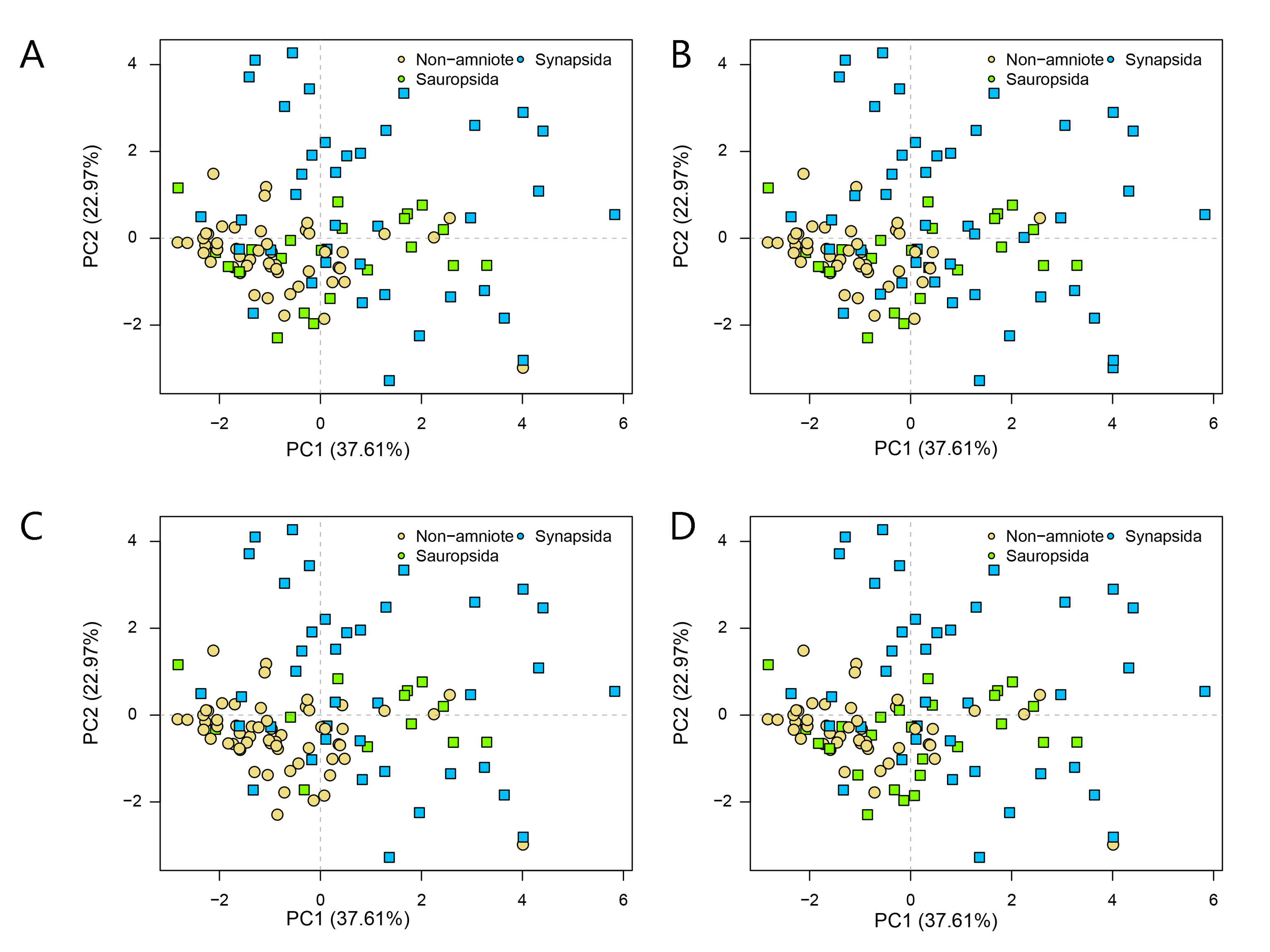

Supplement: Supplemental Information 12 — A = baseline hypothesis; B = hypothesis 2 (Diadectomorpha are synapsid amniotes); C = hypothesis 3 (Captorhinidae and Araeoscelidia are non-amniote tetrapods); D = hypothesis 4 (Recumbirostra are sauropsid amniotes). [file peerj-13-20243-s012.png]

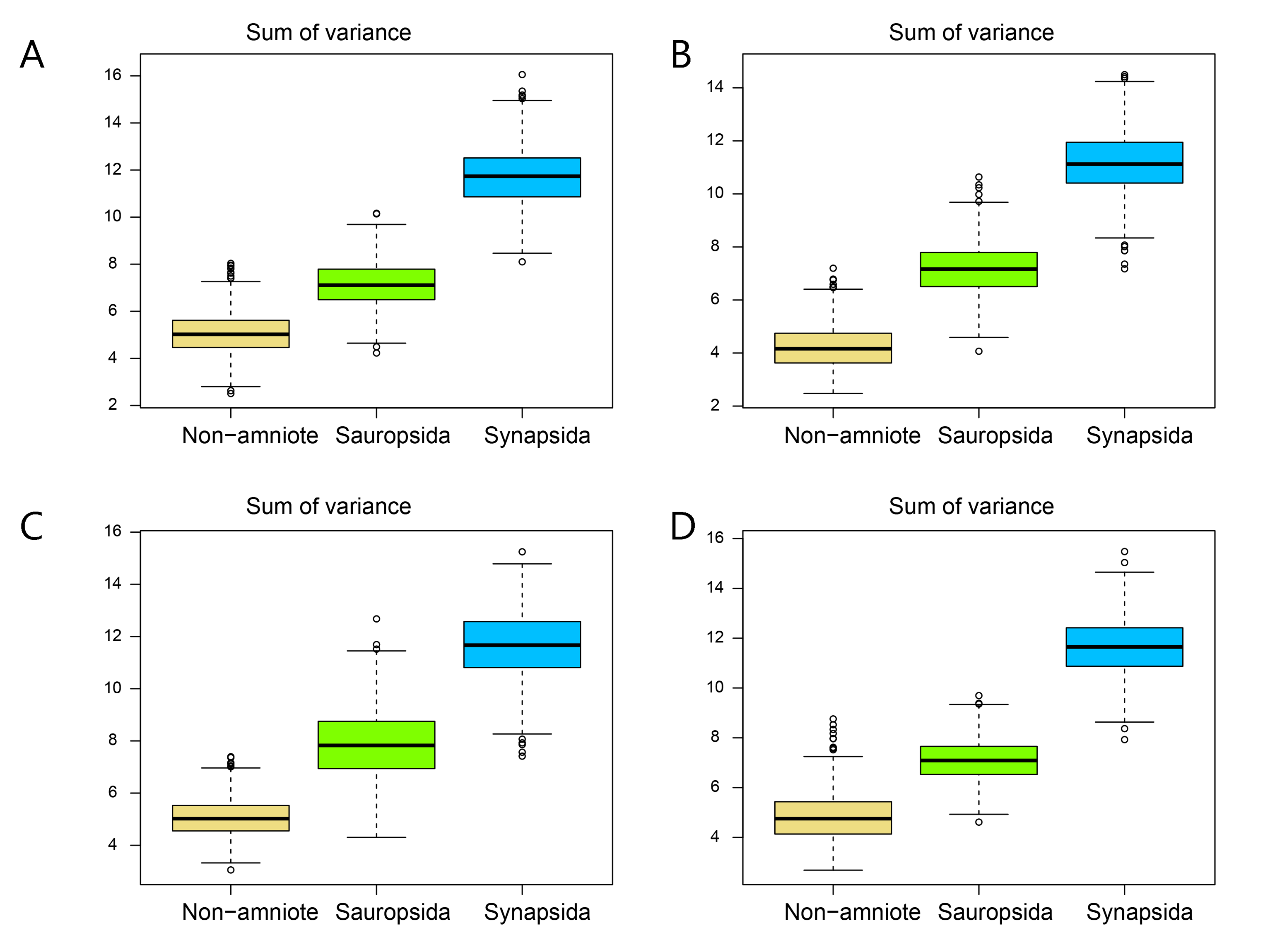

Supplement: Supplemental Information 13 — A = baseline hypothesis; B = hypothesis 2 (Diadectomorpha are synapsid amniotes); C = hypothesis 3 (Captorhinidae and Araeoscelidia are non-amniote tetrapods); D = hypothesis 4 (Recumbirostra are sauropsid amniotes). [file peerj-13-20243-s013.png]

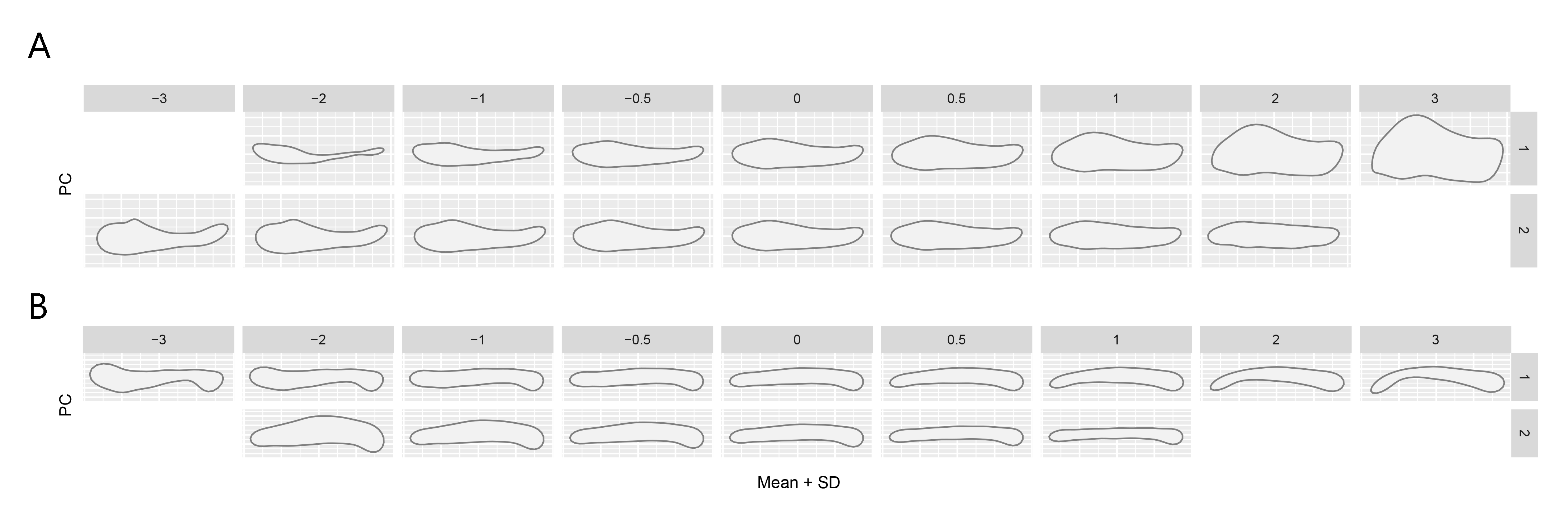

Supplement: Supplemental Information 14 — A lateral shape morphospace and B occlusal shape morphospace [file peerj-13-20243-s014.png]

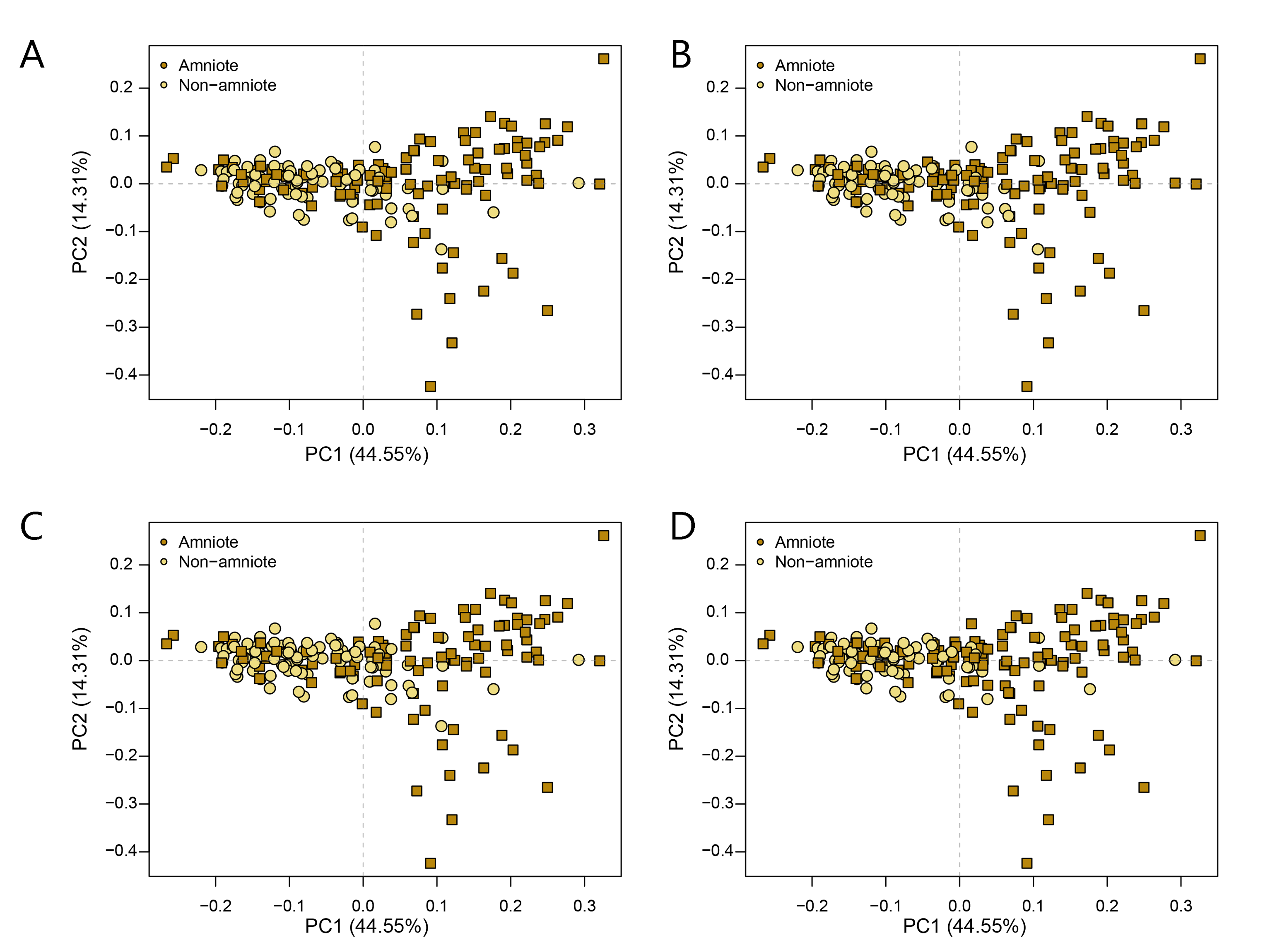

Supplement: Supplemental Information 15 — A = baseline hypothesis; B = hypothesis 2 (Diadectomorpha are synapsid amniotes); C = hypothesis 3 (Captorhinidae and Araeoscelidia are non-amniote tetrapods); D = hypothesis 4 (Recumbirostra are sauropsid amniotes). [file peerj-13-20243-s015.png]

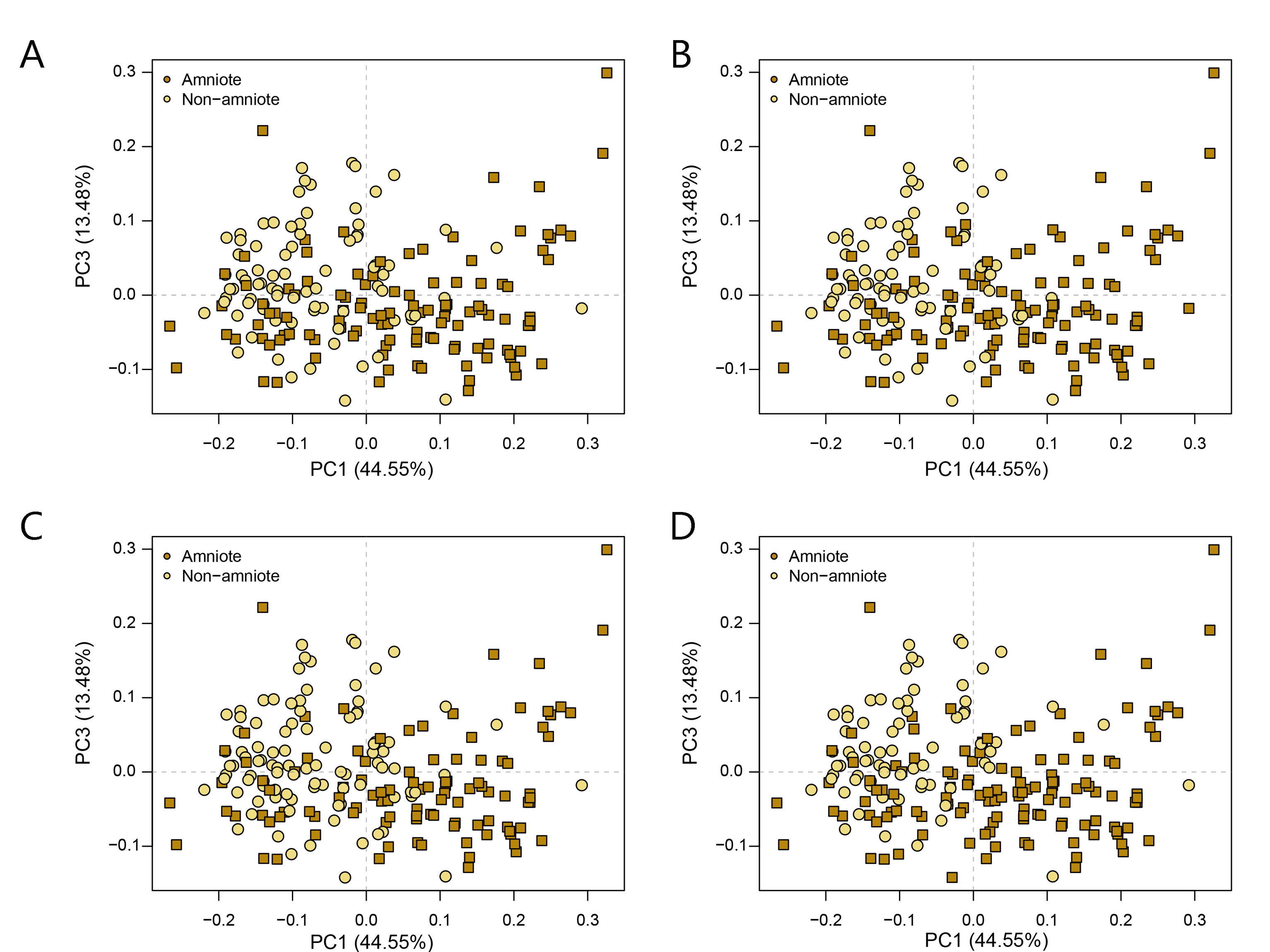

Supplement: Supplemental Information 16 — A = baseline hypothesis; B = hypothesis 2 (Diadectomorpha are synapsid amniotes); C = hypothesis 3 (Captorhinidae and Araeoscelidia are non-amniote tetrapods); D = hypothesis 4 (Recumbirostra are sauropsid amniotes). [file peerj-13-20243-s016.png]

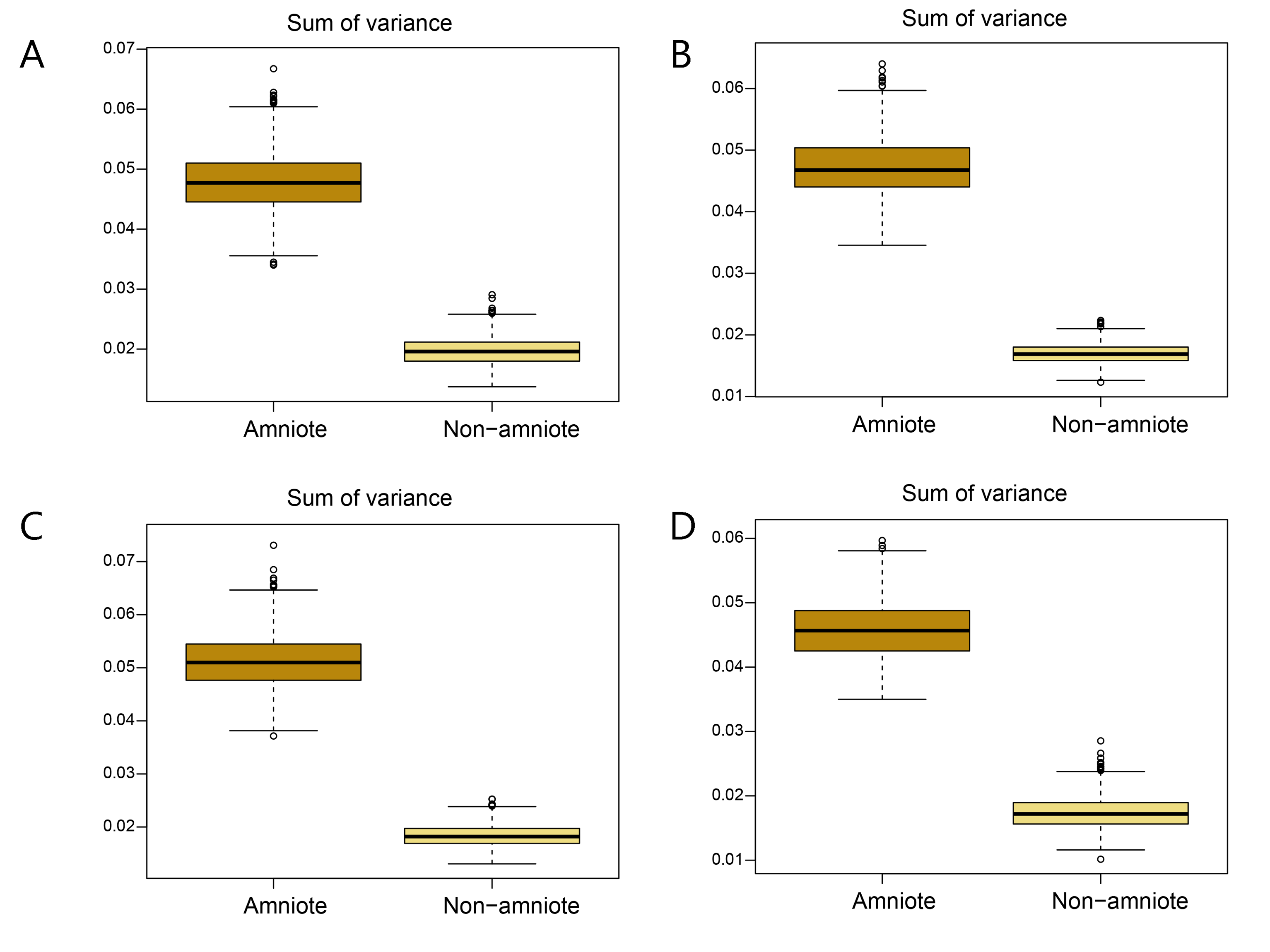

Supplement: Supplemental Information 17 — A = baseline hypothesis; B = hypothesis 2 (Diadectomorpha are synapsid amniotes); C = hypothesis 3 (Captorhinidae and Araeoscelidia are non-amniote tetrapods); D = hypothesis 4 (Recumbirostra are sauropsid amniotes). [file peerj-13-20243-s017.png]

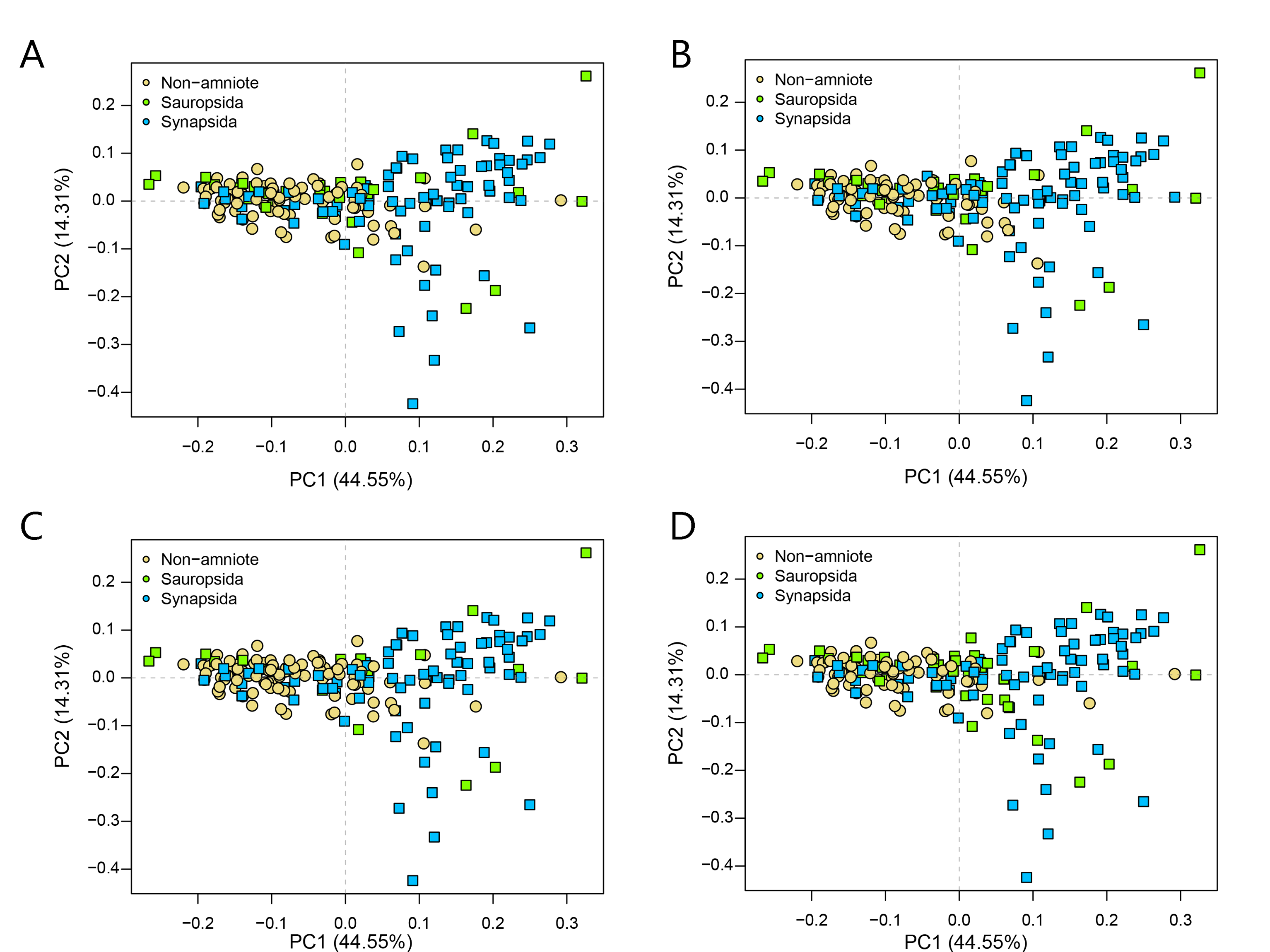

Supplement: Supplemental Information 18 — A = baseline hypothesis; B = hypothesis 2 (Diadectomorpha are synapsid amniotes); C = hypothesis 3 (Captorhinidae and Araeoscelidia are non-amniote tetrapods); D = hypothesis 4 (Recumbirostra are sauropsid amniotes). [file peerj-13-20243-s018.png]

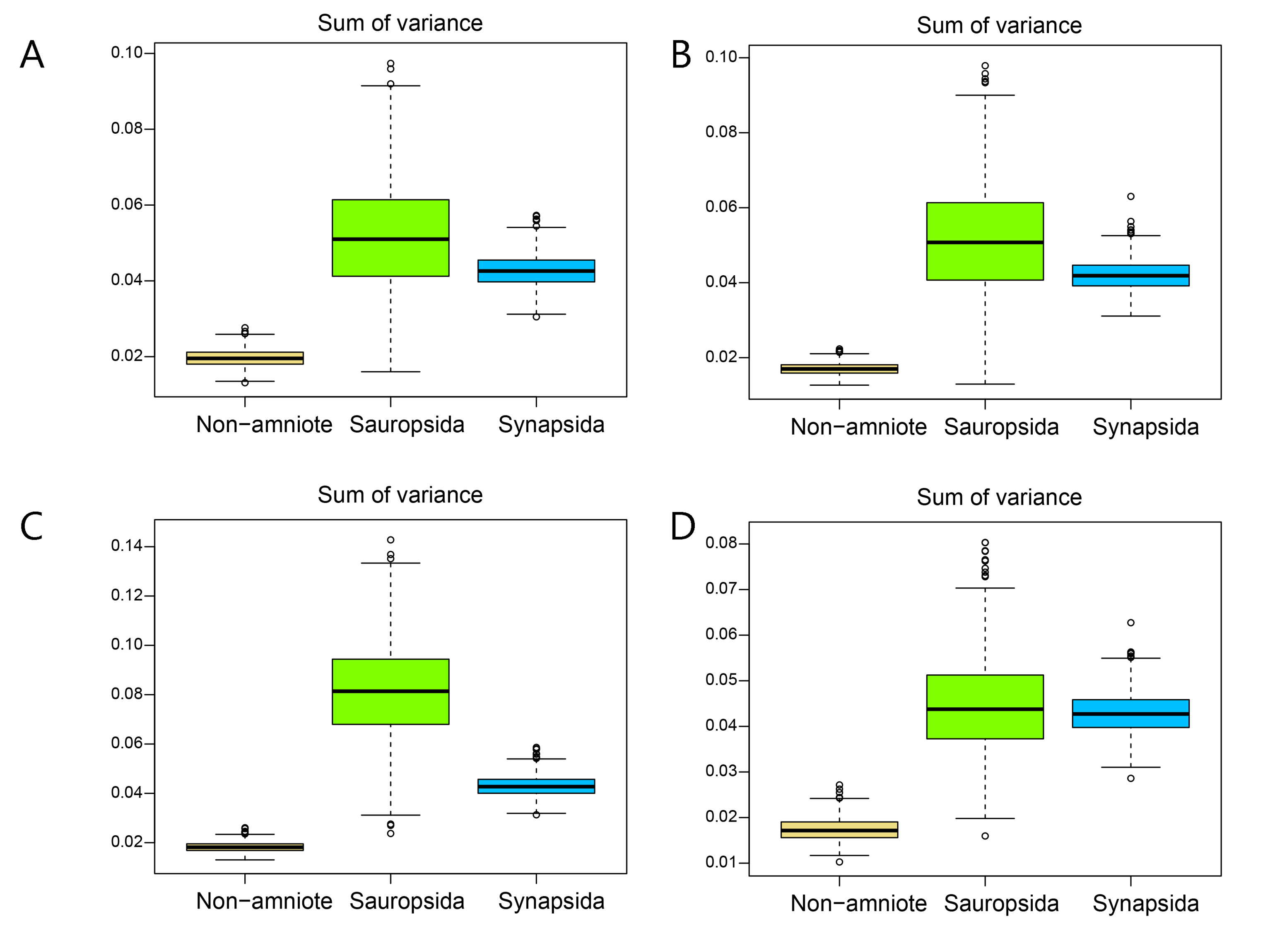

Supplement: Supplemental Information 19 — A = baseline hypothesis; B = hypothesis 2 (Diadectomorpha are synapsid amniotes); C = hypothesis 3 (Captorhinidae and Araeoscelidia are non-amniote tetrapods); D = hypothesis 4 (Recumbirostra are sauropsid amniotes). [file peerj-13-20243-s019.png]

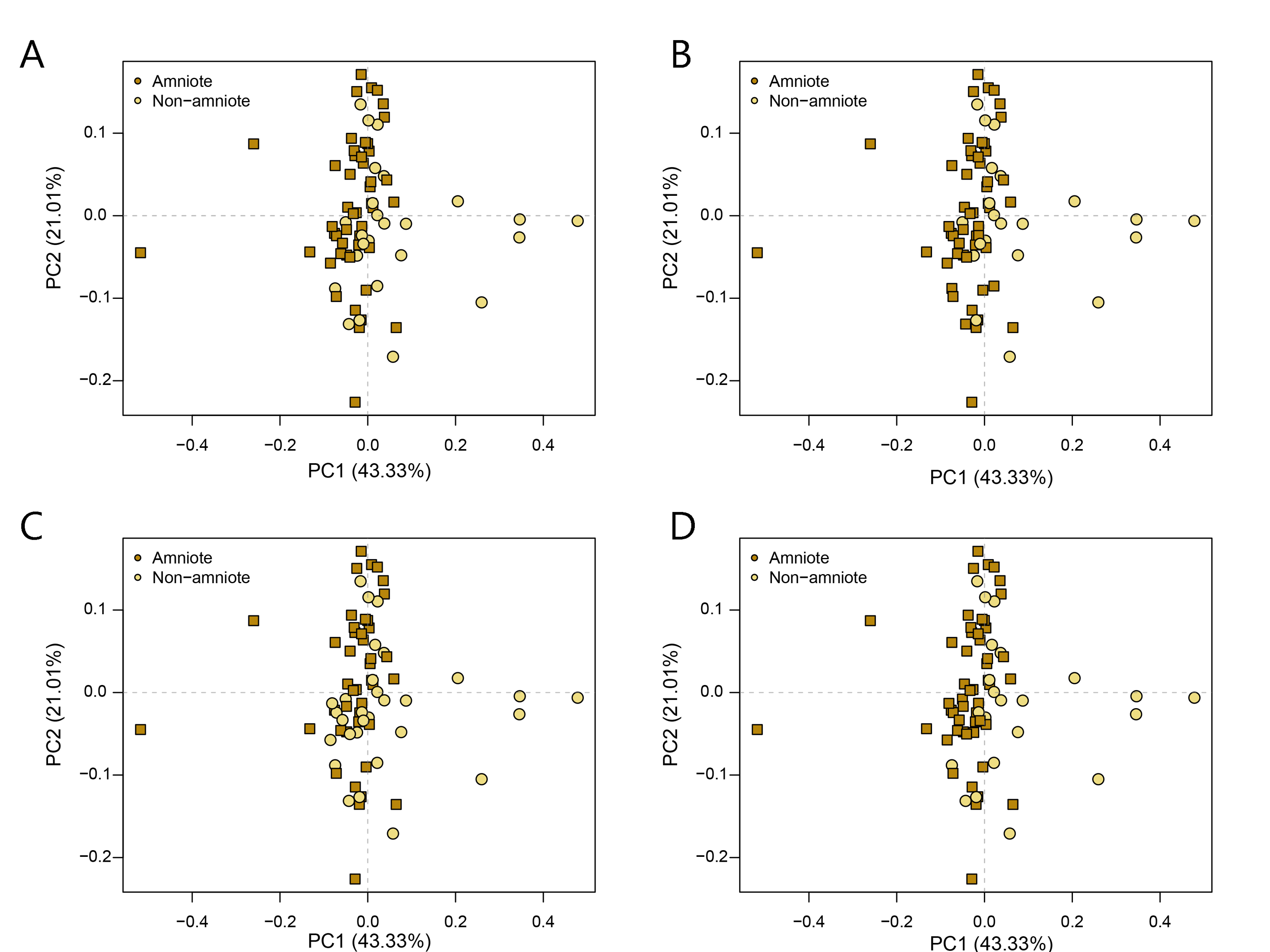

Supplement: Supplemental Information 20 — A = baseline hypothesis; B = hypothesis 2 (Diadectomorpha are synapsid amniotes); C = hypothesis 3 (Captorhinidae and Araeoscelidia are non-amniote tetrapods); D = hypothesis 4 (Recumbirostra are sauropsid amniotes). [file peerj-13-20243-s020.png]

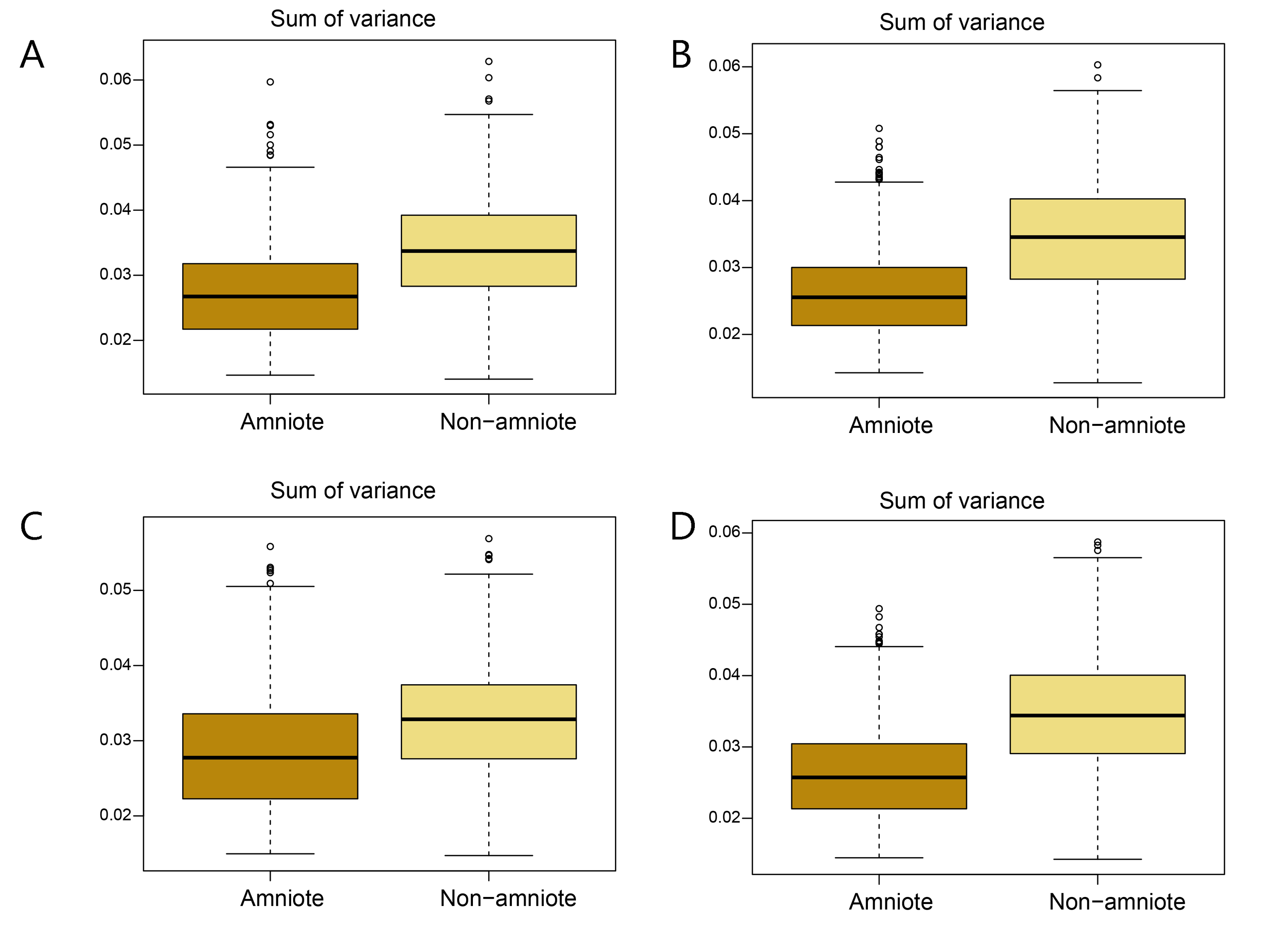

Supplement: Supplemental Information 21 — A = baseline hypothesis; B = hypothesis 2 (Diadectomorpha are synapsid amniotes); C = hypothesis 3 (Captorhinidae and Araeoscelidia are non-amniote tetrapods); D = hypothesis 4 (Recumbirostra are sauropsid amniotes). [file peerj-13-20243-s021.png]

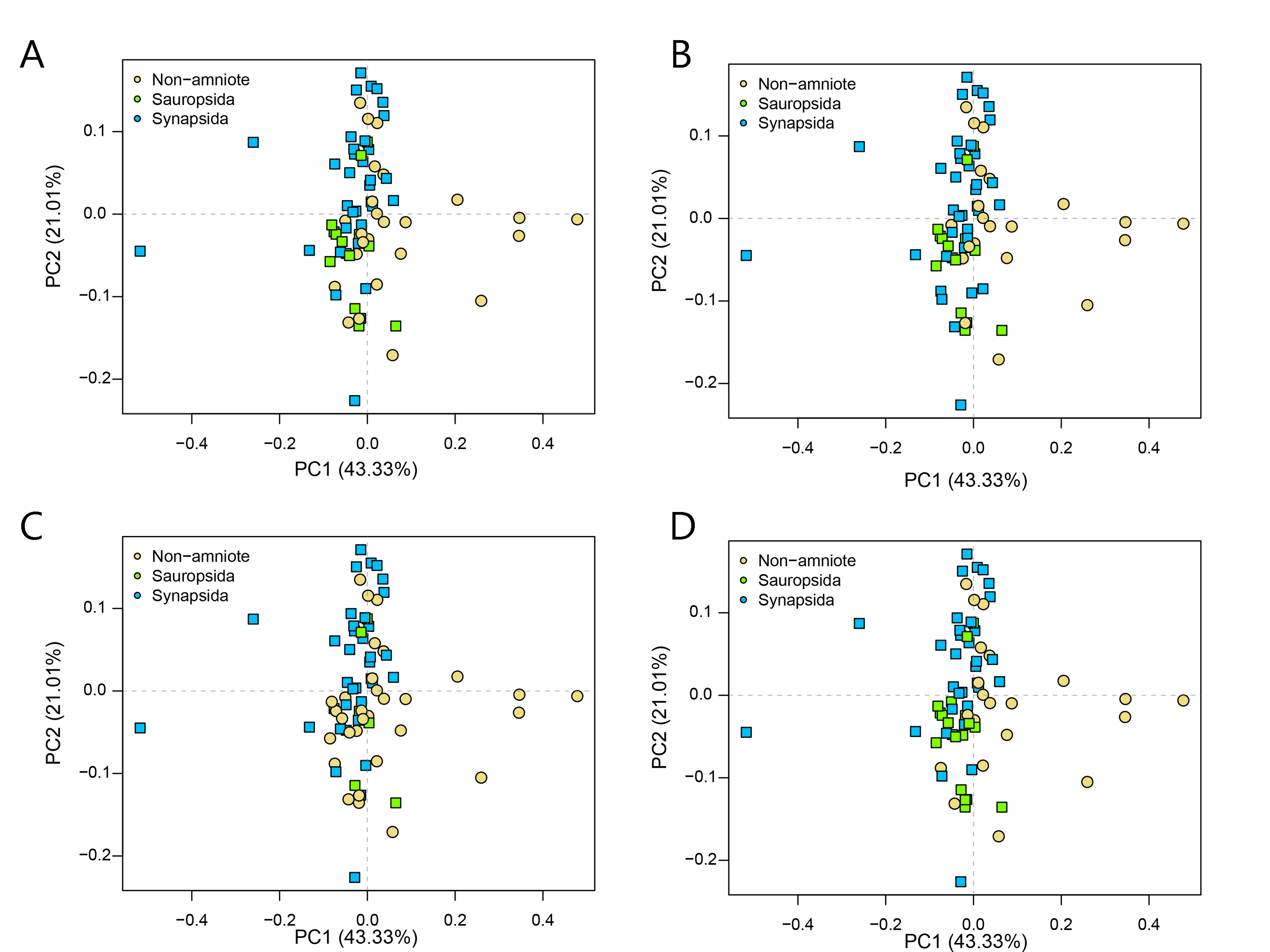

Supplement: Supplemental Information 22 — A = baseline hypothesis; B = hypothesis 2 (Diadectomorpha are synapsid amniotes); C = hypothesis 3 (Captorhinidae and Araeoscelidia are non-amniote tetrapods); D = hypothesis 4 (Recumbirostra are sauropsid amniotes). [file peerj-13-20243-s022.png]

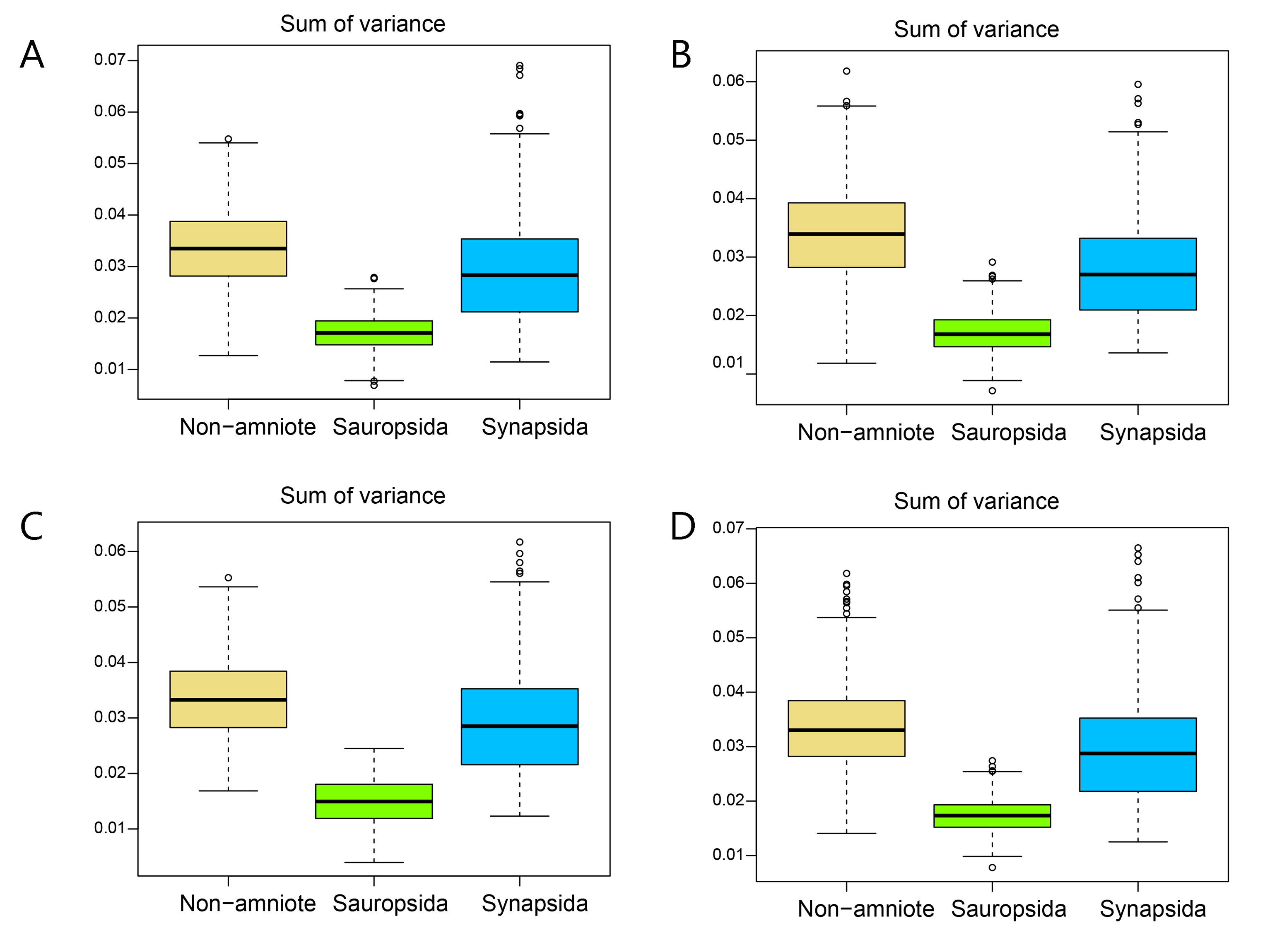

Supplement: Supplemental Information 23 — A = baseline hypothesis; B = hypothesis 2 (Diadectomorpha are synapsid amniotes); C = hypothesis 3 (Captorhinidae and Araeoscelidia are non-amniote tetrapods); D = hypothesis 4 (Recumbirostra are sauropsid amniotes). [file peerj-13-20243-s023.png]

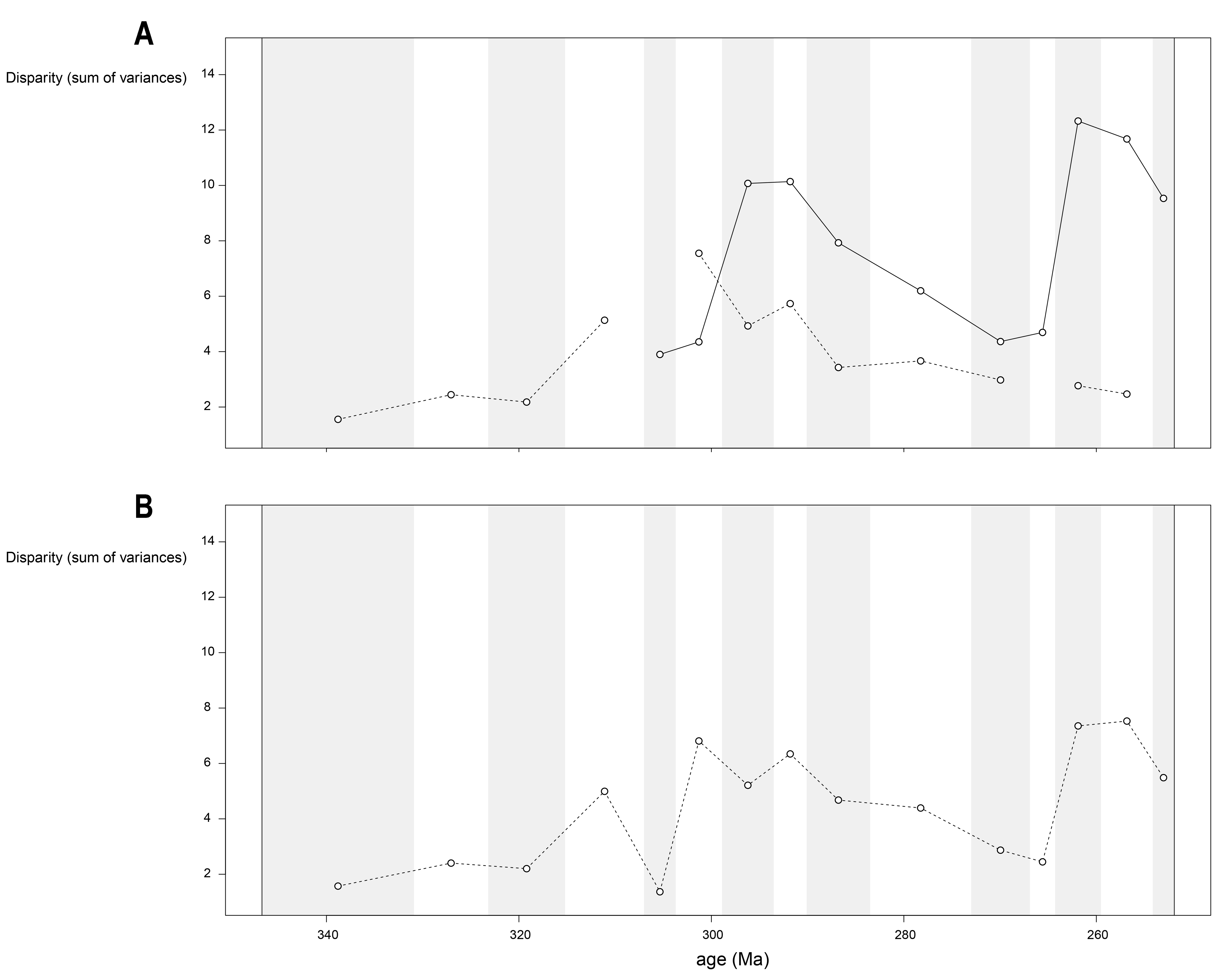

Supplement: Supplemental Information 24 — A amniotes (fair) versus non-amniote tetrapods (dashed line) and B carnivorous tetrapods (dashed line) through time. [file peerj-13-20243-s024.png]

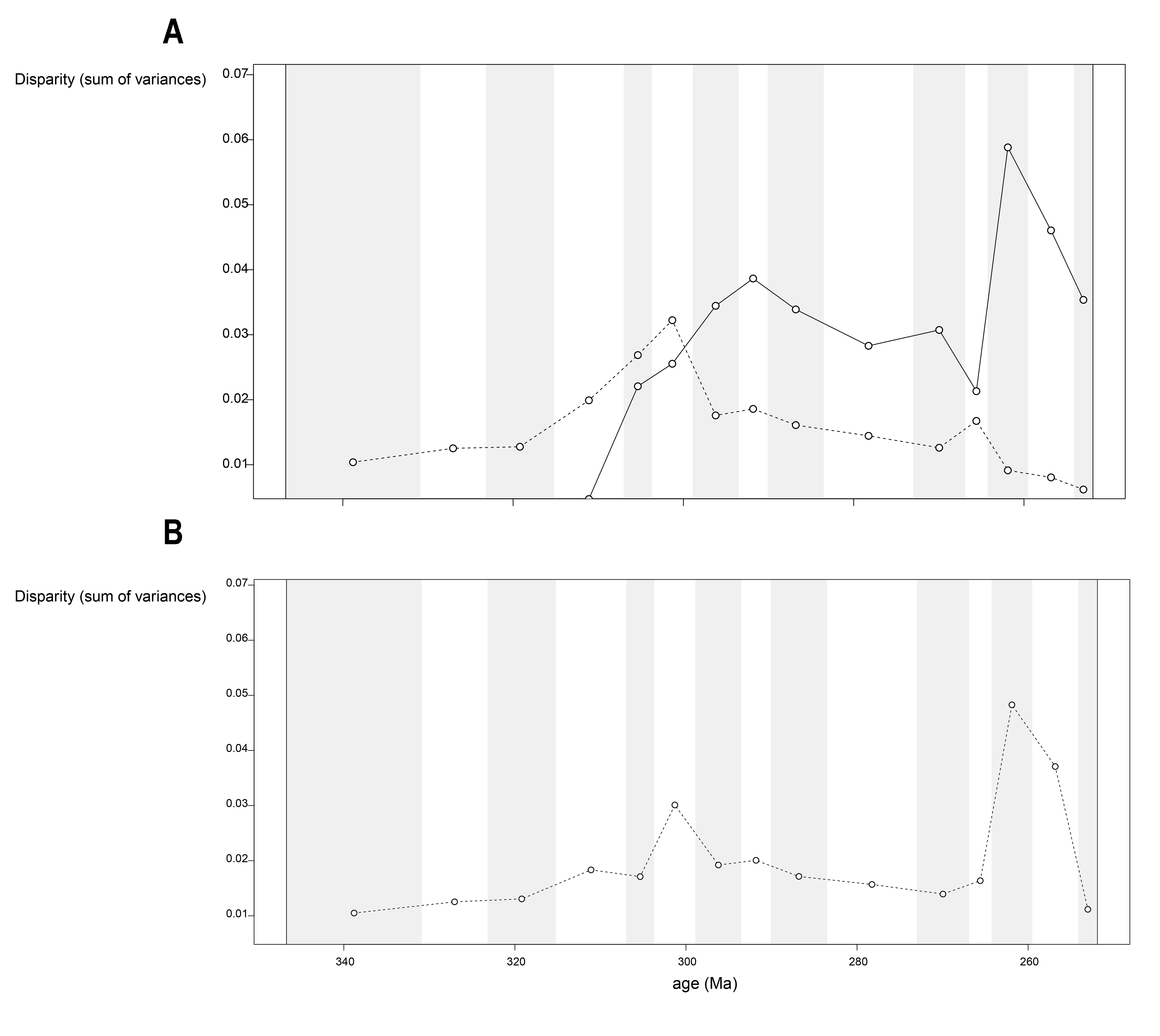

Supplement: Supplemental Information 25 — A amniotes (fair) versus non-amniote tetrapods (dashed line) and B carnivorous tetrapods (dashed line) through time. [file peerj-13-20243-s025.png]

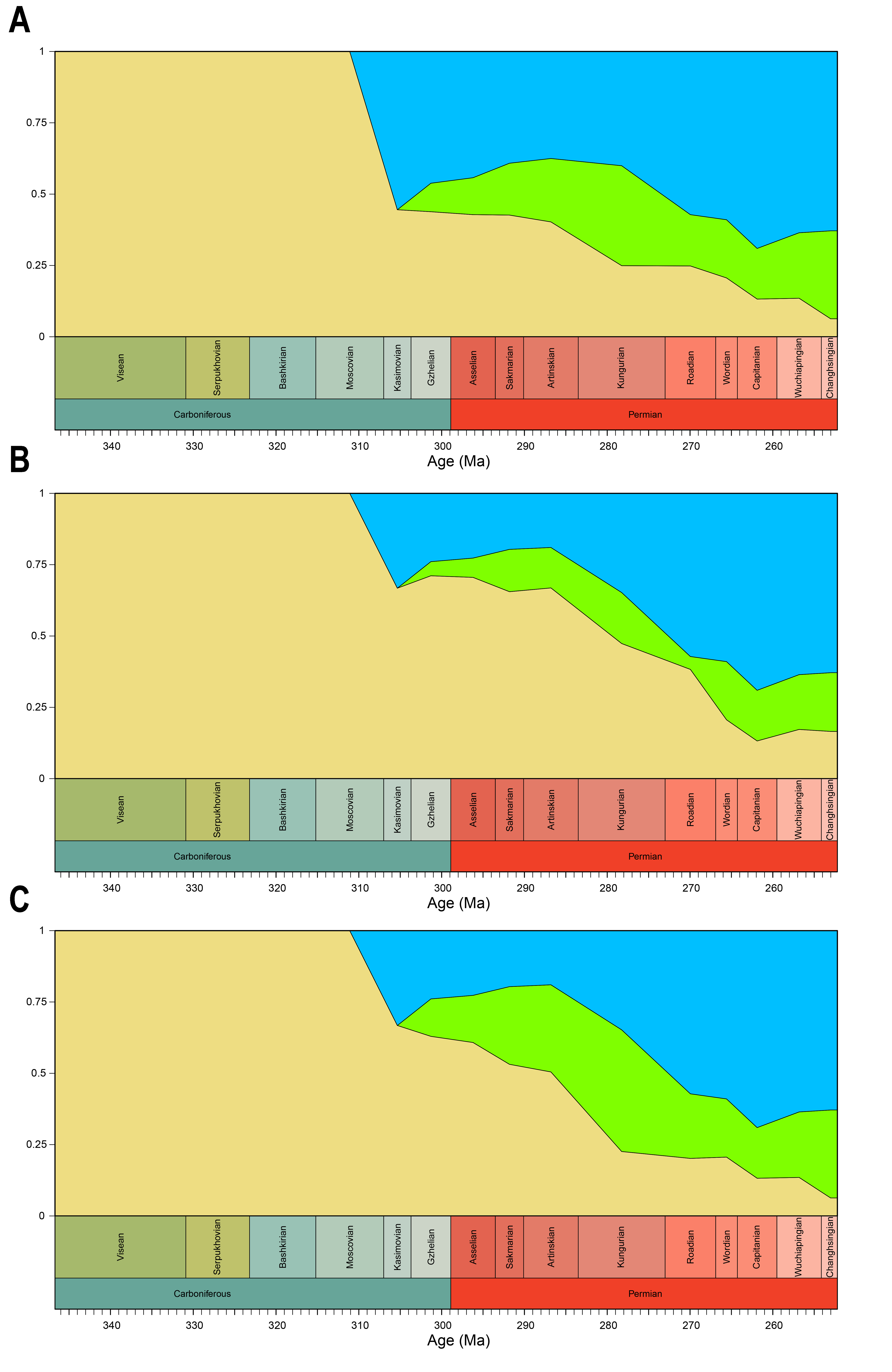

Supplement: Supplemental Information 26 — A = hypothesis 2 (Diadectomorpha are synapsid amniotes); B = hypothesis 3 (Captorhinidae and Araeoscelidia are non-amniote tetrapods); C = hypothesis 4 (Recumbirostra are sauropsid amniotes). Lightgoldenrod = non-amniote tetrapods, green = Sauropsida, blue = Synapsida. [file peerj-13-20243-s026.png]

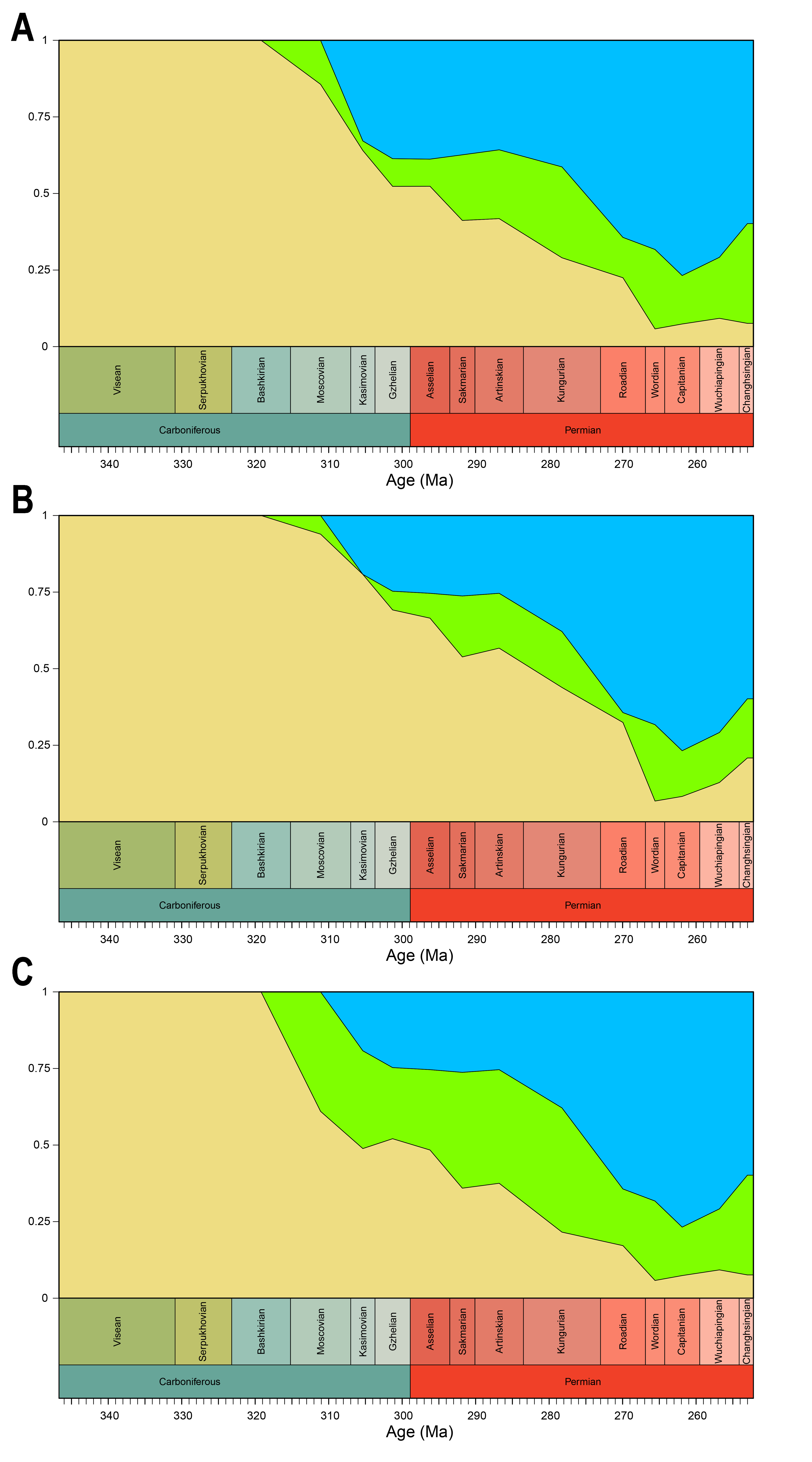

Supplement: Supplemental Information 27 — A = hypothesis 2 (Diadectomorpha are synapsid amniotes); B = hypothesis 3 (Captorhinidae and Araeoscelidia are non-amniote tetrapods); C = hypothesis 4 (Recumbirostra are sauropsid amniotes). Lightgoldenrod = non-amniote tetrapods, green = Sauropsida, blue = Synapsida. [file peerj-13-20243-s027.png]

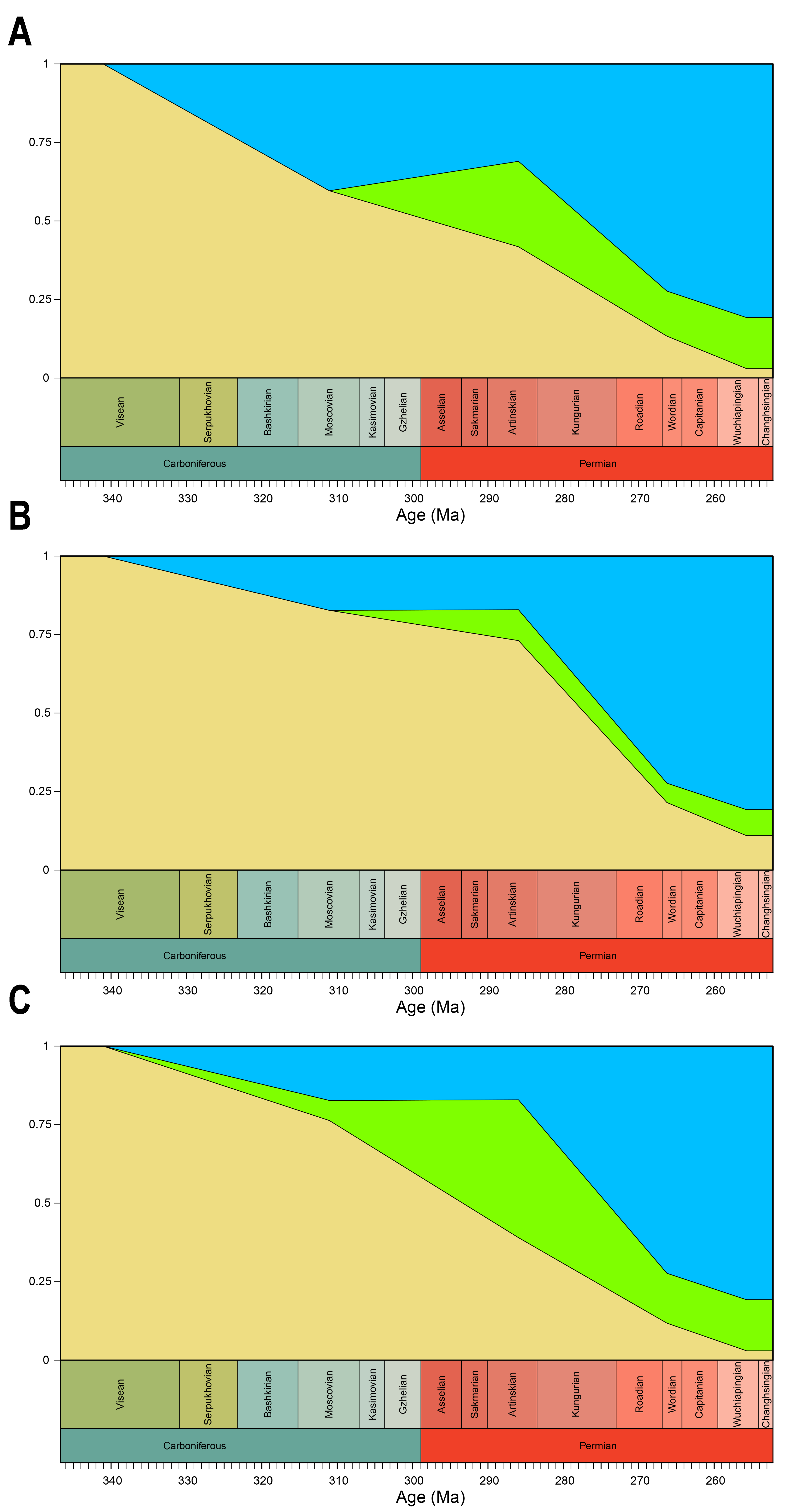

Supplement: Supplemental Information 28 — A = hypothesis 2 (Diadectomorpha are synapsid amniotes); B = hypothesis 3 (Captorhinidae and Araeoscelidia are non-amniote tetrapods); C = hypothesis 4 (Recumbirostra are sauropsid amniotes). Lightgoldenrod = non-amniote tetrapods, green = Sauropsida, blue = Synapsida. [file peerj-13-20243-s028.png]

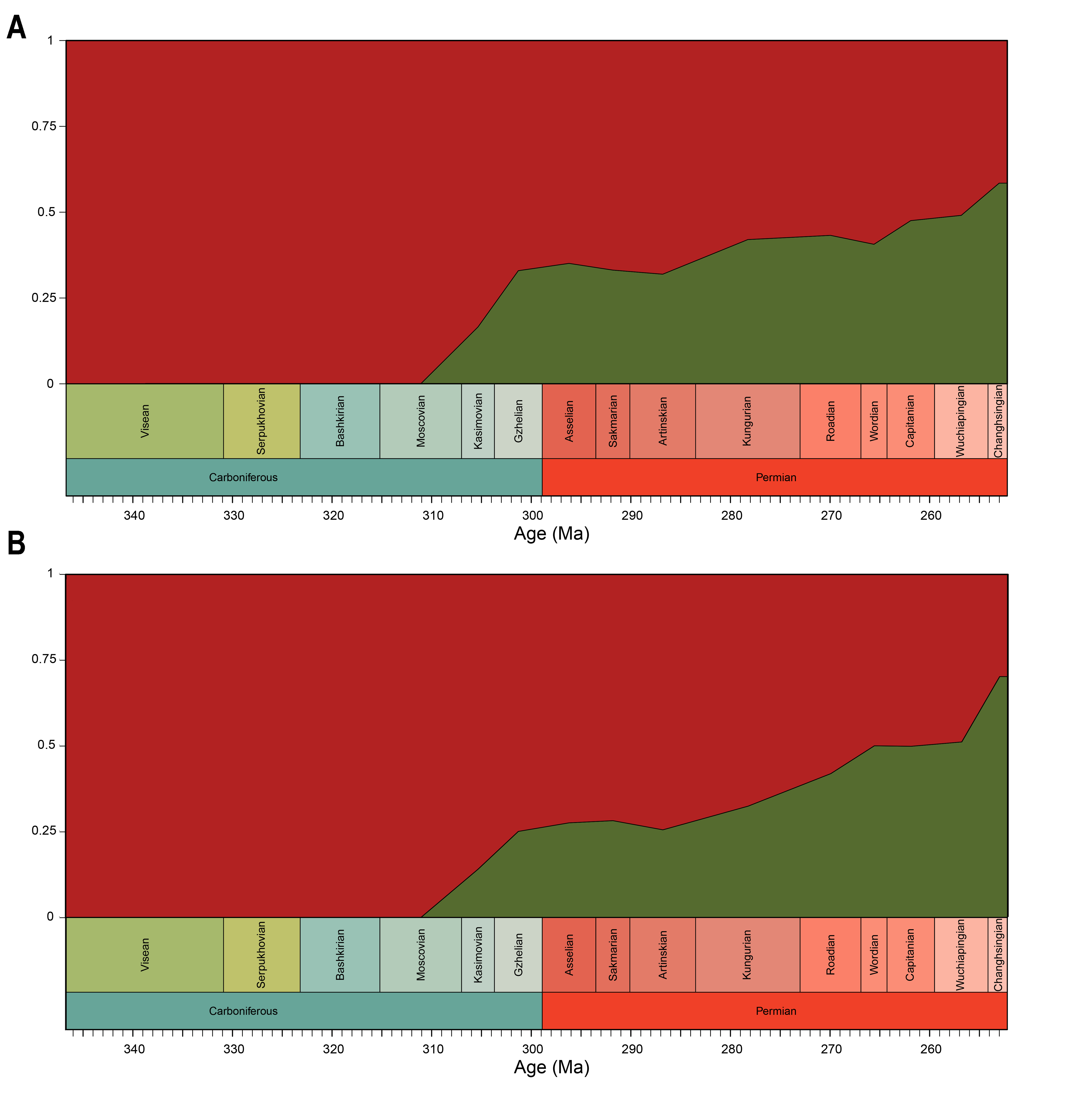

Supplement: Supplemental Information 29 — carnivores (firebrick) versus herbivores (darkolivegreen) in terms of A jaw biomechanics and B lateral shape. [file peerj-13-20243-s029.png]
