# Supplementary figures and images for: Mandibular form and function is more disparate in amniotes than in non-amniote tetrapods from the late Palaeozoic (part 1 of 2)
Source: PeerJ. 2025 Nov 26;13:e20243. doi: 10.7717/peerj.20243 (PMC12664332; doi:10.7717/peerj.20243)

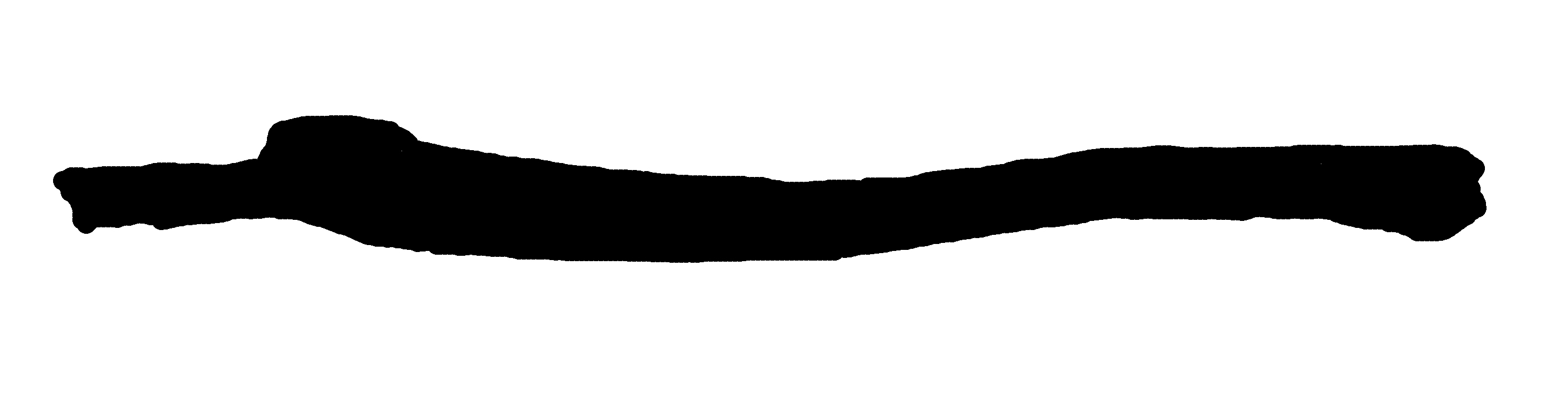

Supplement: Supplemental Information 6 [file peerj-13-20243-s006.zip › SUPPLEMENTARY FILE 7 Code_R2/Code shape occlusal/Silhouette_occlusal/Lycideops_longiceps.jpg]

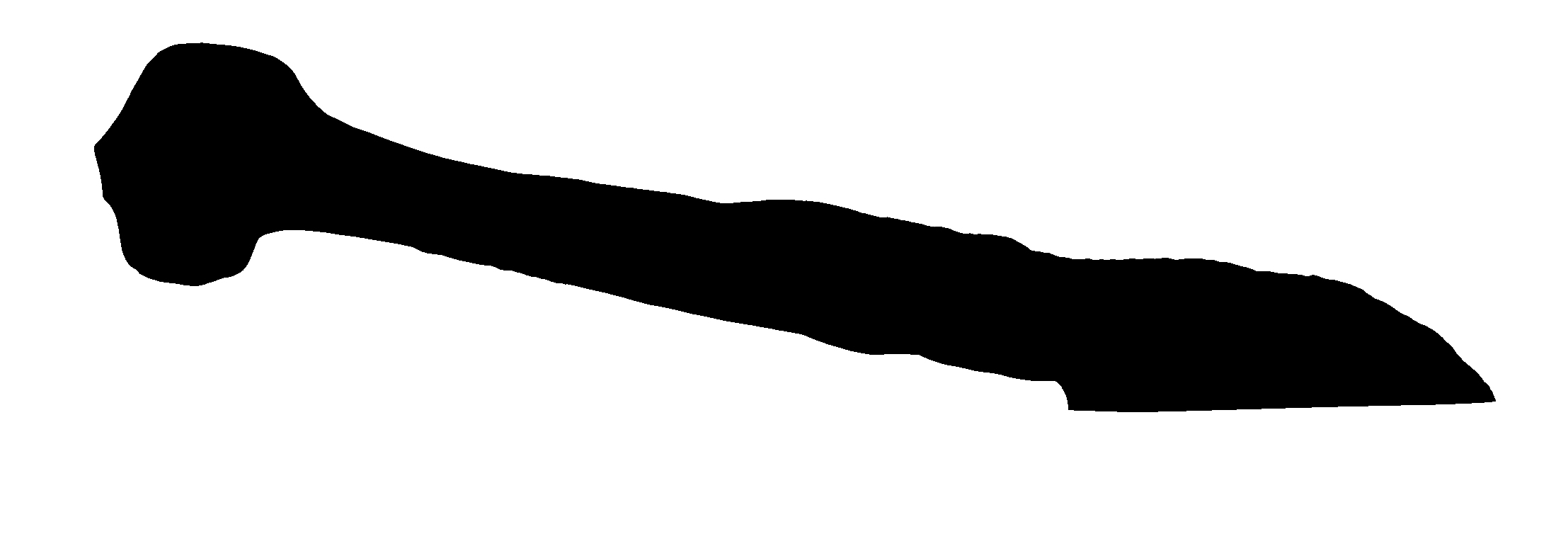

Supplement: Supplemental Information 6 [file peerj-13-20243-s006.zip › SUPPLEMENTARY FILE 7 Code_R2/Code shape occlusal/Silhouette_occlusal/Odontocyclops_whaitsi.jpg]

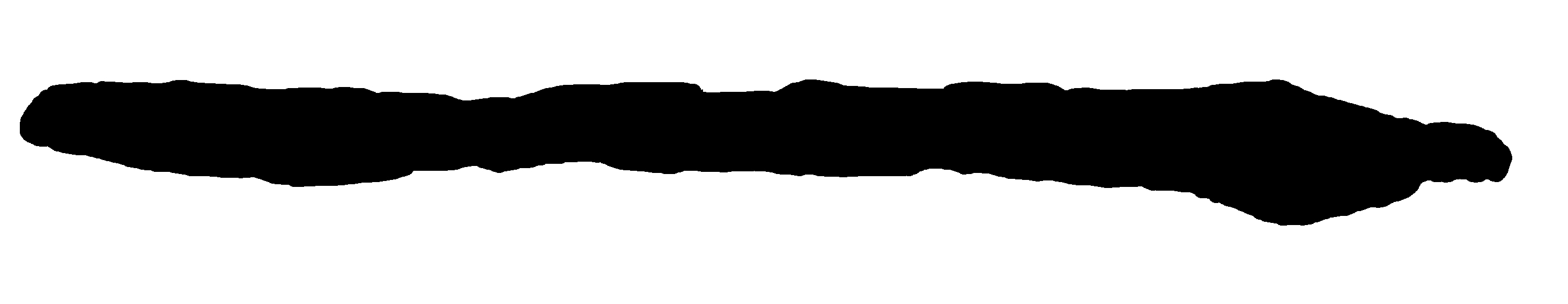

Supplement: Supplemental Information 6 [file peerj-13-20243-s006.zip › SUPPLEMENTARY FILE 7 Code_R2/Code shape occlusal/Silhouette_occlusal/Scylacosaurus_sclateri.jpg]

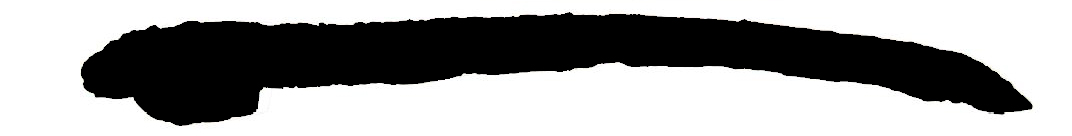

Supplement: Supplemental Information 6 [file peerj-13-20243-s006.zip › SUPPLEMENTARY FILE 7 Code_R2/Code shape occlusal/Silhouette_occlusal/Rastosuchus_hammeri.jpg]

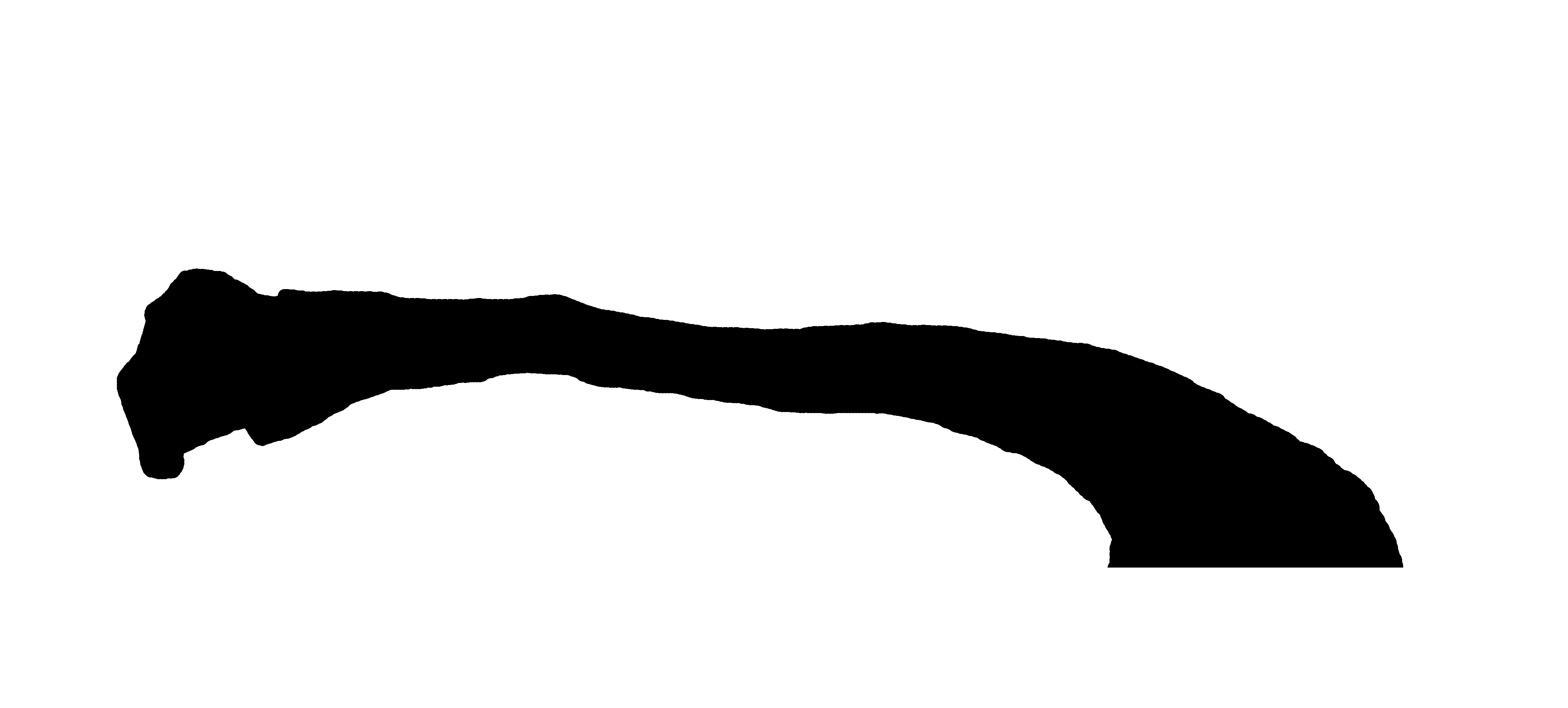

Supplement: Supplemental Information 6 [file peerj-13-20243-s006.zip › SUPPLEMENTARY FILE 7 Code_R2/Code shape occlusal/Silhouette_occlusal/Tapinocaninus_pamelae.jpg]

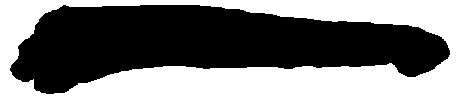

Supplement: Supplemental Information 6 [file peerj-13-20243-s006.zip › SUPPLEMENTARY FILE 7 Code_R2/Code shape occlusal/Silhouette_occlusal/Euryodus_dalyae.jpg]

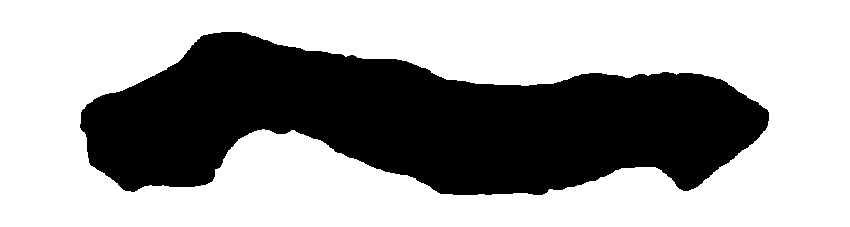

Supplement: Supplemental Information 6 [file peerj-13-20243-s006.zip › SUPPLEMENTARY FILE 7 Code_R2/Code shape occlusal/Silhouette_occlusal/Diadectes_lentus.jpg]

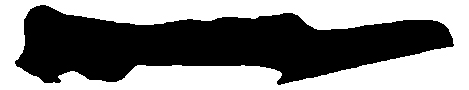

Supplement: Supplemental Information 6 [file peerj-13-20243-s006.zip › SUPPLEMENTARY FILE 7 Code_R2/Code shape occlusal/Silhouette_occlusal/Daptocephalus_leoniceps.jpg]

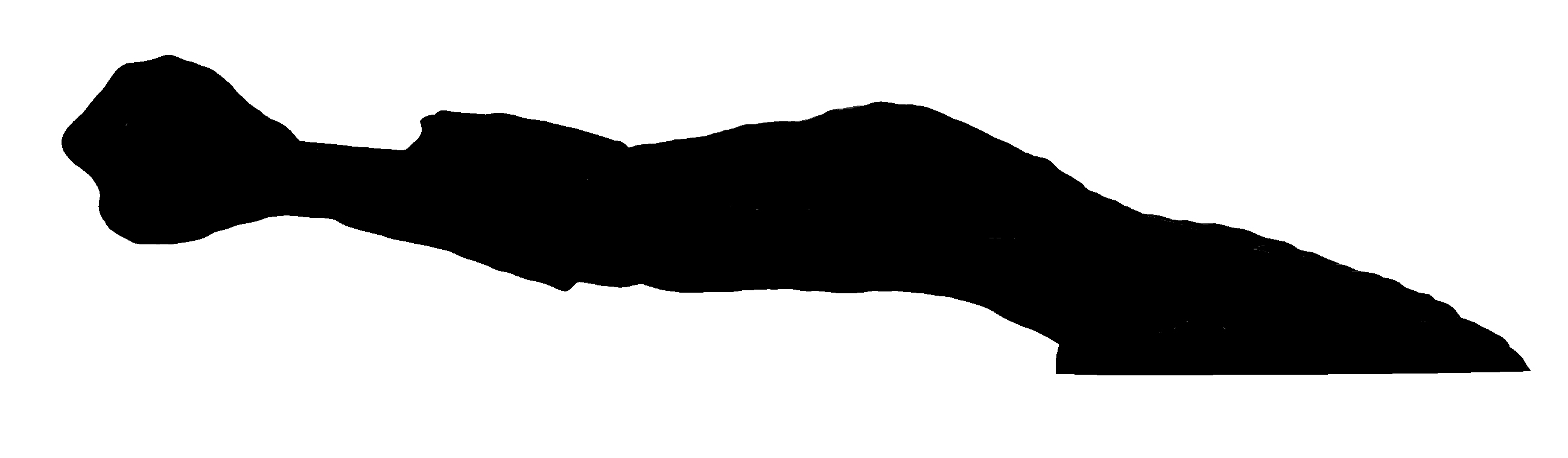

Supplement: Supplemental Information 6 [file peerj-13-20243-s006.zip › SUPPLEMENTARY FILE 7 Code_R2/Code shape occlusal/Silhouette_occlusal/Dicynodontoides_sp.jpg]

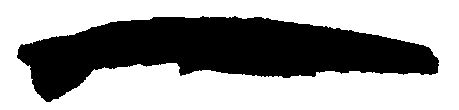

Supplement: Supplemental Information 6 [file peerj-13-20243-s006.zip › SUPPLEMENTARY FILE 7 Code_R2/Code shape occlusal/Silhouette_occlusal/Bolosaurus_major.jpg]

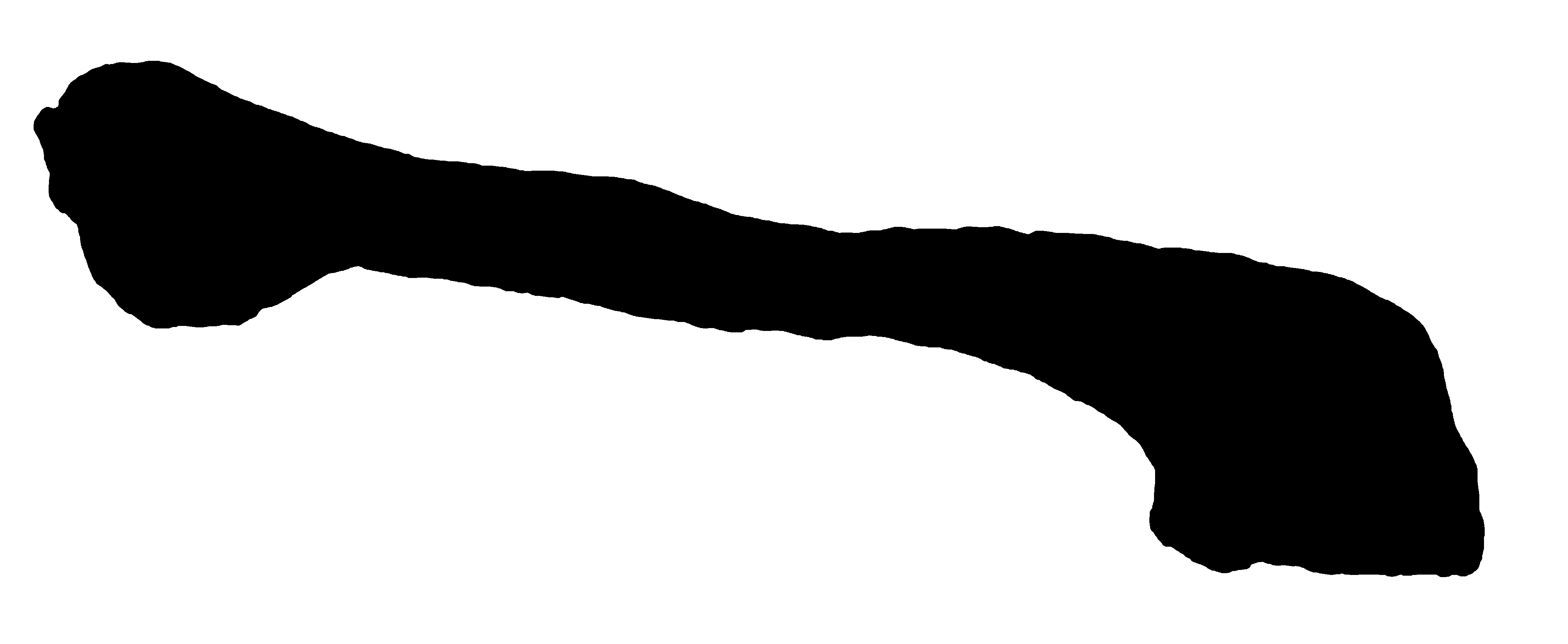

Supplement: Supplemental Information 6 [file peerj-13-20243-s006.zip › SUPPLEMENTARY FILE 7 Code_R2/Code shape occlusal/Silhouette_occlusal/Jonkeria_truculenta.jpg]

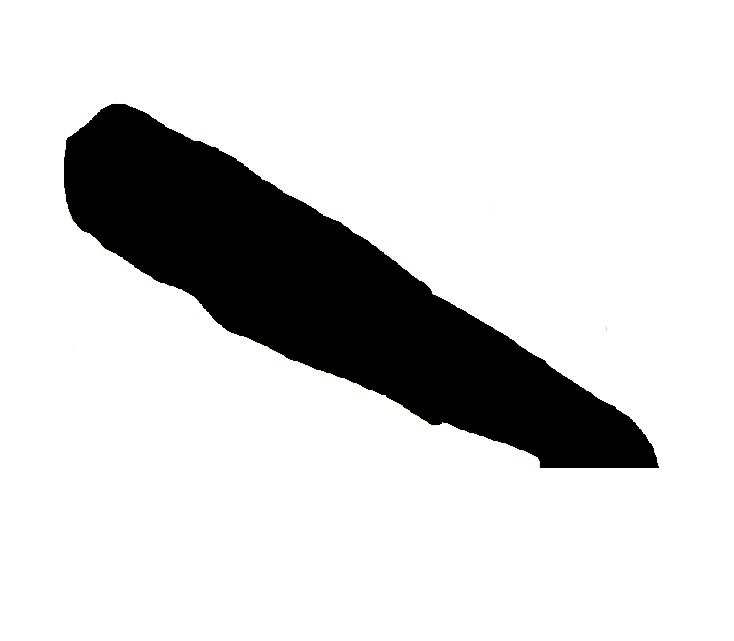

Supplement: Supplemental Information 6 [file peerj-13-20243-s006.zip › SUPPLEMENTARY FILE 7 Code_R2/Code shape occlusal/Silhouette_occlusal/Pantylus_cordatus.jpg]

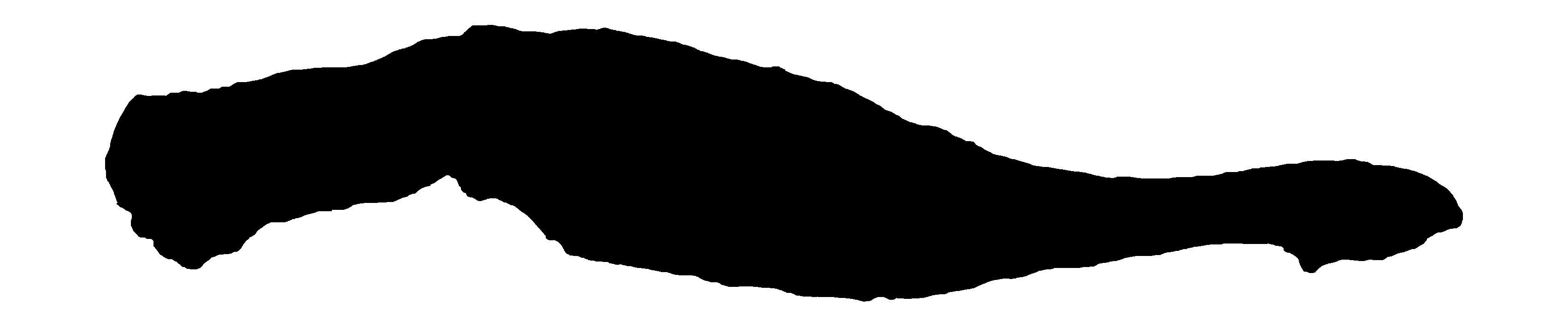

Supplement: Supplemental Information 6 [file peerj-13-20243-s006.zip › SUPPLEMENTARY FILE 7 Code_R2/Code shape occlusal/Silhouette_occlusal/Edaphosaurus_boanerges.jpg]

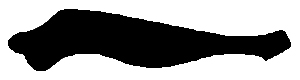

Supplement: Supplemental Information 6 [file peerj-13-20243-s006.zip › SUPPLEMENTARY FILE 7 Code_R2/Code shape occlusal/Silhouette_occlusal/Sumidadectes_chozaensis.jpg]

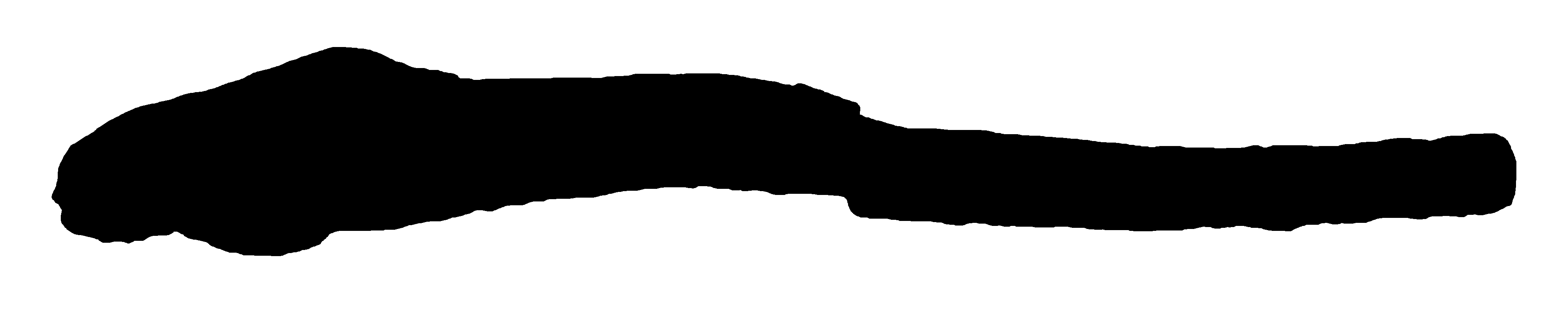

Supplement: Supplemental Information 6 [file peerj-13-20243-s006.zip › SUPPLEMENTARY FILE 7 Code_R2/Code shape occlusal/Silhouette_occlusal/Sauroctonus_progressus.jpg]

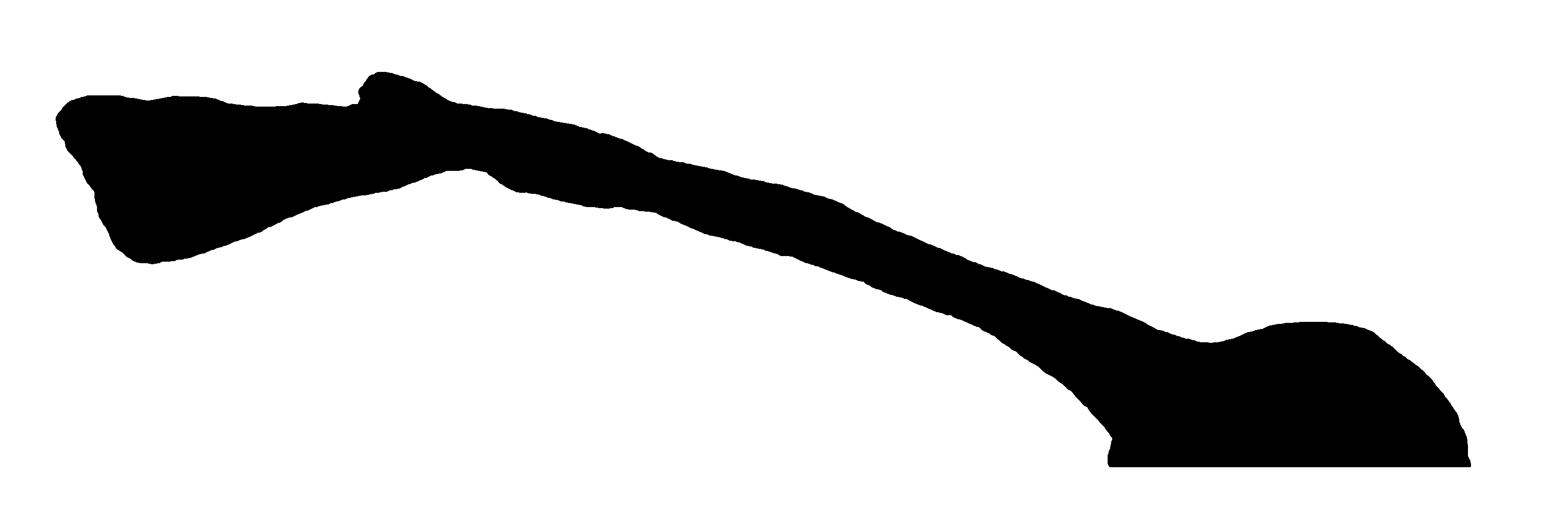

Supplement: Supplemental Information 6 [file peerj-13-20243-s006.zip › SUPPLEMENTARY FILE 7 Code_R2/Code shape occlusal/Silhouette_occlusal/Delphaciognathus_paucidens.jpg]

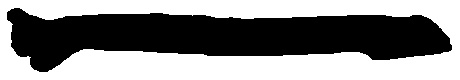

Supplement: Supplemental Information 6 [file peerj-13-20243-s006.zip › SUPPLEMENTARY FILE 7 Code_R2/Code shape occlusal/Silhouette_occlusal/Syodon_efremovi.jpg]

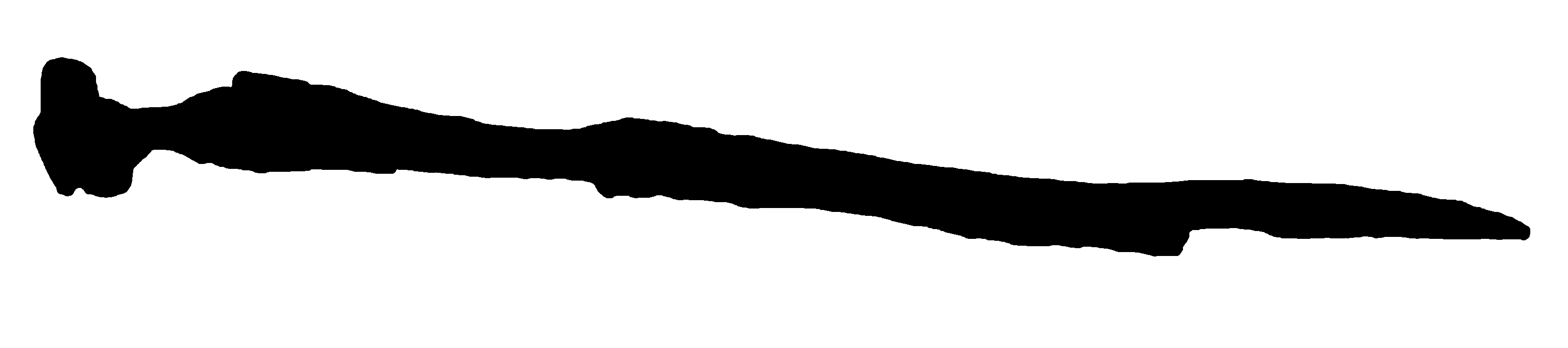

Supplement: Supplemental Information 6 [file peerj-13-20243-s006.zip › SUPPLEMENTARY FILE 7 Code_R2/Code shape occlusal/Silhouette_occlusal/Reiszia_gubini.jpg]

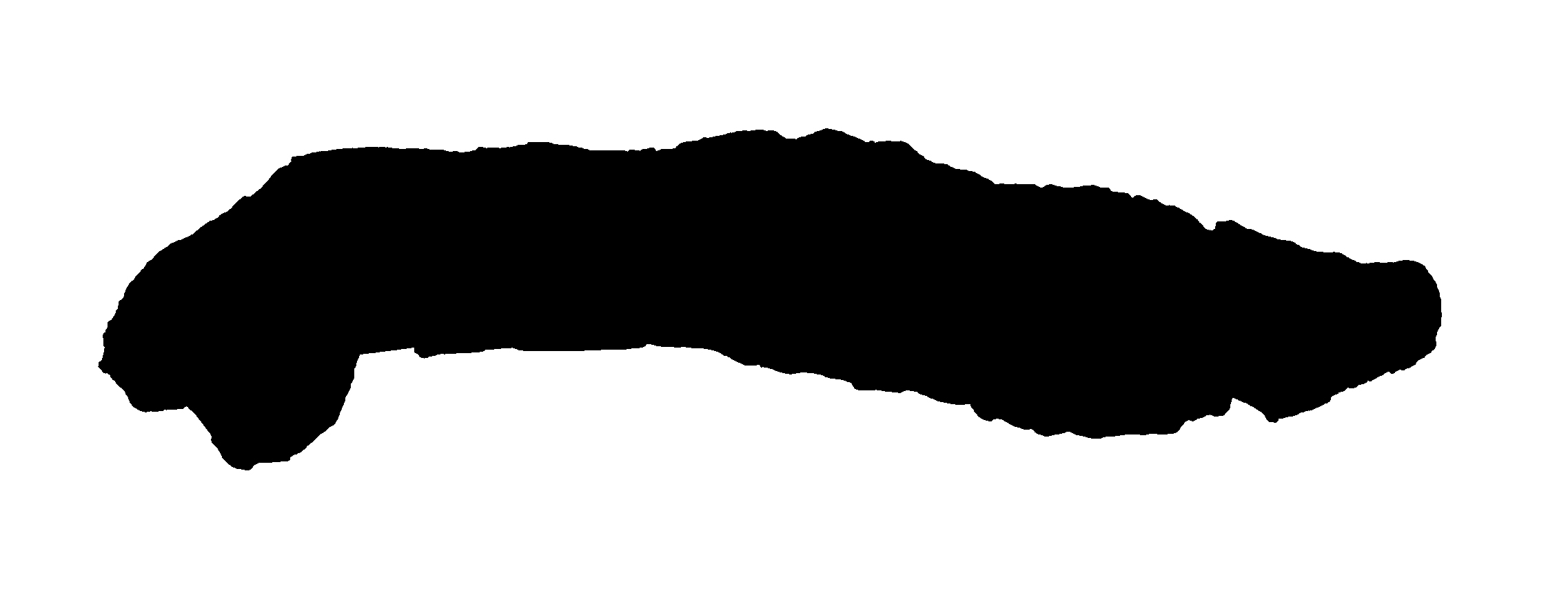

Supplement: Supplemental Information 6 [file peerj-13-20243-s006.zip › SUPPLEMENTARY FILE 7 Code_R2/Code shape occlusal/Silhouette_occlusal/Desmatodon_hesperis.jpg]

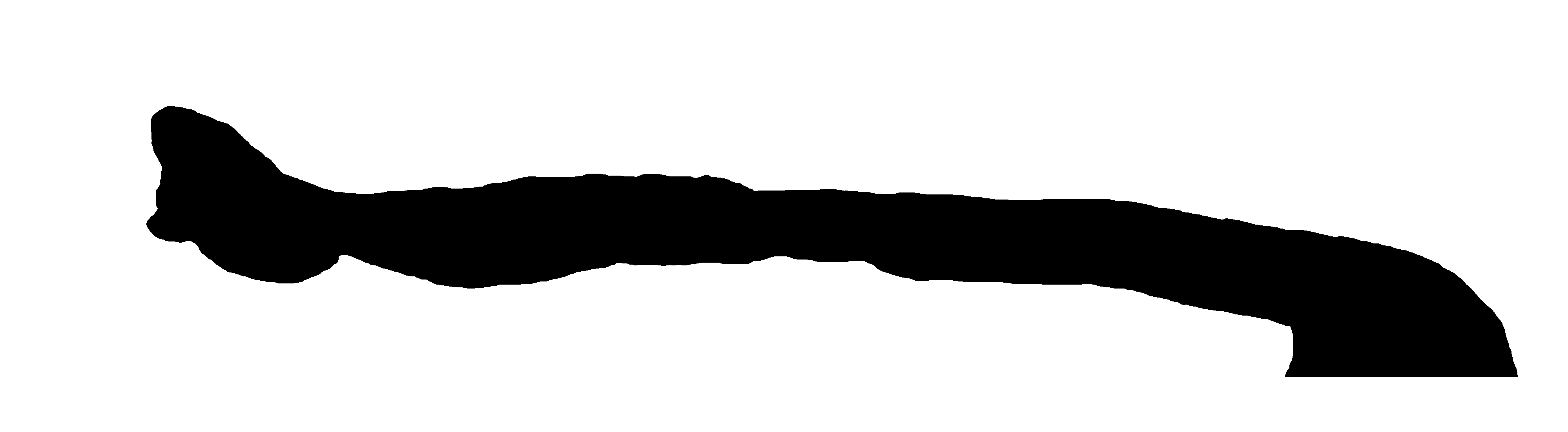

Supplement: Supplemental Information 6 [file peerj-13-20243-s006.zip › SUPPLEMENTARY FILE 7 Code_R2/Code shape occlusal/Silhouette_occlusal/Galeops_whaitsi.jpg]

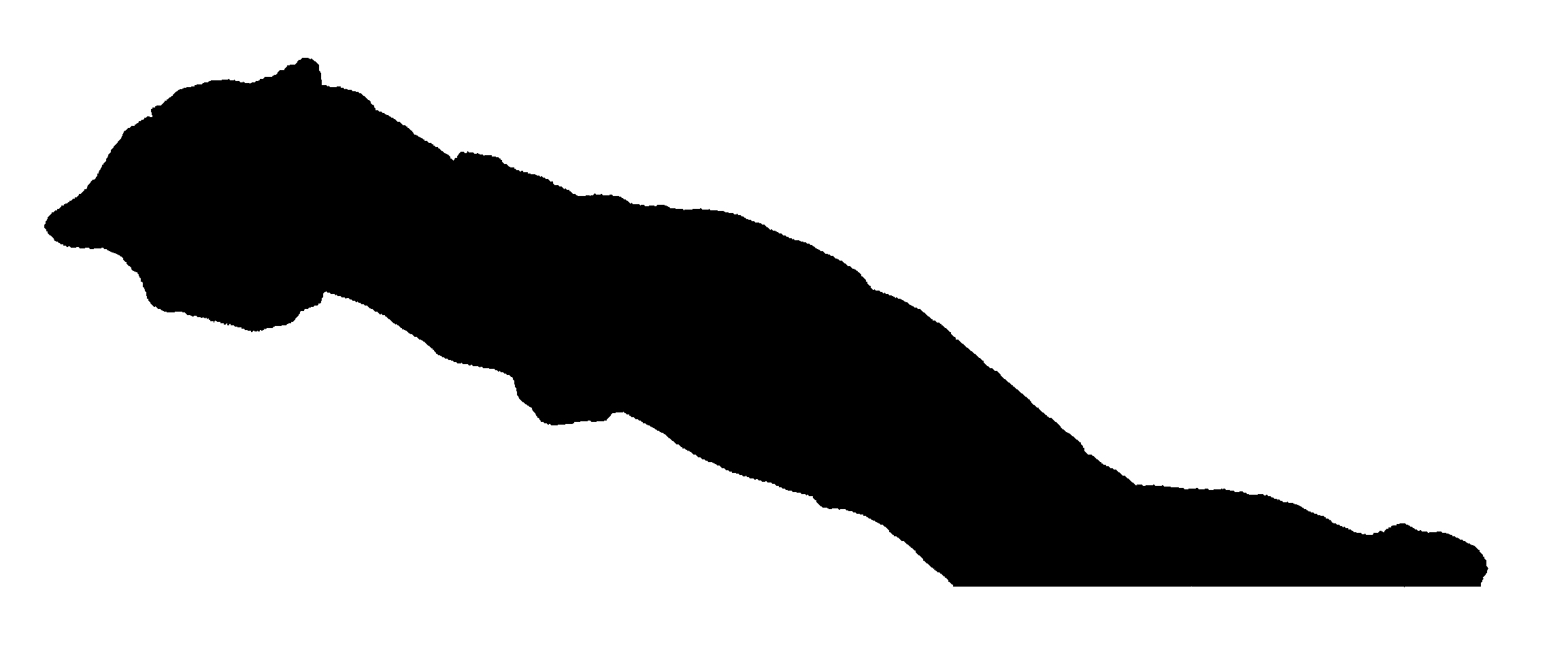

Supplement: Supplemental Information 6 [file peerj-13-20243-s006.zip › SUPPLEMENTARY FILE 7 Code_R2/Code shape occlusal/Silhouette_occlusal/Euptychognathus_bathyrhynchus.jpg]

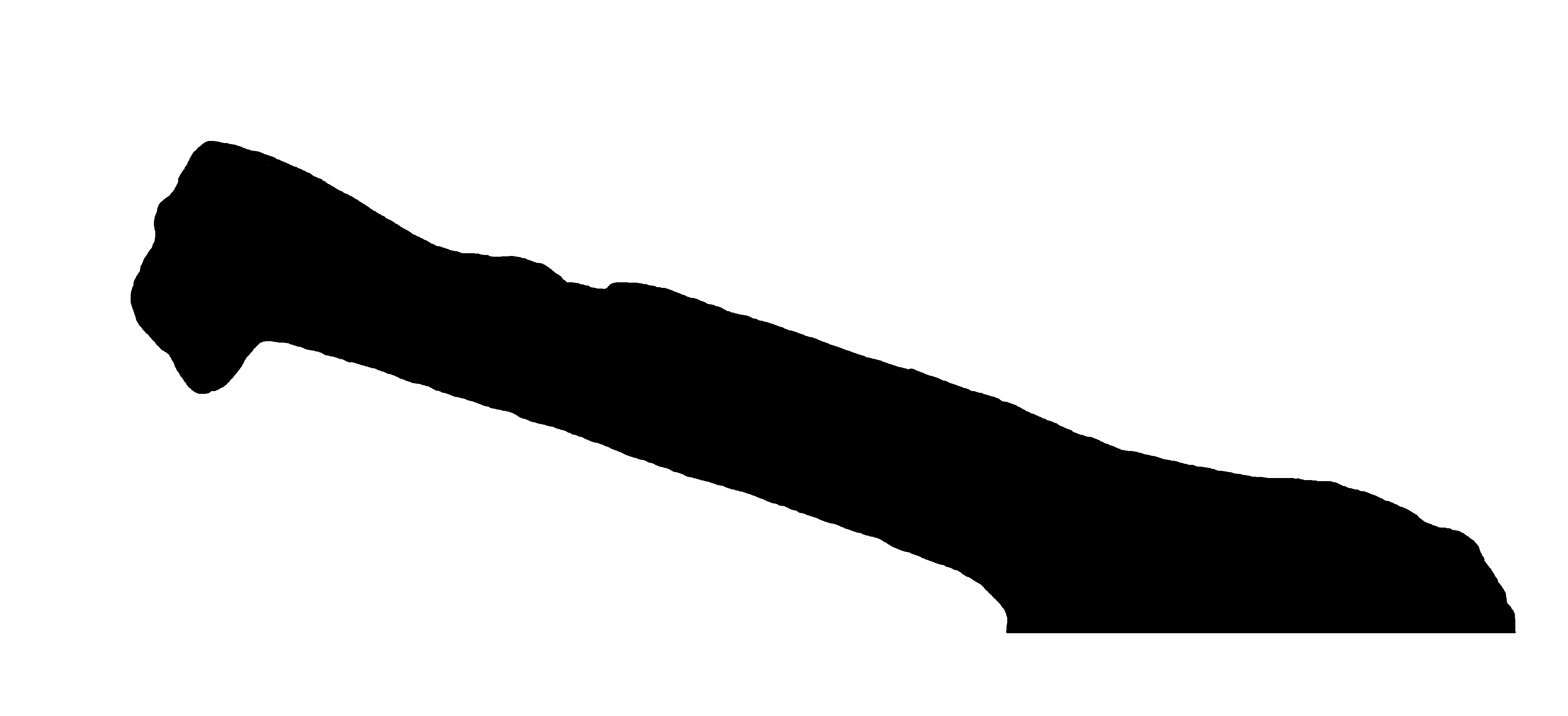

Supplement: Supplemental Information 6 [file peerj-13-20243-s006.zip › SUPPLEMENTARY FILE 7 Code_R2/Code shape occlusal/Silhouette_occlusal/Oudenodon_bainii.jpg]

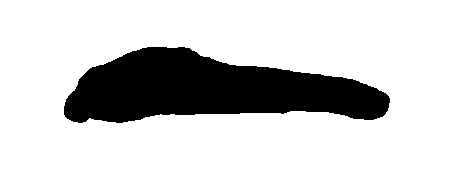

Supplement: Supplemental Information 6 [file peerj-13-20243-s006.zip › SUPPLEMENTARY FILE 7 Code_R2/Code shape occlusal/Silhouette_occlusal/Delorhynchus_cifelli.jpg]

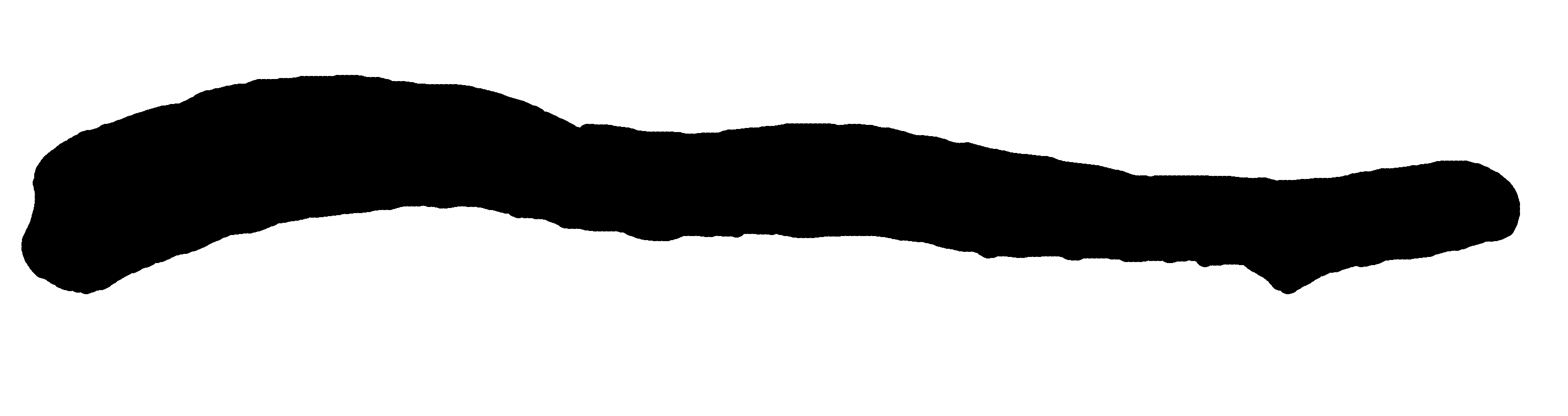

Supplement: Supplemental Information 6 [file peerj-13-20243-s006.zip › SUPPLEMENTARY FILE 7 Code_R2/Code shape occlusal/Silhouette_occlusal/Sphenacodon_ferox.jpg]

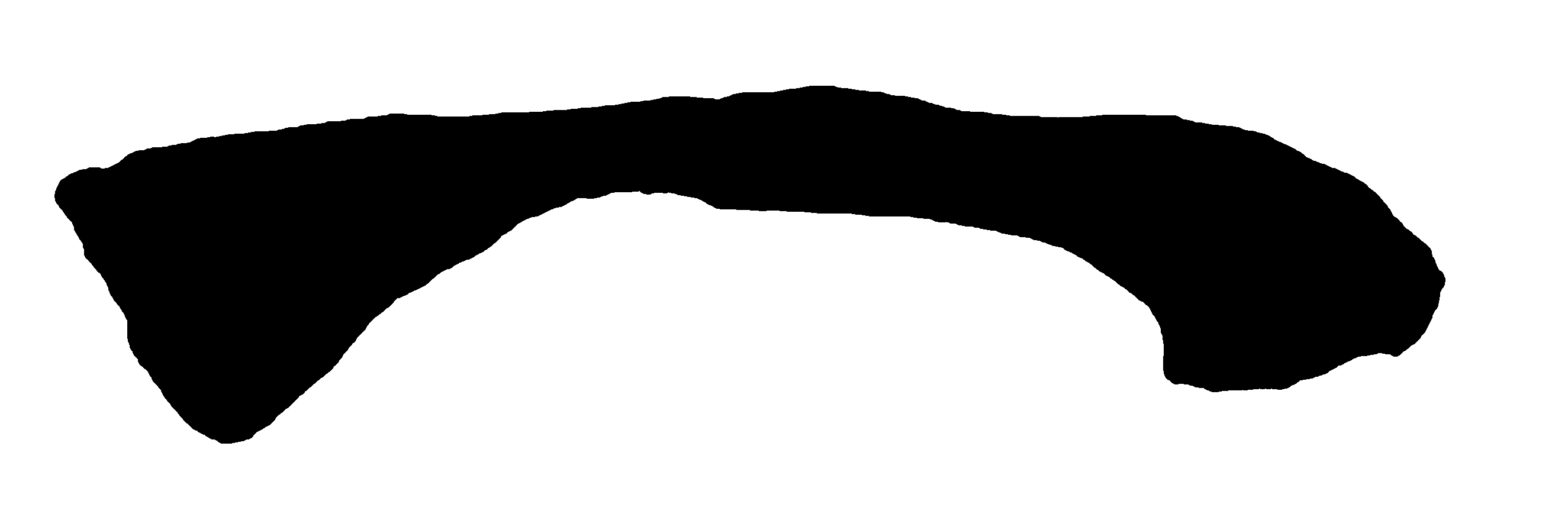

Supplement: Supplemental Information 6 [file peerj-13-20243-s006.zip › SUPPLEMENTARY FILE 7 Code_R2/Code shape occlusal/Silhouette_occlusal/Moschops_capensis.jpg]

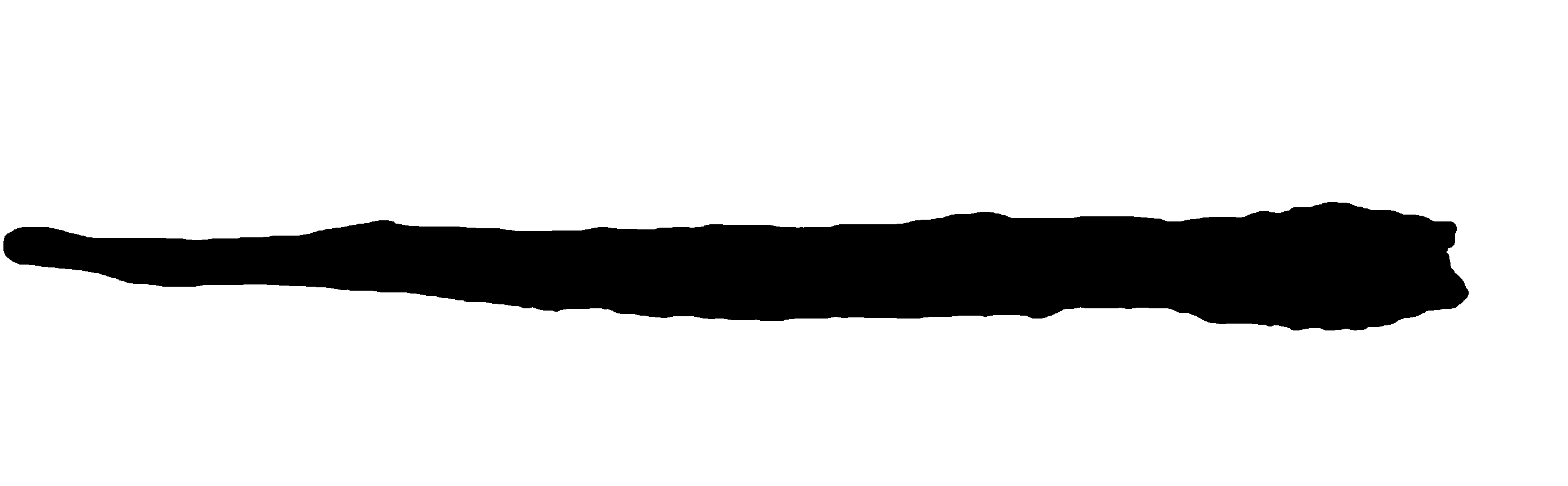

Supplement: Supplemental Information 6 [file peerj-13-20243-s006.zip › SUPPLEMENTARY FILE 7 Code_R2/Code shape occlusal/Silhouette_occlusal/Perplexisaurus_foveatus.jpg]

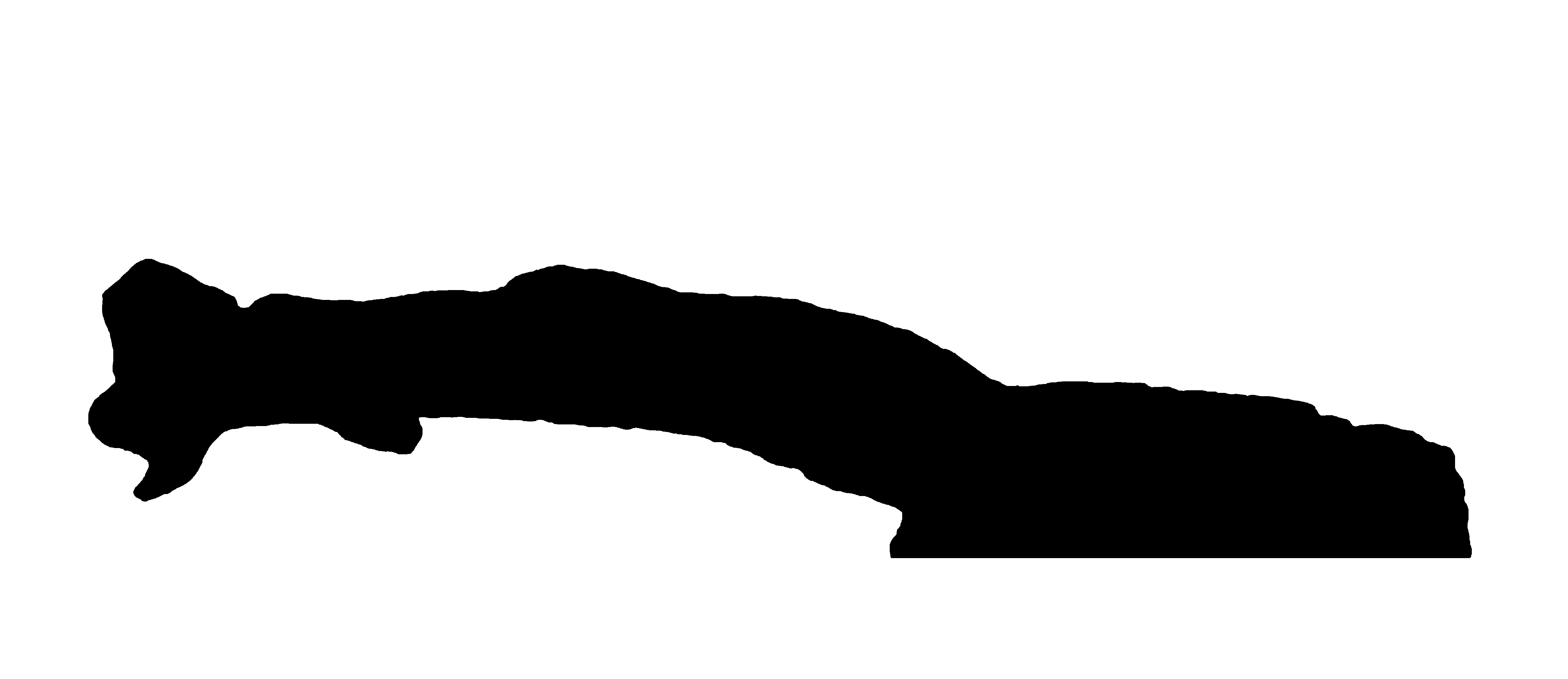

Supplement: Supplemental Information 6 [file peerj-13-20243-s006.zip › SUPPLEMENTARY FILE 7 Code_R2/Code shape occlusal/Silhouette_occlusal/Aulacephalodon_kapoliwacela.jpg]

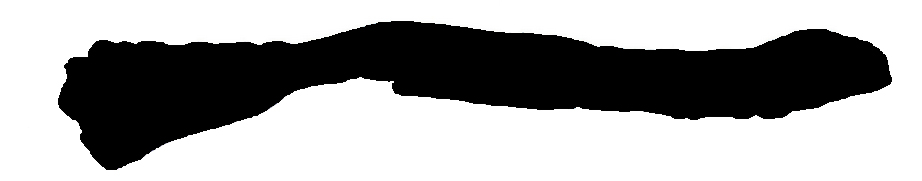

Supplement: Supplemental Information 6 [file peerj-13-20243-s006.zip › SUPPLEMENTARY FILE 7 Code_R2/Code shape occlusal/Silhouette_occlusal/Lycosuchus_vanderrieti.jpg]

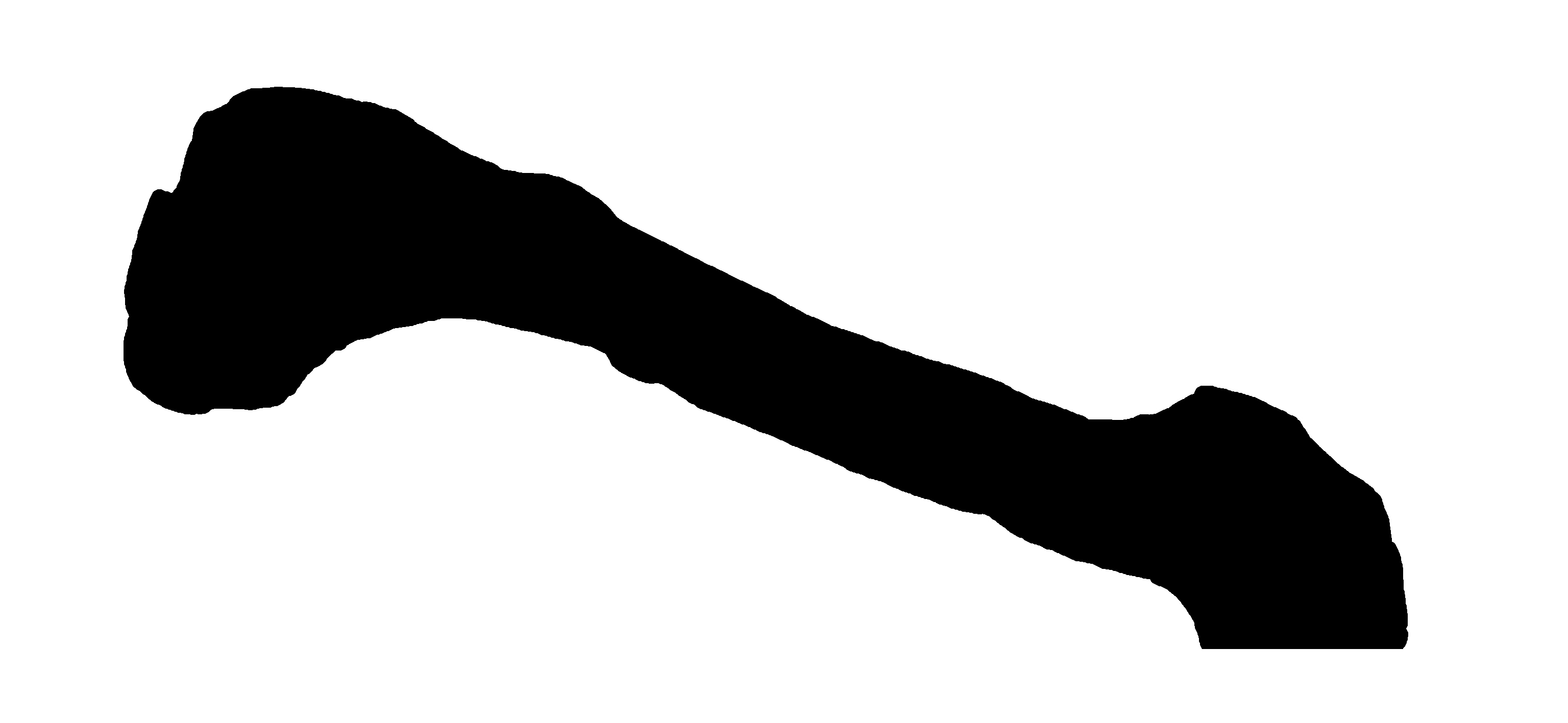

Supplement: Supplemental Information 6 [file peerj-13-20243-s006.zip › SUPPLEMENTARY FILE 7 Code_R2/Code shape occlusal/Silhouette_occlusal/Estemmenosuchus_mirabilis.jpg]

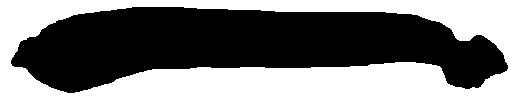

Supplement: Supplemental Information 6 [file peerj-13-20243-s006.zip › SUPPLEMENTARY FILE 7 Code_R2/Code shape occlusal/Silhouette_occlusal/Greererpeton_burkemorani.jpg]

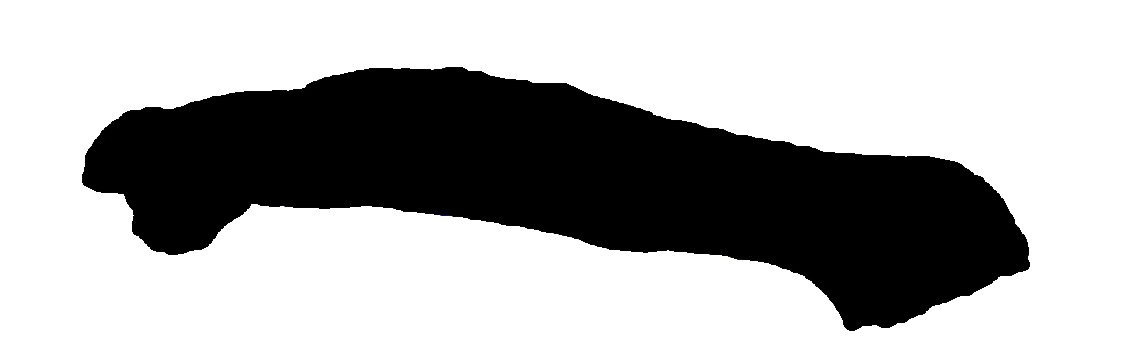

Supplement: Supplemental Information 6 [file peerj-13-20243-s006.zip › SUPPLEMENTARY FILE 7 Code_R2/Code shape occlusal/Silhouette_occlusal/Orobates_pabsti.jpg]

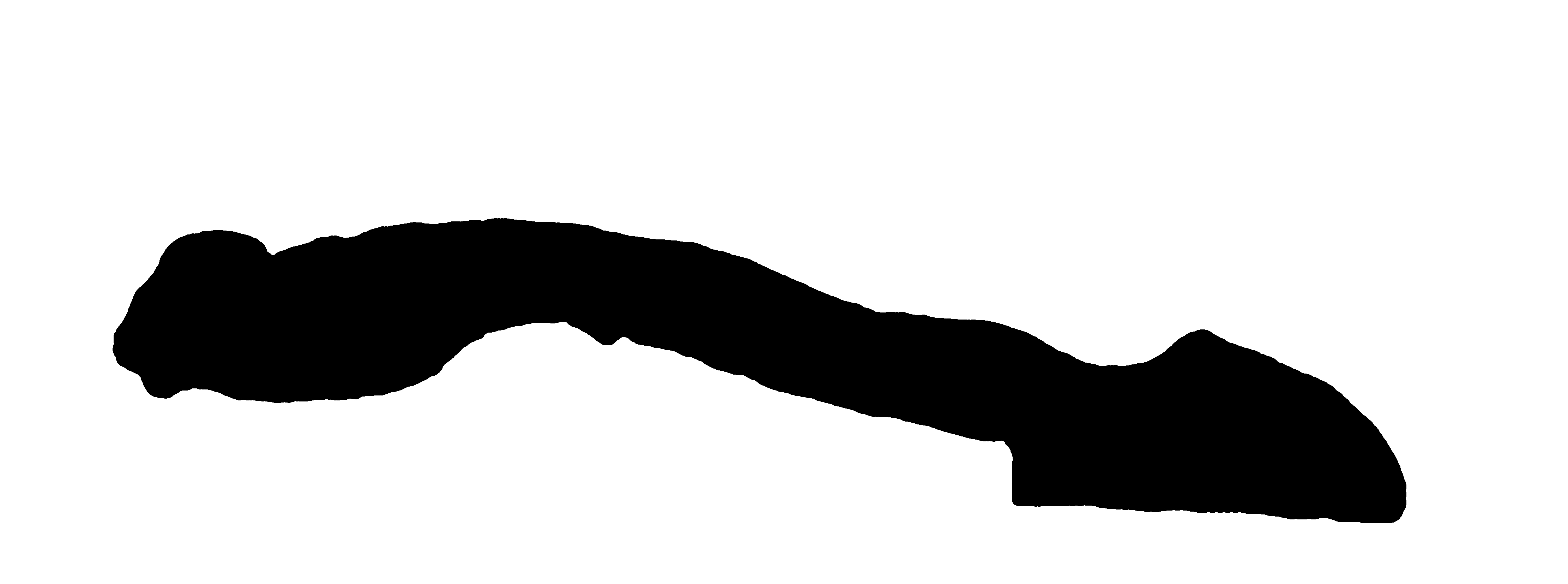

Supplement: Supplemental Information 6 [file peerj-13-20243-s006.zip › SUPPLEMENTARY FILE 7 Code_R2/Code shape occlusal/Silhouette_occlusal/Aelurosaurus_felinus.jpg]

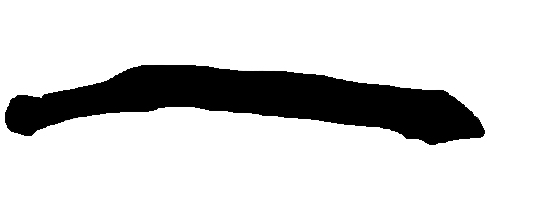

Supplement: Supplemental Information 6 [file peerj-13-20243-s006.zip › SUPPLEMENTARY FILE 7 Code_R2/Code shape occlusal/Silhouette_occlusal/Suminia_getmanovi.jpg]

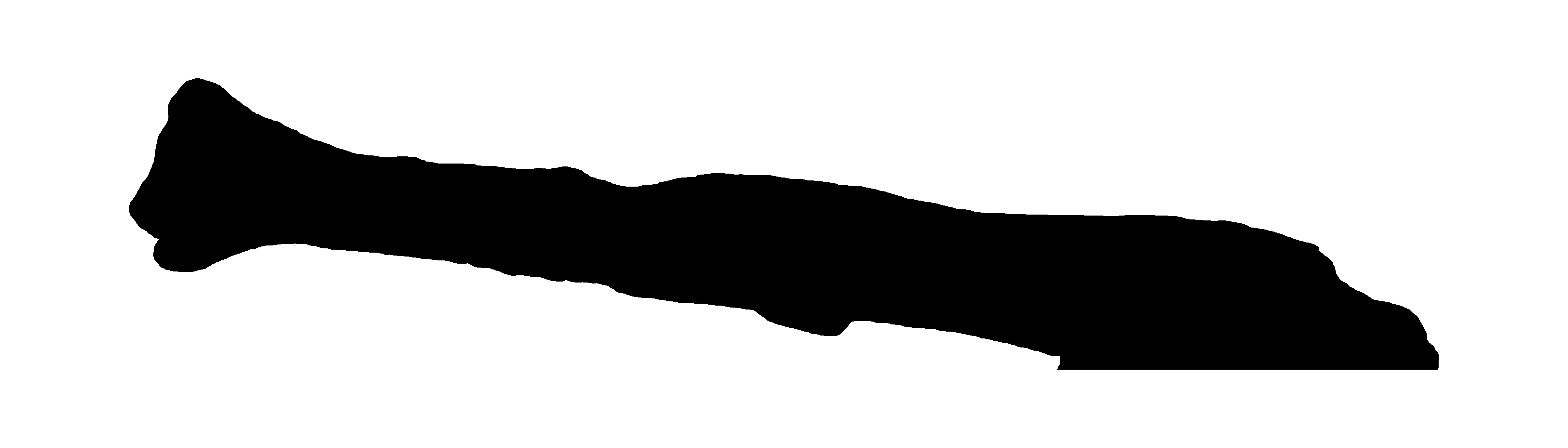

Supplement: Supplemental Information 6 [file peerj-13-20243-s006.zip › SUPPLEMENTARY FILE 7 Code_R2/Code shape occlusal/Silhouette_occlusal/Brachyprosopus_broomi.jpg]

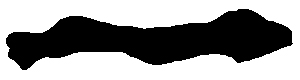

Supplement: Supplemental Information 6 [file peerj-13-20243-s006.zip › SUPPLEMENTARY FILE 7 Code_R2/Code shape occlusal/Silhouette_occlusal/Ulemica_invisa.jpg]

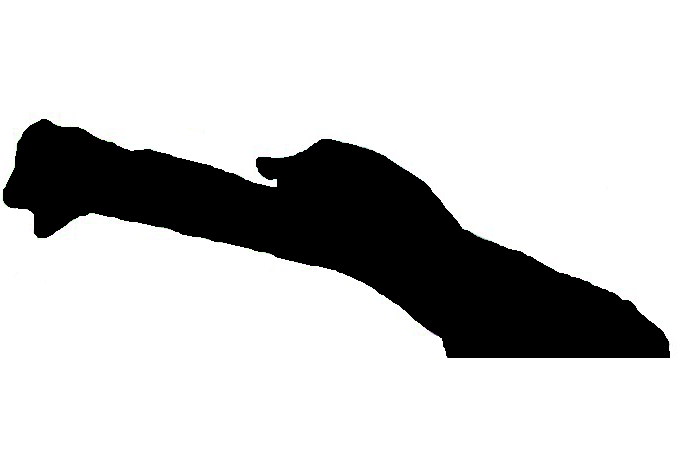

Supplement: Supplemental Information 6 [file peerj-13-20243-s006.zip › SUPPLEMENTARY FILE 7 Code_R2/Code shape occlusal/Silhouette_occlusal/Niassodon_mfumukasi.jpg]

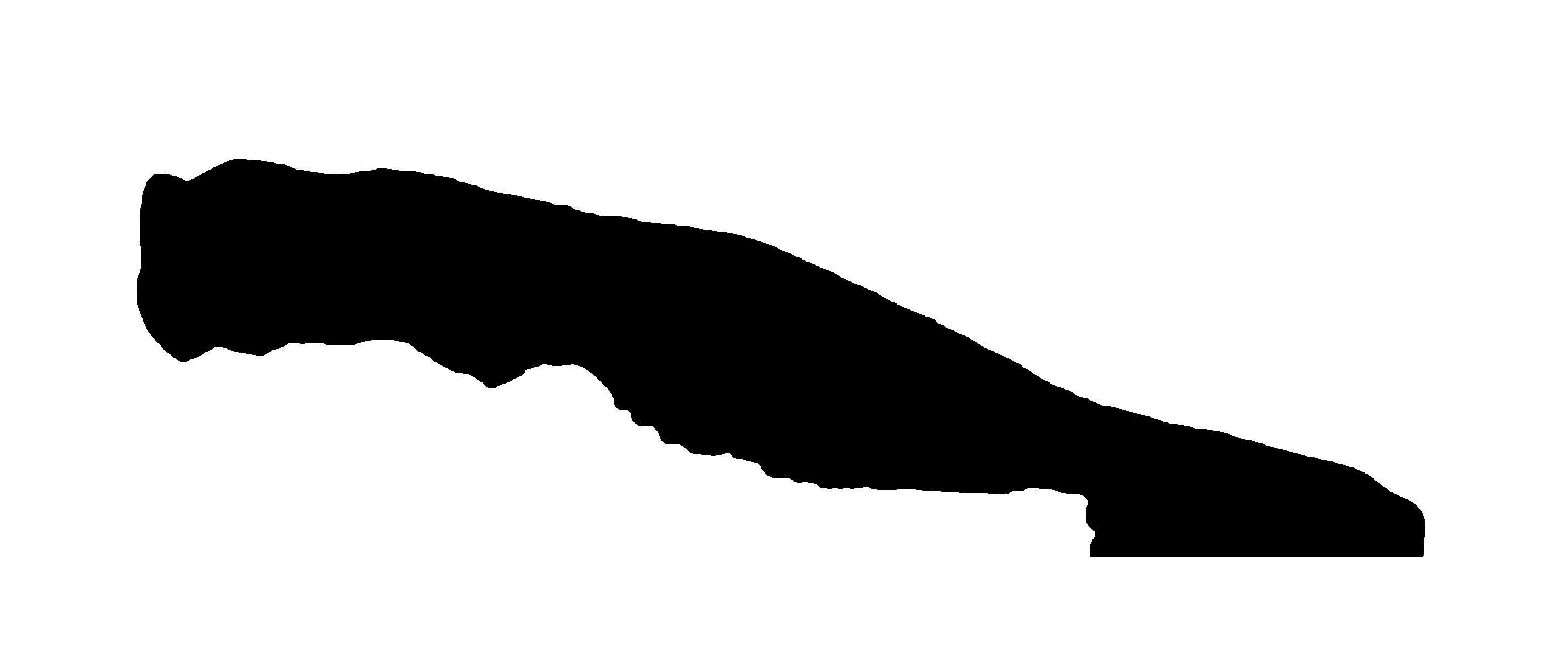

Supplement: Supplemental Information 6 [file peerj-13-20243-s006.zip › SUPPLEMENTARY FILE 7 Code_R2/Code shape occlusal/Silhouette_occlusal/Edaphosaurus_cruciger.jpg]

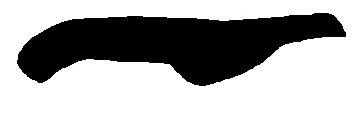

Supplement: Supplemental Information 6 [file peerj-13-20243-s006.zip › SUPPLEMENTARY FILE 7 Code_R2/Code shape occlusal/Silhouette_occlusal/Gansurhinus_naobaogouensis.jpg]

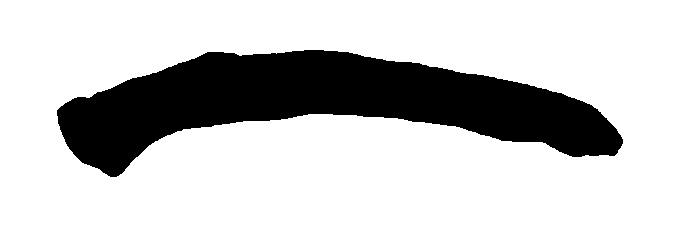

Supplement: Supplemental Information 6 [file peerj-13-20243-s006.zip › SUPPLEMENTARY FILE 7 Code_R2/Code shape occlusal/Silhouette_occlusal/Crassigyrinus_scoticus.jpg]

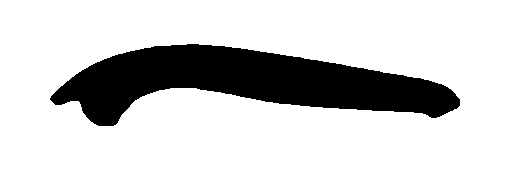

Supplement: Supplemental Information 6 [file peerj-13-20243-s006.zip › SUPPLEMENTARY FILE 7 Code_R2/Code shape occlusal/Silhouette_occlusal/Captorhinus_aguti.jpg]

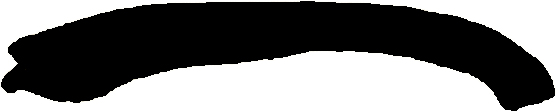

Supplement: Supplemental Information 6 [file peerj-13-20243-s006.zip › SUPPLEMENTARY FILE 7 Code_R2/Code shape occlusal/Silhouette_occlusal/Trimerorhachis_insignis.jpg]

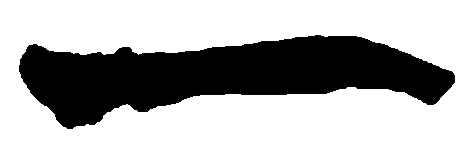

Supplement: Supplemental Information 6 [file peerj-13-20243-s006.zip › SUPPLEMENTARY FILE 7 Code_R2/Code shape occlusal/Silhouette_occlusal/Carrolla_craddocki.jpg]

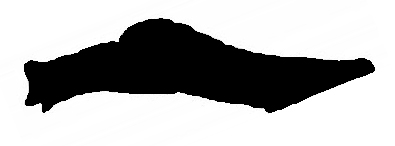

Supplement: Supplemental Information 6 [file peerj-13-20243-s006.zip › SUPPLEMENTARY FILE 7 Code_R2/Code shape occlusal/Silhouette_occlusal/Endothiodon_tolani.jpg]

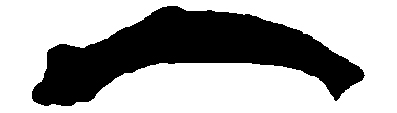

Supplement: Supplemental Information 6 [file peerj-13-20243-s006.zip › SUPPLEMENTARY FILE 7 Code_R2/Code shape occlusal/Silhouette_occlusal/Nochelesaurus_alexanderi.jpg]

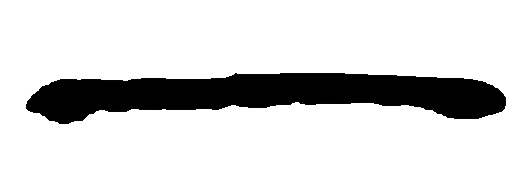

Supplement: Supplemental Information 6 [file peerj-13-20243-s006.zip › SUPPLEMENTARY FILE 7 Code_R2/Code shape occlusal/Silhouette_occlusal/Whatcheeria_deltae.jpg]

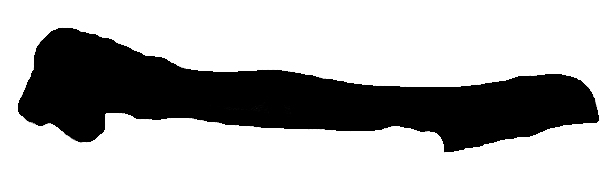

Supplement: Supplemental Information 6 [file peerj-13-20243-s006.zip › SUPPLEMENTARY FILE 7 Code_R2/Code shape occlusal/Silhouette_occlusal/Rhachiocephalus_magnus.jpg]

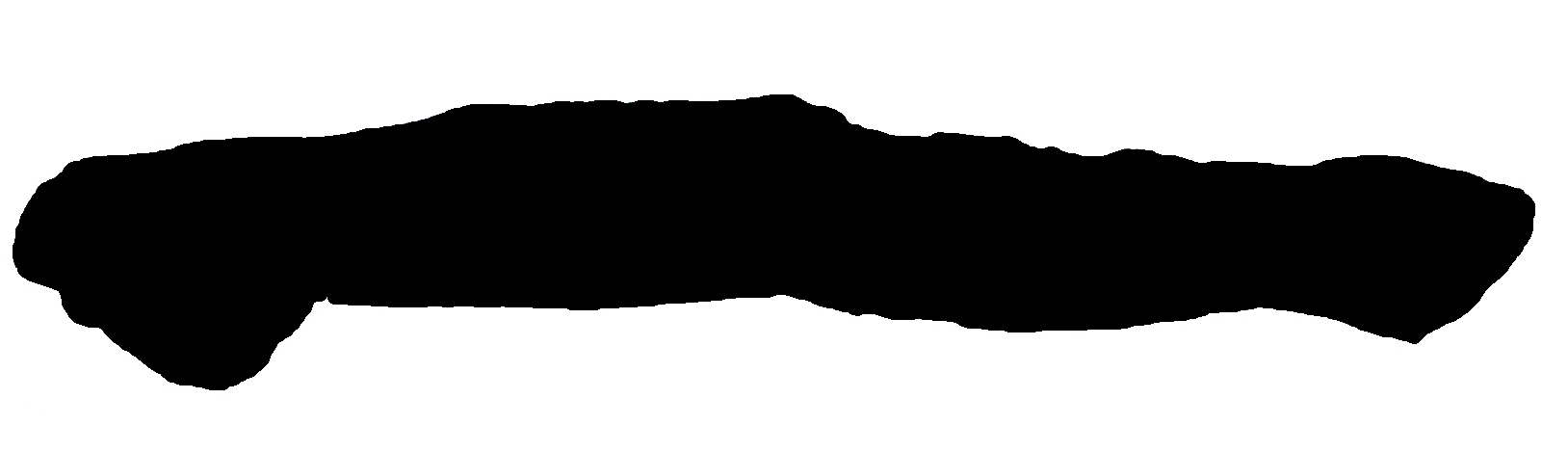

Supplement: Supplemental Information 6 [file peerj-13-20243-s006.zip › SUPPLEMENTARY FILE 7 Code_R2/Code shape occlusal/Silhouette_occlusal/Diadectes_absitus.jpg]

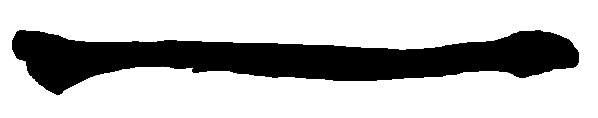

Supplement: Supplemental Information 6 [file peerj-13-20243-s006.zip › SUPPLEMENTARY FILE 7 Code_R2/Code shape occlusal/Silhouette_occlusal/Procynosuchus_delaharpeae.jpg]

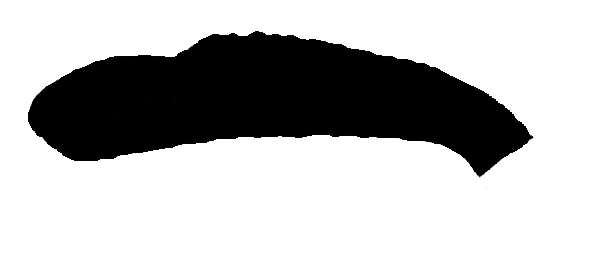

Supplement: Supplemental Information 6 [file peerj-13-20243-s006.zip › SUPPLEMENTARY FILE 7 Code_R2/Code shape occlusal/Silhouette_occlusal/Diplocaulus_magnicornis.jpg]

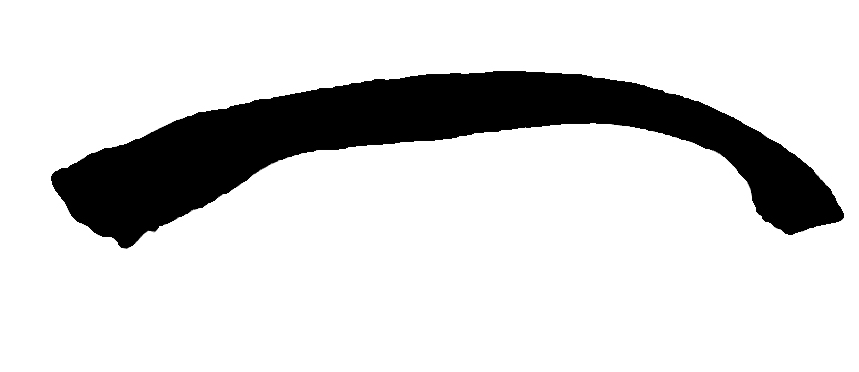

Supplement: Supplemental Information 6 [file peerj-13-20243-s006.zip › SUPPLEMENTARY FILE 7 Code_R2/Code shape occlusal/Silhouette_occlusal/Parrsboro_jaw.jpg]

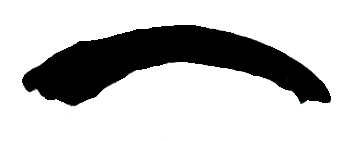

Supplement: Supplemental Information 6 [file peerj-13-20243-s006.zip › SUPPLEMENTARY FILE 7 Code_R2/Code shape occlusal/Silhouette_occlusal/Procuhy_nazariensis.jpg]

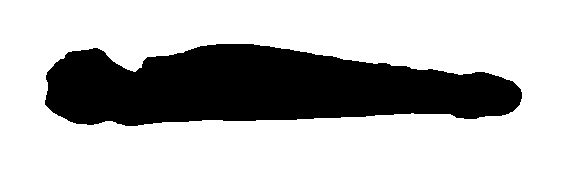

Supplement: Supplemental Information 6 [file peerj-13-20243-s006.zip › SUPPLEMENTARY FILE 7 Code_R2/Code shape occlusal/Silhouette_occlusal/Belebey_vegrandis.jpg]

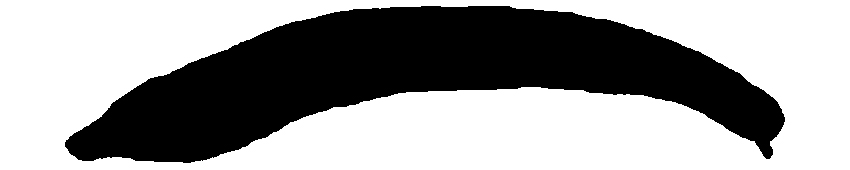

Supplement: Supplemental Information 6 [file peerj-13-20243-s006.zip › SUPPLEMENTARY FILE 7 Code_R2/Code shape occlusal/Silhouette_occlusal/Acroplous_vorax.jpg]

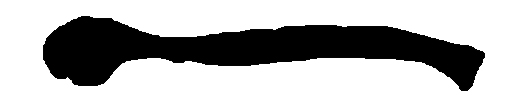

Supplement: Supplemental Information 6 [file peerj-13-20243-s006.zip › SUPPLEMENTARY FILE 7 Code_R2/Code shape occlusal/Silhouette_occlusal/Shihtienfenia_permica.jpg]

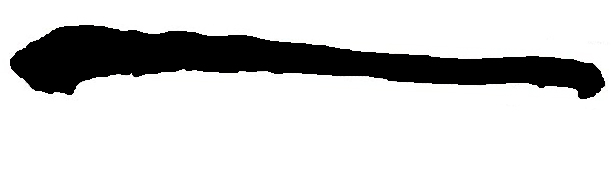

Supplement: Supplemental Information 6 [file peerj-13-20243-s006.zip › SUPPLEMENTARY FILE 7 Code_R2/Code shape occlusal/Silhouette_occlusal/Laosuchus_hun.jpg]

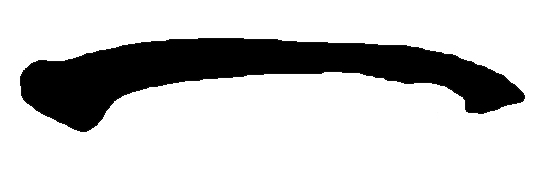

Supplement: Supplemental Information 6 [file peerj-13-20243-s006.zip › SUPPLEMENTARY FILE 7 Code_R2/Code shape occlusal/Silhouette_occlusal/Acheloma_sp.jpg]

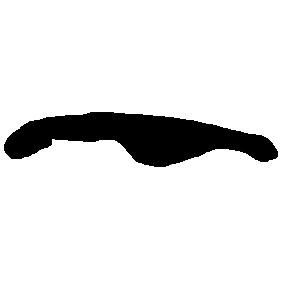

Supplement: Supplemental Information 6 [file peerj-13-20243-s006.zip › SUPPLEMENTARY FILE 7 Code_R2/Code shape occlusal/Silhouette_occlusal/Tramuntanasaurus_tiai.jpg]

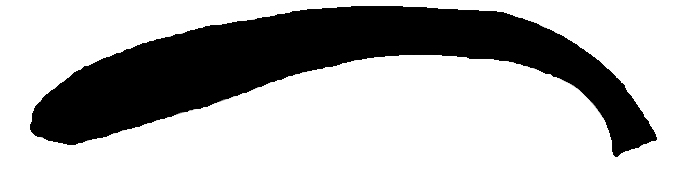

Supplement: Supplemental Information 6 [file peerj-13-20243-s006.zip › SUPPLEMENTARY FILE 7 Code_R2/Code shape occlusal/Silhouette_occlusal/Platyrhinops_lyelli.jpg]

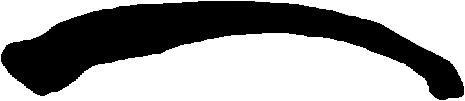

Supplement: Supplemental Information 6 [file peerj-13-20243-s006.zip › SUPPLEMENTARY FILE 7 Code_R2/Code shape occlusal/Silhouette_occlusal/Eryops_megacephalus.jpg]

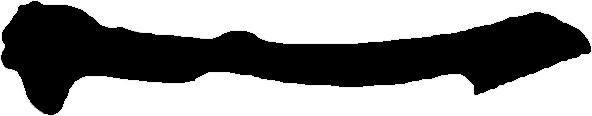

Supplement: Supplemental Information 6 [file peerj-13-20243-s006.zip › SUPPLEMENTARY FILE 7 Code_R2/Code shape occlusal/Silhouette_occlusal/Purlovia_maxima.jpg]

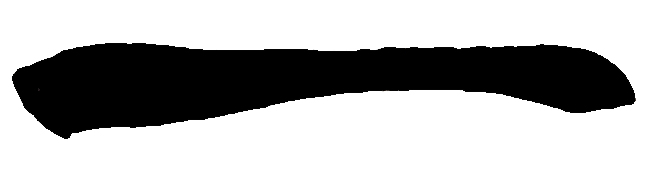

Supplement: Supplemental Information 6 [file peerj-13-20243-s006.zip › SUPPLEMENTARY FILE 7 Code_R2/Code shape occlusal/Silhouette_occlusal/Megalocephalus_pachycephalus.jpg]

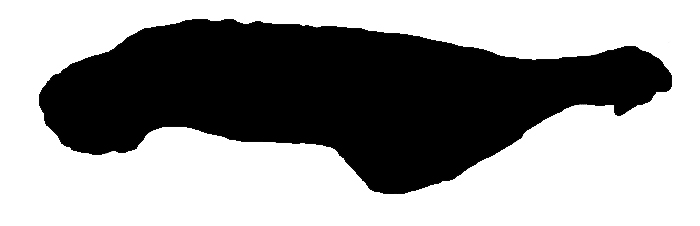

Supplement: Supplemental Information 6 [file peerj-13-20243-s006.zip › SUPPLEMENTARY FILE 7 Code_R2/Code shape occlusal/Silhouette_occlusal/Moradisaurus_grandis.jpg]

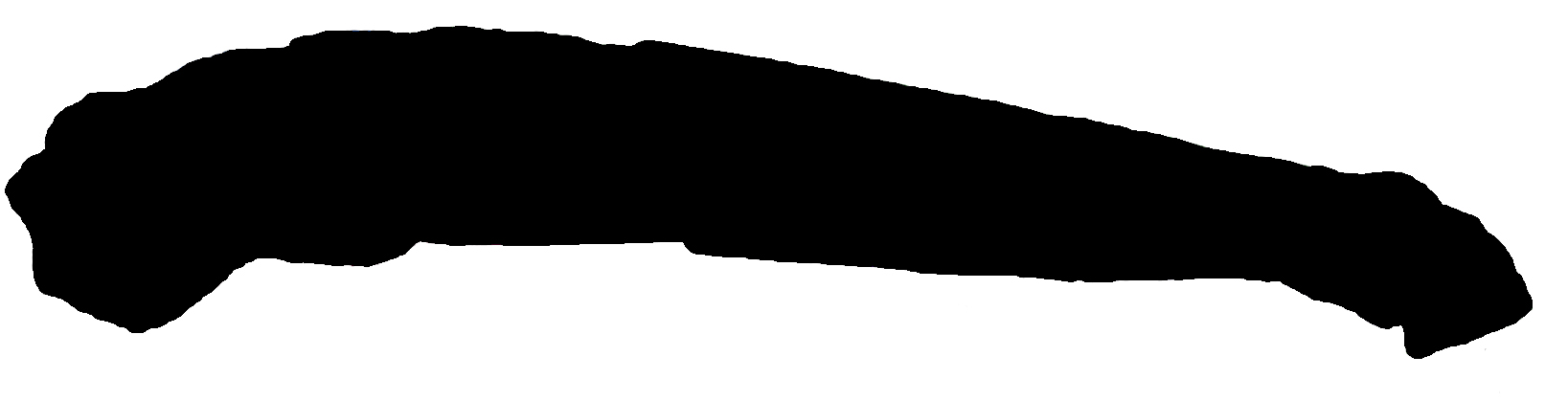

Supplement: Supplemental Information 6 [file peerj-13-20243-s006.zip › SUPPLEMENTARY FILE 7 Code_R2/Code shape occlusal/Silhouette_occlusal/Feeserpeton_oklahomensis.jpg]

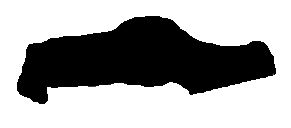

Supplement: Supplemental Information 6 [file peerj-13-20243-s006.zip › SUPPLEMENTARY FILE 7 Code_R2/Code shape occlusal/Silhouette_occlusal/Kembawacela_kitchingi.jpg]

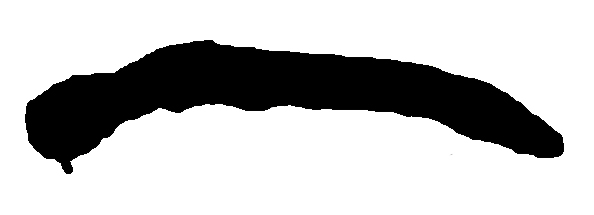

Supplement: Supplemental Information 6 [file peerj-13-20243-s006.zip › SUPPLEMENTARY FILE 7 Code_R2/Code shape occlusal/Silhouette_occlusal/Huskerpeton_englehorni.jpg]

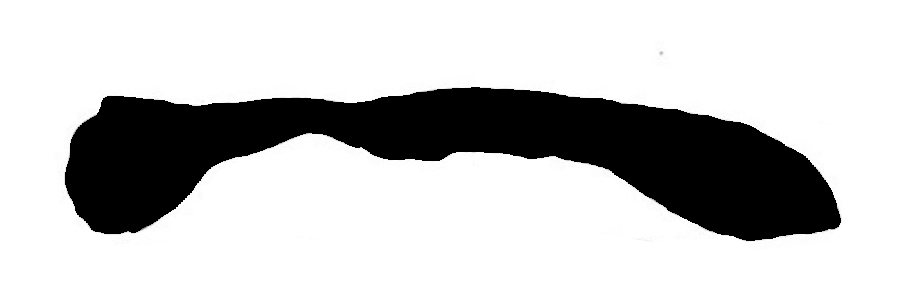

Supplement: Supplemental Information 6 [file peerj-13-20243-s006.zip › SUPPLEMENTARY FILE 7 Code_R2/Code shape occlusal/Silhouette_occlusal/Eoherpeton_watsoni.jpg]

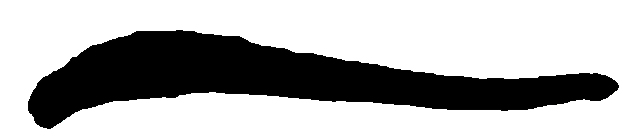

Supplement: Supplemental Information 6 [file peerj-13-20243-s006.zip › SUPPLEMENTARY FILE 7 Code_R2/Code shape occlusal/Silhouette_occlusal/Youngina_capensis.jpg]

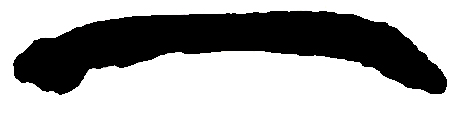

Supplement: Supplemental Information 6 [file peerj-13-20243-s006.zip › SUPPLEMENTARY FILE 7 Code_R2/Code shape occlusal/Silhouette_occlusal/Dendrerpeton_helogenes.jpg]

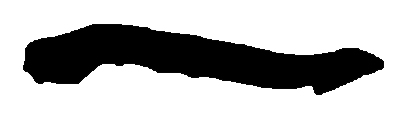

Supplement: Supplemental Information 6 [file peerj-13-20243-s006.zip › SUPPLEMENTARY FILE 7 Code_R2/Code shape occlusal/Silhouette_occlusal/Alveusdectes_fenestratus.jpg]

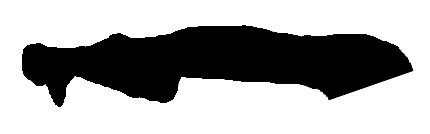

Supplement: Supplemental Information 6 [file peerj-13-20243-s006.zip › SUPPLEMENTARY FILE 7 Code_R2/Code shape occlusal/Silhouette_occlusal/Diictodon_feliceps.jpg]

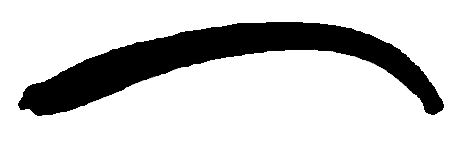

Supplement: Supplemental Information 6 [file peerj-13-20243-s006.zip › SUPPLEMENTARY FILE 7 Code_R2/Code shape occlusal/Silhouette_occlusal/Doragnathus_woodi.jpg]

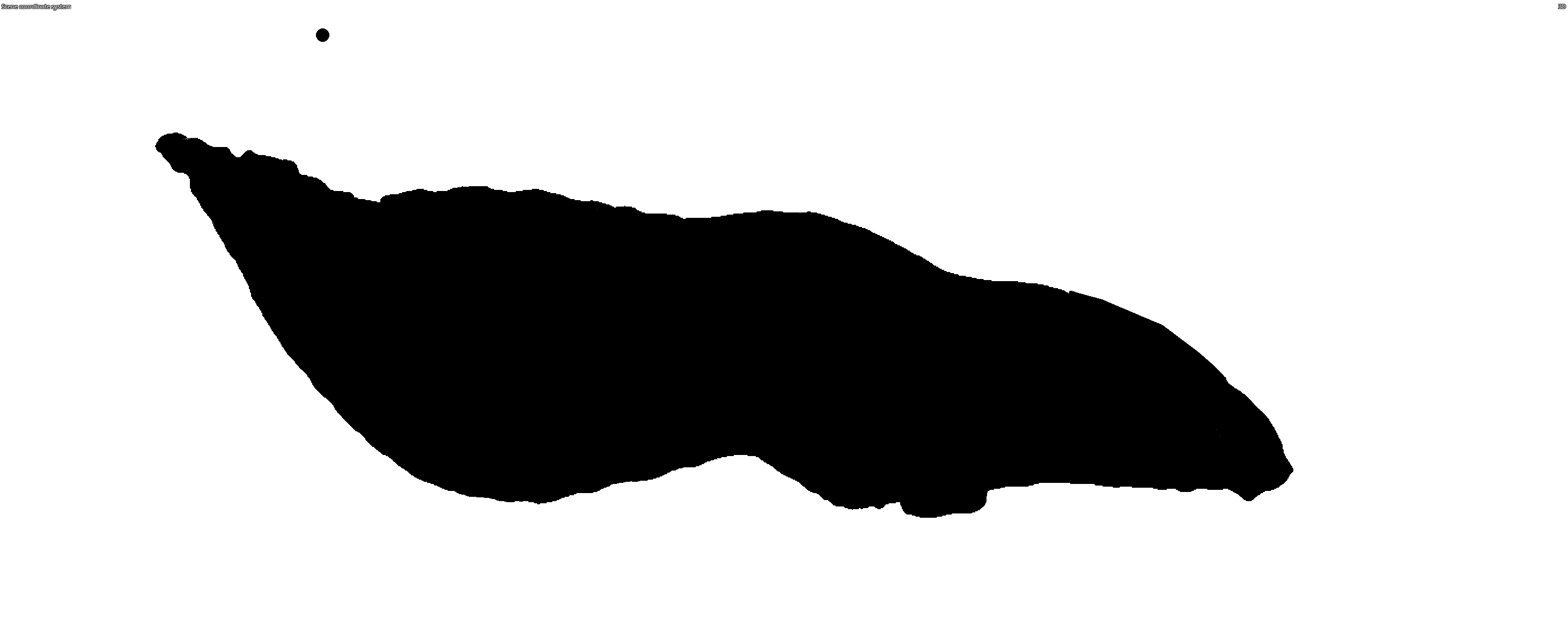

Supplement: Supplemental Information 6 [file peerj-13-20243-s006.zip › SUPPLEMENTARY FILE 7 Code_R2/Code shape lateral/Silhouette_lateral/Euptychognathus_bathyrhynchus.jpg]

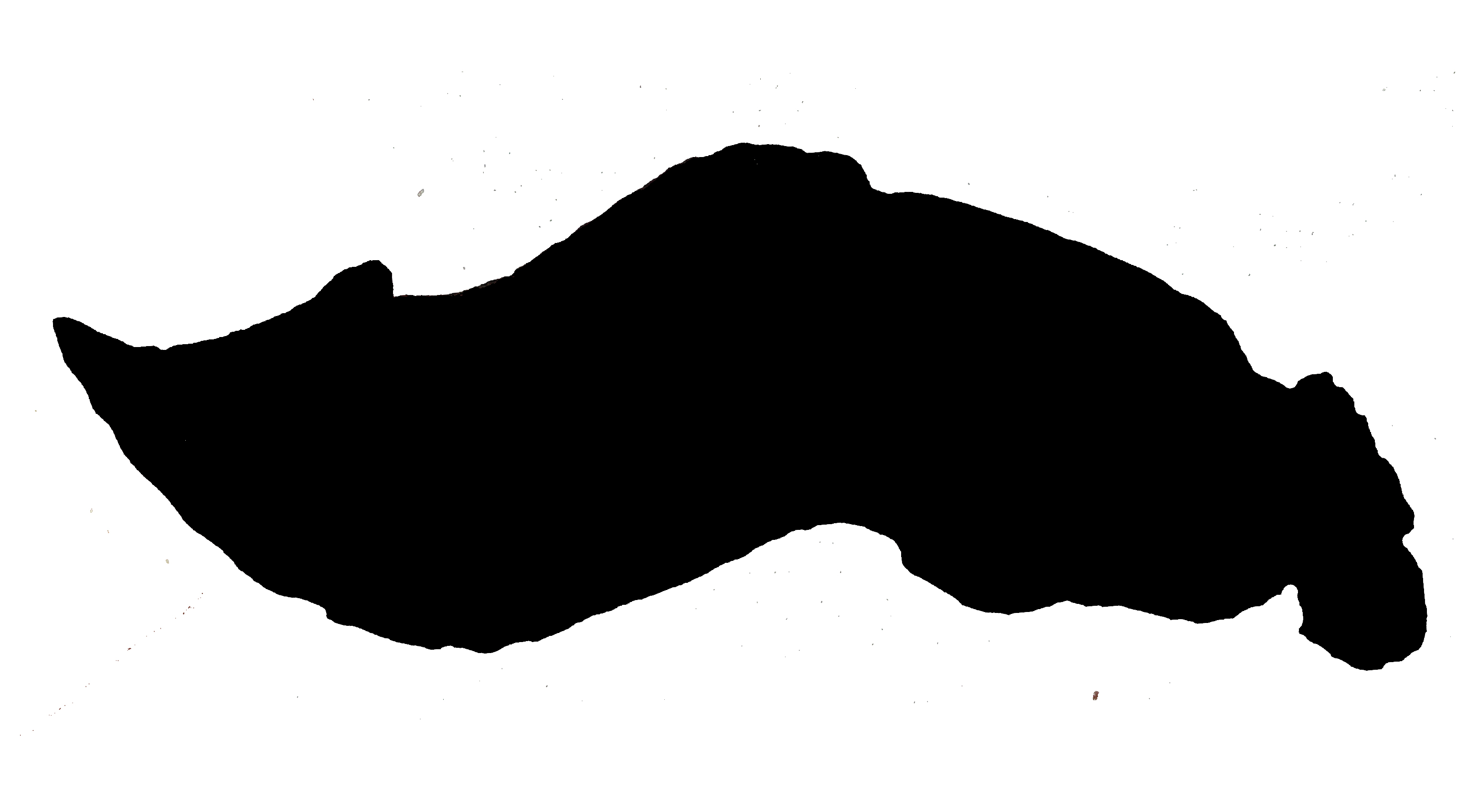

Supplement: Supplemental Information 6 [file peerj-13-20243-s006.zip › SUPPLEMENTARY FILE 7 Code_R2/Code shape lateral/Silhouette_lateral/Eosimops_newtoni.jpg]

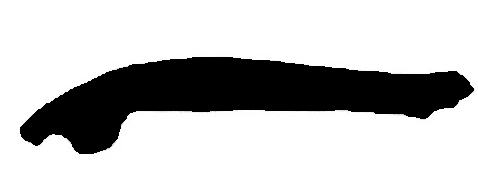

Supplement: Supplemental Information 6 [file peerj-13-20243-s006.zip › SUPPLEMENTARY FILE 7 Code_R2/Code shape occlusal/Silhouette_occlusal/Labidosaurus_hamatus.jpg]

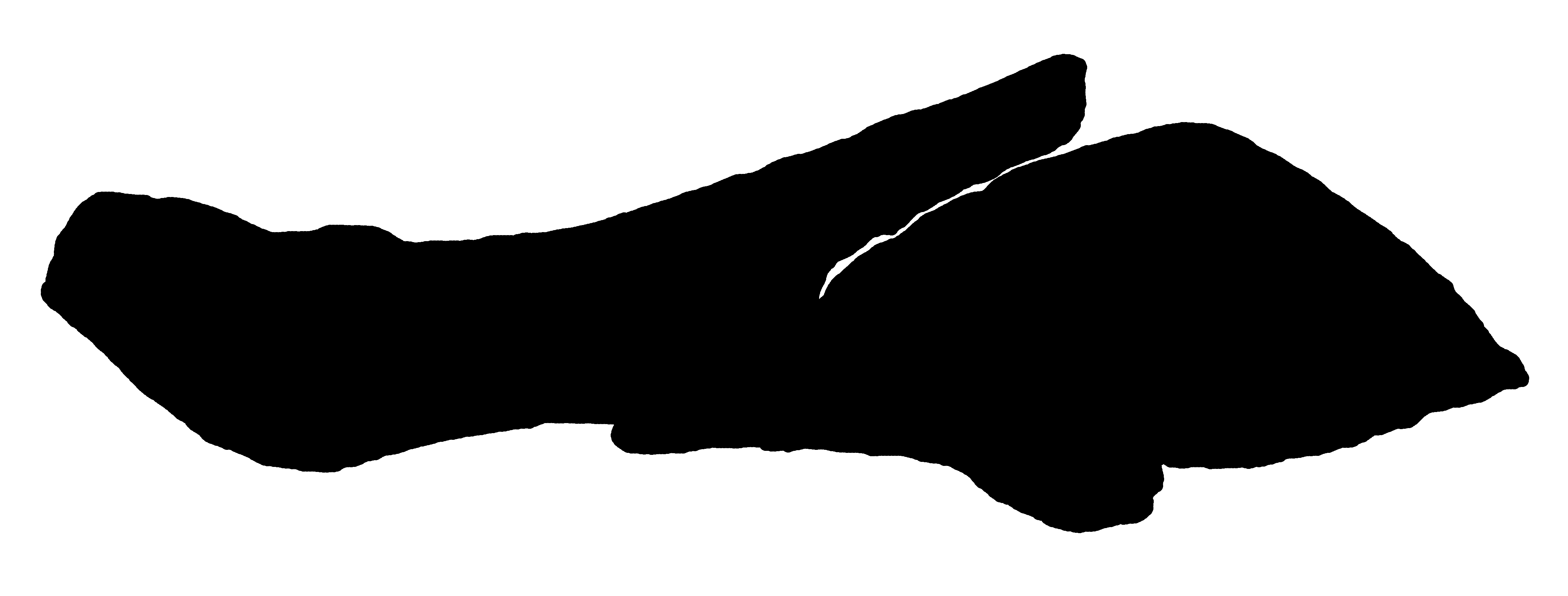

Supplement: Supplemental Information 6 [file peerj-13-20243-s006.zip › SUPPLEMENTARY FILE 7 Code_R2/Code shape lateral/Silhouette_lateral/Sauroctonus_progressus.jpg]

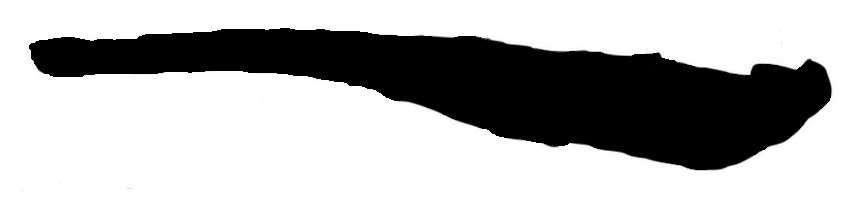

Supplement: Supplemental Information 6 [file peerj-13-20243-s006.zip › SUPPLEMENTARY FILE 7 Code_R2/Code shape lateral/Silhouette_lateral/Australerpeton_cosgriffi.jpg]

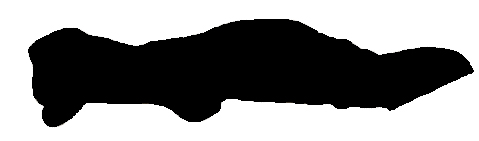

Supplement: Supplemental Information 6 [file peerj-13-20243-s006.zip › SUPPLEMENTARY FILE 7 Code_R2/Code shape occlusal/Silhouette_occlusal/Dicynodon_lacerticeps.jpg]

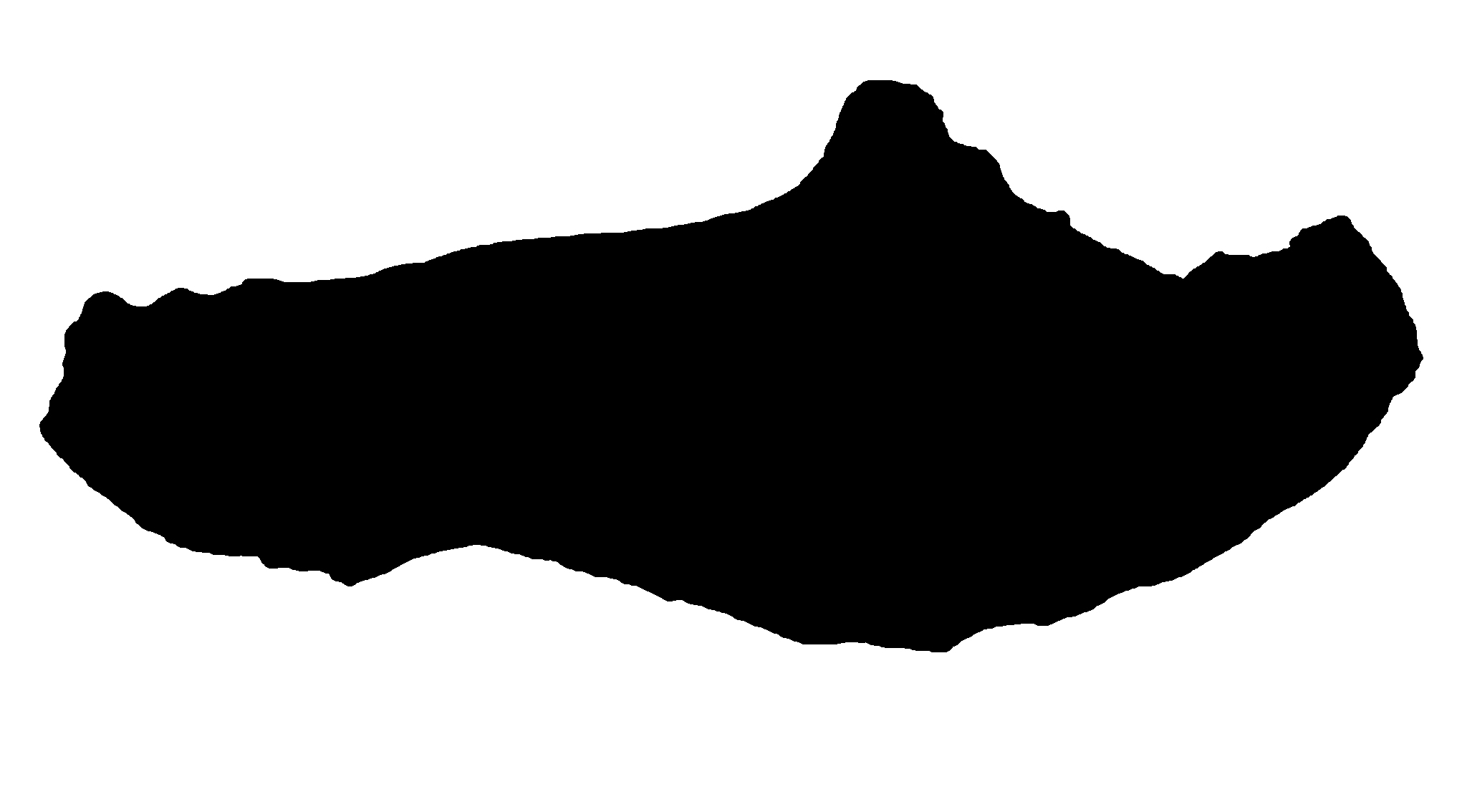

Supplement: Supplemental Information 6 [file peerj-13-20243-s006.zip › SUPPLEMENTARY FILE 7 Code_R2/Code shape lateral/Silhouette_lateral/Desmatodon_hesperis.jpg]

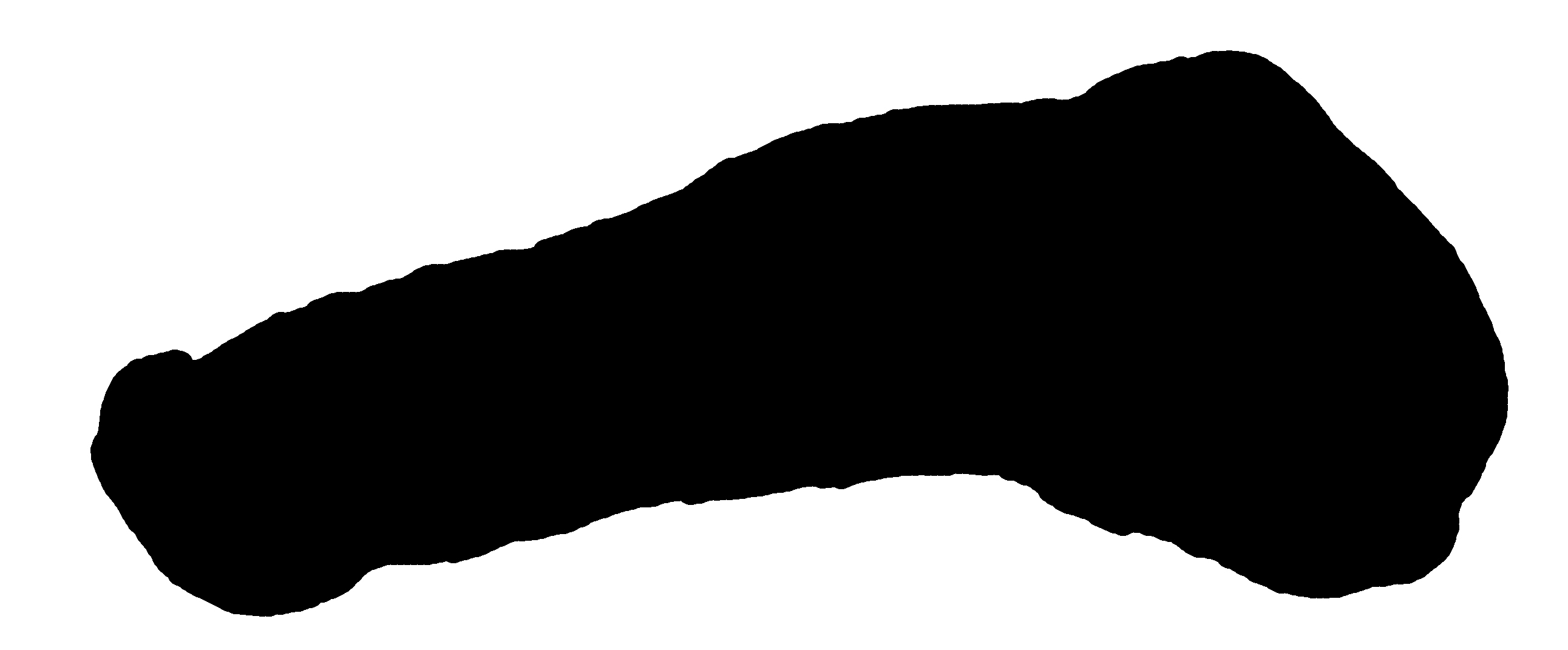

Supplement: Supplemental Information 6 [file peerj-13-20243-s006.zip › SUPPLEMENTARY FILE 7 Code_R2/Code shape lateral/Silhouette_lateral/Moschops_capensis.jpg]

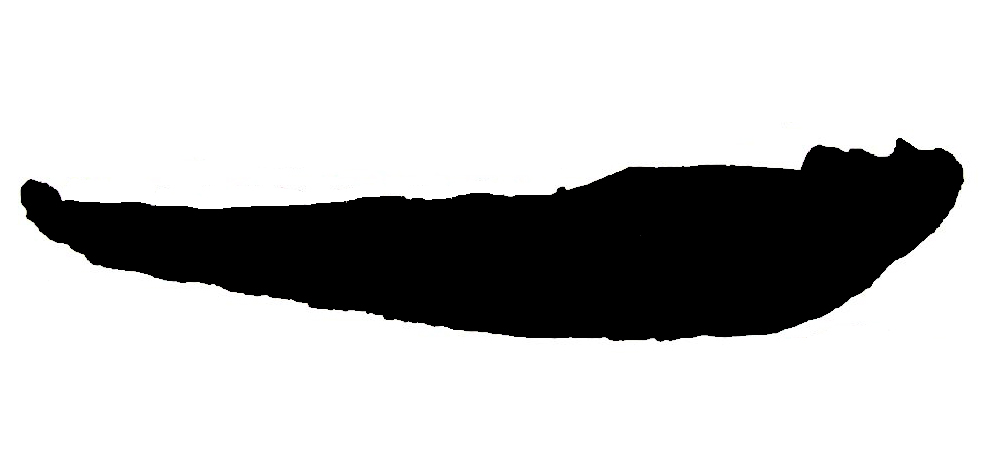

Supplement: Supplemental Information 6 [file peerj-13-20243-s006.zip › SUPPLEMENTARY FILE 7 Code_R2/Code shape lateral/Silhouette_lateral/Rastosuchus_hammeri.jpg]

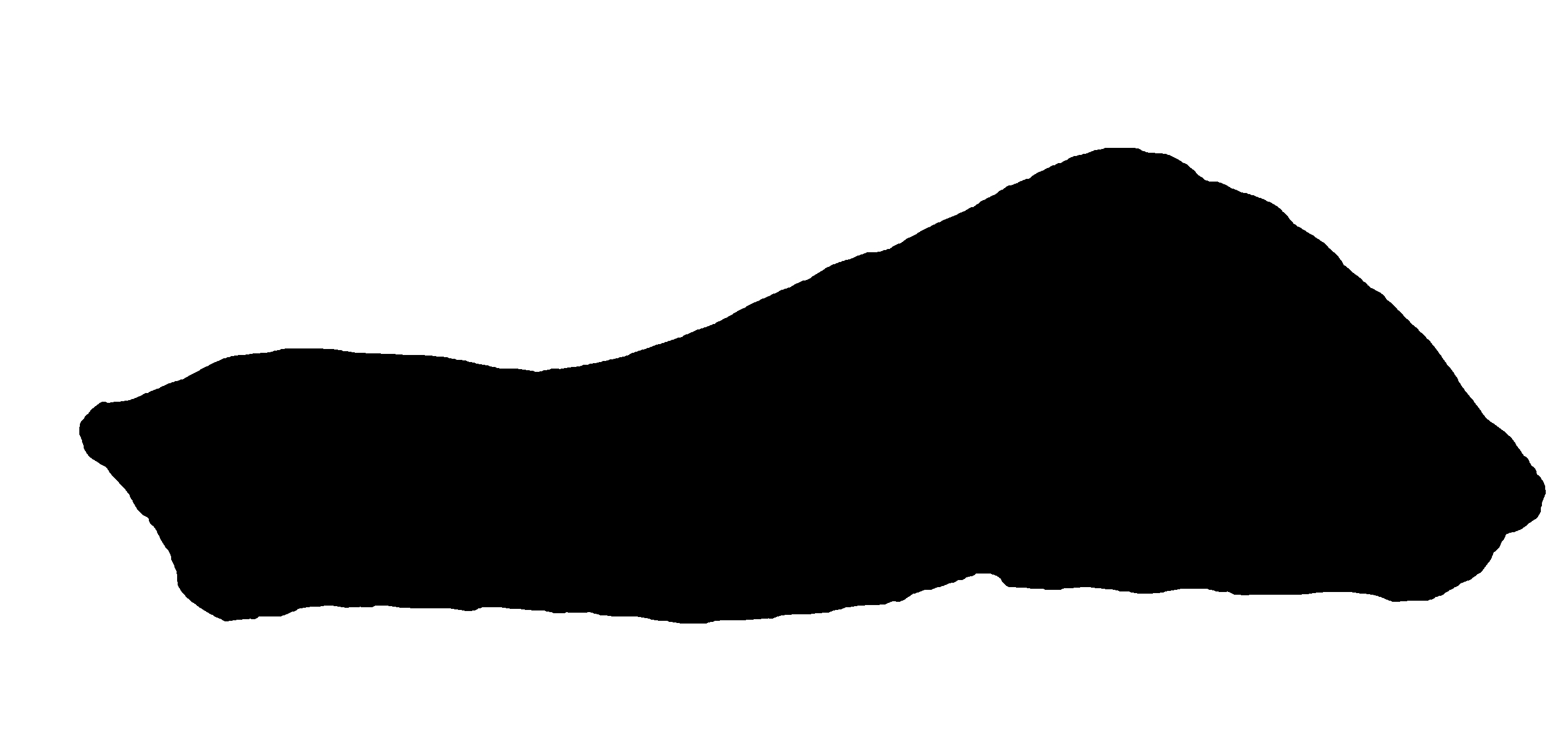

Supplement: Supplemental Information 6 [file peerj-13-20243-s006.zip › SUPPLEMENTARY FILE 7 Code_R2/Code shape lateral/Silhouette_lateral/Estemmenosuchus_mirabilis.jpg]

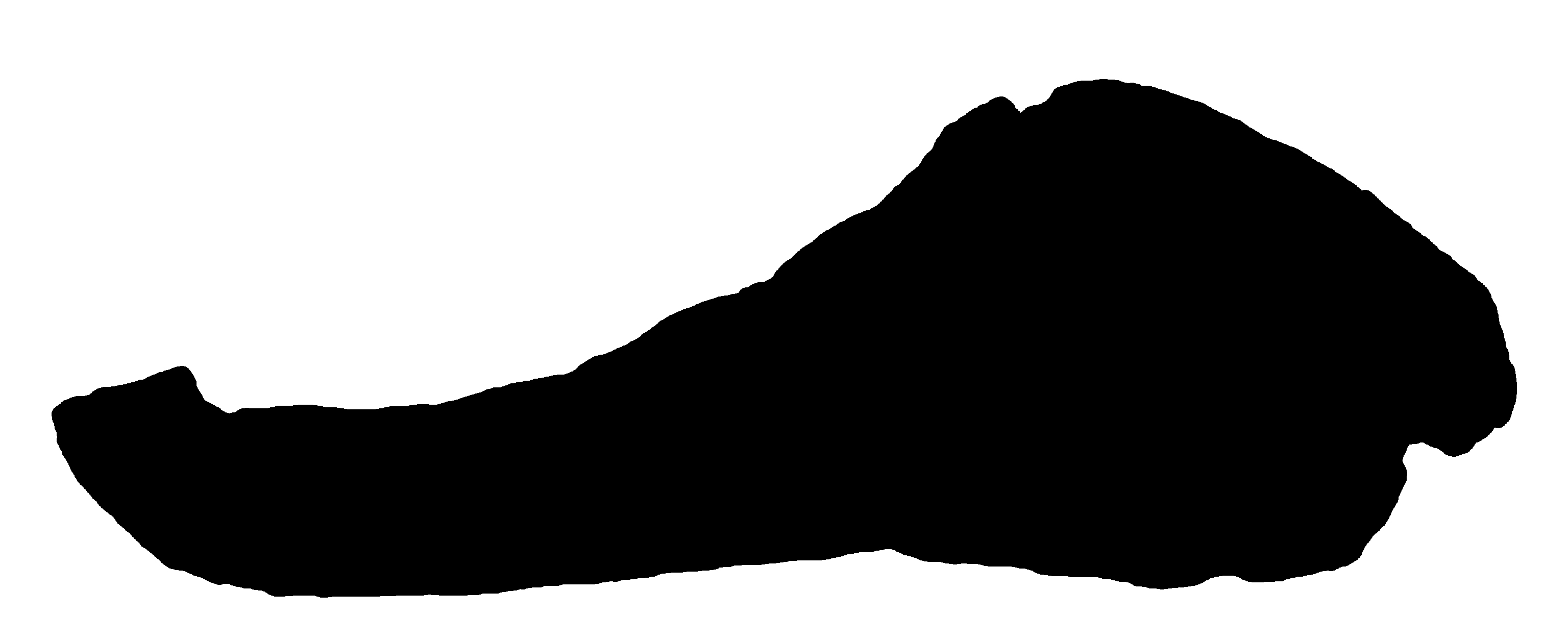

Supplement: Supplemental Information 6 [file peerj-13-20243-s006.zip › SUPPLEMENTARY FILE 7 Code_R2/Code shape lateral/Silhouette_lateral/Sphenacodon_ferox.jpg]

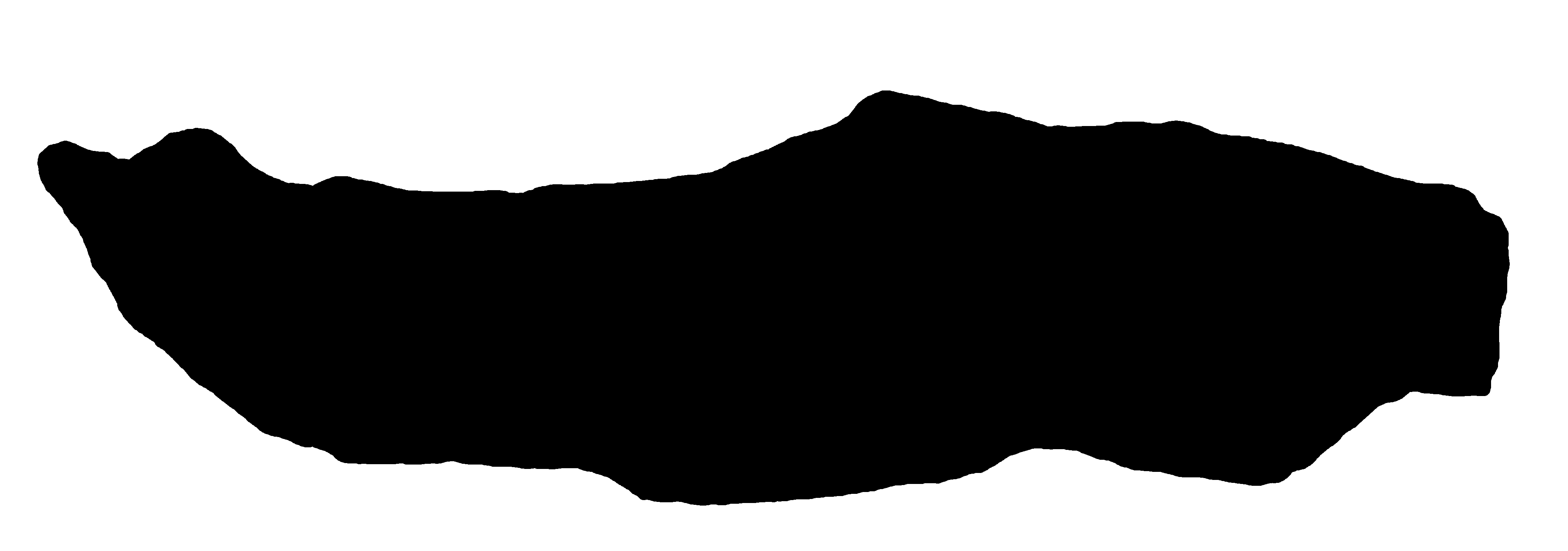

Supplement: Supplemental Information 6 [file peerj-13-20243-s006.zip › SUPPLEMENTARY FILE 7 Code_R2/Code shape lateral/Silhouette_lateral/Tapinocaninus_pamelae.jpg]

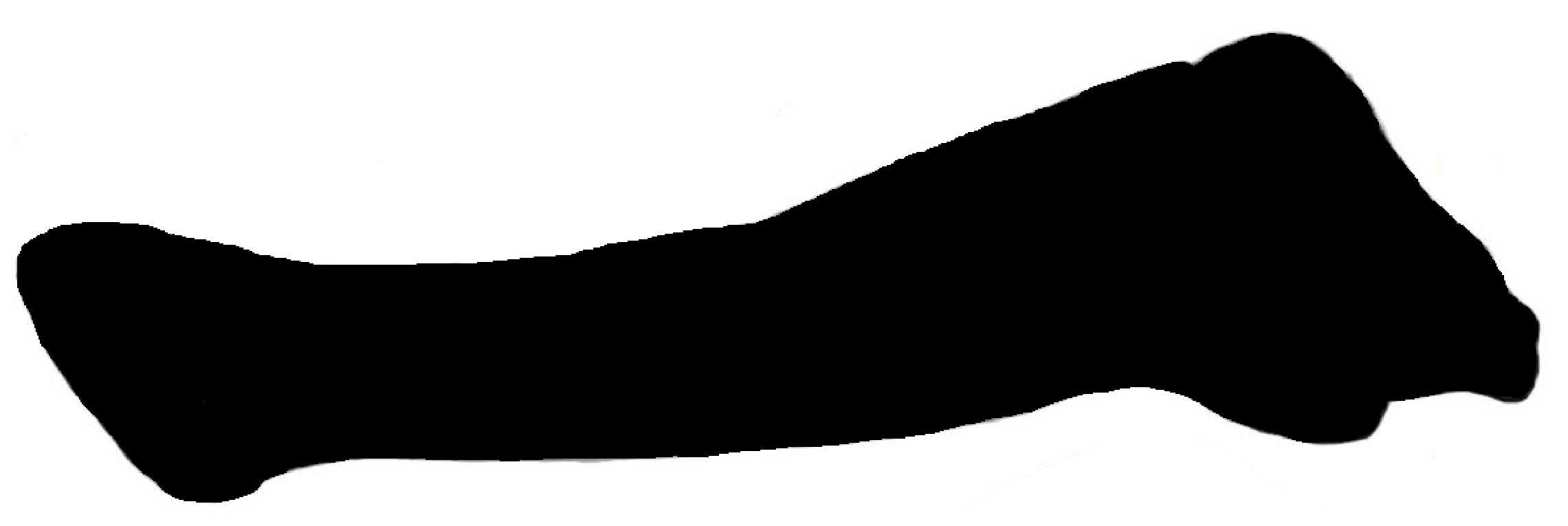

Supplement: Supplemental Information 6 [file peerj-13-20243-s006.zip › SUPPLEMENTARY FILE 7 Code_R2/Code shape lateral/Silhouette_lateral/Syodon_efremovi.jpg]

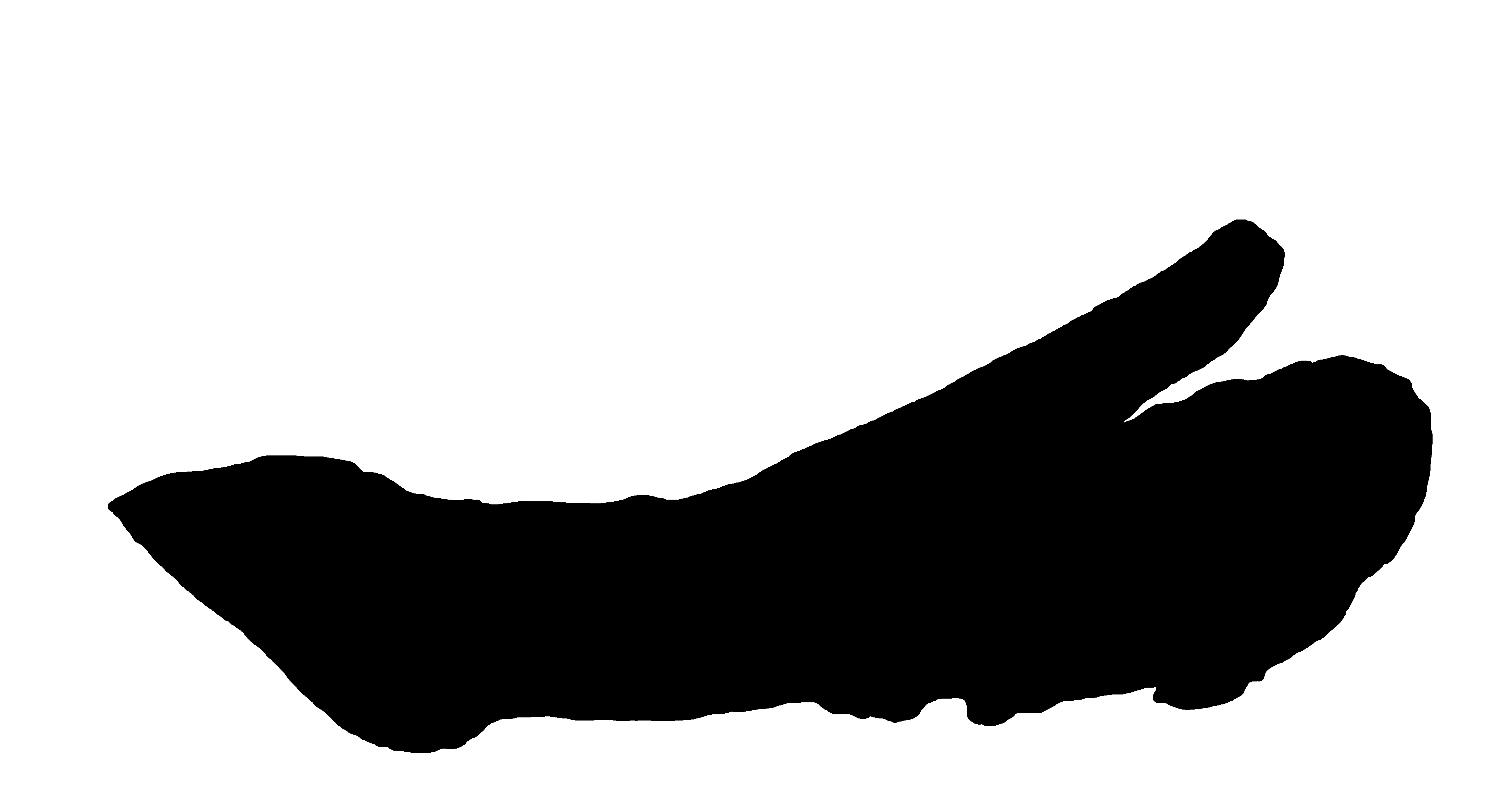

Supplement: Supplemental Information 6 [file peerj-13-20243-s006.zip › SUPPLEMENTARY FILE 7 Code_R2/Code shape lateral/Silhouette_lateral/Aelurosaurus_felinus.jpg]

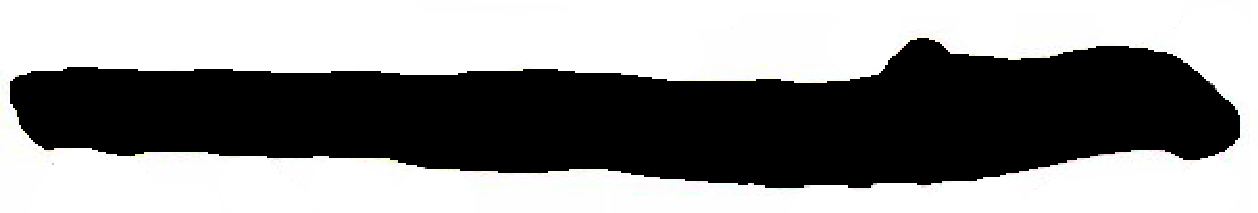

Supplement: Supplemental Information 6 [file peerj-13-20243-s006.zip › SUPPLEMENTARY FILE 7 Code_R2/Code shape lateral/Silhouette_lateral/Youngina_capensis.jpg]

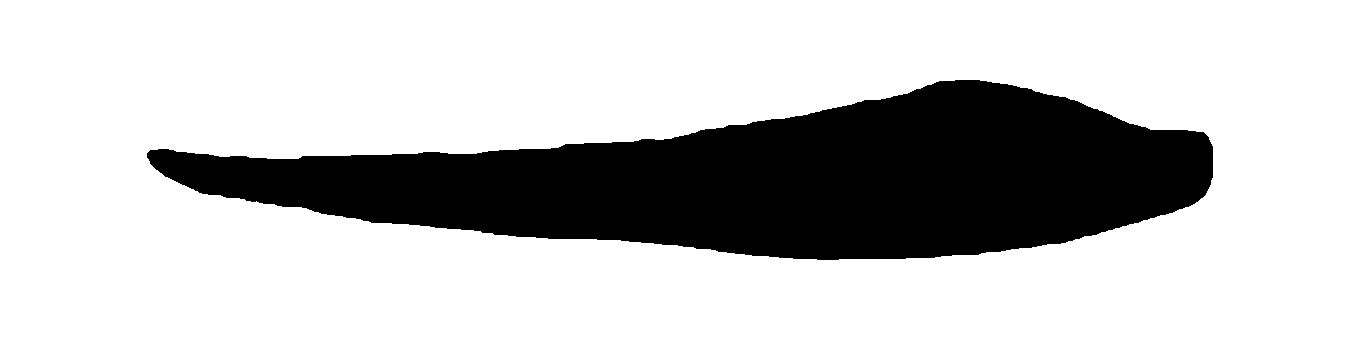

Supplement: Supplemental Information 6 [file peerj-13-20243-s006.zip › SUPPLEMENTARY FILE 7 Code_R2/Code shape lateral/Silhouette_lateral/Brouffia_orientalis.jpg]

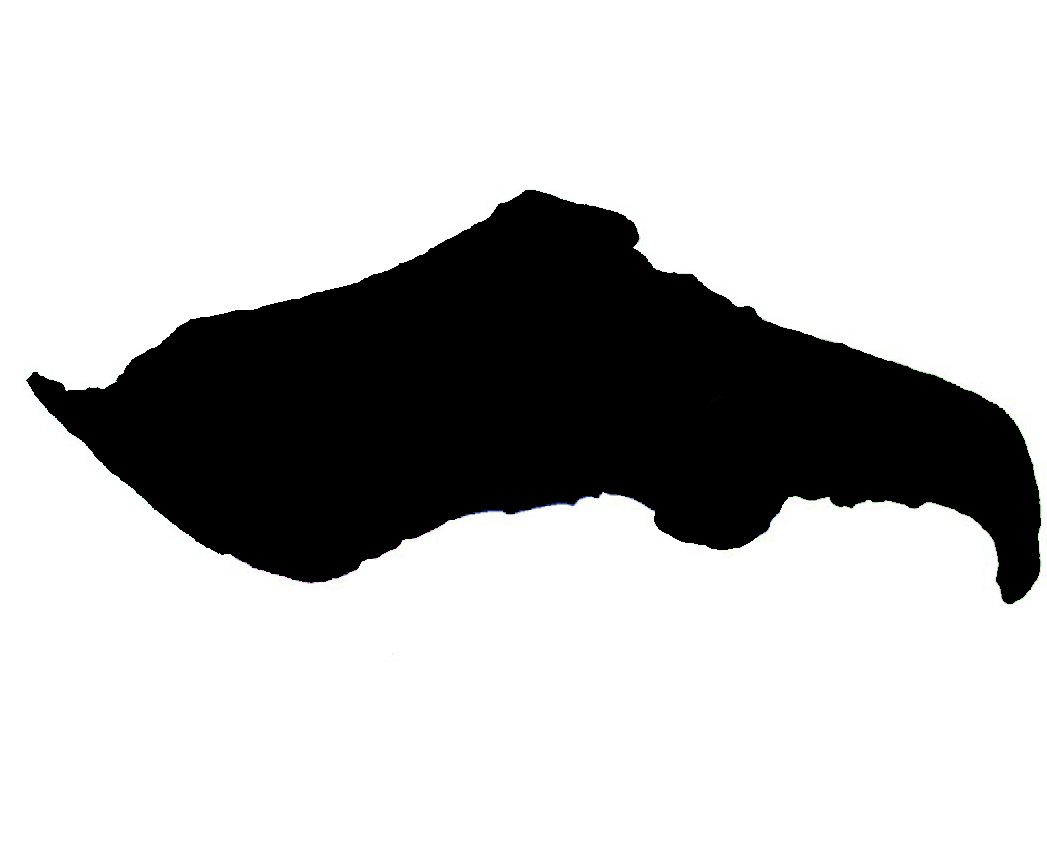

Supplement: Supplemental Information 6 [file peerj-13-20243-s006.zip › SUPPLEMENTARY FILE 7 Code_R2/Code shape lateral/Silhouette_lateral/Niassodon_mfumukasi.jpg]

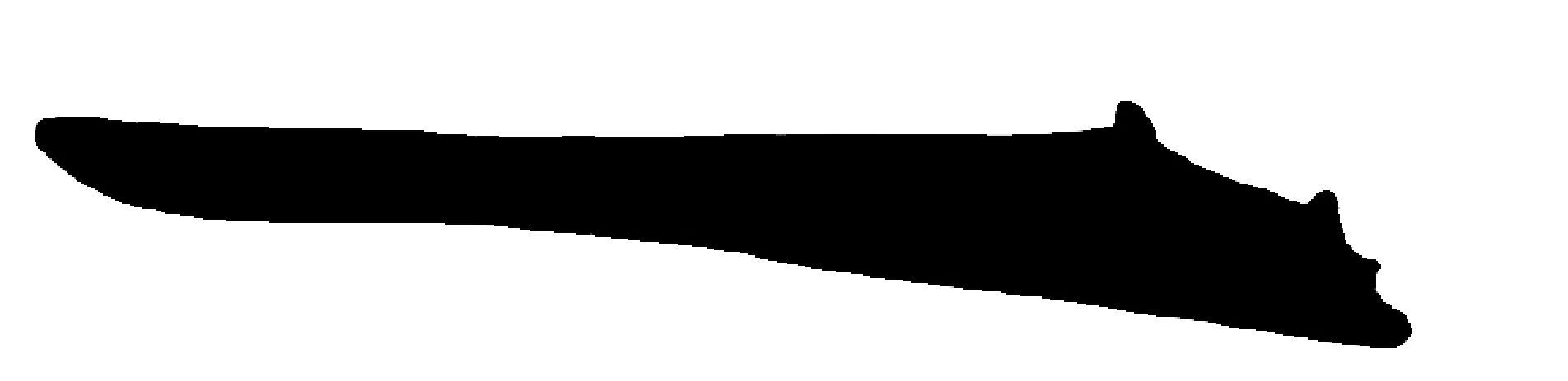

Supplement: Supplemental Information 6 [file peerj-13-20243-s006.zip › SUPPLEMENTARY FILE 7 Code_R2/Code shape lateral/Silhouette_lateral/Weigeltisaurus_jaekeli.jpg]

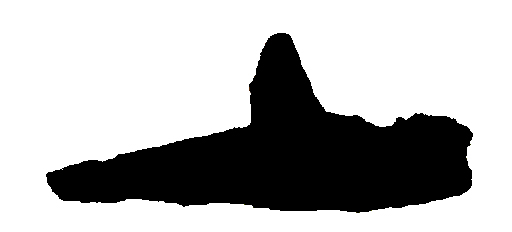

Supplement: Supplemental Information 6 [file peerj-13-20243-s006.zip › SUPPLEMENTARY FILE 7 Code_R2/Code shape lateral/Silhouette_lateral/Bolosaurus_major.jpg]

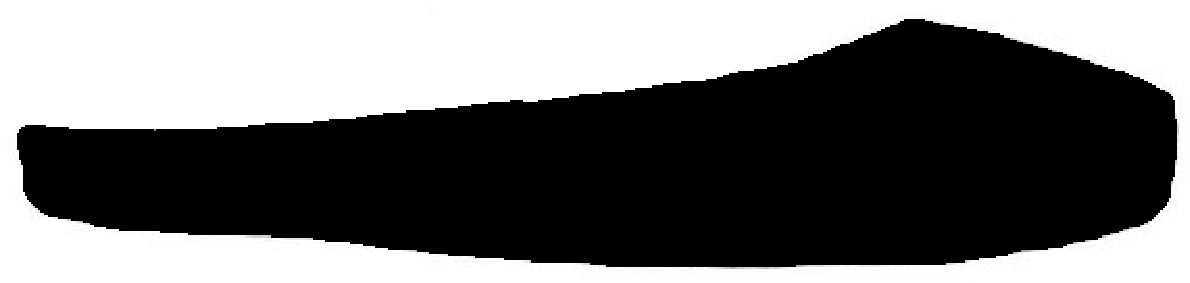

Supplement: Supplemental Information 6 [file peerj-13-20243-s006.zip › SUPPLEMENTARY FILE 7 Code_R2/Code shape lateral/Silhouette_lateral/Spinarerpeton_brevicephalum.jpg]

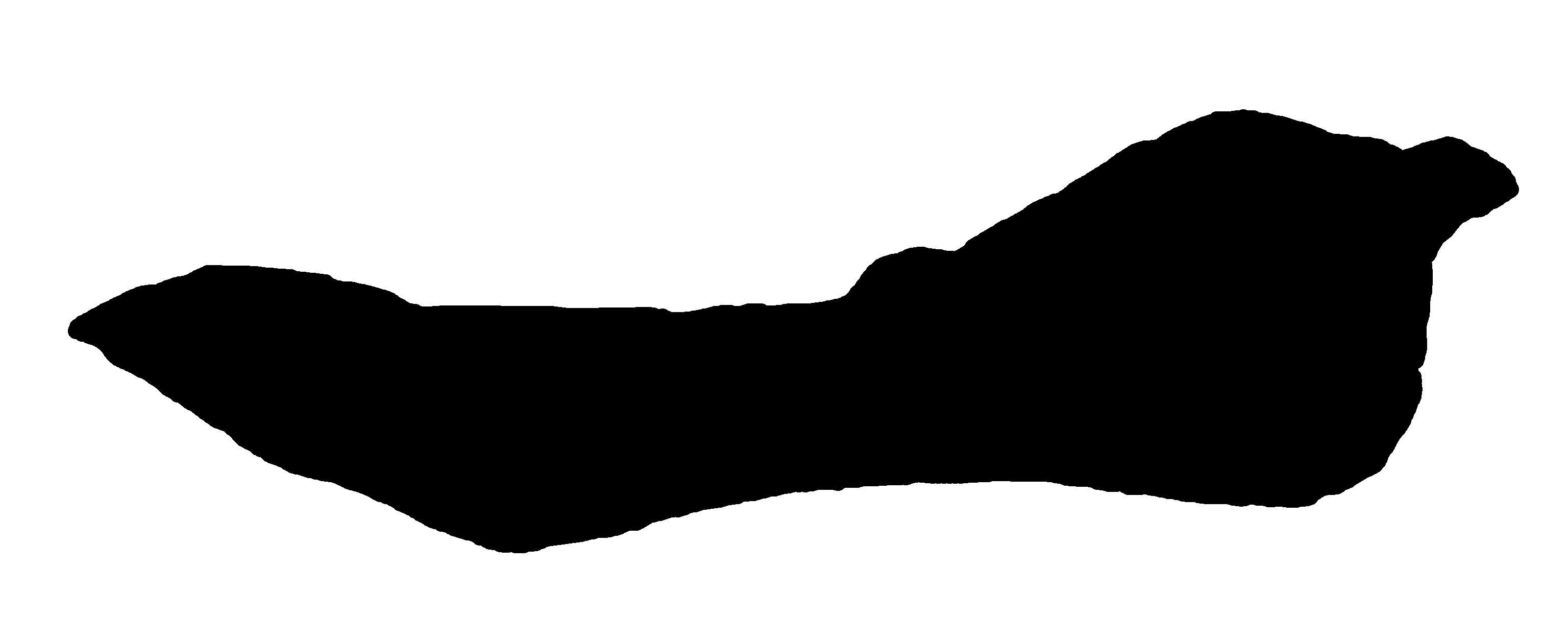

Supplement: Supplemental Information 6 [file peerj-13-20243-s006.zip › SUPPLEMENTARY FILE 7 Code_R2/Code shape lateral/Silhouette_lateral/Scylacosaurus_sclateri.jpg]

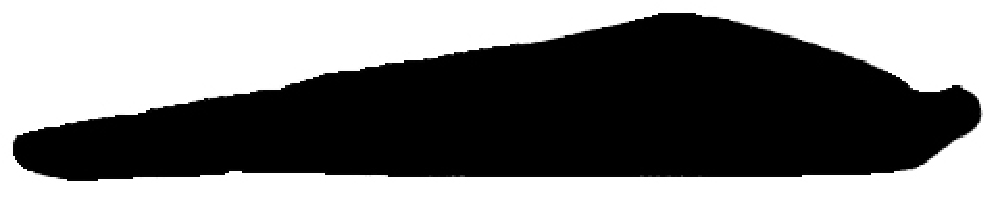

Supplement: Supplemental Information 6 [file peerj-13-20243-s006.zip › SUPPLEMENTARY FILE 7 Code_R2/Code shape lateral/Silhouette_lateral/Westlothiana_lizziae.jpg]

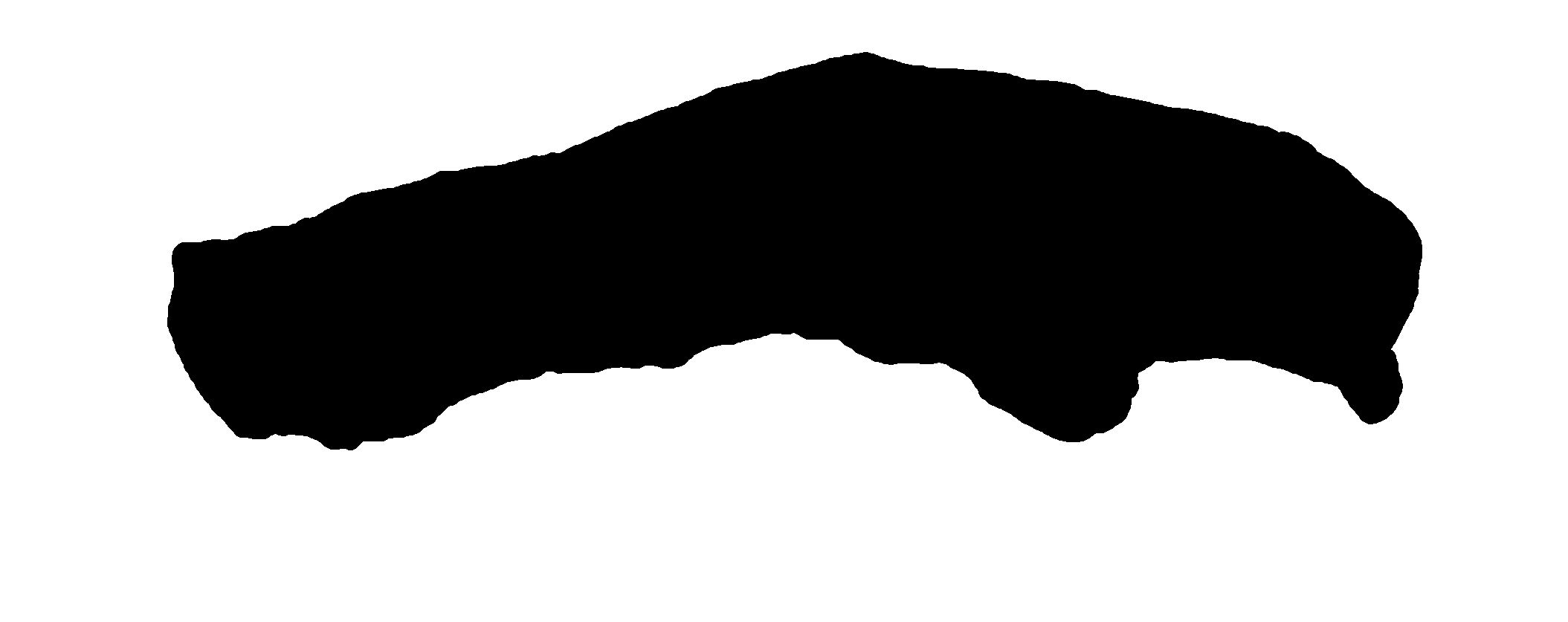

Supplement: Supplemental Information 6 [file peerj-13-20243-s006.zip › SUPPLEMENTARY FILE 7 Code_R2/Code shape lateral/Silhouette_lateral/Patranomodon_nyaphulii.jpg]

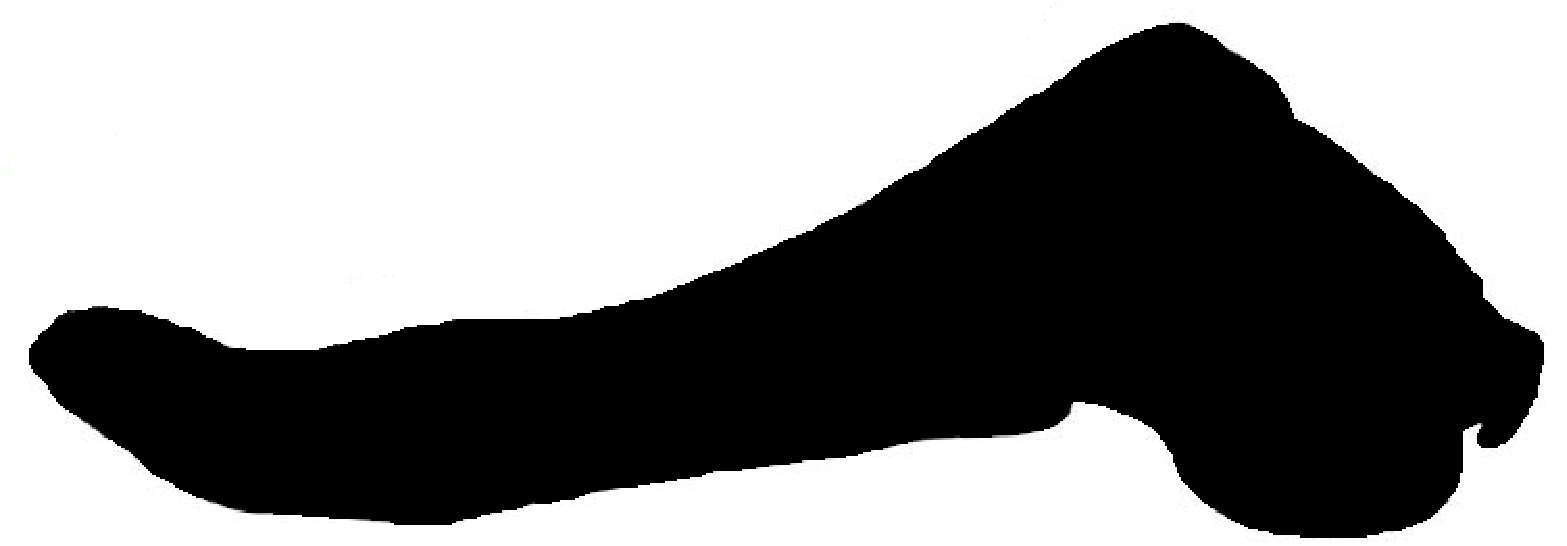

Supplement: Supplemental Information 6 [file peerj-13-20243-s006.zip › SUPPLEMENTARY FILE 7 Code_R2/Code shape lateral/Silhouette_lateral/Viatkosuchus_sumini.jpg]

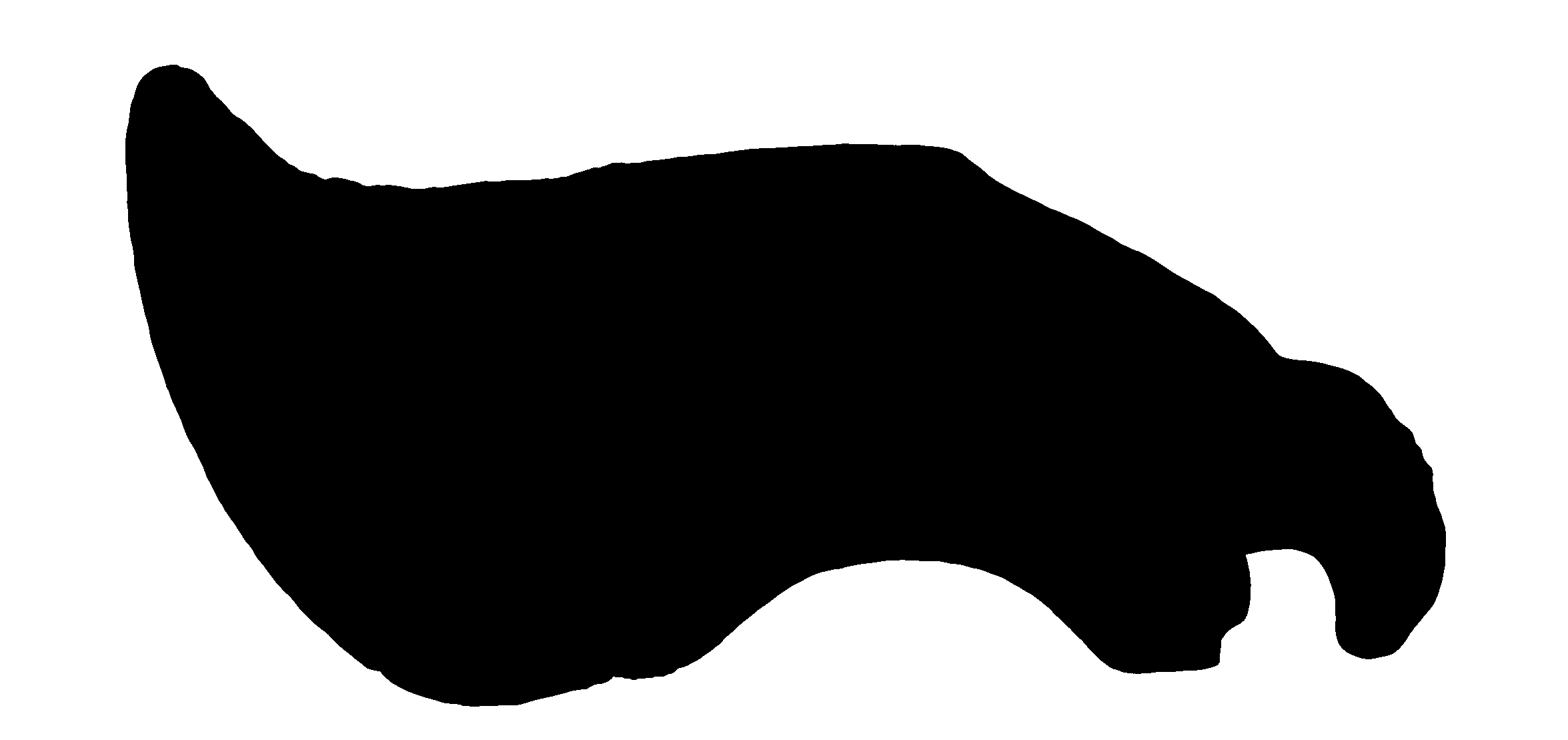

Supplement: Supplemental Information 6 [file peerj-13-20243-s006.zip › SUPPLEMENTARY FILE 7 Code_R2/Code shape lateral/Silhouette_lateral/Odontocyclops_whaitsi.jpg]

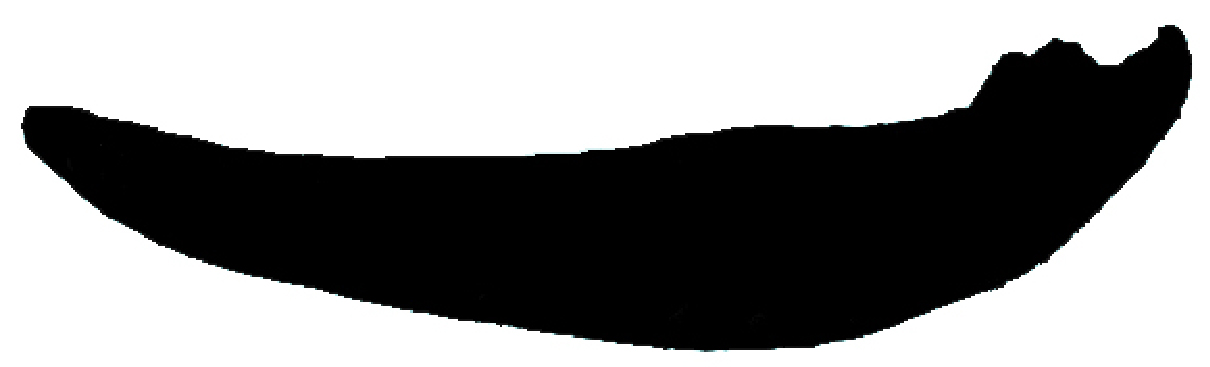

Supplement: Supplemental Information 6 [file peerj-13-20243-s006.zip › SUPPLEMENTARY FILE 7 Code_R2/Code shape lateral/Silhouette_lateral/Whatcheeria_deltae.jpg]

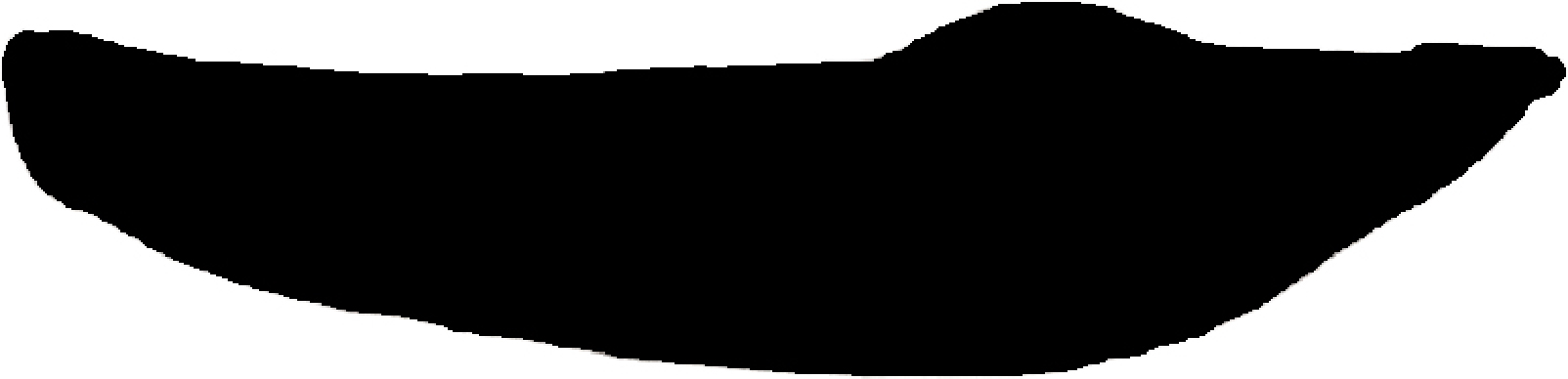

Supplement: Supplemental Information 6 [file peerj-13-20243-s006.zip › SUPPLEMENTARY FILE 7 Code_R2/Code shape lateral/Silhouette_lateral/Tseajaia_campi.jpg]

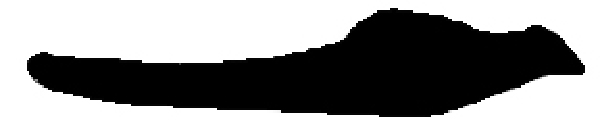

Supplement: Supplemental Information 6 [file peerj-13-20243-s006.zip › SUPPLEMENTARY FILE 7 Code_R2/Code shape lateral/Silhouette_lateral/Tramuntanasaurus_tiai.jpg]

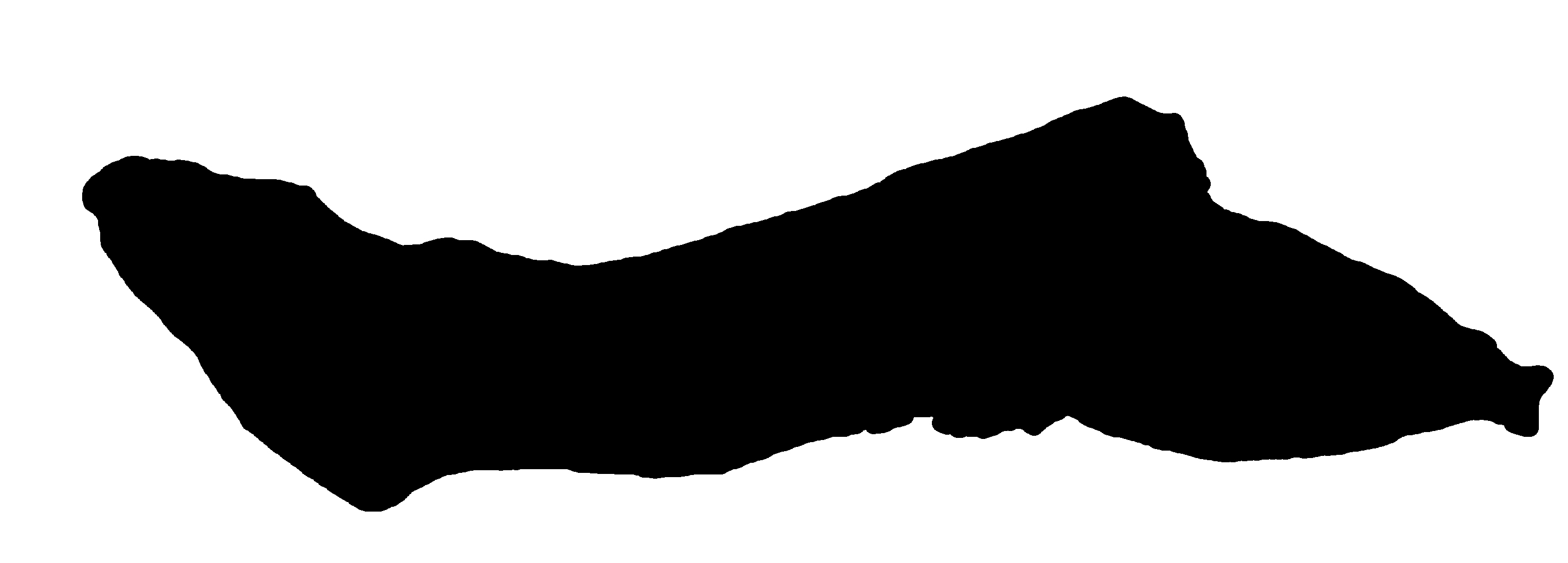

Supplement: Supplemental Information 6 [file peerj-13-20243-s006.zip › SUPPLEMENTARY FILE 7 Code_R2/Code shape lateral/Silhouette_lateral/Delphaciognathus_paucidens.jpg]
